# Supplementary material for: Antibiotic knowledge, attitudes and practices: new insights from cross-sectional rural health behaviour surveys in low-income and middle-income South-East Asia
Source: BMJ Open. 2019 Aug 20;9(8):e028224. doi: 10.1136/bmjopen-2018-028224 (PMC6707701; doi:10.1136/bmjopen-2018-028224)
Supplement: Supplementary data [file bmjopen-2018-028224supp001.pdf]

Supplemental Material

Figure A1. Trend of AMR-related social sciences and multidisciplinary publications, 1970–2017.

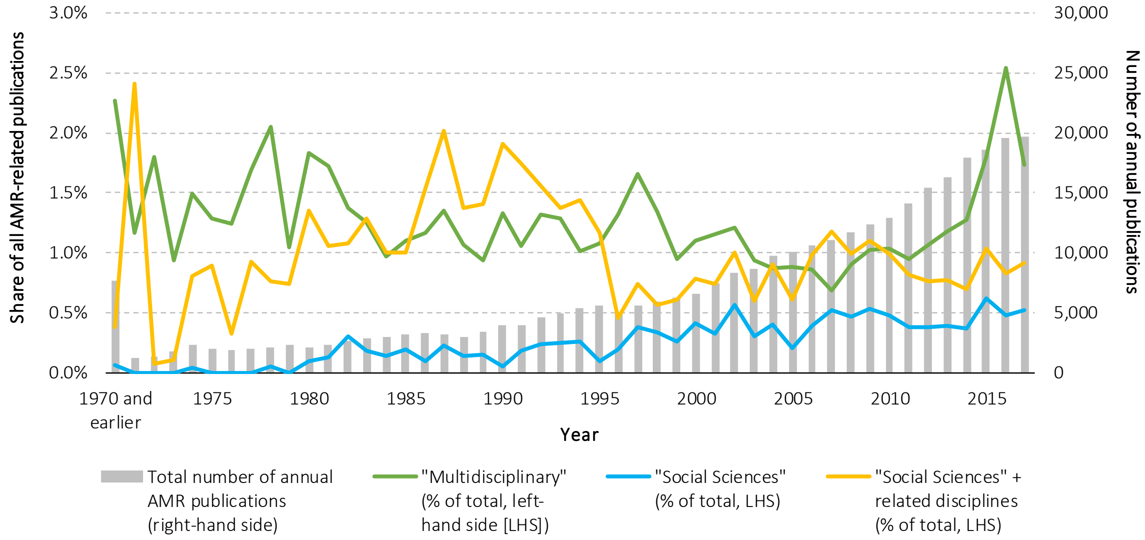

Source: Authors, based on Elsevier B.V. [15].
Notes: Data until 2018, as of 9 October 2018. Disciplines as reported by Scopus database, with "'Social Sciences' + related disciplines" providing upper bound of social science publications including Arts and Humanities;" "Business, Management and Accounting;" "Decision Sciences;" "Economics, Econometrics and Finance;" and "Psychology" alongside "Social Sciences." Based on search query [TITLE-ABS-KEY ("antibiotic resistance" OR "drug resistance" OR "antimicrobial resistance" OR "AMR")]. Total number of publications as of 9 October 2018 was 347,511, of which 66.0% arose from "Medicine;" 28.3% from "Biochemistry, Genetics and Molecular Biology;" and 18.2% from "Immunology and Microbiology" (multiple categories per publication possible.) "Social Sciences" average during the period was 0.4% (0.9% for "'Social Sciences' + related disciplines"). LHS=left-hand side.

Table A1. Variable descriptions.

	Variable	Description
**Demographic attributes**	Female	Binary variable: Sex of respondent (R); [1] if female.
	Age	Continuous variable: Age in years.
	Education	Continuous variable: Completed years of formal education.
	Speaking Thai / Lao	Binary variable: [1] if R reported ability to communicate in main language (irrespective of reading and writing).
	Wealth index	Continuous variable: Average of 17 household assets and amenities on scale from [0] to [1].
	Buddhist religion	Binary variable: [1] if R belongs to the majority religion (Buddhism in both sites).
	Thai/Lao nationality	Binary variable: [1] if R has Thai (Chiang Rai) or Lao (Salavan) nationality.
	Majority ethnic group	Binary variable: [1] if R belongs to the majority ethnic group Thai (Chiang Rai) or Lao Loum (Salavan).
**Antibiotic knowledge / attitudes**	Aware of antibiotics	Binary variable: [1] if R recognised images of antibiotic capsules that are common in the field site and, if not, the most common translation of antibiotics as "anti-inflammatory drug" ("ยาแก้อักเสบ" or "*yah kae ak seb*") in Thai and "germ resister" ("ຢາຕ້ານເຊື້ອ" or "*yah dtan suea*") in Lao. Additional categorical variables (coded ex ante and ex post) recorded the names and purposes that the respondent reported following recognition of the medicine.
	Aware of drug resistance	Binary variable: [1] if R recognised the local terms for "drug resistance." In Thai, "drug resistance" was translated as "ดื้อยา" ("*due yah*"). Lao has two translations of which "ດື້ຢາ" ("*due yah*") is the formal term and "ລື້ງຢາ" ("*lueng yah*") is a more colloquial but broader expression (both translations were asked separately). Additional categorical variables (coded ex ante and ex post) recorded the interpretations of each term.
	Would not buy antibiotics over the counter	Binary variable: [1] if answer to question "Is there any situation for which you would buy this medicine?" corresponded to FAO/OIE/WHO message "When using antibiotics: follow professional advice" (field coded based on survey training manual) [1].
	Prefers alternatives over antibiotics	Binary variable: [1] if answer to question "Do you prefer other remedies such as herbs or cough syrup to this medicine for sore throat?" corresponded to FAO/OIE/WHO message "Ensure medicines are only used when necessary" (field coded) [1].
	Does not keep antibiotics for future use	Binary variable: [1] if answer to question "If you were prescribed this medicine by a doctor and did not finish the course, would you keep it for future use?" corresponded to FAO/OIE/WHO message "When using antibiotics: never share medicines or use leftover drugs to treat a different illness" (field coded) [1].
	Knows that antibiotic resistance can spread	Binary variable: [1] if answer to question "Can your '*due yah*' (drug resistance) spread to other people, for example if you sneeze on them?" corresponded to FAO/OIE/WHO message "antimicrobial resistance can affect us all" (field coded) [1].
	Answer score	Continuous variable: Number of preceding answers ([0] to [4]) corresponding to FAO/OIE/WHO material [1].
**Illness episodes**	Self-rated severity	Ordinal variable: [1] if illness is reported as "mild;" [2] as "moderate;" [3] as "severe."
	Duration of illness episode	Continuous variable: Total duration of illness episode in days, calculated as sum of duration of individual steps in episode.
**Treatment-seeking behaviour**	Public healthcare provider	Binary variable: [1] if R reported accessing health centre or hospital during illness episode.
	Private healthcare provider	Binary variable: [1] if R reported accessing private clinic, hospital, or pharmacy.
	Informal healthcare provider	Binary variable: [1] if R reported accessing grocery store or traditional healer.
	Care from family or self-care	Binary variable: [1] if R reported self-treatment or care from family member or friend.
	Other types of healthcare access	Binary variable: [1] if R reported accessing other healthcare provider (e.g. village health volunteer).
	Medicines use episodes	Continuous variable: Number of reported medicine use episodes per illness, categorised into types of medicine (coded ex post into non-antibiotic medicine, antibiotics, and potential antibiotics) and sources of access (public, private, informal healthcare provider). Note that the actual amount of medicine used during each "medicine use episode" is likely to vary systematically across formal and informal healthcare providers, with "episodes" from the latter typically containing only a small number of pills and capsules for immediate treatment of symptoms.

Source: Authors.

Table A2. Correlation matrixes of independent variables in regression models.

	**Would not buy antibiotics over the counter**	**Prefers antibiotics over alternatives**	**Does not keep antibiotics for future use**	**Knows that antibiotic resistance can spread**	**Aware of antibiotics**	**Aware of drug resistance**	**Female**	**Age**	**Education (years)**	**Speaking Thai / Lao**	**Wealth index**	**Buddhist religion**	**Thai/Lao nationality**	**Majority ethnic group (Thai/Lao Loum)**	**Self-rated severity**	**Log of duration of illness episode (days)**
**Chiang Rai**																
**Would not buy antibiotics over the counter**	1.00															
**Prefers antibiotics over alternatives**	0.05	1.00														
**Does not keep antibiotics for future use**	0.25***	0.16**	1.00													
**Knows that antibiotic resistance can spread**	0.01	-0.03	0.02	1.00												
**Aware of antibiotics**	0.03	0.05	0.00	0.07	1.00											
**Aware of drug resistance**	-0.02	0.02	-0.06	0.09	0.17***	1.00										
**Female**	0.13	-0.02	-0.02	-0.02	-0.03	-0.01	1.00									
**Age**	0.00	-0.03	0.10	0.03	-0.09	-0.11	-0.03	1.00								
**Education (years)**	0.02	0.10	-0.01	0.03	0.13	0.37***	-0.18***	-0.50***	1.00							
**Speaking Thai / Lao**	0.04	0.04	0.04	0.02	0.23***	0.32***	-0.06	-0.08	0.26***	1.00						
**Wealth index**	0.05	0.09	-0.03	0.04	0.07	0.28***	-0.08	0.12	0.29***	0.33***	1.00					
**Buddhist religion**	-0.04	0.02	-0.03	0.08	0.02	0.26***	0.00	0.17***	0.19***	0.19***	0.27***	1.00				
**Thai/Lao nationality**	0.04	0.03	0.01	0.04	0.12	0.16**	-0.06	0.02	0.15**	0.32***	0.22***	0.24***	1.00			
**Majority ethnic group (Thai/Lao Loum)**	-0.02	0.04	0.00	0.04	0.12	0.33***	-0.03	0.21***	0.26***	0.32***	0.43***	0.59***	0.27***	1.00		
**Self-rated severity**	0.04	0.09	0.02	-0.01	0.03	-0.01	0.00	-0.02	-0.01	-0.03	-0.01	-0.14*	0.05	-0.15**	1.00	
**Log of duration of illness episode (days)**	0.02	0.06	-0.03	-0.04	0.03	0.05	0.09	0.04	-0.07	-0.04	0.00	-0.02	0.02	-0.06	0.36***	1.00
**Salavan**																
**Would not buy antibiotics over the counter**	1.00															
**Prefers antibiotics over alternatives**	-0.16	1.00														
**Does not keep antibiotics for future use**	-0.01	0.01	1.00													
**Knows that antibiotic resistance can spread**	0.00	0.03	0.10	1.00												
**Aware of antibiotics**	-0.23***	0.04	-0.07	0.01	1.00											
**Aware of drug resistance**	-0.19**	0.13	-0.03	0.14	0.30***	1.00										
**Female**	-0.01	-0.08	0.02	0.06	-0.06	0.06	1.00									
**Age**	-0.03	0.03	0.02	0.03	0.13	0.15	-0.09	1.00								
**Education (years)**	-0.13	0.13	-0.02	0.14	0.20*	0.36***	-0.23***	-0.19**	1.00							
**Speaking Thai / Lao**	-0.27***	0.15	0.03	0.01	0.26***	0.25***	-0.28***	0.11	0.34***	1.00						
**Wealth index**	-0.33***	0.16	0.04	-0.06	0.28***	0.38***	-0.02	0.08	0.40***	0.36***	1.00					
**Buddhist religion**	-0.42***	0.17	0.02	0.00	0.29***	0.48***	0.05	0.15	0.26***	0.44***	0.62***	1.00				
**Thai/Lao nationality**	0.05	-0.07	0.07	0.02	-0.05	-0.02	-0.02	-0.19**	0.11	-0.05	-0.03	-0.07	1.00			
**Majority ethnic group (Thai/Lao Loum)**	-0.32***	0.16	0.03	0.03	0.30***	0.53***	0.07	0.16	0.32***	0.36***	0.55***	0.76***	0.03	1.00		
**Self-rated severity**	0.19**	0.02	0.07	0.01	-0.12	-0.02	0.11	-0.01	-0.02	-0.08	-0.05	-0.13	0.10	-0.08	1.00	
**Log of duration of illness episode (days)**	0.13	0.07	0.04	0.04	-0.02	-0.04	0.11	0.07	-0.08	-0.07	0.02	-0.05	0.10	-0.09	0.21***	1.00

Source: Authors' analysis of survey data.
Notes: Unweighted statistics.
*p < 0.1, **p < 0.05, ***p < 0.01.

Table A3. Characteristics of individuals who received antibiotics from public, private, and informal sources.

	Chiang Rai			X^2 / z-score[c]			Salavan			X^2 / z-score[c]		
	**Public antibiotic access**	**Private antibiotic access**	**Informal antibiotic access**	**Publ. vs. priv.**	**Publ. vs. inf.**	**Priv. vs. inf.**	**Public antibiotic access**	**Private antibiotic access**	**Informal antibiotic access**	**Publ. vs. priv.**	**Publ. vs. inf.**	**Priv. vs. inf.**
Number	93	115	35	200	110	132	157	38	41	179	178	77
**Demographics**												
Female	53.8% (40.3–66.8)	59.6% (46.6–71.4)	56.9% (34.2–77.1)	0.54	0.11	0.05	71.5% (63.8–78.2)	41.0% (23.5–61.1)	71.8% (51.2–86.1)	9.13***	0.07	6.63**
Age	43 (13)	48 (14)	40 (11)	1.41	0.55	3.54***	35 (19)	33 (12)	39 (18)	0.42	0.96	0.94
Education (years)	6.2 (4.9)	5.8 (5.2)	8.0 (3.8)	0.30	1.18	2.14**	4.7 (6.3)	8.1 (5.9)	5.9 (5.5)	4.75***	0.81	1.48
Speaking Thai / Lao	92.3% (81.9–96.9)	85% (73.7–92)	98% (86.1–99.7)	2.04	1.50	4.63**	92.7% (89.3–95.1)	100.0% (100.0–100.0)	100.0% (100.0–100.0)	7.80***	4.96**	..
Wealth index	0.6 (0.1)	0.7 (0.1)	0.7 (0.1)	2.42**	3.48***	2.52**	0.4 (0.2)	0.5 (0.1)	0.5 (0.2)	3.86***	0.86	1.27
Buddhist religion	78.5% (64.8–87.8)	77.2% (63.9–86.6)	89.0% (74.4–95.8)	0.03	1.44	2.14	66.5% (58.3–73.8)	78.9% (52.9–92.5)	79.2% (58.9–91.0)	1.05	2.61	<0.01
Thai/Lao nationality	97.1% (89.8–99.2)	95.9% (88.8–98.5)	98% (86.1–99.7)	0.14	0.03	0.28	99% (92.9–99.9)	100.0% (100.0–100.0)	97.6% (84.0–99.7)	0.38	0.66	24.41***
Majority ethnic group (Thai/Lao Loum)	58.8% (45.3–71.1)	64.6% (52.3–75.3)	81.9% (65.8–91.4)	0.60	7.36***	2.84*	58.6% (48.9–67.7)	69.9% (45.6–86.6)	68.2% (44.4–85.2)	0.71	0.67	0.03
**Antibiotic knowledge / attitudes**												
Aware of antibiotics	97.5% (90.4–99.4)	95.7% (88.6–98.4)	93.3% (64.5–99.1)	0.42	2.70	0.24	88.7% (82.3–92.9)	92.7% (72.2–98.4)	100.0% (100.0–100.0)	1.21	5.50**	2.32
Aware of drug resistance[a]	75.0% (62.5–84.3)	76.5% (64.9–85.1)	90.2% (76.4–96.4)	0.06	3.35*	3.63*	66.3% (57.8–73.8)	84.7% (61.1–95.1)	79.5% (61.3–90.5)	2.94*	2.22	0.18
Links drug resistance to AMR concepts[b]	1.7% (0.3–9.5)	5.4% (2.0–13.6)	1.3% (0.2–9.0)	1.39	0.01	5.17**	4.4% (2.0–9.3)	9.0% (1.9–33.5)	13.4% (5.6–28.7)	0.89	4.92**	0.27
Would not buy antibiotics over the counter	62.0% (48.2–74.1)	62.1% (48.3–74.2)	42.0% (22.0–65.0)	<0.01	4.19**	4.44**	44.5% (35.9–53.5)	18.3% (9.3–32.7)	23.3% (12.3–39.6)	13.92***	24.99***	0.62
Prefers antibiotics over alternatives	62.7% (49.6–74.2)	50.2% (36.8–63.6)	70.3% (46.2–86.7)	2.48	1.17	3.21*	22.8% (16.8–30.2)	33.7% (19.5–51.7)	29.3% (13.7–52.0)	2.52	0.37	0.36
Does not keep antibiotics for future use	54.3% (41.2–66.8)	53.9% (41.7–65.6)	38.1% (18.9–62.0)	<0.01	0.88	3.35*	21.1% (14.2–30.2)	14.0% (4.6–35.4)	13.9% (5.2–32.1)	0.52	2.07	<0.01
Knows that antibiotic resistance can spread	8.1% (3.9–16.2)	10.9% (5.7–19.9)	6.5% (1.6–23.2)	0.51	0.25	0.53	1.9% (0.5–6.6)	1.8% (0.2–12.4)	0.0% (0.0–0.0)	<0.01	1.90	0.95
Answer score (0 to 4)	1.9 (0.9)	1.8 (0.9)	1.6 (0.8)	0.54	1.72*	1.05	0.9 (1.1)	0.7 (0.7)	0.7 (0.6)	1.86*	1.87*	0.05

Source: Authors' analysis of survey data.

Notes: Including antibiotics and unconfirmed medicines that may include antibiotics. Illness-level data, including only completed illnesses experienced by respondent or a child under their supervision. Population-weighted statistics, accounting for complex survey design. Multiple types of healthcare access per individual and illness episode possible.

a. Comparing Thai "due yah" with the combined Lao "due yah" and "lueng yah."

b. Corresponding to interpretation of "drug resistance" as "Reference to antibiotics, drug-resistant germs" in Exhibit 6.

c. X^2 and Wilcoxon rank-sum tests, omitting simultaneous antibiotic access from more than one type of healthcare provider, which was the case in 82/958 [8.6%] of all pairwise comparisons of antibiotic access.
