# Supplementary material for: Antibiotic knowledge, attitudes and practices: new insights from cross-sectional rural health behaviour surveys in low-income and middle-income South-East Asia
Source: BMJ Open. 2019 Aug 20;9(8):e028224. doi: 10.1136/bmjopen-2018-028224 (PMC6707701; doi:10.1136/bmjopen-2018-028224)
Supplement: Supplementary data [file bmjopen-2018-028224supp003.pdf]

OxTREC reference: 528-17 **ANTIBIOTICS AND ACTIVITY SPACES**

INTERVIEW GUIDES – COGNITIVE INTERVIEWS FOR SURVEY TESTING

บทสัมภาษณ์ – การสัมภาษณ์กระบวนการคิดเพื่อการทดสอบแบบสอบถาม

ບົດສຳພາດ - ການສຳພາດຂະບວນການຄິດເພື່ອການທົດສອບຄຳຖາມ

Objective วัตถุประสงค์ ຈຸດປະສົງ

To understand how survey participants understand the survey questions and arrive at their answers. เพื่อเรียนรู้ว่าผู้เข้าร่วมงานวิจัยมีการทำความเข้าใจคำถามที่ใช้ในแบบสอบถามอย่างไร และมีความคิดอย่างไรก่อนตัดสินใจตอบคำถามแต่ละข้อ ເພື່ອຮຽນຮູ້ວ່າຜູ້ເຂົ້າຮ່ວມການຄົ້ນຄ້ວາມີຄວາມເຂົ້າໃຈຄຳຖາມທີ່ໃຊ້ໃນແບບສອບຖາມແບບໃດ ແລະມີຄວາມຄິດແບບໃດກ່ອນຕັດສິນໃຈຕໍ່ຄຳຖາມແຕ່ລະຂໍ້

Introduction บทนำ ບົດນຳ

<Obtaining verbal informed consent in accordance with Oral Consent Script> <ดำเนินการขอคำยินยอมโดยใช้ข้อความสำหรับอธิบายงานวิจัยและขอคำยินยอมเข้าร่วมการวิจัยด้วยวาจา> <ດຳເນີນການຂໍຄຳຍິນຍອມໂດຍໃຊ້ຂໍ້ຄວາມສຳລັບອະທິບາຍວຽກຄົ້ນຄ້ວາແລະຂໍຄຳຍິນຍອມເຂົ້າຮ່ວມການຄົ້ນຄ້ວາດ້ວຍວາຈາ>

Thank you for agreeing to participate in this study. ขอบคุณท่านสำหรับการตกลงเข้าร่วมการวิจัยครั้งนี้ ຂອບໃຈທ່ານສຳລັບການຕົກລົງເຂົ້າຮ່ວມການຄົ້ນຄ້ວາໃນຄັ້ງນີ້

Part I – About Yourself ส่วนที่ 1 – เกี่ยวกับตัวท่าน ສ່ວນທີ່ 1 - ກ່ຽວກັບຕົວທ່ານ

Let us start with a few questions about yourself. เราจะเริ่มโดยถามคำถามเกี่ยวกับข้อมูลส่วนตัวของท่าน ເຮົາຈະເລີ່ມໂດຍຖາມຄຳຖາມກ່ຽວກັບຂໍ້ມູນສ່ວນຕົວຂອງທ່ານ

Guiding question: Who is my informant? คำถามนำ: ผู้ให้สัมภาษณ์คือใคร ຄຳຖາມນຳ: ຜູ້ໃຫ້ສຳພາດແມ່ນໃຜ

1. How old are you? อายุของท่าน ອາຍຸທ່ານ
2. What is your level of education? การศึกษาสูงสุดของท่าน ການສຶກສາສູງສຸດຂອງທ່ານ
3. What is your ethnicity and nationality? เชื้อชาติ และ สัญชาติของท่าน ເຊື້ອຊາດແລະສັນຊາດຂອງທ່ານ

Part II – Practicing the "Think Aloud" Technique ส่วนที่ 2 – เทคนิคการคิดออกเสียง (การบรรยายกระบวนการคิด) ສ່ວນທີ່ 2 - ເທັກນິກການຄິດອອກສຽງ (ການບັນລະຍາຍຂະບວນການຄິດ)

Thank you. As we are trying to improve our survey, you can help us develop the questionnaire by participating in this interview. I would like to ask you to describe your thoughts as you think about the question and the answer as though you are talking to yourself. For example, what do you think as you receive the question? What does the question make you think of? In what way do you want answer to question? How do you arrive at your answer? Let me give you an example so you understand this type of interview better.

Let's say I was asked a question about "What do you see when you go from your home to the nearest temple?" If I was thinking out loud, I would say "[interviewer describing their own village]." So you can see how it works, though that was only one of many ways. The important thing is that there are no right or wrong answers. What really matters is your view and thought process. And this is what I would like you to share with me. So now it is your turn to practice.

ขอบคุณครับ/ค่ะ เนื่องจากเรากำลังอยู่ในช่วงปรับปรุงแบบสอบถาม ท่านสามารถช่วยเราพัฒนาแบบสอบถามนี้ได้โดยการเข้าร่วมการสัมภาษณ์นี้ ซึ่งเราจะขอให้ท่านบรรยายความคิดของท่านในขณะที่ท่านพิจารณาคำถามและคำตอบเสมือนกับท่านกำลังคุยกับตนเอง เช่น ท่านคิดอย่างไรเมื่อท่านได้รับคำถาม คำถามทำให้ท่านนึกถึงอะไรบ้าง ท่านต้องการตอบคำถามเหล่านี้อย่างไร ท่านได้คำตอบมาได้อย่างไร เราจะยกตัวอย่างให้ท่านหนึ่งตัวอย่างเพื่อให้ท่านสามารถเข้าใจการสัมภาษณ์ในลักษณะนี้มากขึ้น

ยกตัวอย่าง ถ้าเราต้องตอบคำถามที่ว่า "ท่านมองเห็นอะไรบ้างหากท่านเดินทางจากบ้านไปวัดที่ใกล้ที่สุด" และเราต้องบรรยายความคิดของเราให้ท่านฟัง เราจะพูดว่า "[ผู้สัมภาษณ์บรรยายการเดินทางในหมู่บ้านของตนเอง]" ซึ่งเป็นเพียงแค่หนึ่งในหลากหลายวิธีที่ท่านสามารถใช้ได้ สิ่งที่สำคัญที่สุดเกี่ยวกับการคิดออกเสียงของท่านคือคำตอบของท่านจะไม่ถูกตัดสินว่าถูกหรือผิด เพราะสิ่งที่เราต้องการเรียนรู้มากที่สุดคือมุมมองและวิธีการคิดของท่าน และนั่นคือสิ่งที่เราอยากให้ท่านแบ่งปันกับเรา ดังนั้นเราจึงขอให้ท่านลองคิดออกเสียงกับเราในคำถามต่อไปนี้

Haenssgen MJ, *et al. BMJ Open* 2019; 9:e028224. doi: 10.1136/bmjopen-2018-028224

ຂອບໃຈ ເນື່ອງຈາກເຮົາກຳລັງຢູ່ໃນຊ່ວງປັບປຸງແບບສອບຖາມ ທ່ານສາມາດຊ່ວຍເຮົາພັດທະນາແບບສອບຖາມນີ້ໄດ້ໂດຍການເຂົ້າຮ່ວມການສຳພາດນີ້ ເຊິ່ງເຮົາຈະຂໍໃຫ້ທ່ານບັນລະຍາຍຄວາມຄິດຂອງທ່ານໃນຕອນທີ່ທ່ານກຳລັງພິຈາລະນາຄຳຖາມແລະຄຳຕອບຄືກັບທ່ານກຳລັງລົມກັບຕົນເອງ ເຊັ່ນ ທ່ານຄິດແນວໃດເມື່ອທ່ານໄດ້ຮັບຄຳຖາມ ຄຳຖາມເຮັດໃຫ້ເຮົາຄິດຫາຫຍັງແດ່ ທ່ານຕ້ອງການຕອບຄຳຖາມເຫຼົ່ານີ້ແນວໃດ ທ່ານໄດ້ຄຳຕອບມາໄດ້ແນວໃດ ເຮົາຈະຍົກຕົວຢ່າງໃຫ້ທ່ານໜຶ່ງຕົວຢ່າງເພື່ອໃຫ້ທ່ານສາມາດເຂົ້າໃຈການສຳພາດໃນລັກຊະນະນີ້ຫຼາຍຂຶ້ນ.

ຍົກຕົວຢ່າງ ຖ້າເຮົາຕ້ອງຕອບຄຳຖາມວ່າ ທ່ານເບິ່ງເຫັນຫຍັງແດ່ຖ້າທ່ານເດີນທາງຈາກເຮືອນຫາວັດທີ່ໃກ້ທີ່ສຸດ ແລະເຮົາຕ້ອງບັນລະຍາຍຄວາມຄິດຂອງເຮົາໃຫ້ທ່ານຟັງເຮົາຈະເວົ້າວ່າ "[ຜູ້ສຳພາດບັນຍາຍການເດີນທາງໃນໝູ່ບ້ານຂອງຕົນເອງ]" ເຊິ່ງເປັນພຽງໜຶ່ງໃນຫຼາກຫຼາຍວິທີທີ່ທ່ານສາມາດໃຊ້ໄດ້ ສິ່ງທີ່ສຳຄັນທີ່ສຸດກ່ຽວກັບການຄິດອອກສຽງຂອງທ່ານຄືຄຳຕອບຂອງທ່ານຈະບໍ່ຖືກຕັດສິນວ່າຖືກຫຼືຜິດ ເພາະສິ່ງທີ່ເຮົາຕ້ອງການຮຽນຮູ້ຫຼາຍທີ່ສຸດມຸມມອງແລະວິທີການຄິດຂອງທ່ານ ແລະນັ້ນແມ່ນສິ່ງທີ່ເຮົາຢາກໃຫ້ທ່ານແບ່ງປັນກັບເຮົາ ດັ່ງນັ້ນເຮົາຈຶ່ງຂໍໃຫ້ທ່ານລອງຄິດອອກສຽງກັບເຮົາໃນຄຳຖາມຕໍ່ໄປນີ້

Guiding question: Is the respondent comfortable with the "think aloud" technique? คำถามนำ: ผู้ให้สัมภาษณ์สามารถใช้เทคนิคการคิดออกเสียงหรือไม่ ຄຳຖາມນຳ: ຜູ້ໃຫ້ສຳພາດໃຊ້ເຕັກນິກການຄິດອອກສຽງຫຼືບໍ່

4. I would like you to imagine your village. Could you please describe to me what you see when you go from your home to the nearest temple, in as much detail as you can? เราขอให้ท่านนึกภาพหมู่บ้านของท่านและบรรยายให้เราฟังว่าท่านเห็นอะไรเมื่อท่านเดินทางจากบ้านของท่านไปยังวัดที่ใกล้ที่สุด โดยใช้รายละเอียดมากที่สุดเท่าที่ท่านสามารถทำได้ ເຮົາຂໍໃຫ້ທ່ານນຶກພາບໝູ່ບ້ານຂອງທ່ານແລະບັນຍາຍໃຫ້ເຮົາຟັງວ່າທ່ານເຫັນຫຍັງເມື່ອທ່ານເດີນທາງຈາກເຮືອນຂອງທ່ານໄປຫາວັດທີ່ໃກ້ທີ່ສຸດ ໂດຍໃຊ້ລາຍລະອຽດຫຼາຍທີ່ສຸດທີ່ທ່ານສາມາດເຮັດໄດ້

Before we start, I'd like to give you another example that is more closely related to the actual questions from our questionnaire. Say you ask me to list all persons age ≥18 years in my household with their sex and age, I'd go [*interviewer answering question about adults in household*]. *ก่อนที่เราจะเริ่ม เราขอยกตัวอย่างที่ใกล้เคียงกับคำถามจริงกับแบบสอบถามของเราให้ท่านอีกหนึ่งครั้ง สมมติท่านขอให้เราบอกข้อมูลของสมาชิกอายุ 18 ปีหรือมากกว่า ที่อาศัยอยู่ในครัวเรือนนี้ทุกคน รวมถึงเพศและอายุ เราจะพูดว่า "[ผู้สัมภาษณ์ให้คำตอบเกี่ยวกับสมาชิกในครัวเรือนของตนเอง]." ກ່ອນທີ່ເຮົາຈະເລີ່ມເຮົາຂໍຍົກຕົວຢ່າງທີ່ຄ້າຍກັນກັບແບບສອບຖາມຂອງເຮົາ ສົມມຸດທ່ານບອກໃຫ້ເຮົາບອກຂໍ້ມູນຂອງຊະມະຊິກອາຍຸ 18 ປີຫຼືຫຼາຍກ່ວາ ທີ່ອາໄສຢູ່ໃນຄົວເຮືອນນີ້ທຸກຄົນ ລວມເຖິງເພດແລະອາຍຸ ເຮົາຈະເວົ້າວ່າ "[ຜູ້ສຳພາດໃຫ້ຄຳຕອບກ່ຽວກັບສະມາຊິກໃນຄົວເຮືອນຂອງຕົນເອງ]"*

List all persons aged 18+ years in household กรอกข้อมูลของสมาชิกอายุ 18 ปีหรือมากกว่า ที่อาศัยอยู่ในครัวเรือนนี้ ໃສ່ຂໍ້ມູນຂອງຊະມະຊິກອາຍຸ 18 ປີຫຼືຫຼາຍກ່ວາ ທີ່ອາໃສຢູ່ຄົວເຮືອນນີ້

[*1 respondent per every 5 household members will be selected randomly from this list*] [*ตัวแทน 1 คนต่อสมาชิกจำนวน 5 คน จะถูกสุ่มเลือกให้เข้าร่วมการสัมภาษณ์*] [*ຕົວແທນ 1 ຄົນຕໍ່ຊະມະຊິກຈຳນວນ 5 ຄົນ ຈະຖືກສຸ່ມເລືອກໃຫ້ເຂົ້າຮ່ວມການສຳພາດ*]

Name ชื่อ ຊື່	Sex เพศ ເພດ<br>(M / F) (ช/ญ) (ຊ/ຍ)	Age อายุ ອາຍຸ<br>(in completed Years) (จำนวนปีเต็ม) (ຈຳນວນປີເຕັມ)	Available for interview today ผู้เข้าร่วมสามารถให้สัมภาษณ์ในวันนี้หรือไม่ ຜູ້ເຂົ້າຮ່ວມສາມາດໃຫ້ສຳພາດໃນມື້ນີ້ໄດ້ຫຼືບໍ່?<br>(Yes / No) (ใช่/ไม่ใช่) (ແມ່ນ/ບໍ່ແມ່ນ)

Part III – Social-Network-Related Survey Questions ส่วนที่ 3 – คำถามเกี่ยวกับเครือข่ายสังคม ສ່ວນທີ່ 3 - ຄຳຖາມກ່ຽວກັບເຄືອຂ່າຍສັງຄົມ

I will now ask you some questions about your interactions with other people within and outside of your village. เราจะเริ่มโดยการถามคำถามท่านเกี่ยวกับปฏิสัมพันธ์ของท่านกับชาวบ้านคนอื่นๆ ทั้งในและนอกหมู่บ้าน ເຮົາຈະເລີ່ມໂດຍການຖາມຄຳຖາມທ່ານກ່ຽວກັບການພົວພັນຂອງທ່ານກັບຊາວບ້ານຄົນອື່ນໆ ທັງໃນແລະພາຍນອກຂອງໝູ່ບ້ານ

5. What are you thinking about when you hear the following question? ท่านมีความคิดอะไรบ้างเมื่อท่านได้ยินคำถามต่อไปนี้ ທ່ານມີຄວາມຄິດຫຍັງແດ່ເມື່ອທ່ານໄດ້ຍິນຄຳຖາມຕໍ່ໄປນີ້

OxTREC reference: 528-17 **ANTIBIOTICS AND ACTIVITY SPACES**

Where do you spend most of your time interacting with other people from your village? ท่านใช้เวลาส่วนมากในการพบปะพูดคุยกับชาวบ้านในหมู่บ้านของท่านในสถานที่ใด ທ່ານໃຊ້ເວລາຫຼາຍທີ່ສຸດໃນການພົບປະໂອ້ລົມກັບຊາວບ້ານໃນໝູ່ບ້ານຂອງທ່ານໃນບ່ອນໃດແດ່<br><br>[*mark all that apply or select 99 if respondent indicates no noteworthy interaction*] [*ตอบได้มากกว่า 1 ตัวเลือก หรือเลือก 99 หากไม่มีปฏิสัมพันธ์ที่เด่นชัดปรากฏ*] [*ຕອບໄດ້ຫຼາຍກວ່າ 1 ຕົວເລືອກ ຫຼືເລືອກ 99 ຖ້າບໍ່ມີການພົວພັນທີ່ຊັດເຈນ*]	Field (specify) ไร่นา (โปรดระบุ) ໄຮ່ນາ (ກະລຸນາບອກ) ___ ....1<br>Temple (specify) วัด (โปรดระบุ) ວັດ (ກະລຸນາບອກ) ___ ....2<br>Local store (specify) ร้านค้า (โปรดระบุ) ຮ້ານຄ້າ (ກະລຸນາບອກ) ___ ....3<br>Market (specify) ตลาด (โปรดระบุ) ຕະຫຼາດ (ກະລຸນາບອກ) ___..4<br>Children's schools (specify) โรงเรียนของบุตรหลาน (โปรดระบุ) ໂຮງຮຽນຂອງລູກ (ກະລຸນາບອກ) __ ....5<br>Home (specify) ที่บ้าน (โปรดระบุ) ເຮືອນ (ກະລຸນາບອກ) ____ ....6<br>Workplace (specify) ที่ทำงาน (โปรดระบุ) ບ່ອນເຮັດວຽກ (ກະລຸນາບອກ) ___.........................7<br>Other site (specify) อื่นๆ (โปรดระบุ) ອື່ນໆ (ກະລຸນາບອກ) ___.......................................8<br>I don't interact notably with others ไม่มีปฏิสัมพันธ์ที่เด่นชัดกับชาวบ้านคนอื่นๆ ບໍ່ມີການພົວພັນທີ່ຊັດເຈນກັບຊາວບ້ານຄົນອື່ນໆ...99

a. How do you understand "interacting?" สำหรับท่าน ท่านคิดว่าคำว่า "พบปะพูดคุย" มีความหมายว่าอย่างไร ສຳລັບທ່ານ ທ່ານຄິດວ່າ "ການພົບປະໂອ້ລົມ" ມີຄວາໝາຍວ່າແນວໃດ
b. What does "most of your time" mean to you? Which time frame did you have in mind? คำว่า "เวลาส่วนมาก" สำหรับท่านมีความหมายว่าอย่างไร ในขณะที่ท่านตอบคำถามในใจของท่านนึกถึงช่วงเวลาใด ຄຳວ່າ "ເວລາຫຼາຍທີ່ສຸດ" ສຳລັບທ່ານມີຄວາມໝາຍວ່າແບບໃດ ໃນຂະນະທີ່ທ່ານຕອບຄຳຖາມໃນໃຈຂອງທ່ານນຶກເຖິງຊ່ວງເວລາໃດ
c. What are the possible places where people spend time together? มีที่ใดบ้างที่ชาวบ้านสามารถใช้เวลาร่วมกันได้ ມີບ່ອນໃດແດ່ທີ່ຊາວບ້ານສາມາດໃຊ້ເວລາຮ່ວມກັນໄດ້
d. Please ask us the same question in your own words. กรุณาถามคำถามนี้กับเราโดยใช้คำพูดในแบบของท่านเอง ກະລຸນາຖາມຄຳຖາມນີ້ກັບເຮົາໂດຍໃຊ້ຄຳເວົ້າໃນແບບຂອງທ່ານເອງ
e. What kind of things do you normally talk about when you interact with other villagers? โดยปกติเมื่อท่านพบปะกับชาวบ้านคนอื่นๆ ท่านสนทนากันเรื่องอะไรบ้าง ໂດຍປົກກະຕິທ່ານພົບປະກັບຊາວບ້ານຄົນອື່ນໆ ທ່ານລົມເລື່ອງຫຍັງແດ່

Haenssgen MJ, *et al. BMJ Open* 2019; 9:e028224. doi: 10.1136/bmjopen-2018-028224

OxTREC reference: 528-17 **ANTIBIOTICS AND ACTIVITY SPACES**

6. I will now ask you a set of questions, regarding the people you interact with. ชุดคำถามต่อไปจะถามถึงบุคคลที่ท่านพบปะพูดคุยด้วย ຊຸດຄຳຖາມຕໍ່ໄປຈະຖາມເຖິງບຸກຄົນທີ່ທ່ານພົບປະໂອ້ລົມນຳ

With whom do you interact on a regular basis? โดยปกติในชีวิตประจำวันท่านพบปะพูดคุยกับใครบ้าง ໂດຍປົກກະຕິໃນຊີວິດປະຈຳວັນທ່ານພົບປະໂອ້ລົມກັບໃຜແດ່									
	a) What is the name of the person? ชื่อ ຊື່	b) How is this person related to you? ความสัมพันธ์กับท่าน ຄວາມສຳພັນກັບທ່ານ<br>[*give examples if respondent is unsure about answer categories*] [*หากผู้ให้สัมภาษณ์ไม่มั่นใจในหมวดคำตอบ ให้ยกตัวอย่างคำตอบได้*] [*ຖ້າຜູ້ໃຫ້ສຳພາດບໍ່ໝັ້ນໃຈໃນໝວດຄຳຕອບ ໃຫ້ຍົກຕົວຢ່າງຄຳຕອບໄດ້*]	c) What is the sex of this person? เพศ ເພດ	d) Where does this person live? ที่อยู่ ທີ່ຢູ່	e) What is the name of the household head of this person? ใครคือหัวหน้าครัวเรือนของบุคคลนี้ ໃຜແມ່ນຫົວໜ້າຄົວເຮືອນຂອງບຸກຄົນນີ້	f) How often do you interact with this person? ท่านพบปะพูดคุยกับบุคคลนี้บ่อยเท่าใด ທ່ານພົບປະໂອ້ລົມກັບບຸກຄົນນີ້ບ່ອຍປານໃດ	g) How do you interact with this person? ท่านพบปะพูดคุยกับบุคคลนี้อย่างไร ທ່ານພົບປະໂອ້ລົມກັບບຸກຄົນນີ້ແນວໃດ<br>[*Mark all that apply*] [*ตอบได้มากกว่า 1 ตัวเลือก*] [*ຕອບໄດ້ຫຼາຍກ່ວາ 1 ຕົວເລືອກ*]	h) How often do your conversations relate to health and well-being? ท่านมีการสนทนากับบุคคลนี้เกี่ยวกับเรื่องสุขภาพบ่อยเท่าใด ທ່ານໂອ້ລົມກັບບຸກຄົນນີ້ເລື່ອງສຸຂະພາບບ່ອຍປານໃດ<br>[*If respondent does not discuss health, then move on to next contact after indicating "never"*] [*หากผู้ให้ไม่สบายใจกับการพูดคุยเรื่องสุขภาพ ให้ผู้สัมภาษณ์ใส่รหัส **0** และข้ามไปที่บุคคลต่อไป*] [*ຖ້າຜູ້ໃຫ້ສຳພາດບໍ່ສະບາຍໃຈທີ່ຈະລົມເລື່ອງສຸຂະພາບໃຫ້ຜູ້ສຳພາດໃສ່ລະຫັດ 0 ແລະຂ້າມໄປຫາບຸກຄົນຕໍ່ໄປ*]	i) What health-related subjects do your conversations cover? หัวข้อการสนทนาเกี่ยวกับสุขภาพของท่านกับบุคคลนี้คือเรื่องอะไร ຫົວຂໍ້ສົນທະນາກ່ຽວກັບສຸຂະພາບຂອງທ່ານກັບບຸກຄົນນີ້ແມ່ນເລື່ອງຫຍັງ<br>[*Mark all that apply*] [*ตอบได้มากกว่า 1 ตัวเลือก*] [*ຕອບໄດ້ຫຼາຍກ່ວາ 1 ຕົວເລືອກ*]
Contact 1<br>คนที่ 1<br>ຄົນທີ່ 1	Name ชื่อ ຊື່<br>_____	Spouse คู่สมรส ຄູ່ແຕ່ງດອງ........ 1<br>Parent บิดามารดา ພໍ່ແມ່............ 2<br>Child บุตร ລູກ...................... 3<br>Sibling พี่น้อง ອ້າຍນ້ອງ .... 4<br>Other relative ญาติ ພີ່ນ້ອງ ...... 5<br>Neighbour เพื่อนบ้าน ເພື່ອນບ້ານ . 6<br>Friend (if not neighbour) เพื่อน (ที่ไม่ใช่เพื่อนบ้าน) ໝູ່ (ທີ່ບໍ່ແມ່ນເພື່ອນບ້ານ) .... 7<br>Other villager ชาวบ้านคนอื่นๆ ຊາວບ້ານຄົນອື່ນໆ................... 8<br>Other (specify) อื่นๆ (โปรดระบุ) ອື່ນໆ (ກະລຸນາບອກ) _ 9	Female..1 หญิง ຍິງ<br>Male .....2 ชาย ຊາຍ	In village ....1 (specify: _____) ในหมู่บ้าน (โปรดระบุ: __) ໃນໝູ່ບ້ານ(ກະລຸນາບອກ: __)<br>Outside village ...2 (specify: _____) นอกหมู่บ้าน (โปรดระบุ: __) ນອກໝູ່ບ້ານ(ກະລຸນາບອກ: __)	Name of household head ชื่อหัวหน้าครัวเรือน: ຊື່ຫົວໜ້າຄົວເຮືອນ<br>_______	Daily or more often ทุกวันหรือบ่อยกว่า ທຸກມື້ຫຼືໜ້ອຍກ່ວາ.........4<br>Weekly or few times/week ทุกสัปดาห์หรือมากกว่าหนึ่งครั้งต่อสัปดาห์ ທຸກອາທິດຫຼືຫຼາຍກ່ວາໜຶ່ງເທື່ອຕໍ່ອາທິດ...............................3<br>Monthly or few times/month ทุกเดือนหรือมากกว่าหนึ่งครั้งต่อเดือน ທຸກເດືອນຫຼືຫຼາຍກ່ວາໜຶ່ງເທື່ອຕໍ່າເດືອນ..............................2<br>Yearly or few times/year ทุกปีหรือมากกว่าหนึ่งครั้งต่อปี ທຸກປີຫຼືຫຼາຍກວ່າໜຶ່ງເທື່ອຕໍ່ປີ 1	Face-to-face ตัวต่อตัว ໂຕຕໍ່ໂຕ..........1<br>Voice call โทรศัพท์ ໂທລະສັບ ....2<br>Messenger ติดต่อสื่อสารโดยใช้อินเตอร์เน็ต ສື່ສານໂດຍໃຊ້ອິນເຕີເນັດ.......3<br>Other (specify) อื่นๆ (โปรดระบุ) ອື່ນໆ (ກະລຸນາບອກ)<br>______ 4	Always (100%) เป็นประจำ (100%) ເປັນປະຈຳ (100%) .. 5<br>Nearly always (<100%) เกือบเป็นประจำ (<100%) ເກືອບເປັນປະຈຳ (<100%) .... 4<br>Often (<90%) บ่อยครั้ง (<90%) ບ່ອຍ (<90%) .... 3<br>Sometimes (<50%) บางครั้ง (<50%) ບາງຄັ້ງ (<50%)........ 2<br>Hardly ever (<10%) แทบไม่เคย (<10%) ເກືອບບໍ່ເຄີຍ (<10%). 1<br>Never (0%) ไม่เคยเลย (0%).... ບໍ່ເຄີຍເລີຍ......................... 0<br>➔ [***go to next contact***] [*ข้ามไปบุคคลต่อไป*] [*ຂ້າມໄປບຸກຄົນຕໍ່ໄປ*]	Talk about general well-being ไถ่ถามสารทุกข์สุกดิบทั่วไป ຖາມກ່ຽວກັບການເປັນຢູ່ທົ່ວໄປ ....1<br>Update on health knowledge/news อัพเดทเรื่องความรู้หรือข่าวสารสุขภาพใหม่ๆ ອັບເດດເລື່ອງຄວາມຮູ້ຫຼືຂ່າວສານສຸຂະພາບໃໝ່ໆ................2<br>Update on personal health status .... อัพเดทเรื่องสุขภาพส่วนตัว ອັບເດດເລື່ອງສຸຂະພາບສ່ວນຕົວ ....3<br>Advice on diagnosis ให้คำแนะนำเกี่ยวกับการวินิจฉัย ໃຫ້ຄຳແນະນຳກ່ຽວກັບການວິເຄາະພະຍາດ.......................4<br>Advice on doctors ให้คำแนะนำเกี่ยวกับแพทย์ ໃຫ້ຄຳແນະນຳກ່ຽວກັບໝໍ...5<br>Advice on treatment/medicine ให้คำแนะนำเกี่ยวกับวิธีการรักษา/ยา ໃຫ້ແນະນຳກ່ຽວກັບວິທີຮັກສາ/ຢາ 6

20 June 2017. Version No: 2.0 Page **4** of **17**

OxTREC reference: 528-17

ANTIBIOTICS AND ACTIVITY SPACES

						Less often or never ไม่ค่อยบ่อยหรือไม่เคยเลย ບໍ່ເຄີຍເລີຍ 0			Emotional support/reassure ให้กำลังใจ ให้ความมั่นใจ ໃຫ້ກຳລັງໃຈ ໃຫ້ຄວາມໝັ້ນໃຈ ....7<br>Provide help during illness ให้ความช่วยเหลือระหว่างการป่วย ໃຫ້ຄວາມຊ່ວຍເຫຼືອຕອນບໍ່ສະບາຍ ....8<br>Other (specify) อื่นๆ (โปรดระบุ) ອື່ນໆ (ກະລຸນາບອກ)___ .9
Contact 2<br>คนที่ 2<br>ຄົນທີ່ 2	Name ชื่อ ຊື່	1 2 3 4 5 6 7 8 9	1 2	1 2	Name ชื่อ ຊື່	0 1 2 3 4	1 2 3 4	0 1 2 3 4 5	1 2 3 4 5 6 7 8 9
[*continue until Contact x – maximum 10 contacts*]<br>[บันทึกบุคคลที่มีความเกี่ยวข้องระหว่างการเจ็บป่วยทุกคน ไม่เกิน 10 คน]<br>[*ບັນທຶກບຸກຄົນທີ່ມີຄວາມກ່ຽວຂ້ອງລະຫວ່າງອາການເຈັບປ່ວຍທຸກຄົນ ບໍ່ຫຼາຍກ່ວາ 10 ຄົນ*]	Name ชื่อ ຊື່	1 2 3 4 5 6 7 8 9	1 2	1 2	Name ชื่อ ຊື່	0 1 2 3 4	1 2 3 4	0 1 2 3 4 5	1 2 3 4 5 6 7 8 9

20 June 2017. Version No: 2.0

a. How did you go about answering these questions? ท่านมีวิธีการตอบคำถามเหล่านี้อย่างไร ທ່ານມີວິທີການຕອບຄຳຖາມເຫຼົ່ານີ້ ແນວໃດ
b. What did you imagine while answering the question, e.g. the “interaction setting” that you had in mind? ในขณะที่ท่านตอบคำถามเหล่านี้ท่านมีภาพอะไรอยู่ในใจ เช่น ลักษณะของสถานที่ ฯลฯ ໃນຂະນະທີ່ທ່ານຕອບຄຳຖາມເຫຼົ່ານີ້ທ່ານມີພາບຫຍັງຢູ່ໃນໃຈ ເຊັ່ນ ລັກຊະນະຂອງສະຖານທີ່ ແລະອື່ນໆ
c. What time period were you thinking of? จากคำตอบที่ท่านให้เราเกี่ยวกับบุคคลที่ท่านมีปฏิสัมพันธ์ด้วย ท่านนึกถึงช่วงเวลาใด ຈາກຄຳຕອບທີ່ທ່ານໃຫ້ເຮົາກ່ຽວກັບບຸກຄົນທີ່ທ່ານມີປະຕິສຳພັນນຳ ທ່ານນຶກເຖິງຊ່ວງເວລາໃດ
d. How did you come up with the order of people? Why did you think of these people first rather than others? ท่านมีการเรียงลำดับบุคคลเหล่านี้อย่างไร เพราะเหตุใดท่านจึงบอกชื่อบุคคลเหล่านี้กับเราเป็นลำดับแรกๆ หากเปรียบเทียบกับบุคคลอื่น ທ່ານມີການລຽງລຳດັບບຸກຄົນເຫຼົ່ານີ້ແບບໃດ ຍ້ອນຫຍັງທ່ານຈຶ່ງບອກຊື່ບຸກຄົນເຫຼົ່ານີ້ກັບກ່ອນຄົນອື່ນ
e. What is the best way we can find these people from your interaction circle? How would you describe where they live? หากเราต้องการพูดคุยกับบุคคลที่ท่านกล่าวถึงเหล่านี้ เราสามารถพบพวกเขาได้อย่างไร ท่านจะสามารถอธิบายที่อยู่ของพวกเขาได้อย่างไร ຖ້າເຮົາຕ້ອງການລົມກັບບຸກຄົນທີ່ທ່ານກ່າວເຖິງເຫຼົ່ານີ້ ເຮົາສາມາດພົບພົບພວກເຂົາໄດ້ແນວໃດ ທ່ານສາມາດອະທິບາຍທີ່ຢູ່ຂອງພວກເຂົາໄດ້ແນວໃດ

Part IV – Health-Related Survey Questions ส่วนที่ 4 – คำถามเกี่ยวกับสุขภาพ ສ່ວນທີ່ 4 - ຄຳຖາມກ່ຽວກັບສຸຂະພາບ

Thank you for this. Now we come to a part where I will ask you some questions about health and health providers around here. ขอบคุณครับ/ค่ะ คำถามช่วงต่อไปจะเป็นเรื่องสุขภาพและสถานบริการสุขภาพในพื้นที่นี้ ຂອບໃຈ ຄຳຖາມຕໍ່ໄປນີ້ຈະເປັນຄຳຖາມກ່ຽວກັບສຸຂະພາບ ແລະສະຖານບໍລິການສຸຂະພາບໃນພື້ນທີ່ນີ້

7. For the following question, can you please “think out loud” about your answers? เราขอให้ท่าน “คิดออกเสียง” ในระหว่างที่ท่านตอบคำถามต่อไปนี้ ເຮົາຂໍໃຫ້ທ່ານ “ຄິດອອກສຽງ” ໃນລະຫ່ວາງການຕອບຄຳຖາມຕໍ່ໄປນີ້

I would now like to ask you about the sources of health advice and medicine or other treatment that are available to you. Please think about all the places where you can go to get advice, treatment, or drugs if you (or your children) are sick. คำถามต่อไปเป็นคำถามเกี่ยวกับสถานที่หรือบุคคลที่ท่านสามารถรับคำปรึกษา รับยา หรือ รับการรักษาอื่นๆ ได้ กรุณานึกถึงสถานที่ทุกแห่งหรือคนทุกคนที่ท่านสามารถรับคำปรึกษา รับการรักษา หรือรับยา หากท่านหรือเด็กในปกครองของท่านป่วย ຄຳຖາມຕໍ່ໄປເປັນຄຳຖາມກ່ຽວກັບສະຖານທີ່ຫຼືບຸກຄົນທີ່ທ່ານສາມາດຮັບຄຳປຶກສາ ຮັບຢາ ຫຼືຮັບການຮັກສາອື່ນໆ ໄດ້ ກະລຸນານຶກເຖິງສະຖານທີ່ທຸກແຫ່ງຫຼືບຸກຄົນທຸກຄົນທີ່ທ່ານສາມາດຮັບຄຳປຶກສາ ຮັບການຮັກສາ ຫຼືຮັບຢາ ຖ້າຫາກທ່ານຫຼືເດັກທີ່ທ່ານດູແລບໍ່ສະບາຍ<br><br>Which of the following options do you consider for medical treatment, advice, or to get medicine? ท่านเคยได้รับคำปรึกษา รับยา หรือ รับ	Drug dispensary, other local store selling medicine ร้านขายของชำหรือร้านค้าทั่วไปที่มีการจำหน่ายยา ຮ້ານທົ່ວໄປທີ່ມີການຂາຍຢາ	Medical treatment เพื่อการรักษา ສຳລັບການປິ່ນປົວ .... 1<br>Medical advice เพื่อคำแนะนำในการรักษา ສຳລັບຄຳແນະນຳໃນການປິ່ນປົວ .... 2<br>Access to medicine เพื่อซื้อ/รับยา ສຳລັບຊື້/ຮັບຢາ .... 3<br>Don’t consider this provider ไม่มีการใช้บริการประเภทนี้ ບໍ່ມີການໃຊ້ບໍລິການປະເພດນີ້..0<br>Don’t know such a provider ไม่รู้จักผู้ให้บริการประเภทนี้ ບໍ່ຮູ້ຈັກຜູ້ໃຫ້ບໍລິການປະເພດນີ້. 99
	Traditional healer หมอชาวบ้าน ໝໍຊາວບ້ານ	Medical treatment เพื่อการรักษา ສຳລັບການປິ່ນປົວ .... 1<br>Medical advice เพื่อคำแนะนำในการรักษา ສຳລັບຄຳແນະນຳໃນການປິ່ນປົວ .... 2<br>Access to medicine เพื่อซื้อ/รับยา ສຳລັບຊື້/ຮັບຢາ .... 3<br>Don’t consider this provider ไม่มีการใช้บริการประเภทนี้ ບໍ່ມີການໃຊ້ບໍລິການປະເພດນີ້..0<br>Don’t know such a provider ไม่รู้จักผู้ให้บริการประเภทนี้ ບໍ່ຮູ້ຈັກຜູ້ໃຫ້ບໍລິການປະເພດນີ້. 99
	Pharmacist เภสัชกร ຜູ້ຂາຍຢາ	Medical treatment เพื่อการรักษา ສຳລັບການປິ່ນປົວ .... 1<br>Medical advice เพื่อคำแนะนำในการรักษา ສຳລັບຄຳແນະນຳໃນການປິ່ນປົວ .... 2<br>Access to medicine เพื่อซื้อ/รับยา ສຳລັບຊື້/ຮັບຢາ .... 3<br>Don’t consider this provider ไม่มีการใช้บริการประเภทนี้ ບໍ່ມີການໃຊ້ບໍລິການປະເພດນີ້..0<br>Don’t know such a provider ไม่รู้จักผู้ให้บริการประเภทนี้ ບໍ່ຮູ້ຈັກຜູ້ໃຫ້ບໍລິການປະເພດນີ້. 99
	Private clinic คลินิกเอกชน ຄລີນິກເອກະຊົນ	Medical treatment เพื่อการรักษา ສຳລັບການປິ່ນປົວ .... 1<br>Medical advice เพื่อคำแนะนำในการรักษา ສຳລັບຄຳແນະນຳໃນການປິ່ນປົວ .... 2<br>Access to medicine เพื่อซื้อ/รับยา ສຳລັບຊື້/ຮັບຢາ .... 3<br>Don’t consider this provider ไม่มีการใช้บริการประเภทนี้ ບໍ່ມີການໃຊ້ບໍລິການປະເພດນີ້..0<br>Don’t know such a provider ไม่รู้จักผู้ให้บริการประเภทนี้ ບໍ່ຮູ້ຈັກຜູ້ໃຫ້ບໍລິການປະເພດນີ້. 99

Haenssgen MJ, et al. BMJ Open 2019; 9:e028224. doi: 10.1136/bmjopen-2018-028224

การรักษา จากใครบ้าง ທ່ານເຄີຍໄດ້ຮັບຄຳປຶກສາ ຮັບຢາ ຫຼືຮັບການຮັກສາອື່ນຈາກໃຜແດ່<br><br>[*Mark all that apply*] [*ตอบได้มากกว่า 1 ตัวเลือก*] [*ຕອບໄດ້ຫຼາຍກ່ວາ 1 ຕົວເລືອກ*]	Private hospital โรงพยาบาลเอกชน ໂຮງໝໍເອກະຊົນ	Medical treatment เพื่อการรักษา ສຳລັບການປິ່ນປົວ .... 1<br>Medical advice เพื่อคำแนะนำในการรักษา ສຳລັບຄຳແນະນຳໃນການປິ່ນປົວ .... 2<br>Access to medicine เพื่อซื้อ/รับยา ສຳລັບຊື້/ຮັບຢາ .... 3<br>Don't consider this provider ไม่มีการใช้บริการประเภทนี้ ບໍ່ມີການໃຊ້ບໍລິການປະເພດນີ້..0<br>Don't know such a provider ไม่รู้จักผู้ให้บริการประเภทนี้ ບໍ່ຮູ້ຈັກຜູ້ໃຫ້ບໍລິການປະເພດນີ້. 99
	Primary care unit สถานีอนามัยของรัฐ ສະຖານີອານາໄມຂອງລັດ	Medical treatment เพื่อการรักษา ສຳລັບການປິ່ນປົວ .... 1<br>Medical advice เพื่อคำแนะนำในการรักษา ສຳລັບຄຳແນະນຳໃນການປິ່ນປົວ .... 2<br>Access to medicine เพื่อซื้อ/รับยา ສຳລັບຊື້/ຮັບຢາ .... 3<br>Don't consider this provider ไม่มีการใช้บริการประเภทนี้ ບໍ່ມີການໃຊ້ບໍລິການປະເພດນີ້..0<br>Don't know such a provider ไม่รู้จักผู้ให้บริการประเภทนี้ ບໍ່ຮູ້ຈັກຜູ້ໃຫ້ບໍລິການປະເພດນີ້. 99
	Public hospital โรงพยาบาลรัฐ ໂຮງໝໍລັດ	Medical treatment เพื่อการรักษา ສຳລັບການປິ່ນປົວ .... 1<br>Medical advice เพื่อคำแนะนำในการรักษา ສຳລັບຄຳແນະນຳໃນການປິ່ນປົວ .... 2<br>Access to medicine เพื่อซื้อ/รับยา ສຳລັບຊື້/ຮັບຢາ .... 3<br>Don't consider this provider ไม่มีการใช้บริการประเภทนี้ ບໍ່ມີການໃຊ້ບໍລິການປະເພດນີ້..0<br>Don't know such a provider ไม่รู้จักผู้ให้บริการประเภทนี้ ບໍ່ຮູ້ຈັກຜູ້ໃຫ້ບໍລິການປະເພດນີ້. 99
	Other providers or Internet? Specify: ผู้ให้บริการรูปแบบอื่นๆ หรือ อินเตอร์เน็ต (โปรดระบุ) ຜູ້ບໍລິການໃນຮູບແບບອື່ນໆຫຼື ອິນເຕີເນັດ (ກະລຸນາບອກ)<br>__________	Medical treatment เพื่อการรักษา ສຳລັບການປິ່ນປົວ .... 1<br>Medical advice เพื่อคำแนะนำในการรักษา ສຳລັບຄຳແນະນຳໃນການປິ່ນປົວ .... 2<br>Access to medicine เพื่อซื้อ/รับยา ສຳລັບຊື້/ຮັບຢາ .... 3<br>Don't consider this provider ไม่มีการใช้บริการประเภทนี้ ບໍ່ມີການໃຊ້ບໍລິການປະເພດນີ້..0<br>Don't know such a provider ไม่รู้จักผู้ให้บริการประเภทนี้ ບໍ່ຮູ້ຈັກຜູ້ໃຫ້ບໍລິການປະເພດນີ້. 99

a. Do you choose any of these providers for specific symptoms or illnesses? ท่านมีการเลือกสถานบริการสุขภาพแห่งใดแห่งหนึ่งสำหรับรักษาโรคใดโดยเฉพาะหรือไม่ ທ່ານມີການເລືອກສະຖານສຸຂະພາບທີ່ໃດທີ່ໜຶ່ງສຳລັບກວດພະຍາດໃດໂດຍສະເພາະຫຼືບໍ່
b. Do you think these questions are too hard? ท่านรู้สึกว่าคำถามเหล่านี้ยากเกินไปหรือไม่ ທ່ານຮູ້ສຶກວ່າຄຳຖາມເຫຼົ່ານີ້ຍາກເກີນໄປຫຼືບໍ່
c. How big of an area do you think of when you answered these questions? E.g. within the village, the province, the surrounding provinces ในขณะที่ท่านตอบคำถามเหล่านี้ ท่านนึกถึงสถานบริการสุขภาพภายในบริเวณใด เช่น ภายในหมู่บ้าน ภายในจังหวัด หรือรวมจังหวัดรอบนอกด้วย ຕອນທີ່ທ່ານຕອບຄຳຖາມເຫຼົ່ານີ້ ທ່ານນຶກເຖິງສະຖານທີ່ບໍລິການສຸຂະພາບພາຍໃນບໍລິເວນໃດ ເຊັ່ນ ພາຍໃນໝູ່ບ້ານ ພາຍໃນແຂວງ ຫຼືແຂວງຮອບນອກ
d. Are there other forms of health provider that you could use but we have not talked about? มีสถานบริการสุขภาพลักษณะอื่นที่ท่านสามารถใช้บริการได้แต่เรายังไม่ได้พูดถึงอีกหรือไม่ ມີສະຖານທີ່ບໍລິການສຸຂະພາບລັກຊະນະອື່ນທີ່ທ່ານສາມາດໃຊ້ບໍລິການໄດ້ແຕ່ເຮົາຍັງບໍ່ໄດ້ເວົ້າເຖິງອີກຫຼືບໍ່

8. I will now ask you another series of questions regarding a recent illness. ต่อไปเราจะถามคำถามเกี่ยวกับอาการป่วยครั้งล่าสุดของท่าน ຕໍ່ໄປນີ້ເຮົາຈະຖາມຄຳຖາມກ່ຽວກັບການເຈັບເປັນຄັ້ງລ່າສຸດຂອງທ່ານ

Did you have an acute illness (not a chronic, long-term condition that comes again and again) or an accident in the last two months? ในช่วงสองเดือนที่ผ่านมา ท่านมีอาการป่วย (ไม่รวมโรคเรื้อรังหรือโรคที่เป็นๆ หายๆ) หรือประสบอุบัติเหตุ หรือไม่ ໃນຊ່ວງສອງເດືອນທີ່ຜ່ານມາ ທ່ານມີອາການເຈັບເປັນ (ບໍ່ລວມພະຍາດຊຳເຮື້ອຫຼືພະຍາດທີ່ເປັນຕໍ່ເນື່ອງ) ຫຼືອຸປະຕິເຫດຫຼືບໍ່?	No ไม่ใช่ ບໍ່ແມ່ນ .... 2<br>Yes ใช่ ແມ່ນ............ 1
[*if yes*:] [*ถ้าใช่:*] [*ຖ້າແມ່ນ:*]	Description of condition: คำอธิบายอาการ ຄຳອະທິບາຍອາການ<br>__________

Haenssgen MJ, *et al. BMJ Open* 2019; 9:e028224. doi: 10.1136/bmjopen-2018-028224

Can you please describe the symptoms or problem in your own words? กรุณาอธิบายอาการหรือปัญหาในแบบที่ท่านเข้าใจ ກະລຸນາອະທິບາຍອາການຫຼືບັນຫາໃນແບບທີ່ທ່ານເຂົ້າໃຈ	__________________________________

Haenssgen MJ, *et al. BMJ Open* 2019; 9:e028224. doi: 10.1136/bmjopen-2018-028224

OxTREC reference: 528-17 **ANTIBIOTICS AND ACTIVITY SPACES**

Can you please describe the diagnosis of the illness if you received any and where you received it?<br>[*note: the diagnosis might be given by any medical provider including untrained and informal. Record all diagnoses if more than one.*] หากท่านได้รับการวินิจฉัยอาการ กรุณาอธิบายการวินิจฉัยนั้นและระบุแหล่งที่มา [หมายเหตุ: ผู้ให้สัมภาษณ์อาจได้รับการวินิจฉัยจากผู้ให้บริการทางสุขภาพลักษณะใดก็ได้ ซึ่งรวมถึงผู้ให้บริการที่ไม่รับการศึกษาด้านการแพทย์และผู้ให้บริการนอกระบบ กรอกข้อมูลการวินิจฉัยทั้งหมด หากผู้ให้สัมภาษณ์ได้รับการวินิจฉัยมากกว่า 1 ครั้ง] ຖ້າຫາກທ່ານໄດ້ຮັບການວິເຄາະພະຍາດ ກະລຸນາອະທິບາຍການວິເຄາະພະຍາດເທື່ອນັ້ນແລະລະບຸແຫຼ່ງຂໍ້ມູນ [ຫມາຍເຫດ: ຜູ້ໃຫ້ສໍາພາດອາດໄດ້ຮັບການວິເຄາະພະຍາດຈາກຜູ້ໃຫ້ບໍລິການສຸຂະພາບປະເພດໃດກໍ່ໄດ້ ເຊິ່ງຮວມເຖິງຜູ້ໃຫ້ບໍລິການສຸຂະພາບທີ່ບໍ່ມີການສຶກສາທາງການແພດແລະຜູ້ໃຫ້ບໍລິການນອກລະບົບ ຕື່ມໃສ່ຂໍ້ມູນການວິເຄາະພະຍາດທັງຫມົດ ຖ້າຜູ້ໃຫ້ສໍາພາດໄດ້ຮັບການວິເຄາະພະຍາດຫຼາຍກ່ວາ 1 ຄັ້ງ]<br>[*Response codes* รหัสคำตอบ ລະຫັດຄໍາຕອບ]<br>Drug dispensary, other local store selling medicine<br>ร้านขายของชำหรือร้านค้าทั่วไปที่มีการจำหน่ายยา ຮ້ານທົ່ວໄປທີ່ມີການຂາຍຢາ .... 1<br>Traditional healer หมอชาวบ้าน ໝໍຊາວບ້ານ .... 2<br>Pharmacist เภสัชกร ຜູ້ຂາຍຢາ .... 3<br>Private clinic คลินิกเอกชน ຄລີນິກເອກະຊົນ .... 4<br>Private hospital โรงพยาบาลเอกชน ໂຮງໝໍເອກະຊົນ .... 5<br>Primary care unit สถานีอนามัยของรัฐ ສະຖານີອານາໄມຂອງລັດ .... 6<br>Public hospital โรงพยาบาลรัฐ ໂຮງໝໍລັດ .... 7<br>Other providers or Internet? Specify: ผู้ให้บริการรูปแบบอื่นๆ หรืออินเตอร์เน็ต (โปรดระบุ) ຜູ້ບໍລິການໃນຮູບແບບອື່ນໆຫຼືອິນເຕີເນັດ (ກະລຸນາບອກ) ________ 8	Diagnosis 1 การวินิจฉัยโรค 1 ການວິເຄາະພະຍາດ 1:<br>________________________________<br>Medical provider 1 ผู้ให้บริการทางสุขภาพ 1 ຜູ້ໃຫ້ບໍລິການສຸຂະພາບ 1:<br>1 2 3 4 5 6 7 8<br>Diagnosis 2 การวินิจฉัยโรค 2 ການວິເຄາະພະຍາດ 2:<br>________________________________<br>Medical provider 2 ผู้ให้บริการทางสุขภาพ 2 ຜູ້ໃຫ້ບໍລິການສຸຂະພາບ 2:<br>1 2 3 4 5 6 7 8<br>Diagnosis 3 การวินิจฉัยโรค 3 ການວິເຄາະພະຍາດ 3:<br>________________________________<br>Medical provider 3 ผู้ให้บริการทางสุขภาพ 3 ຜູ້ໃຫ້ບໍລິການສຸຂະພາບ 3:<br>1 2 3 4 5 6 7 8
When did you experience the accident/discomfort (for the first time) ท่านเริ่มมีอาการป่วยหรือประสบอุบัติเหตุตั้งแต่เมื่อใด ທ່ານເລີ່ມມີອາການເຈັບເປັນຫຼືຖືກອຸບັດຕິເຫດຕັ້ງແຕ່ເມື່ອໃດ?	Onset: ___ days / ______ weeks / _____ months ago<br>เริ่มอาการป่วย/อุบัติเหตุ: _______ วัน / _______ สัปดาห์ / ______ เดือนที่แล้ว<br>ເລີ່ມມີອາການບໍ່ສະບາຍຫຼືອຸບັດຕິເຫດ: ___ ມື້ / ___ ອາທິດ / ___ ເດືອນກ່ອນ
Would you describe the illness/accident as “mild,” “moderate,” or “severe”? ท่านคิดว่าการป่วยหรืออุบัติเหตุครั้งนั้นอยู่ในขั้นน้อย ปานกลาง หรือหนัก ທ່ານຄິດວ່າອາການເຈັບເປັນຫຼືອຸບັດຕະເຫດຄັ້ງນັ້ນຢູ່ຂັ້ນນ້ອຍ, ປານກາງ, ຫຼືໜັກ	Mild น้อย ນ້ອຍ.................................1<br>Moderate ปานกลาง ປານກາງ ....2<br>Severe หนัก ໜັກ.............................3

a. Did you feel comfortable talking about your health? ท่านรู้สึกสบายใจ (หรืออึดอัดใจ) ในการพูดคุยเรื่องสุขภาพหรือไม่ ທ່ານຮູ້ສຶກສະບາຍໃຈ (ຫຼືອຶດອັດໃຈ) ໃນການລົມເລື່ອງສຸຂະພາບຫຼືບໍ່

b. Do you think these questions are too hard? ท่านรู้สึกว่าคำถามเหล่านี้ยากเกินไปหรือไม่ ທ່ານຮູ້ສຶກວ່າຄໍາຖາມເຫຼົ່ານີ້ຍາກເກີນໄປຫຼືບໍ່

c. How do you rank mild, moderate, and severe? What do those words mean to you? ท่านใช้อะไรเป็นเครื่องบ่งบอกว่าอาการป่วยของท่านน้อย ปานกลาง หรือหนัก, ในความคิดของท่าน น้อย ปานกลาง และหนัก หมายความว่าอย่างไร ທ່ານໃຊ້ຫຍັງເປັນເຄື່ອງບົ່ງບອກອາການເຈັບເປັນຂອງທ່ານ ນ້ອຍ ປານກາງ ຫຼືໜັກ, ໃນຄວາມຄິດຂອງທ່ານ ໜ້ອຍ ປານກາງແລະໜັກ ໝາຍຄວາມວ່າແນວໃດ

9. The next set of questions still relates to the same illness. คำถามชุดต่อไปยังคงเกี่ยวกับอาการป่วยครั้งเดียวกับคำถามก่อนหน้า ຄໍາຖາມຊຸດຕໍ່ໄປຍັງກ່ຽວກັບອາການບໍ່ສະບາຍເຊັ່ນດຽວກັບຄໍາຖາມກ່ອນໜ້ານີ້

Haenssgen MJ, *et al. BMJ Open* 2019; 9:e028224. doi: 10.1136/bmjopen-2018-028224

OxTREC reference: 528-17 **ANTIBIOTICS AND ACTIVITY SPACES**

Can you please explain the stages of the treatment? I will ask you step-by-step what you did, starting from the moment you first detected the condition. กรุณาอธิบายขั้นตอนการรักษาทุกขั้นตอนระหว่างการป่วยครั้งนี้ เราขอให้ท่านเล่าให้เราฟังทีละขั้นตอนตั้งแต่ท่านเริ่มรู้สึกมีอาการป่วย ກະລຸນາອະທິບາຍຂັ້ນຕອນການປິ່ນປົວທຸກຂັ້ນຕອນລະຫ່ວາງການເຈັບເປັນຄັ້ງນີ້ ເຮົາຂໍໃຫ້ທ່ານເລົ່າໃຫ້ເຮົາຟັງເທື່ອລະຂັ້ນຕອນຕັ້ງແຕ່ຕອນທີ່ທ່ານເລີ່ມມີອາການບໍ່ສະບາຍ

[*ask respondent what he or she did first, then code answer and continue. After each column, ask: "What did you do next?" Only one activity per step. If e.g. medical treatment and then home care, first step is medical treatment, second step is home care. Repeat until respondent was cured or today otherwise.*] [*ถามคำถามเกี่ยวกับขั้นตอนการรักษาขั้นแรกสุด จากนั้นกรอกรหัสคำตอบ แล้วจึงถามคำถามต่อไป หลังจากกรอกข้อมูลในแต่ละคอลัมน์ ให้ถาม "ท่านทำอะไรต่อไปหลังจากนั้น" กรอกข้อมูลของการรักษาแต่ละชนิดต่อหนึ่งขั้นตอนเท่านั้น ตัวอย่าง: "เข้าพบแพทย์แล้วรักษาอาการที่บ้าน" ให้กรอก ขั้นที่ 1 เข้าพบแพทย์ และขั้นที่ 2 รักษาอาการที่บ้าน บันทึกขั้นตอนทุกขั้นตอนเช่นนี้จนถึงขั้นที่หายจากอาการหรือจนถึงวันที่ทำการเก็บข้อมูล*] [*ຖາມຄຳຖາມກ່ຽວກັບຂັ້ນຕອນການປິ່ນປົວຂັ້ນທຳອິດ ຈາກນັ້ນຂຽນລະຫັດຄຳຕອບ ແລ້ວຈຶ່ງຖາມຄຳຖາມຕໍ່ໄປ ຫຼັງຈາກຂຽນຂໍ້ມູນໃນແຕ່ລະຫ້ອງ ໃຫ້ຖາມ "ທ່ານເຮັດຫຍັງຕໍ່ໄປຫຼັງຈາກນັ້ນ" ຂຽນຂໍ້ມູນຂອງການປິ່ນປົວແຕ່ລະຊະນິດຕໍ່ໜຶ່ງຂັ້ນຕອນເທົ່ານັ້ນ ຕົວຢ່າງ: "ເຂົ້າພົບໝໍແລ້ວປິ່ນປົວອາການຢູ່ເຮືອນ" ໃຫ້ຂຽນຂັ້ນທີ່ 1 ເຂົ້າພົບໝໍ ແລະຂັ້ນທີ່ 2 ປົວອາການຢູ່ເຮືອນ ບັນທຶກຂັ້ນຕອນທຸກຂັ້ນຕອນແບບນີ້ຈົນເຖິງຂັ້ນທີ່ດີຈາກອາການຫຼືຈົນຮອດມື້ເກັບຂໍ້ມູນ*]

	Step 1 (detection)<br>ขั้นที่ 1 (เมื่อเริ่มรู้สึกป่วย)<br>ຂັ້ນທີ່ 1 (ເມື່ອເລີ່ມບໍ່ສະບາຍ)	Step 2<br>ขั้นที่ 2<br>ຂັ້ນທີ່ 2	[*continue until Step x when discomfort was cured or until today*] [*บันทึกขั้นตอนทุกขั้นตอนในตารางนี้ตั้งแต่เริ่มป่วยจนถึงขั้นที่หายจากอาการหรือจนถึงวันที่ทำการเก็บข้อมูล*] [*ບັນທຶກຂັ້ນຕອນທຸກຂັ້ນຕອນໃນຕາຕະລາງນີ້ຕັ້ງແຕ່ເລີ່ມຈັບເປັນຈົນເຖິງຫາຍດີຫຼືຈົນຮອດມື້ເກັບຂໍ້ມູນ*]
a) What kind of help or treatment did you get at this stage? ท่านมีการรักษาในขั้นตอนนี้อย่างไร ທ່ານມີການປິ່ນປົວໃນຂັ້ນຕອນນີ້ແບບໃດ<br><br>[*if unsure, specify*] [*หากไม่มั่นใจ ให้กรอกรายละเอียดในหมวด "อื่นๆ"*] [*ຖ້າບໍ່ໝັ້ນໃຈໃຫ້ຂຽນລາຍລະອຽດໃນໝວດ "ອື່ນໆ"*]	Ignored /did nothing ละเลย/ไม่ทำอะไร ບໍ່ສົນໃຈ/ບໍ່ເຮັດຫຍັງເລີຍ. 1<br>Self-care (sleep, rest, medicine at home) รักษาตัวเอง (นอนหลับ, พักผ่อน, ใช้ยาที่มีที่บ้าน) ປົວຕົນເອງ (ນອນ, ພັກຜ່ອນ, ໃຊ້ຢາທີ່ມີຢູ່ເຮືອນ) .... 2<br>Care from family and friends (full-time) ได้รับการรักษาจากครอบครัวหรือเพื่อน (ตลอดเวลา) ໄດ້ຮັບການປິ່ນປົວຈາກຄອບຄົວຫຼືໝູ່ (ຕະຫຼອດເວລາ).......... 3<br>Treated/consulted at a pharmacist รับการรักษา/คำปรึกษาจากเภสัชกร ຮັບການປິ່ນປົວ/ຄຳປຶກສາຈາກຜູ້ຂາຍຢາ.......... 4<br>Treated/consulted at shop selling drugs รับการรักษา/คำปรึกษาจากร้านขายของชำหรือร้านค้าทั่วไปที่มีการจำหน่ายยา ຮັບການປິ່ນປົວ/ຄຳປຶກສາຈາກຮ້ານທົ່ວໄປທີ່ມີການຂາຍຢາ .... 5<br>Treated/consulted at priv. clinic/hospital รับการรักษา/คำปรึกษาจากคลินิก/โรงพยาบาลเอกชน ຮັບການປິ່ນປົວ/ຄຳປຶກສາຈາກຈາກຄລີນິກ/ໂຮງໝໍເອກະຊົນ .... 6<br>Treated/consulted at primary care unit รับการรักษา/คำปรึกษาจากสถานีอนามัยของรัฐ ຮັບການປິ່ນປົວ/ຄຳປຶກສາຈາກສະຖານີອານາໄມຂອງລັດ.......... 7<br>Treated/consulted at a gvt. Hospital รับการรักษา/คำปรึกษาจากโรงพยาบาลของรัฐ ຮັບການປິ່ນປົວ/ຄຳປຶກສາຈາກຈາກໂຮງໝໍຂອງລັດ ... 8<br>Other (specify) อื่นๆ (โปรดระบุ) ອື່ນໆ (ກະລຸນາບອກ) _ 9	1<br>2<br>3<br>4<br>5<br>6<br>7<br>8<br>9	1<br>2<br>3<br>4<br>5<br>6<br>7<br>8<br>9

b) Where did this activity take place? ขั้นตอนการรักษานี้เกิดขึ้นที่ไหน ຂັ້ນຕອນການປິ່ນປົວນີ້ເກີດຂຶ້ນຢູ່ໃສ	At home ที่บ้าน ຢູ່ເຮືອນ..1<br>Less than 10 min. from home น้อยกว่า 10 นาทีจากบ้าน ນ້ອຍກ່ວາ 10 ນາທີຈາກເຮືອນ..2<br>10 to 29 min. 10 ถึง 29 นาทีจากบ้าน 10 ຫາ 29 ນາທີຈາກເຮືອນ ....3<br>30 to 59 min. 30 ถึง 59 นาทีจากบ้าน 30 ຫາ 59 ນາທີຈາກເຮືອນ ....4<br>60 to 119 min. 60 ถึง 119 นาทีจากบ้าน 60 ຫາ 119 ນາທີຈາກເຮືອນ..5<br>2 hours or more from home 2 ชั่วโมงหรือมากกว่านั้น ສອງຊົ່ວໂມງຫຼືຫຼາຍກ່ວາ 6	1<br>2<br>3<br>4<br>5<br>6	1<br>2<br>3<br>4<br>5<br>6
c) How did you get to the place of the activity? ท่านเดินทางไปสถานที่ที่ท่านทำการรักษาในขั้นตอนนี้อย่างไร ທ່ານເດີນທາງໄປສະຖານທີ່ທີ່ ປິ່ນປົວໃນຂັ້ນຕອນນີ້ແນວໃດ<br><br>[*select "at home" according to prior responses* *เลือก "ที่บ้าน" ให้สอดคล้องกับคำตอบก่อนหน้าหากเข้าข่ายกรณีนั้น*] [*ເລືອກ "ຢູ່ເຮືອນ" ໃຫ້ສອດຄ່ອງ ກັບຄຳຕອບກ່ອນໜ້າຫາກກ່ຽວຂ້ອງກັນ*]	At home ที่บ้าน ຢູ່ເຮືອນ ....1<br>Walk เดินเท้า ຍ່າງ ....2<br>Own bicycle จักรยานของท่าน ລົດຖີບຂອງທ່ານ ....3<br>Own motorcycle / Three-wheeler รถจักรยานยนต์/รถสามล้อของท่าน ລົດຈັກ/ລົດສາມລໍ້ຂອງທ່ານ ....4<br>Own car / four-wheeler รถยนต์/ขับเคลื่อนสี่ล้อของท่าน ລົດຍົນ/ລົດໃຫຍ່ສີ່ລໍ້ຂອງທ່ານ..5<br>Taxi or other hired ride รถแท็กซี่/รถรับจ้าง ລົດແທັກຊີ/ລົດຮັບຈ້າງ ....6<br>Public transport ขนส่งสาธารณะ ລົດຂົນສົ່ງສາທາລະນະ.............7<br>Other (specify) อื่นๆ (โปรดระบุ) ອື່ນໆ (ກະລຸນາບອກ) _ 8	1<br>2<br>3<br>4<br>5<br>6<br>7<br>8	1<br>2<br>3<br>4<br>5<br>6<br>7<br>8
d) How long did this stage last? ท่านรักษาในขั้นตอนนี้เป็นระยะเวลาเท่าใด ທ່ານປິ່ນປົວໃນຂັ້ນຕອນນີ້ເປັນໄລຍະເວລາເທົ່າໃດ<br><br>[*let respondent choose category; if less than one day, code "1" day*] [*ให้ผู้ให้สัมภาษณ์เลือกหน่วยเวลาเอง หากน้อยกว่าหนึ่งวันให้กรอก "1" วัน*] [*ໃຫ້ຜູ້ໃຫ້ສຳພາດເລືອກໜ່ວຍເວລາເອງ ຖ້ານ້ອຍກວ່າໜຶ່ງມື້ໃຫ້ຂຽນ "1" ມື້*]	Duration ระยะเวลา ໄລຍະເວລາ:<br>___ days วัน ມື້<br>___ weeks สัปดาห์ ອາທິດ<br>___ months เดือน ເດືອນ	___ days วัน ມື້<br>___ weeks สัปดาห์ ອາທິດ<br>___ months เดือน ເດືອນ	___ days วัน ມື້<br>___ weeks สัปดาห์ ອາທິດ<br>___ months เดือน ເດືອນ
e) Why did you seek this advice/treatment? เพราะเหตุใดท่านจึงเลือกรักษา/รับคำแนะนำนี้ ເປັນຫຍັງທ່ານຈຶ່ງເລືອກການປິ່ນປົວ/ຮັບຄຳແນະນຳນີ້<br><br>[*Mark all that apply*] [*ตอบได้มากกว่า 1 ตัวเลือก*] [*ຕອບໄດ້ຫຼາຍກ່ວາ 1 ຕົວເລືອກ*]	This is the normal choice เป็นสิ่งที่ทำอยู่ปกติ ເປັນແນວປະຕິບັດປົກກະຕິ.. 1<br>Advice from others เป็นคำแนะนำจากคนอื่น ເປັນຄຳແນະນຳຈາກຄົນອື່ນ.. 2<br>Too busy for alternatives ไม่มีตัวเลือกอื่นเพราะไม่ว่าง ບໍ່ມີທາງເລືອກອື່ນເນື່ອງຈາກບໍ່ມີເວລາ .... 3<br>No access to money/transportation มีปัญหาเรื่องเงินหรือการเดินทาง ມີບັນຫາກ່ຽວກັບເງິນຫຼືການເດີນທາງ .... 4<br>Ran out of medicines ยาที่ใช้อยู่หมด ຢາທີ່ໃຊ້ຢູ່ຫມົດ .... 5<br>Not feeling better พยายามรักษาด้วยวิธีอื่นแล้วไม่หาย ປິ່ນປົວໂດຍວິທີອື່ນແລ້ວບໍ່ດີຂຶ້ນ.. 6<br>New/more serious symptoms อาการหนักลงกว่าเก่าหรือมีอาการอื่นปรากฏ ອາການຫນັກລົງກວ່າເກົ່າຫຼືມີອາການອື່ນປາກົດເພີ່ມຂຶ້ນ .... 7<br>Other (specify) อื่นๆ (โปรดระบุ) ອື່ນໆ (ກະລຸນາບອກ) _ 8	1<br>2<br>3<br>4<br>5<br>6<br>7<br>8	1<br>2<br>3<br>4<br>5<br>6<br>7<br>8

Haenssgen MJ, *et al. BMJ Open* 2019; 9:e028224. doi: 10.1136/bmjopen-2018-028224

OxTREC reference: 528-17 **ANTIBIOTICS AND ACTIVITY SPACES**

f) Can you please name or describe all the medicines that you received or were prescribed during this step? กรุณาระบุชื่อหรืออธิบายลักษณะของยาทั้งหมดที่ท่านได้รับจากการรักษาขั้นตอนนี้ ກະລຸນາບອກຊື່ຫຼືອະທິບາຍລັກສະນະຂອງຢາທີ່ທ່ານໄດ້ຮັບຈາກການປິ່ນປົວຂັ້ນຕອນນີ້<br>[*leave blank if no medicine received*] [*include medicine stored at home if "self-care at home"*] [*continue for all medicines received, then complete Questions g to k for each medicine individually*] [เว้นช่องว่างหากผู้ให้สัมภาษณ์ไม่ได้รับยาจากการรักษาในขั้นตอนนี้] [รวมถึงยาที่มีเก็บไว้ที่บ้านด้วยหากผู้ให้สัมภาษณ์รักษาตัวเองอยู่ที่บ้าน] [บันทึกยาทุกชนิดที่ผู้ให้สัมภาษณ์ได้รับ จากนั้นกรอกข้อมูลในคำถาม g ถึง k สำหรับยาแต่ละชนิด] [*ຈົ່ງຊ່ອງຫວ່າງຖ້າຜູ້ໃຫ້ສຳພາດບໍ່ໄດ້ຮັບຢາຈາກການປິ່ນປົວໃນຂັ້ນຕອນນີ້*] [*ຮວມຢາທີ່ມີເກັບຢູ່ເຮືອນດ້ວຍ ຖ້າຫາກຜູ້ໃຫ້ສຳພາດປົວຕົນເອງຢູ່ເຮືອນ*] [*ບັນທຶກຢາທຸກຊະນິດທີ່ຜູ້ໃຫ້ສຳພາດໄດ້ຮັບ ຈາກນັ້ນຕື່ມຂໍ້ມູນໃສ່ໃນຄຳຖາມ g ເຖິງ k ສຳລັບຢາແຕ່ລະຊະນິດ*]	Medicine 1 ยาชนิดที่ 1 ຢາຊະນິດທີ່ 1:<br>Name/description ชื่อ/คำอธิบาย ຊື່/ຄຳອະທິບາຍ:<br>________________<br>Medicine 2 ยาชนิดที่ 2 ຢາຊະນິດທີ່ 2:<br>Name/description ชื่อ/คำอธิบาย ຊື່/ຄຳອະທິບາຍ:<br>________________<br>Medicine 3 ยาชนิดที่ 3 ຢາຊະນິດທີ່ 3:<br>Name/description ชื่อ/คำอธิบาย ຊື່/ຄຳອະທິບາຍ:<br>________________	Medicine 1 ยาชนิดที่ 1 ຢາຊະນິດທີ່ 1<br>Medicine 2 ยาชนิดที่ 2 ຢາຊະນິດທີ່ 2<br>Medicine 3 ยาชนิดที่ 3 ຢາຊະນິດທີ່ 3	Medicine 1 ยาชนิดที่ 1 ຢາຊະນິດທີ່ 1<br>Medicine 2 ยาชนิดที่ 2 ຢາຊະນິດທີ່ 2<br>Medicine 3 ยาชนิดที่ 3 ຢາຊະນິດທີ່ 3
[*for each medicine individually*] [สำหรับยาแต่ละชนิด] [*ສຳລັບຢາແຕ່ລະຊະນິດ*]<br>g) For how long did you take the medicine? ท่านใช้ยาชนิดนี้อยู่นานเท่าใด ທ່ານໃຊ້ຢາຊະນິດນີ້ດົນປານໃດ<br>[*let respondent choose category; if more than one repeated episode, indicate total duration*] [ให้ผู้ให้สัมภาษณ์เลือกหน่วยเวลาเอง หากมีการใช้ยาตัวเดียวกันหลายครั้ง ให้ผู้สัมภาษณ์รวมระยะเวลาทั้งหมด] [*ໃຫ້ຜູ້ໃຫ້ສຳພາດເລືອກໜ່ວຍເວລາເອງ ຖ້າມີການໃຊ້ຕົວຢາຊະນິດດຽວກັນຫຼາຍເທື່ອໃຫ້ຜູ້ສຳພາດລວມໄລຍະເວລາທັງໝົດ*]	Duration ระยะเวลา ໄລຍະເວລາ:<br>___ days วัน ມື້<br>___ weeks สัปดาห์ ອາທິດ<br>___ months เดือน ເດືອນ	___ days วัน ມື້<br>___ weeks สัปดาห์ ອາທິດ<br>___ months เดือน ເດືອນ	___ days วัน ມື້<br>___ weeks สัปดาห์ ອາທິດ<br>___ months เดือน ເດືອນ
h) How often per day did you take the medicine? ท่านใช้ยาชนิดนี้วันละกี่ครั้ง ທ່ານໃຊ້ຢາຊະນິດນີ້ມືລະຈັກເທື່ອ<br>[*calculate into daily use according to respondent's chosen frequency*]<br>[คำนวณเป็นจำนวนครั้งต่อวันจากคำตอบของผู้ให้สัมภาษณ์]<br>[*ຄິດໄລ່ເປັນຈຳນວນເທື່ອຕໍ່ມື້ຈາກຄຳຕອບຂອງຜູ້ໃຫ້ສຳພາດ*]	Frequency ความถี่ ຄວາມຖີ່: ___ times daily ครั้งต่อวัน ເທື່ອຕໍ່ມື້	___ times daily ครั้งต่อวัน ເທື່ອຕໍ່ມື້	___ times daily ครั้งต่อวัน ເທື່ອຕໍ່ມື້

Haenssgen MJ, *et al. BMJ Open* 2019; 9:e028224. doi: 10.1136/bmjopen-2018-028224

OxTREC reference: 528-17 **ANTIBIOTICS AND ACTIVITY SPACES**

i) What dosage did you normally take? ท่านใช้ยาชนิดนี้ในปริมาณเท่าใดต่อหนึ่งครั้ง ທ່ານໃຊ້ຢາຊະນິດນີ້ປະລິມານເທົ່າໃດຕໍ່ໜຶ່ງເທື່ອ<br>[*let respondent choose category according to type of medicine*] [*ให้ผู้ให้สัมภาษณ์เลือกหน่วยปริมาณยาเอง โดยขึ้นอยู่กับชนิดของยา*] [*ໃຫ້ຜູ້ໃຫ້ສໍາພາດເລືອກໜ່ວຍປະລິມານຢາເອງ ໂດຍຂຶ້ນຢູ່ກັບຊະນິດຂອງຢາ*]	Dosage ปริมาณ ຄວາມຖີ່:<br>___ tablets / capsules<br>เม็ด/แคปซูล ເມັດ/ແຄບຊູນ<br>___ drops (for liquid medicine)<br>หยด (สำหรับยาน้ำ) ຢອດ (ສໍາລັບຢານໍ້າ)<br>___ spoons (for liquid medicine)<br>ช้อน (สำหรับยาน้ำ) ບ່ວງ (ສໍາລັບຢານໍ້າ)<br>per time administered ต่อครั้งที่ใช้ ຕໍ່ເທື່ອທີ່ໃຊ້	___ tablets เม็ด ເມັດ<br>___ drops หยด ຢອດ<br>___ spoons ช้อน ບ່ວງ	___ tablets เม็ด ເມັດ<br>___ drops หยด ຢອດ<br>___ spoons ช้อน ບ່ວງ
j) Did you take the medicine exactly as it was recommended to you by the person who prescribed/sold them? ท่านใช้ยาชนิดนี้ตามคำแนะนำของผู้สั่งจ่าย/จำหน่ายหรือไม่ ທ່ານໃຊ້ຢາຊະນິດນີ້ຕາມຄໍາແນະນໍາຈາກໝໍ/ຜູ້ຂາຍຢາຫຼືບໍ່	Yes ใช่ ແມ່ນ ....1<br>No ไม่ใช่ ບໍ່ແມ່ນ....2<br>Did not receive advice ไม่ได้รับคำแนะนำ ບໍ່ໄດ້ຮັບຄໍາແນະນໍາ....9<br>Don't know ไม่ใช่ ບໍ່ແມ່ນ 99	1<br>2<br>9<br>99	1<br>2<br>9<br>99
k) Did you finish the medicine? ท่านใช้ยาชนิดนี้จนหมดหรือไม่ ທ່ານໃຊ້ຢາຊະນິດນີ້ຈົນໝົດຫຼືບໍ່	Yes ใช่ ແມ່ນ .... 1<br>No ไม่ใช่ ບໍ່ແມ່ນ......... 2	1<br>2	1<br>2

Have you now recovered from the illness/accident? ท่านฟื้นตัวจากอาการป่วย/อุบัติเหตุแล้วหรือไม่ ທ່ານດີຈາກອາການເຈັບເປັນ/ອຸບັດຕິເຫດແລ້ວບໍ່?	Yes ใช่ ແມ່ນ ....1<br>No ไม่ใช่ ບໍ່ແມ່ນ 2

Was anybody of your personal relationships involved in providing advice or help during the illness? ท่านได้รับคำปรึกษาหรือความช่วยเหลือในด้านใดๆ ก็ตามจากบุคคลที่เกี่ยวข้องกับท่านระหว่างการป่วยครั้งนี้หรือไม่ ທ່ານໄດ້ຮັບຄໍາປຶກສາຫຼືຄວາມຊ່ວຍເຫຼືອໃນດ້ານໃດໆ ກໍ່ຕາມຈາກບຸກຄົນທີ່ກ່ຽວຂ້ອງກັບທ່ານລະຫ່ວາງການເຈັບເປັນຄັ້ງນີ້ຫຼືບໍ່

[*record up to ten names*] [*กรอกชื่อของบุคคลที่มีความเกี่ยวข้องระหว่างอาการป่วยไม่เกิน 10 คน*] [*ຂຽນຊື່ຂອງບຸກຄົນທີ່ມີຄວາມກ່ຽວຂ້ອງລະຫ່ວາງອາການເຈັບປ່ວຍບໍ່ຫຼາຍກ່ວາ 10 ຄົນ*]

	a) What is the name of the person? ชื่อ ຊື່	b) How is this person related to you? ความสัมพันธ์กับท่าน ຄວາມສໍາພັນກັບທ່ານ?<br>[*give examples if respondent is unsure about answer categories*] [*หากผู้ให้สัมภาษณ์ไม่มั่นใจในหมวดคำตอบ ให้ยกตัวอย่างคำตอบได้*] [*ຖ້າຜູ້ໃຫ້ສໍາພາດບໍ່ໝັ້ນໃຈໃນໝວດຄໍາຕອບ ໃຫ້ຍົກຕົວຢ່າງຄໍາຕອບໄດ້*]	c) What kind of support was provided? *ท่านได้รับความช่วยเหลือจากบุคคลนี้อย่างไร* ທ່ານໄດ້ຮັບການຊ່ວຍເຫຼືອຈາກບຸກຄົນເຫຼົ່ານີ້ແນວໃດ?<br>[*mark all that apply*] [*ตอบได้มากกว่า 1 ตัวเลือก*] [*ຕອບໄດ້ຫຼາຍກ່ວາ 1 ຕົວເລືອກ*]

Haenssgen MJ, *et al. BMJ Open* 2019; 9:e028224. doi: 10.1136/bmjopen-2018-028224

OxTREC reference: 528-17 **ANTIBIOTICS AND ACTIVITY SPACES**

Contact 1 คนที่ 1 ຄົນທີ່ 1	Name ชื่อ ຊື່: ________	Spouse คู่สมรส ຄູ່ແຕ່ງດອງ ....1<br>Parent บิดามารดา ພໍ່ແມ່ ....2<br>Child บุตร ລູກ..................................3<br>Sibling พี่น้อง ອ້າຍນ້ອງ........................4<br>Other relative ญาติ ພີ່ນ້ອງອື່ນໆ...........5<br>Neighbour เพื่อนบ้าน ເພື່ອນບ້ານ............6<br>Friend (if not neighbour) เพื่อน (ที่ไม่ใช่เพื่อนบ้าน) ໝູ່ (ທີ່ບໍ່ແມ່ນເພື່ອນບ້ານ)..........7<br>Other villager ชาวบ้านคนอื่นๆ ຊາວບ້ານຄົນອື່ນໆ.......................................8<br>Other (specify) อื่นๆ (โปรดระบุ) ອື່ນໆ (ກະລຸນາບອກ) _ 9	Providing healthcare/attending ให้การรักษา/การดูแล ໃຫ້ການເບິ່ງແຍງ..11<br>Providing advice ให้คำปรึกษา ໃຫ້ຄຳປຶກສາ ....12<br>Providing medicine ให้ยา ໃຫ້ຢາ ....13<br>Lending/granting money ให้หรือให้ยืมเงิน ໃຫ້ຫຼືໃຫ້ຢືມເງິນ..21<br>Transportation/Lending vehicle อำนวยความสะดวกในด้านการเดินทาง/ให้ยืมพาหนะ ສ້າງຄວາມສະດວກດ້ານການເດີນທາງ/ໃຫ້ຢືມຍານພາຫະນະ ....22<br>Contacting family/friends ติดต่อครอบครัว/เพื่อน ຕິດຕໍ່ຄອບຄົວ/ໝູ່ ....23<br>Providing food สนับสนุนอาหาร ສະໜັບສະໜູນອາຫານ ....31<br>Helping with children/housework ช่วยดูแลลูกหลาน/งานบ้าน ຊ່ວຍເບິ່ງແຍງລູກຫລານຫຼືເດັກນ້ອຍ/ວຽກບ້ານ ....32<br>Helping with jobs/agriculture work (feeding animals/tending crops/covering shifts, etc.) ช่วยเหลือเกี่ยวกับอาชีพ/การเกษตร (ช่วยดูแลสัตว์เลี้ยง/งานเพาะปลูก/ทำงานแทน) ຊ່ວຍເຮັດວຽກ/ການກະສິກຳ (ຊ່ວຍເບິ່ງແຍງສັດລ້ຽງ/ເຮັດໄຮ່ເຮັດນາ/ເຮັດວຽກແທນ)...33<br>Other (specify) อื่นๆ (โปรดระบุ) ອື່ນໆ (ກະລຸນາບອກ) _ ..99
Contact 2 คนที่ 2 ຄົນທີ່ 2	Name ชื่อ ຊື່	1 2 3 4 5 6 7 8 9	11 12 13 21 22 23 31 32 33 99
[*continue until Contact x – maximum 10 contacts*] [บันทึกบุคคลที่มีความเกี่ยวข้องระหว่างการเจ็บป่วยทุกคน ไม่เกิน 10 คน] [*ບັນທຶກບຸກຄົນທີ່ມີຄວາມກ່ຽວຂ້ອງລະຫ່ວາງອາການເຈັບປ່ວຍທຸກຄົນ ບໍ່ຫຼາຍກ່ວາ 10ຄົນ*]	Name ชื่อ ຊື່	1 2 3 4 5 6 7 8 9	11 12 13 21 22 23 31 32 33 99

a. Do you think these questions are too hard? ท่านรู้สึกว่าคำถามเหล่านี้ยากเกินไปหรือไม่ ທ່ານຮູ້ສຶກວ່າຄຳຖາມເຫຼົ່ານີ້ຍາກເກີນໄປຫຼືບໍ່

b. Did you find it tedious to go through the illness in such detail? เป็นเรื่องน่าเบื่อหรือไม่ที่ท่านต้องอธิบายอาการป่วยของท่านโดยละเอียด ເປັນເລື່ອງໜ້າເບື່ອຫຼືບໍ່ທີ່ທ່ານຕ້ອງມາອະທິບາຍອາການບໍ່ສະບາຍຂອງທ່ານຢ່າງລະອຽດ

c. How well do you remember the medicines that you used during that illness? ท่านสามารถจดจำยาที่ท่านใช้ระหว่างการป่วยได้ดีเพียงใด ທ່ານສາມາດຈື່ຢາທີ່ທ່ານໃຊ້ລະຫ່ວາງຕອນເຈັບປ່ວຍ ໄດ້ດີພຽງໃດ

d. Did you think about anything in particular to help you recall the medicines? ที่ท่านนึกถึงอะไรเพื่อเป็นตัวช่วยในการทวนความจำเกี่ยวกับยาเหล่านี้ ທ່ານນຶກເຖິງຫຍັງເປັນຕົວຊ່ວຍໃນການຈື່ກ່ຽວກັບຢາເຫຼົ່ານີ້

e. How do you indicate that the symptoms are cured? Would you stop taking medicines then? What if the doctor's instruction is to complete the dose? ท่านสามารถบอกได้อย่างไรว่าอาการป่วยของท่านหายแล้ว หากอาการป่วยของท่านหายท่านจะหยุดรับประทานยาทันทีหรือไม่ หากแพทย์แนะนำให้ท่านใช้ยาจนหมดแต่อาการของท่านหายแล้วท่านยังจะปฏิบัติตามคำสั่งแพทย์หรือไม่ ທ່ານສາມາດບອກໄດ້ແນວໃດວ່າອາການເຈັບເປັນຂອງທ່ານດີແລ້ວ ຖ້າອາການເຈັບເປັນຂອງທ່ານດີ ທ່ານຈະຢຸດກິນຢາທັນທີຫຼືບໍ່ ຖ້າໝໍແນະນຳໃຫ້ທ່ານໃຊ້ຢາຈົນໝົດແຕ່ອາການຂອງທ່ານດີແລ້ວທ່ານຍັງຈະປະຕິບັດຕາມຄຳສັ່ງໝໍຫຼືບໍ່

10. The next question deals with medicine. Could you please tell me what is going through your mind while you are answering the question? คำถามต่อไปจะเป็นคำถามเกี่ยวกับยารักษาโรค เราขอให้ท่านบอกเราว่าท่านนึกถึงอะไรระหว่างการตอบคำถามเหล่านี้ ຄຳຖາມຕໍ່ໄປຈະເປັນຄຳຖາມກ່ຽວກັບຢາປົວພະຍາດ ເຮົາຂໍໃຫ້ທ່ານບອກເຮົາວ່າທ່ານນຶກເຖິງຫຍັງລະຫ່ວາງການຕອບຄຳຖາມເຫຼົ່ານີ້

Haenssgen MJ, *et al. BMJ Open* 2019; 9:e028224. doi: 10.1136/bmjopen-2018-028224

OxTREC reference: 528-17 **ANTIBIOTICS AND ACTIVITY SPACES**

I would now like to ask you your opinion about medicine. There are no right or wrong answers, I only want to understand what you think. เราอยากทราบความคิดเห็นของท่านเกี่ยวกับการใช้ยา คำถามเหล่านี้ไม่มีคำตอบที่ถูกหรือผิด เราเพียงต้องการรู้ว่าท่านคิดอย่างไร ເຮົາຢາກຮູ້ຄວາມຄິດເຫັນຂອງທ່ານກ່ຽວກັບການໃຊ້ຢາ ຄຳຖາມເຫຼົ່ານີ້ບໍ່ມີຄຳຕອບຖືກຫຼືຜິດ ເຮົາພຽງຕ້ອງການຮູ້ວ່າທ່ານຄິດແນວໃດ

Consider the following medicines – they are commonly referred to as “antibiotics.”

พิจารณายาเหล่านี้ซึ่งเป็นยาที่ถูกรู้จักในนาม “ยาปฏิชีวนะ” ພິຈາລະນາຢາເຫຼົ່ານີ້ເຊິ່ງເປັນຢາທີ່ຮູ້ຈັກກັນໃນນາມ “ຢາຕ້ານເຊື້ອ”

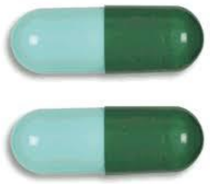
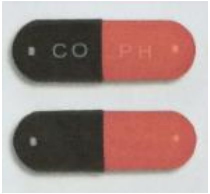

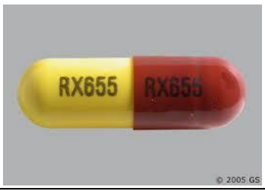

Would you buy this medicine if you think you need it but cannot get it prescribed by a doctor? หากท่านไม่ได้รับยานี้หลังจากเข้าพบแพทย์แม้ท่านรู้สึกว่าท่านควรรับประทานยานี้ ท่านจะซื้อยานี้จากแหล่งอื่นหรือไม่ ຖ້າທ່ານບໍ່ໄດ້ຮັບຢານີ້ຫຼັງຈາກເຂົ້າພົບໝໍເຖິງທ່ານຈະຮູ້ສຶກວ່າທ່ານຄວນກິນຢານີ້ ທ່ານຈະຊື້ຢານີ້ຈາກແຫຼ່ງອື່ນຫຼືບໍ່	Yes ใช่ ແມ່ນ .... 1<br>No ไม่ใช่ ບໍ່ແມ່ນ .... 2<br>I don’t know ไม่ทราบ ບໍ່ຮູ້ 99
Would you try other remedies such as sponge bath, herbs, or paracetamol before using this medicine? ท่านจะลองใช้วิธีการรักษาอื่น เช่น เช็ดตัว สมุนไพร หรือ พาราเซตามอลก่อนใช้ยานี้หรือไม่ ທ່ານຈະພະຍາຍາມປິ່ນປົວໂດຍວິທີການອື່ນ ເຊັ່ນ ເຊັດຕົວ ຢາຮາກໄມ້ ຫຼືພາຣາເຊຕາມອນກ່ອນໃຊ້ຢານີ້ຫຼືບໍ່	Yes ใช่ ແມ່ນ .... 1<br>No ไม่ใช่ ບໍ່ແມ່ນ .... 2<br>I don’t know ไม่ทราบ ບໍ່ຮູ້ 99
If you had to take this medicine and your symptoms disappear after 2 days, would you finish a complete course of 7 days or stop taking it immediately? หากท่านจำเป็นต้องรับประทานยานี้และอาการของท่านดีขึ้นภายใน 2 วัน ท่านจะรับประทานยาต่อจนครบ 7 วัน หรือหยุดรับประทานทันที ຖ້າຫາກທ່ານມີຄວາມຈຳເປັນຕ້ອງກິນຢານີ້ເພື່ອການປິ່ນປົວ ແລະອາການຂອງທ່ານດີຂຶ້ນພາຍໃນສອງມື້ ທ່ານຈະກິນຢາຕໍ່ຈົນສຸດ 7 ມື້ ຫຼືຢຸດກິນທັນທີ	Finish course รับประทานต่อจนหมด<br>ກິນຢາຕໍ່ຈົນສຸດ .... 1<br>Stop taking หยุดรับประทาน ຢຸດກິນທັນທີ .... 2<br>I don’t know ไม่ทราบ ບໍ່ຮູ້ 99
Do you think the way other people use this medicine affects its efficacy on you? ท่านคิดว่าการใช้ยานี้ของคนอื่นมีผลกระทบต่อผลลัพธ์ของการใช้ยานี้ของท่านหรือไม่ ທ່ານຄິດວ່າການໃຊ້ຢານີ້ຂອງຄົນອື່ນມີຜົນກະທົບກັບສະມັດຖະພາບຂອງການໃຊ້ຢານີ້ຂອງທ່ານຫຼືບໍ່	Yes ใช่ ແມ່ນ .... 1<br>No ไม่ใช่ ບໍ່ແມ່ນ .... 2<br>I don’t know ไม่ทราบ ບໍ່ຮູ້ 99

a. As I have said before, these survey questions are not intended to measure your knowledge. Did you feel tested or pressured being asked these questions at all? เราได้บอกท่านข้างต้นเกี่ยวกับคำถามเหล่านี้ที่ไม่ได้ถูกออกแบบเพื่อเป็นข้อสอบวัดความรู้ ท่านรู้สึกถูกทดสอบหรือกดดันในการตอบคำถามเหล่านี้หรือไม่ ເຮົາໄດ້ບອກທ່ານຂັ້ນຕົ້ນກ່ຽວກັບຄຳຖາມເຫຼົ່ານີ້ທີ່ບໍ່ໄດ້ອອກແບບເພື່ອເປັນບົດເສັງວັດແທກຄວາມຮູ້ ທ່ານຮູ້ສຶກຖືກທົດສອບຫຼືກົດດັນໃນການຕອບຄຳຖາມເຫຼົ່ານີ້ຫຼືບໍ່
b. Have you heard of antibiotics? ท่านเคยได้ยินเกี่ยวกับยาปฏิชีวนะหรือไม่ ທ່ານເຄີຍໄດ້ຍິນກ່ຽວກັບຢາຕ້ານເຊື້ອຫຼືບໍ່
c. Other than the term “antibiotics,” how do you normally call these medicines? นอกจากคำว่า “ยาปฏิชีวนะ” ท่านเรียกยาชนิดนี้ได้ว่าอะไรอีกบ้าง ນອກຈາກຄຳວ່າ ຢາຕ້ານເຊື້ອ ທ່ານເອີ້ນຢາຊະນິດນີ້ໄດ້ວ່າຫຍັງອີກແດ່
d. Do you think other villagers will associate the pictures with other types of medicine? ท่านคิดว่าชาวบ้านคนอื่นๆ จะนึกถึงยาชนิดอื่นหรือไม่เมื่อเห็นภาพเหล่านี้ ທ່ານຄິດວ່າຊາວບ້ານຄົນອື່ນໆ ຈະນຶກເຖິງຢາຊະນິດອື່ນຫຼືບໍ່ເມື່ອເຫັນພາບເຫຼົ່ານີ້

Haenssgen MJ, *et al. BMJ Open* 2019; 9:e028224. doi: 10.1136/bmjopen-2018-028224

OxTREC reference: 528-17 **ANTIBIOTICS AND ACTIVITY SPACES**

Part V – Household-Related Survey Questions ส่วนที่ 5 – คำถามเกี่ยวกับครัวเรือน ສ່ວນທີ່ 5 - ຄຳຖາມກ່ຽວກັບຄົວເຮືອນ

Thank you. We now come to the last part. ขอบคุณครับ/ค่ะ ส่วนต่อไปเป็นส่วนสุดท้ายของการสัมภาษณ์ ຂອບໃຈ ສ່ວນຕໍ່ເປັນເປັນສ່ວນສຸດທ້າຍຂອງການສຳພາດ

11. The following questions relate to your household. คำถามต่อไปนี้เป็นคำถามเกี่ยวกับครัวเรือนของท่าน ຄຳຖາມຕໍ່ໄປນີ້ເປັນຄຳຖາມກ່ຽວກັບຄົວເຮືອນຂອງທ່ານ

I will now ask you for some items in your household. Please tell me… ต่อไปเราจะถามท่านเกี่ยวกับสิ่งของที่ท่านครอบครองในครัวเรือน ຕໍ່ໄປເຮົາຈະຖາມກ່ຽວກັບສິ່ງຂອງທີ່ທ່ານຄອບຄອງໃນຄົວເຮືອນ		Number of items in household นวนของสิ่งของที่มีภายในครัวเรือน ຈຳນວນຂອງສິ່ງຂອງທີ່ມີພາຍໃນຄົວເຮືອນ:
	Have you got a *functioning* **radio** in your household? If so, how many? ท่านมี**วิทยุ**ที่ใช้งานได้อยู่ในครัวเรือนของท่านหรือไม่ ถ้ามี กี่เครื่อง ທ່ານມີ**ວິທະຍຸ**ທີ່ໃຊ້ງານໄດ້ໃນຄົວເຮືອນຂອງທ່ານຫຼືບໍ່ ຖ້າມີ ຈັກອັນ	________
	Have you got a *functioning* **TV** in your household? If so, how many? ท่านมี**โทรทัศน์**ที่ใช้งานได้อยู่ในครัวเรือนของท่านหรือไม่ ถ้ามี กี่เครื่อง ທ່ານມີ**ທໍລະທັດ**ທີ່ໃຊ້ງານໄດ້ຢູ່ໃນຄົວເຮືອນຂອງທ່ານຫຼືບໍ່ ຖ້າມີ ຈັກອັນ	________
	Have you got a *functioning* **rice cooker** in your household? If so, how many? ท่านมี**หม้อหุงข้าว**ที่ใช้งานได้อยู่ในครัวเรือนของท่านหรือไม่ ถ้ามี กี่เครื่อง ທ່ານມີ**ໝໍ້ຫຸງເຂົ້າ**ທີ່ໃຊ້ງານໄດ້ຢູ່ໃນຄົວເຮືອນຂອງທ່ານຫຼືບໍ່ ຖ້າມີ ຈັກອັນ	________
	Have you got a *functioning* **landline telephone** in your household? If so, how many? ท่านมี**โทรศัพท์บ้าน**ที่ใช้งานได้อยู่ในครัวเรือนของท่านหรือไม่ ถ้ามี กี่เครื่อง ທ່ານມີ**ໂທລະສັບຕັ້ງໂຕະ**ທີ່ໃຊ້ງານໄດ້ຢູ່ໃນຄົວເຮືອນຂອງທ່ານຫຼືບໍ່ ຖ້າມີ ຈັກອັນ	________
	Have you got a *functioning* **mobile phone** in your household? If so, how many? ท่านมี**โทรศัพท์มือถือ**ที่ใช้งานได้อยู่ในครัวเรือนของท่านหรือไม่ ถ้ามี กี่เครื่อง ທ່ານມີ**ໂທລະສັບມືຖື**ທີ່ໃຊ້ງານໄດ້ຢູ່ໃນຄົວເຮືອນຂອງທ່ານຫຼືບໍ່ ຖ້າມີ ຈັກອັນ	________
	Have you got a *functioning* **computer** in your household? If so, how many? ท่านมี**คอมพิวเตอร์**ที่ใช้งานได้อยู่ในครัวเรือนของท่านหรือไม่ ถ้ามี กี่เครื่อง ທ່ານມີ**ຄອມພິວເຕີ**ທີ່ໃຊ້ງານໄດ້ຢູ່ໃນຄົວເຮືອນຂອງທ່ານຫຼືບໍ່ ຖ້າມີ ຈັກອັນ	________
	Have you got a *functioning* **bicycle** in your household? If so, how many? ท่านมี**รถจักรยาน**ที่ใช้งานได้อยู่ในครัวเรือนของท่านหรือไม่ ถ้ามี กี่คัน ທ່ານມີ**ລົດຖີບ**ທີ່ໃຊ້ງານໄດ້ຢູ່ໃນຄົວເຮືອນຂອງທ່ານຫຼືບໍ່ ຖ້າມີ ຈັກຄັນ	________
	Have you got a *functioning* **scooter, motorcycle, or tricycle** in your household? If so, how many? ท่านมี**จักรยานยนต์หรือรถสามล้อ**ที่ใช้งานได้อยู่ในครัวเรือนของท่านหรือไม่ ถ้ามี กี่คัน ທ່ານມີ**ລົດຈັກຫຼືລົດສາມລໍ້**ທີ່ໃຊ້ງານໄດ້ຢູ່ໃນຄົວເຮືອນຂອງທ່ານຫຼືບໍ່ ຖ້າມີ ຈັກຄັນ	________
	Have you got a *functioning* **car or truck** in your household? If so, how many? ท่านมี**รถยนต์ รถกระบะ หรือรถบรรทุก**ที่ใช้งานได้อยู่ในครัวเรือนของท่านหรือไม่ ถ้ามี กี่คัน ທ່ານມີ**ລົດໃຫ່ຍ ລົດກະບະ ຫຼືລົດບັນທຸກ**ທີ່ໃຊ້ງານໄດ້ຢູ່ໃນຄົວເຮືອນຂອງທ່ານຫຼືບໍ່ ຖ້າມີ ຈັກຄັນ	________
	Have you got a *functioning* **tractor** in your household? If so, how many? ท่านมี**รถแทรกเตอร์หรือรถไถนา**ที่ใช้งานได้อยู่ในครัวเรือนของท่านหรือไม่ ถ้ามี กี่คัน ທ່ານມີ**ລົດໄຖນາ**ທີ່ໃຊ້ງານໄດ້ຢູ່ໃນຄົວເຮືອນຂອງທ່ານຫຼືບໍ່ ຖ້າມີ ຈັກຄັນ	________
	Have you got a *functioning* **refrigerator or freezer** in your household? If so, how many? ท่านมี**ตู้เย็นหรือตู้แช่เข็ง**ที่ใช้งานได้อยู่ในครัวเรือนของท่านหรือไม่ ถ้ามี กี่ตู้ ທ່ານມີ**ຕູ້ເຢັນຫຼືຕູ້ແຊ່ແຂງ**ທີ່ໃຊ້ງານໄດ້ຢູ່ໃນຄົວເຮືອນຂອງທ່ານຫຼືບໍ່ ຖ້າມີ ຈັກຕູ້	________

a. Do you feel comfortable (or uncomfortable) recalling these points for your household? ท่านรู้สึกสบายใจ (หรืออึดอัดใจ) ในการนึกถึงข้าวของเครื่องใช้เหล่านี้ในครัวเรือนของท่านหรือไม่ ທ່ານຮູ້ສຶກສະບາຍໃຈ (ຫຼືອຶດອັດໃຈ) ໃນການນຶກເຖິງເຄື່ອງໃຊ້ເຫຼົ່ານີ້ໃນຄົວເຮືອນຂອງທ່ານຫຼືບໍ່
b. Are there some common household items that you normally see in your village? ข้าวของเครื่องใช้ที่เรากล่าวมาข้างต้นเป็นสิ่งที่คนในหมู่บ้านนี้ใช้กันทั่วไปหรือไม่ ເຄື່ອງໃຊ້ທີ່ເຮົາກ່າວມາໃນຂັ້ນຕົ້ນເປັນສິ່ງທີ່ຄົນໃນໝູ່ບ້ານນີ້ໃຊ້ທົ່ວໄປຫຼືບໍ່
c. How would you distinguish whether somebody in your village is wealthy or not?

20 June 2017. Version No: 2.0

ในหมู่บ้านของท่าน ท่านสามารถแยกได้อย่างไรว่าใครรวยหรือจน ໃນໝູ່ບ້ານຂອງທ່ານ ທ່ານສາມາດແຍກໄດ້ແນວໃດວ່າໃຜລວຍຫຼືທຸກ

d. If you had to group these houses into 2 categories, rich and poor, how would you do that? [Sample pictures will be provided] หากท่านต้องจัดกลุ่มบ้านในภาพเหล่านี้ออกเป็นสองกลุ่ม ได้แก่กลุ่มรวยและกลุ่มจน ท่านจะจัดอย่างไร [แสดงบัตรภาพให้ผู้ให้สัมภาษณ์จัดกลุ่ม] ຫາກທ່ານຕ້ອງຈັດກຸ່ມເຮືອນໃນຮູບເຫຼົ່ານີ້ອອກເປັນສອງກຸ່ມໄດ້ແກ່ກຸ່ມລວຍແລະກຸ່ມທຸກ ທ່ານຈະຈັດແນວໃດ [ສະແດງບັດພາບໃຫ້ຜູ້ສຳພາດຈັດກຸ່ມ]

e. What kinds of "meals" did you have in mind when you answered the question? ตอนที่ท่านตอบคำถามเราเรื่องมื้ออาหารที่ท่านรับประทานสัปดาห์ที่แล้ว ท่านจินตนาการภาพถึงมื้ออาหารลักษณะใด ຕອນທ່ານຕອບຄຳຖາມເຮົາເລື່ອງຄາບອາຫານທີ່ທ່ານກິນໃນອາທິດທີ່ແລ້ວ ທ່ານຈິນຕະນາການພາບເຖິງຄາບອາຫານລັກຊະນະໃດ

12. And these are the last two questions. I would like to ask you once more to share your thoughts as you answer these questions. และคำถามสองคำถามสุดท้าย เราต้องการขอให้ท่านเล่าให้เราฟังอีกครั้งว่าท่านคิดอะไรบ้างระหว่างการตอบคำถามต่อไปนี้ ແລະຄຳຖາມສອງຄຳຖາມສຸດທ້າຍ ເຮົາຂໍໃຫ້ທ່ານເລົ່າໃຫ້ເຮົາຟັງອີກເທື່ອໜຶ່ງວ່າທ່ານຄິດຫຍັງແດ່ລະຫ່ວາງການຕອບຄຳຖາມເຫຼົ່ານີ້

And finally the last two questions: Have you heard about drug resistance? คำถามสองข้อสุดท้าย: ท่านเคยได้ยินเกี่ยวกับการดื้อยาหรือไม่ ຄຳຖາມສອງຂໍ້ສຸດທ້າຍ: ທ່ານເຄີຍໄດ້ຍິນກ່ຽວກັບການດື້ຢາຫຼືບໍ່	Yes เคย ເຄີຍ.......... 1<br>No ไม่เคย ບໍ່ເຄີຍ 2
What do you think is drug resistance? ท่านคิดว่าการดื้อยาคืออะไร ທ່ານຄິດວ່າການດື້ຢາແມ່ນຫຍັງ	Answer relates to antibiotic or antimicrobial resistance คำตอบสอดคล้องกับการดื้อยาต้านจุลชีพ ຄຳຕອບສອດຄ່ອງກັບການດື້ຢາຕ້ານຈຸລະຊີບ.......... 1<br>Answer relates to resistance to other types of drugs คำตอบสอดคล้องกับการดื้อยาชนิดอื่นๆ ຄຳຕອບສອງຄ່ອງກັບການດື້ຢາຊະນິດອື່ນໆ .... 2<br>Answer does not relate to drug resistance คำตอบไม่สอดคล้องกับการดื้อยา ຄຳຕອບບໍ່ສອດຄ່ອງກັບການດື້ຢາ.......... 3<br>"Don't know" ไม่ทราบ ບໍ່ຮູ້ 99

a. What your intuitive understanding of "drug resistance?" เมื่อพูดถึง "การดื้อยา" ท่านเข้าใจความหมายของคำคำนี้อย่างไร ເມື່ອເວົ້າເຖິງ "ການດື້ຢາ" ທ່ານເຂົ້າໃຈຄວາມໝາຍຂອງຄຳນີ້ແນວໃດ

b. Do you think other villagers will translate the meaning of "drug resistance" differently from you? ท่านคิดว่าชาวบ้านคนอื่นๆ จะแปลความหมายของคำว่า "ดื้อยา" แตกต่างจากท่านหรือไม่ ທ່ານຄິດວ່າຊາວບ້ານຄົນອື່ນ ຈະແປຄວາມໝາຍຂອງຄຳວ່າ "ດື້ຢາ" ແຕກຕ່າງຈາກທ່ານຫຼືບໍ່

c. Did you feel pressured answering these questions? ท่านรู้สึกกดดันในการตอบคำถามนี้หรือไม่ ທ່ານຮູ້ສຶກກົດດັນໃນການຕອບຄຳຖາມເຫຼົ່ານີ້ຫຼືບໍ່

We are coming to the end of our interview. Thank you so much for sharing your views with me. Your responses have been very helpful. การสัมภาษณ์นี้สิ้นสุดลงแล้ว คำตอบที่ท่านให้กับเราเป็นประโยชน์กับการวิจัยของเราอย่างมาก ขอบคุณสำหรับความคิดเห็นที่ท่านแบ่งปันกับเรา ການສຳພາດນີ້ສິ້ນສຸດແລ້ວ ຄຳຕອບທີ່ທ່ານໃຫ້ກັບເຮົາຈະເປັນປະໂຫຍດກັບການຄົ້ນຄ້ວາຂອງເຮົາຫຼາຍ ຂອບໃຈສຳລັບຄຳຄິດເຫັນທີ່ທ່ານແບ່ງປັນກັບເຮົາ

Haenssgen MJ, *et al. BMJ Open* 2019; 9:e028224. doi: 10.1136/bmjopen-2018-028224
