# Supplementary material for: Antibiotic knowledge, attitudes and practices: new insights from cross-sectional rural health behaviour surveys in low-income and middle-income South-East Asia
Source: BMJ Open. 2019 Aug 20;9(8):e028224. doi: 10.1136/bmjopen-2018-028224 (PMC6707701; doi:10.1136/bmjopen-2018-028224)
Supplement: Supplementary data [file bmjopen-2018-028224supp004.pdf]

Appendix

Appendix Table 1. Provincial-level estimates of rural surveys in Chiang Rai and Salavan.

	Chiang Rai	Salavan	X^2 / z-score
**Demographics**			
Number	1158	983	..
Female[a]	51.3% (44.9–57.6)	50.9% (47.0–54.9)	0.01
Age[a]	46 (13)	37 (20)	10.83***
Education (years)	6.3 (4.5)	4.4 (5.5)	2.10**
Speaking Thai / Lao	92.4% (89.9–94.2)	93.6% (91.9–94.9)	0.88
Wealth index	0.7 (0.1)	0.4 (0.2)	13.12***
Buddhist religion	81.9% (77.4–85.7)	67.5% (61.7–72.9)	16.85***
Thai/Lao nationality	95.0% (93.1–96.4)	98.8% (97.7–99.4)	18.50***
Majority ethnic group (Thai/Lao Loum)	65.2% (59.6–70.4)	56.2% (49.9–62.2)	4.73**
**Antibiotic knowledge / attitudes**			
Number	1158	983	..
Aware of antibiotics	95.7% (94.0–96.9)	86.4% (83.6–88.7)	41.47***
Aware of drug resistance[b]	74.8% (71.1–78.2)	62.5% (58.1–66.7)	18.80***
Would not buy antibiotics over the counter	57.0% (52.7–61.1)	27.7% (24.6–31.0)	115.03***
Prefers antibiotics over alternatives	61.8% (57.9–65.5)	24.8% (21.1–29.0)	151.35***
Does not keep antibiotics for future use	57.1% (53.1–61.0)	16.2% (13.2–19.8)	201.30***
Knows that antibiotic resistance can spread	9.1% (7.2–11.5)	3.4% (2.1–5.5)	15.09***
Answer score (0 to 4)	1.8 (0.9)	0.7 (1.0)	17.13***
**Illness episodes[c]**			
Number	608	356	..
Self-rated severity (1=mild, 2=medium, 3=severe)	1.6 (0.7)	1.8 (0.8)	2.43**
Duration of illness episode (days)	6.8 (7.1)	6.5 (7.9)	0.82
**Treatment-seeking behaviour[c]**			
Number	608	356	..
Public healthcare provider	29.0% (24.8–33.7)	44.8% (37.8–52.0)	14.17***
Private healthcare providers	25.0% (20.5–30.1)	23.8% (17.8–31.0)	0.08
Informal healthcare provider	8.5% (6.1–11.8)	6.9% (3.9–11.8)	0.45
Care from family or self-care	88.8% (84.3–92.2)	93.2% (88.3–96.1)	2.27
Other types of healthcare access	0.3% (0.1–1.4)	6.0% (2.8–12.3)	23.43***
**Medicine use episodes per illness[c]**			
Number	608	356	..
Medicine use episodes	2.2 (1.7)	2.5 (2.3)	1.96**
Non-antibiotic medicine use episodes	1.6 (1.2)	1.3 (1.4)	1.98**
Antibiotic use episodes	0.2 (0.5)	0.4 (0.7)	3.51***
Potential antibiotic use episodes	0.4 (0.9)	0.9 (1.8)	4.32***
Antibiotic use episodes per illness from public sources	0.1 (0.3)	0.1 (0.4)	0.78
Antibiotic use episodes per illness from private sources	0.1 (0.3)	0.2 (0.5)	1.65*
Antibiotic use episodes per illness from informal sources	0.0 (0.2)	0.1 (0.4)	1.69*
Antibiotic / potential antibiotic use episodes from public sources	0.3 (0.8)	0.3 (1.1)	0.57
Antibiotic / potential antibiotic use episodes from private sources	0.2 (0.6)	0.7 (1.5)	3.11***
Antibiotic / potential antibiotic use episodes from informal sources	0.1 (0.3)	0.2 (0.7)	2.33**

Source: Authors' analysis of survey data.

Notes: Population-weighted statistics, accounting for complex survey design. Not applicable categories indicated with ".." Group comparison using X^2 tests for binary and Wilcoxon rank-sum tests for non-normally distributed variables.

*p < 0.1, **p < 0.05, ***p < 0.01.

a. Due to population weighting, samples reflect the same sex and age profiles as the respective censuses.

b. Comparing Thai "due yah" with the combined Lao "due yah" and "lueng yah."

c. Completed illnesses experienced by respondent or child under their supervision, excluding incomplete episodes.
