# Supplementary material for: Antibiotic knowledge, attitudes and practices: new insights from cross-sectional rural health behaviour surveys in low-income and middle-income South-East Asia
Source: BMJ Open. 2019 Aug 20;9(8):e028224. doi: 10.1136/bmjopen-2018-028224 (PMC6707701; doi:10.1136/bmjopen-2018-028224)
Supplement: Author's manuscript [file bmjopen-2018-028224.draft_revisions.pdf]

BMJ Open is committed to open peer review. As part of this commitment we make the peer review history of every article we publish publicly available.

When an article is published we post the peer reviewers' comments and the authors' responses online. We also post the versions of the paper that were used during peer review. These are the versions that the peer review comments apply to.

The versions of the paper that follow are the versions that were submitted during the peer review process. They are not the versions of record or the final published versions. They should not be cited or distributed as the published version of this manuscript.

BMJ Open is an open access journal and the full, final, typeset and author-corrected version of record of the manuscript is available on our site with no access controls, subscription charges or pay-per-view fees (http://bmjopen.bmj.com).

If you have any questions on BMJ Open's open peer review process please email info.bmjopen@bmj.com

BMJ Open

Antibiotic Knowledge, Attitudes, and Practices: New Insights from Representative Social Surveys in Low- and Middle-Income Southeast Asia

Journal:	*BMJ Open*
Manuscript ID	bmjopen-2018-028224
Article Type:	Research
Date Submitted by the Author:	04-Dec-2018

Complete List of Authors:	Haenssgen, Marco; University of Oxford Centre for Tropical Medicine, Center for Tropical Medicine and Global Health; University of Warwick, School of Cross Faculty Studies<br>Charoenboon, Nutcha; Mahidol Oxford Tropical Medicine Research Unit<br>Zanello, Giacomo; University of Reading, School of Agriculture, Policy and Development; Leverhulme Centre for Integrative Research on Agriculture and Health<br>Mayxay, Mayfong; Lao-Oxford-Mahosot Hospital-Wellcome Trust Research Unit; Faculty of Postgraduate Studies, University of Health Sciences<br>Reed-Tsochas, Felix; University of Oxford Said Business School, Said Business School; University of Oxford, Oxford Martin School<br>Lubell, Yoel ; University of Oxford Centre for Tropical Medicine, Center for Tropical Medicine and Global Health; Mahidol Oxford Tropical Medicine Research Unit<br>Wertheim, Heiman; Radbuod University, Medical Microbiology Department; Oxford University Clinical Research Unit<br>Lienert, Jeffrey; University of Oxford Said Business School, ; National Human Genome Research Institute,<br>Xayavong, Thipphaphone; University for Peace, Department of Peace and Conflict Studies; Ateneo de Manila University, Department of Political Science<br>Khine Zaw, Yuzana; London School of Hygiene and Tropical Medicine, Department of Global Health and Development<br>Thepkhamkong, Amphayvone; Lao-Oxford-Mahosot Hospital-Wellcome Trust Research Unit<br>Sithongdeng, Nicksan; Lao-Oxford-Mahosot Hospital-Wellcome Trust Research Unit<br>Khamsoukthavong, Nid; Lao-Oxford-Mahosot Hospital-Wellcome Trust Research Unit<br>Phanthavong, Chanthasone; Lao-Oxford-Mahosot Hospital-Wellcome Trust Research Unit<br>Boualaiseng, Somsanith; Lao-Oxford-Mahosot Hospital-Wellcome Trust Research Unit<br>Vongsavang, Souksakhone; Lao-Oxford-Mahosot Hospital-Wellcome Trust Research Unit<br>Wibunjak, Kanokporn; Mahidol Oxford Tropical Medicine Research Unit<br>Chai-in, Poowadon; Mahidol Oxford Tropical Medicine Research Unit,<br>Thavethanutthanawin, Patthanan; Mahidol Oxford Tropical Medicine

	Research Unit<br>Althaus, Thomas; University of Oxford Centre for Tropical Medicine, Center for Tropical Medicine and Global Health; Mahidol Oxford Tropical Medicine Research Unit<br>Greer, Rachel; Mahidol Oxford Tropical Medicine Research Unit, Faculty of Tropical Medicine Mahidol University; Chiangrai Regional Hospital, Chiangrai Clinical Research Unit<br>Nedsuwan, Supalert; Chiangrai Regional Hospital, Primary Care Department<br>Wangrangsimakul, Tri; Mahidol Oxford Tropical Medicine Research Unit; Chiangrai Regional Hospital, Chiangrai Clinical Research Unit<br>Limmathurotsakul, Direk; Mahidol Oxford Tropical Medicine Research Unit,<br>Elliott, Elizabeth; University College London, UCL Anthropology; Institut de recherche pour le développement<br>Ariana, Proochista; University of Oxford Centre for Tropical Medicine, Center for Tropical Medicine and Global Health
Keywords:	Health policy < HEALTH SERVICES ADMINISTRATION & MANAGEMENT, TROPICAL MEDICINE, PUBLIC HEALTH

SCHOLARONE™ Manuscripts

Antibiotic Knowledge, Attitudes, and Practices: New Insights from Representative Social Surveys in Low- and Middle-Income Southeast Asia

Marco J HAENSSGEN [a, b, c, d], Nutcha CHAROENBOON [d], Giacomo ZANELLO [e, f], Mayfong MAYXAY [a, g, h], Felix REED-TSOCHAS [b, c, i], Yoel LUBELL [a, d], Heiman FL WERTHEIM [j, k], Jeffrey LIENERT [b, c, l], Thipphaphone XAYAVONG [m, n], Yuzana KHINE ZAW [o], Amphayvone THEPKHAMKONG [g], Nicksan SITHONGDENG [g], Nid KHAMSOUKTHAVONG [g], Chanthasone PHANTHAVONG [g], Somsanith BOUALAISENG [g], Souksakhone VONGSAVANG [g], Kanokporn WIBUNJAK [d], Poowadon CHAI-IN [d], Patthanan THAVETHANUTTHANAWIN [d], Thomas ALTHAUS [a, d], Rachel C GREER [a, d], Supalert NEDSUWAN [p], Tri WANGRANGSIMAKUL [a, d], Direk LIMMATHUROTSAKUL [d], Elizabeth ELLIOTT [q, r], Proochista ARIANA [a]

[a.] Centre for Tropical Medicine and Global Health, Nuffield Department of Clinical Medicine, University of Oxford, Oxford, UK
[b.] CABDyN Complexity Centre, Saïd Business School, University of Oxford, Oxford, UK
[c.] Green Templeton College, Oxford, UK
[d.] Mahidol Oxford Tropical Medicine Research Unit, Faculty of Tropical Medicine, Mahidol University, Bangkok, Thailand
[e.] School of Agriculture, Policy and Development, University of Reading, Reading, UK
[f.] Leverhulme Centre for Integrative Research on Agriculture and Health, London, UK
[g.] Lao Oxford Mahosot Wellcome Trust Research Unit (LOMWRU), Mahidol Oxford Tropical Medicine Research Unit (MORU), Faculty of Tropical Medicine, Mahidol University, Bangkok, Thailand
[h.] Institute of Research and Education Development, University of Health Sciences, Vientiane, Lao PDR
[i.] Oxford Martin School, University of Oxford, Oxford, UK
[j.] Oxford University Clinical Research Unit (OUCRU), Ho Chi Minh City, Viet Nam
[k.] Medical Microbiology Department, Radboudumc, Nijmegen, Netherlands
[l.] National Human Genome Research Institute, National Institutes of Health, Bethesda, MD, US
[m.] Department of Peace and Conflict Studies, University for Peace, Costa Rica
[n.] Department of Political Science, School of Social Science, Ateneo de Manila University, Manila, Philippines
[o.] Department of Global Health and Development, London School of Hygiene & Tropical Medicine, London, UK
[p.] Primary Care Department, Chiangrai Prachanukroh Hospital, Chiang Rai, Thailand
[q.] UCL Anthropology, University College London, London, UK
[r.] Institut de recherche pour le développement, Vientiane, Lao PDR

Corresponding author: Marco J HAENSSGEN, Centre for Tropical Medicine and Global Health, Nuffield Department of Medicine, University of Oxford, Old Road Campus, Roosevelt Drive, Oxford OX3 7FZ, UK; phone: +66(0)951906543; email: marco.haenssgen@warwick.ac.uk

WORD COUNT

3,544 (main text, excl. references, figures and tables)

KEYWORDS

Antimicrobial resistance, antibiotics, awareness, treatment-seeking behaviour, survey, social sciences, development studies, interdisciplinary research, Thailand, Lao PDR

For peer review only

ABSTRACT

Introduction: Low- and middle-income countries (LMICs) are crucial in the global response to antimicrobial resistance (AMR), but diverse health systems, healthcare practices, and cultural conceptions of medicine can complicate global education and awareness-raising campaigns. The social sciences can help understand LMIC contexts but remain underrepresented in AMR research.

Objective: To contribute to the social understanding of AMR-related population behaviour in LMICs.

Design: Observational study: cross-sectional rural health behaviour survey, representative on the population level.

Setting: General rural population in Chiang Rai (Thailand) and Salavan (Lao PDR), surveyed between November 2017 and May 2018.

Participants: 2141 adult members (≥18 years) of the general rural population, representing 712,000 villagers.

Outcome measures: Antibiotic-related knowledge, attitudes, and practices across sites and healthcare access channels.

Findings: Villagers were aware of antibiotics (Chiang Rai: 95.7%; Salavan: 86.4%; $p<0.001$) and drug resistance (Chiang Rai: 74.8%; Salavan: 62.5%; $p<0.001$), but the usage of technical concepts for antibiotics was dwarfed by local expressions like "anti-inflammatory medicine" in Chiang Rai (87.6%; 95% confidence interval: 84.9–90.0) and "*ampi*" in Salavan (75.6%; 95% CI: 71.4–79.4). Attitudes against over-the-counter antibiotics were not linked to lower antibiotic consumption from informal providers (Chiang Rai: $p=0.245$; Salavan: $p=0.695$), but to disproportionately high antibiotic consumption from public healthcare providers in Salavan ($p<0.001$; Chiang Rai: $p=0.374$).

Conclusions: Locally specific conceptions and counter-intuitive practices around antimicrobials can complicate AMR communication efforts and entail unforeseen consequences. Overcoming "knowledge deficits" alone will therefore be insufficient for global AMR behaviour change. We call

For peer review only

for an expansion of behavioural AMR strategies towards "AMR-sensitive interventions" that address context-specific upstream drivers of antimicrobial use (e.g. unemployment insurance) and complement education and awareness campaigns.

Registration: clinicaltrials.gov identifier NCT03241316

STRENGTH AND LIMITATIONS OF THIS STUDY

- Provincial-level representative survey using three-stage stratified cluster random sampling design
- Survey based on preceding qualitative research on antibiotic use in Southeast Asia
- Inclusion of general population enables insights into formal and informal healthcare utilisation
- Cross-sectional analysis of rural health behaviours excludes seasonal change and urban settings
- Two-month recall period enabled greater inclusion but may bias responses towards better educated population groups

MAIN TEXT

Introduction

Antimicrobial resistance (AMR) threatens modern medicine by rendering antimicrobial drugs inefficacious. Multi-faceted global strategies target human, animal, and plant health alongside the environment and food production and safety to respond to this "superbug crisis" [1]. In human health, supply-sided responses include incentives to stimulate drug research and development; action on the demand side intends to limit and target antimicrobial use for instance through new diagnostic technologies, public health intervention to improve vaccine coverage and hygiene, and other antimicrobial stewardship activities like restricted dispensing of antibiotics and prescriber feedback [2-4]. As an interdisciplinary field, the social dimensions of the problem are being recognised in global

For peer review only

AMR policy, which are typically addressed via education and awareness-raising activities aimed at governmental staff, healthcare workers, and the general public [2].

Low- and middle-income countries (LMICs) play an important role in the global response to AMR. However, diverse health systems, healthcare practices, and conceptions related to the use of antimicrobials require social sciences research to understand local contexts and the complexity of human behaviour in LMIC settings. For example, with a focus on the health behaviour of the general public, the anthropological literature suggests that social factors like precarity and discrimination can influence medicine use independently of awareness [5]; psychology and behavioural economics indicate that health decision-making processes interact with the social environment and contextual change to create adverse behavioural biases [6 7]; and communication studies research points at interferences between awareness campaigns and local contexts that can entail unforeseen consequences like politicisation, stigmatisation, or accidentally encouraging the behaviours they try to discourage [8 9]. Such examples underline the possible contribution of the social sciences to AMR, but they remain persistently underrepresented with less than 2% of all AMR-related publications (see Appendix Figure A1 for a time trend) [10]. This is problematic for at least three reasons:

- We currently have an insufficient social science knowledge base for behavioural interventions in AMR—a global health priority that has attracted more than £600 million of AMR expenditure and future commitments [11-13].
- The recent withdrawal of large pharmaceutical companies from antimicrobial research and development [14] threatens the AMR supply-side response, requiring yet more effective action on the demand side.
- More extensive social sciences work can yield novel social innovations as a benefit of disciplinary diversification [15].

The objective of this paper was to contribute to the understanding of LMIC contexts and AMR-related human behaviour from a social science perspective, for which we explored antibiotic-related

For peer review only - http://bmjopen.bmj.com/site/about/guidelines.xhtml

knowledge, attitudes, and practices of the general population in two LMICs. We report findings from a provincial-level representative survey of rural health behaviours as part of the interdisciplinary "Antibiotics and Activity Spaces" project [16].[i] We implemented the study in Southeast Asia, which is characterised as a region "at high risk of the emergence and spread of antibiotic resistance in humans" [17]. With more than 9% of global air passengers and more than 110 million international tourist arrivals in 2016 [18]. the potential of cross-border spread of drug-resistant microbes also gives AMR research in Southeast Asia a global relevance—as the recent importation of multi-drug-resistant *Neisseria gonorrhoeae* to the UK showed [19]. Within Southeast Asia, Thailand and Lao PDR lent themselves for a comparative analysis because of their physical and cultural proximity, and Chiang Rai (Thailand) and Salavan (Lao PDR) in particular had similarly varied terrain and large and ethnically diverse rural populations. The main field site differences were Thailand's more advanced economic and health system context and more established AMR action plan [20].

Methods

Research Design

Our study design was a three-stage stratified cluster random survey (Figure 1): Following the purposive selection of five districts per province, we selected a random sample of 30 primary sampling units (PSUs) per province (six per district), stratified by distance to the nearest district headquarters. The second stage was the selection of an interval sample of 5% but at least 30 of all households in the PSU, which we approximated as residential structures on satellite maps [21]. The third and final stage was the random selection of available household members (one for every five members). At each sampling stage, we substituted unavailable selections (1) with a stratified random replacement for the

---

[i] This paper contributes to the project's research question, "*What are the manifestations and determinants of problematic antibiotic use in patients' healthcare-seeking pathways?*" [16].

For peer review only

random PSU sample, (2) with the nearest available neighbour for the interval sample of households, and (3) with a simple random replacement for the random household member sample (replacement numbers indicated in Figure 1). The cross-sectional data collection took place between November 2017 and May 2018.

[Insert Figure 1 about here]

Figure 1. Survey sites and multi-stage sampling process.

Source: Authors, adapted from Wikimedia Commons [22].
Notes: Unavailable selections at each sampling stage were substituted with a random replacement for the random samples of PSUs and household members, and with the nearest available neighbour for the interval sample of households. One PSU could contain more than one administrative village; if the first-chosen village contained less than 600 houses, then adjacent villages would be included. PSU=Primary Sampling Unit.

Study Population

Our study population was the general adult population of rural Chiang Rai and Salavan (522,000 in Chiang Rai and 190,000 in Salavan as per census data), from whom we drew a representative sample of 1158 villagers in Chiang Rai and 983 in Salavan. We did not specifically sample patients, but we recorded any acute illness episode or accident-related injury if one occurred within the last two months of the interview, both for the respondents and any children under their supervision.

Patient and Public Involvement

This study did not sample patients but only adult members of the general public. The survey instrument was based on preceding qualitative research in Southeast Asia [24 25], in which patients, healthcare providers, and healthy adults participated. This preceding research prompted the research interest in treatment-seeking behaviours and conceptions of medicine and illness among the broader rural population in Southeast Asia. We will disseminate our findings through outreach to policy stakeholders

For peer review only

and local development organisations, through public engagement activities like the World Antibiotic Awareness Week, and though our local network of collaborators in the field sites.

Data Collection

Our survey instrument was a 45-minute face-to-face questionnaire (see Supplemental Material). It was administered on tablets running the survey software SurveyCTO (Dobility Inc., Cambridge, MA, USA) by locally recruited survey teams comprising seven enumerators and two survey supervisors per country, who received five days of full-time classroom and field training. The original English questionnaire was translated into Thai and Lao by the research team, and local translators were recruited for the 228 instances where we encountered language barriers in the village. The questionnaires were piloted in rural Chiang Rai and Salavan, with 50 cognitive interviews supporting the questionnaire development and revision as well as the contextualisation of the survey data (not reported here; interview guide in Supplemental Material) [23].

The questionnaire covered basic demographic and socio-economic information, antibiotic-related knowledge and attitudes, and treatment-seeking behaviour during acute illnesses and accident-related injuries. When measuring people's awareness of antibiotics, we could not simply ask villagers whether they knew what "antibiotics" are, considering that (a) a variety of local terms related to antibiotics, (b) people may be familiar with specific antibiotic brands but not aware of their antibiotic attributes, and (c) the understanding of technical language was uncommon (see Results section for evidence on this point). We therefore asked respondents first if they recognised images of common antibiotics in the field site.[ii] In the 108/1974 (5.5%) of cases where the respondents did not mention "antibiotics," its colloquial equivalents, or the names of specific antibiotic types, we asked them if they had heard about

[ii] Three images on the survey tablet in Chiang Rai and a bag with seven local antibiotics in Salavan (considering the wider range of terms and medicines in circulation). See questionnaire in the Supplementary Material.

For peer review only

"anti-inflammatory drugs" ("ยาแก้อักเสบ" or "*yah kae ak seb*") in Thai and "germ resisters" ("ຢາຕ້ານເຊື້ອ" or "*yah dtan suea*") in Lao as common local notions of "antibiotics." We next asked about the purposes for which the respondent would use these antibiotics, which served as information alongside inputs from local pharmacists to triangulate in later parts of the questionnaire whether the respondent received antibiotics during an illness. However, 752/2986 (25.2%) medicine use episodes could not be confirmed as either antibiotic or non-antibiotic (e.g. "white powder" or "green capsule"). We included these uncertain cases as "potential" antibiotic use episodes to capture behaviour more comprehensively.

Data Analysis

In order to inform the current global health agenda on antibiotic education and awareness raising, we used descriptive statistical analysis to describe the patterns of knowledge, attitudes, and practices across the two field sites, using the variables described in Appendix Table A2. If the common policy narrative holds, then we would expect rural populations in Chiang Rai and Salavan to exhibit:

- low degrees of antibiotic-related knowledge,
- generally high levels of antibiotic consumption especially from informal sources (e.g. unregistered shops selling antibiotics over the counter), and
- lower general antibiotic use and a higher share of supervised antibiotic use from formal healthcare providers among people whose attitudes correspond to awareness-raising messages for AMR (based on FAO/OIE/WHO material, see Appendix Table A2) [1].

We stratified and compared the samples by field site and estimated provincially representative patterns using post-stratification weights based on census data (considering village size and district-specific age and gender composition) and adjusting the results for the multi-stage sampling design with the

For peer review only

help of the SVY suite of commands in Stata 15 (StataCorp, College Station, TX, USA). We separately analysed the full sample and the subset of respondents who reported a recent illness, whereby we tested differences in knowledge, attitudes, and behaviours across sites and across antibiotic access channels with X^2 tests for binary and Wilcoxon rank-sum tests (two-sided) for non-normally distributed variables. We considered p values below 0.05 statistically significant.

Results

Representative statistical data of Chiang Rai and Salavan are presented in Table 1. In terms of socio-demographic characteristics of the rural population in the two provinces, Chiang Rai villagers were older on average (p<0.001), tended to have received more formal education (p<0.001), and had higher asset wealth (p<0.001), while fewer Salavan villagers belonged to the local majority ethnicity (p=0.030).

Respondents' recognition of antibiotics in Chiang Rai was significantly higher than in Salavan, but overall high in both sites (Chiang Rai: 95.7%; Salavan: 86.4%; p<0.001). Recognition of the phrase "drug resistance" was high as well, whereby 74.8% recognised the term in Chiang Rai and 62.5% (p<0.001) recognised either of the two common variations in Salavan. Table 1 further indicates that antibiotic-related knowledge and attitudes aligned more closely with FAO/OIE/WHO messages in Chiang Rai than in Salavan (p<0.001 for all four questions). Across the four questions, respondents in rural Chiang Rai had an average answer score of 1.8 as opposed to rural Salavan with 0.7 (p<0.001).

Figure 2, Panel a demonstrates the ways in which people related to "antibiotics." In Chiang Rai, respondents commonly referred to antibiotics as "anti-inflammatory drug," representing 87.6% of all responses ("ยาแก้อักเสบ" or "*yah kae ak seb*;" a vernacular notion specific to antibiotics).[iii] Only 7.2%

[iii] Actual anti-inflammatory medicine like ibuprofen would usually be referred to by its brand names.

For peer review only

used the official term for "antibiotic" ("ยาปฏิชีวนะ" or "*yah pa ti chee wa na*") alongside "*germ killer*" and specific antibiotic types like "*corlam*" (chloramphenicol; 4.6%). In rural Salavan, a larger portion of 38.6% used the official term for antibiotics ("ຢາຕ້ານເຊື້ອ" or "*yah dtan suea*," translated as "germ resister"), but Salavan respondents were also more likely to use various colloquial expressions for specific types of antibiotics, like "*Ampi*" with 75.6% and "*Amok*" with 35.3%.

Table 1. Provincial-level estimates of rural surveys in Chiang Rai and Salavan.

	Chiang Rai	Salavan	p value
**Demographics**			
Number	1158	983	..
Female[a]	51.3% (44.9–57.6)	50.9% (47.0–54.9)	0.927
Age[a]	46 (13)	37 (20)	<0.001
Education (years)	6.3 (4.5)	4.4 (5.5)	<0.001
Speaking Thai / Lao	92.4% (89.9–94.2)	93.6% (91.9–94.9)	0.348
Wealth index	0.7 (0.1)	0.4 (0.2)	<0.001
Buddhist religion	81.9% (77.4–85.7)	67.5% (61.7–72.9)	<0.001
Thai/Lao nationality	95.0% (93.1–96.4)	98.8% (97.7–99.4)	<0.001
Majority ethnic group (Thai/Lao Loum)	65.2% (59.6–70.4)	56.2% (49.9–62.2)	0.030
**Antibiotic knowledge / attitudes**			
Number	1158	983	..
Aware of antibiotics	95.7% (94.0–96.9)	86.4% (83.6–88.7)	<0.001
Aware of drug resistance[b]	74.8% (71.1–78.2)	62.5% (58.1–66.7)	<0.001
Would not buy antibiotics over the counter	57.0% (52.7–61.1)	27.7% (24.6–31.0)	<0.001
Prefers antibiotics over alternatives	61.8% (57.9–65.5)	24.8% (21.1–29.0)	<0.001
Does not keep antibiotics for future use	57.1% (53.1–61.0)	16.2% (13.2–19.8)	<0.001
Knows that antibiotic resistance can spread	9.1% (7.2–11.5)	3.4% (2.1–5.5)	<0.001
Answer score (0 to 4)	1.8 (0.9)	0.7 (1.0)	<0.001
**Illness episodes[c]**			
Number	608	356	..
Self-rated severity (1=mild, 2=medium, 3=severe)	1.6 (0.7)	1.8 (0.8)	0.015
Duration of illness episode (days)	6.8 (7.1)	6.5 (7.9)	0.413
**Treatment-seeking behaviour[c]**			
Number	608	356	..
Public healthcare provider	29.0% (24.8–33.7)	44.8% (37.8–52.0)	<0.001
Private healthcare provider	25.0% (20.5–30.1)	23.8% (17.8–31.0)	0.777
Informal healthcare provider	8.5% (6.1–11.8)	6.9% (3.9–11.8)	0.504
Care from family or self-care	88.8% (84.3–92.2)	93.2% (88.3–96.1)	0.133
Other types of healthcare access	0.3% (0.1–1.4)	6.0% (2.8–12.3)	<0.001
**Medicine use episodes per illness[c]**			
Number	608	356	..
Medicine use episodes	2.2 (1.7)	2.5 (2.3)	0.050
Non-antibiotic medicine use episodes	1.6 (1.2)	1.3 (1.4)	0.048
Antibiotic use episodes	0.2 (0.5)	0.4 (0.7)	<0.001
Potential antibiotic use episodes	0.4 (0.9)	0.9 (1.8)	<0.001

For peer review only

Antibiotic use episodes per illness from informal sources	0.0 (0.2)	0.1 (0.4)	0.092
Antibiotic / potential antibiotic use episodes from informal sources	0.1 (0.3)	0.2 (0.7)	0.020

Source: Authors' analysis of survey data.
Notes: Population-weighted statistics, accounting for complex survey design. Not applicable categories indicated with ".." Group comparison using X2 tests for binary and Wilcoxon rank-sum tests for non-normally distributed variables.
a. Due to population weighting, samples reflect the same sex and age profiles as the respective censuses.
b. Comparing Thai "due yah" with the combined Lao "due yah" and "lueng yah."
c. Completed illnesses experienced by respondent or child under their supervision, excluding incomplete episodes.

Reported purposes of antibiotic use were yet more varied and are displayed in Figure 2, Panel b. The most common use was the treatment of external wounds (Chiang Rai: 33.7%; Salavan: 44.4%; p<0.001). Other frequently reported uses in Salavan included coughs (30.5%; Chiang Rai: 10.9%; p<0.001) and fevers (30.5%; Chiang Rai: 8.3%; p<0.001). Thai respondents further indicated common use of antibiotics for sore throats (Chiang Rai: 36.3%; Salavan: 28.9%; p=0.016) and for the more general idea of an "inflammation" of the body (Chiang Rai: 23.5%; Salavan: 18.6%; p=0.083). Thai respondents would also more often limit their use to whatever a healthcare worker would recommend (Chiang Rai: 9.5%; Salavan: 5.4%; p=0.037), while 2.3% indicated that they would treat their plants or animals (dogs and chickens) with antibiotics (Salavan: 0.1%; p<0.001). Using antibiotics to treat infections or to fight bacteria and germs was only mentioned by a small minority of the rural populations (Chiang Rai: 2.4%; Salavan: 2.8%; p=0.243).

[Insert Figure 2 about here]

Figure 2. Common names and purposes for antibiotics.

Source: Authors' analysis of survey data.
Notes: Only including respondents who indicated that they had seen the presented medicine (i.e. common antibiotics) before. Chiang Rai: *n* = 1076; Salavan: *n* = 775. Population-weighted statistics, accounting for complex survey design. Multiple response permitted. Error bars indicate 95% confidence interval.

Table 2 indicates that—though people typically recognised the term "drug resistance"—the responses to the question "*What do you think is drug resistance?*" only rarely corresponded to clinical definitions, and the coexistence of two common translations of the term in Lao PDR complicated the picture

For peer review only

further. In Chiang Rai, 10.6% of the interpretations related to antibiotics and/or drug-resistant germs. Lao respondents linked the official term “*due yah*” to clinical definitions in 7.7% of all interpretations, and the colloquial term “*lueng yah*” in 9.6% of all interpretations. Not unlike other high- and low-income countries [26], drug resistance was typically interpreted as a growing tolerance of the body towards medicine as a result of repeated use (not limited to antibiotics). Other common interpretations in Chiang Rai were the incorrect or erratic use of medicine (12.5%), and an understanding of drug resistance as side-effects of or allergic reactions to medicine in general (4.2%). In Salavan, “*due yah*” was often interpreted as a refusal or “stubbornness” to take medicine (21.8%; possibly due to its literal translation into “*stubborn* [to the effect of] *medicine*”), while its vernacular equivalent “*lueng yah*” was often interpreted in the opposite way as a psychological dependence or addiction to medicine (24.9%).

Table 2. Awareness and interpretations of “drug resistance.”

	**Chiang Rai**		**Salavan**			
	“due yah”		***“due yah”***		***“lueng yah”***	
Awareness among rural population	72.9% (67.4–77.8)		27.1% (22.2–32.6)		58.8% (54.6–63.0)	
**Top 5 interpretations**						
Rank 1	Body becomes tolerant to medicine	54.1% (49.3–58.9)	Body becomes tolerant to medicine	38.1% (30.4–46.4)	Body becomes tolerant to medicine	50.9% (44.7–57.1)
Rank 2	Taking medicine incorrectly	12.5% (10.2–15.3)	Patient is “stubborn,” refuses medicine	21.8% (14.8–31.0)	Addicted to / preference for medicine	24.9% (20.2–30.2)
Rank 3	**Reference to antibiotics, drug-resistant germs**	**10.6% (8.1–13.8)**	Side-effects, drug allergy	9.2% (5.2–15.8)	**Reference to antibiotics, drug-resistant germs**	**9.6% (6.9–13.1)**
Rank 4	Don’t know	6.3% (4.6–8.7)	**Reference to antibiotics, drug-resistant germs**	**7.7% (4.7–12.5)**	Don’t know	4.0% (2.3–6.8)

For peer review only - http://bmjopen.bmj.com/site/about/guidelines.xhtml

Rank 5	Side-effects, drug allergy	4.2% (2.6–6.7)	Addicted to / preference for medicine	7.1% (3.5–13.8)	Sickness is "stubborn" / unresponsive	2.9% (1.3–6.2)

Source: Authors' analysis of survey data.
Notes: Ranking percentages only include respondents who indicated that they had heard the respective term "drug resistance" before. Chiang Rai: *n* = 871; Salavan (*due yah*): *n* = 206; Salavan (*lueng yah*): *n* = 470. Population-weighted statistics, accounting for complex survey design. Only single response permitted. In Salavan, the common response "*due yah* means *lueng yah*" (24.8% [18.4–32.6]) was recoded to incorporate respondent's definition of *lueng yah*.

Among our 2141 respondents, we captured 608 illness episodes in Chiang Rai and 356 in Salavan. Table 1 illustrates that healthcare utilisation during these episodes varied slightly across the two field sites. Chiang Rai respondents accessed a narrower spectrum of healthcare providers and were significantly less likely to access public and "other" healthcare providers (p<0.001 in both cases). Both sites also exhibited a high level of medicine access, with 2.2 and 2.5 medicine use episodes during an illness Chiang Rai and Salavan, respectively (p=0.050). Respondents in Chiang Rai thereby indicated higher use of non-antibiotic medicine (Chiang Rai: 1.6; Salavan: 1.3; p=0.048). In contrast, respondents in Salavan had more episodes of antibiotic use per illness (Chiang Rai: 0.2; Salavan: 0.4; p<0.001), and more usage of medicines that could potentially include antibiotics (Chiang Rai: 0.4; Salavan: 0.9; p<0.001). The pattern of antibiotic access was similar for informal sources, but generally lower in Chiang Rai: confirmed antibiotic use from informal channels represented 1.6% of all medicine use episodes in Chiang Rai and 3.3% in Salavan; and 3.6% in Chiang Rai and 7.9% in Salavan if unconfirmed but potential antibiotic use episodes are included.

Appendix Table A3 compares differences between individuals who accessed antibiotics from public, private, and informal sources. Contrary to intuition, patients receiving antibiotics from informal sources had no less wealth or formal education than users of public healthcare. Indeed, wealthier and more educated individuals in Chiang Rai were significantly associated with receiving antibiotics from informal sources (wealth: p=0.012; education: p=0.032). Similarly, awareness of drug resistance was not significantly lower among patients who received antibiotics from informal sources, while the share of respondents who linked drug resistance to biomedical notions of AMR in Salavan was significantly

For peer review only

higher among individuals accessing antibiotics through informal channels compared to public channels (13.4% vs. 4.4%, p=0.030). Patients who accessed antibiotics through informal channels were nevertheless significantly more inclined towards buying over-the-counter antibiotics than public antibiotic users (Chiang Rai: p=0.040; Salavan: p<0.001).

Figure 3 compares antibiotic use episodes from public, private, and informal healthcare providers, depending on the patient's attitude towards buying over-the-counter antibiotics (upper section: Chiang Rai; lower section: Salavan; dark-grey-shaded bars for confirmed antibiotics, light-grey-shaded bars for potential antibiotics). The figure demonstrates that the average number of antibiotic use episodes from informal sources was only marginally different for people with different attitudes (although statistically significant in Chiang Rai at p=0.030 for the subset of confirmed antibiotics). However, antibiotic use episodes from public healthcare providers were disproportionately *higher* among people who reported that they would not buy over-the-counter antibiotics, the difference of which was statistically significant in Salavan (p<0.001).[iv]

[Insert Figure 3 about here]

Figure 3. Antibiotic use episodes across field sites and channels of antibiotics access, by attitude towards buying over-the-counter antibiotics.

Source: Authors' analysis of survey data.
Notes: Including antibiotics and unclassified medicines that may be antibiotics. Illness-level data, including only completed illnesses experienced by respondent or a child under their supervision. Chiang Rai: n = 608; Salavan: n = 356. Population-weighted statistics, accounting for complex survey design. Multiple types of healthcare access per individual and illness episode possible. Group comparison using Wilcoxon rank-sum tests. Arrows illustrate differences and do not imply a causal relationship. OTC=over-the-counter.

[iv] Considering only confirmed antibiotic use episodes, the difference was still statistically significant at p=0·030.

Discussion and Conclusion

Our paper aimed at informing the understanding of LMIC contexts and AMR-related general population behaviour through a study of antibiotic-related knowledge, attitudes, and practices in rural Thailand and Lao PDR. We demonstrated that rural populations exhibited:

- mixed but surprisingly high levels of awareness and attitudes corresponding to AMR awareness-raising material, although only a minority of villagers were familiar with technical notions of antibiotics and drug resistance;
- relatively low levels of antibiotic access from informal sources; and
- surprisingly counter-intuitive links between informal antibiotic use, people's socio-economic status, and their attitudes—especially among villagers in Salavan, who had disproportionately high antibiotic use if their attitude showed a disinclination against over-the-counter antibiotics.

Our survey data also revealed profound differences between the two field sites despite their cultural and geographical proximity. For example, villagers referred to antibiotics with wide-ranging and locally specific vernacular expressions (only a minority adopted technical language in either site), and "drug resistance" was typically understood as a general tolerance of the body to medicine but local interpretations ranged from patients refusing medicine to patients being addicted to medicine.

The surveys were implemented after the Monsoon season to reduce accessibility barriers like landslides, floods, and farm work. This temporal focus meant that our survey was not able to capture internal migration or seasonal change affecting the epidemiological environment. The rural survey is also unable to speak for urban health behaviour or behavioural patterns outside rural Thailand and Lao PDR, or for awareness and behaviour among healthcare staff and policy makers (with which awareness-raising activities for the general public may interact). Lastly, our focus on health behaviour and our 60-day recall period could introduce recall and social desirability biases. Most LMIC health behaviour research uses 14-30-day recall periods; longer recall periods can lead to underrepresentation

For peer review only

of lower educated groups [27]. However, for a survey of behaviour rather than of epidemiological patterns, 14-day recall would have truncated the sample to an impractical size (omitting 540/964 [56.0%] of all responses) and neglected that illness episodes often extended beyond a fortnight (as was the case for 91/964 [8.7%] of the recorded illnesses). In response, we conducted regular review sessions with our survey team to identify and alleviate social desirability; we excluded chronic illnesses; and our questionnaire asked our respondents to walk through the sequence of events, which improves recall [28]. While we cannot rule out a residual risk of social desirability and recall bias, it is not clear *a priori* whether and how any remaining bias would affect our comparison of antibiotic uses across different healthcare providers.

By virtue of being a representative rural survey in northern Thailand and southern Lao PDR, the specific notions and behavioural patterns around antibiotic use are not generalisable beyond the study context, even though similar interpretations of antibiotics as “anti-inflammatory medicine” exist elsewhere (e.g. in China) [29]. However, the findings of our study have a broader relevance insofar as they expose the complexity of local knowledge and its relationship to AMR-related behaviour. On the one hand, our work underlines the challenges facing public awareness campaigns as the current principal strategy to change AMR-related population behaviour. For example, if not mindful of the local context, the slogan of the 2017 World Antibiotic Awareness Week to “use antibiotics wisely to combat rising drug resistance” could plausibly entail *increased* antibiotic use or the use of stronger medicine if people understand drug resistance as stubbornness of patients or as a problem applying to all types of medicines.

On the other hand, our study also demonstrated that the link between knowledge, attitudes, and antibiotic-related behaviour may be weak in LMIC contexts. This disjunction is not new [5], but the counter-intuitive link between education, antibiotic-related attitudes, and antibiotic use from informal sources suggests that AMR-related information can easily entail unintended consequences—knowledge and awareness empower, but people themselves decide how they will use this new “power”

For peer review only

in their daily lives [30]. For instance, villagers may not necessarily buy antibiotics from unregulated corner shops because of ignorance, but because they become more assertive about their health.

Considering potential misunderstandings in AMR communication on the one hand and contextual determinants of behaviour beyond knowledge deficits on the other, we call for an expansion of behavioural AMR strategies to address structural factors of behavioural change. For example, vulnerability and adversity may drive people into seemingly irrational antimicrobial use [31]. A sick labourer or factory worker may take antimicrobials desperately to maintain their job and to sustain their families, in which case it would be futile trying to convince them that their hardship is secondary to the global health goal of tackling AMR. Yet, it may be possible to alleviate their pressure to consume antimicrobials through paid sick leave and unemployment insurance. We propose the exploration of such "AMR-sensitive interventions" to address upstream drivers of antimicrobial use and to complement education and awareness campaigns—similar to nutrition-sensitive interventions that target the determinants of mal- and undernutrition through upstream interventions like social safety nets (rather than e.g. providing supplements directly to people) [32]. AMR-sensitive interventions require us to venture out of health policy terrain into broader development policy. There is yet little evidence whether and how such context-oriented approaches bear fruit. Greater involvement of the social sciences is necessary to uncover this gap and to find constructive solutions that address the social factors of which AMR is a symptom.

ACKNOWLEDGEMENTS

We thank our collaborators Caroline OH Jones, Romyen Kosaikanont, Pollavat Praphattong, Pathompong Manohan, Paul N Newton, Sommay Keomany, Penporn Warapikuptanun, Daranee Intralawan, Patchapoom U-Thong, Patipat Benjaroon, Narinnira Sangkham, Sirirat Chailert, Krittanon Promsutt, Maipheth Keovilayvanh, and Phaengnitta Phanthasomchit for guidance during the study design and for field survey implementation assistance. We further thank Ernest R Guevarra and Eric

For peer review only

Ohuma for helpful discussions, feedback, and support during the survey design phase; Edward Gibbs, Lisa White, the Centre for Tropical Medicine and Global Health, the CABDyN Complexity Centre, and the Mahidol-Oxford Tropical Medicine Research Unit / Wellcome Trust Strategic Award for institutional support; Zoë Doran and the Clinical Trials Support Group at the Mahidol Oxford Tropical Medicine Research Unit for quality assurance during the protocol development process; Alison Brindle, Claire-Lise Kessler, and John Bleho for engaging communication work; and Karen Valentine, Holly Blades, and Kanchana Pongsaswat for indispensable operational and logistical assistance.

AUTHOR CONTRIBUTIONS

MJH conceptualised the study, its design, and its theoretical framing. All authors provided inputs into the study design. MJH, GZ, and NC developed the survey instruments. MJH and NC developed the study protocol and cleaned and coded the data. MJH analysed the data and drafted the manuscript. All authors reviewed and approved the manuscript.

COMPETING INTERESTS

We declare that no conflict of interest – financial or otherwise – exists.

DATA SHARING

The data set will be made publicly available in January 2019 on the UK Data Service and equivalent repositories in Thailand and Lao PDR.

ETHICS APPROVAL

The research was reviewed and approved by the University of Oxford Tropical Research Ethics Committee (Ref. OxTREC 528-17), and it received local ethical approval in Thailand from the Mae Fah Luang University Research Ethics Committee on Human Research (Ref. REH 60099), and in Lao

For peer review only

PDR from the National Ethics Committee for Health Research (Ref. NEHCR 074). Participation in the and educational activity was voluntary and we obtained informed verbal consent from all participants, which was audio recorded and documented by the survey field investigators with a written record of oral consent for each participant.

FUNDING SOURCES

This project is funded by the Antimicrobial Resistance Cross Council Initiative supported by the seven research councils in partnership with the Department of Health and Department for Environment Food & Rural Affairs (grant ref. ES/P00511X/1, administered by the UK Economic and Social Research Council). YKZ was further supported by internal research placement funding by the MSc International Health and Tropical Medicine (University of Oxford). The funders had no involvement in the design and implementation of the project.

REFERENCES

1. FAO, OIE, WHO. Handle antimicrobials with care: Tripartite brochure [infographic]. Geneva: World Health Organization, 2017.
2. The Review on Antimicrobial Resistance. Tackling drug-resistant infections globally: final report and recommendations. London: The UK Prime Minister, 2016.
3. WHO. Global action plan on antimicrobial resistance. Geneva: World Health Organization, 2015.
4. MacDougall C, Polk RE. Antimicrobial stewardship programs in health care systems. Clinical Microbiology Reviews 2005;**18**(4):638-56 doi: 10.1128/CMR.18.4.638-656.2005.
5. Chandler CIR, Hutchinson E, Hutchison C. Addressing antimicrobial resistance through social theory: an anthropologically oriented report. London: London School of Hygiene & Tropical Medicine, 2016.

For peer review only

6. Cawley J, Ruhm CJ. The economics of risky health behaviors. In: Pauly MV, Mcguire TG, Barros PP, eds. Handbook of health economics. Amsterdam: Elsevier, 2011:95-199.

7. Sheehy-Skeffington J, Rea J. How poverty affects people's decision-making processes. York: Joseph Rowntree Foundation, 2017.

8. Davis M, Whittaker A, Lindgren M, et al. Understanding media publics and the antimicrobial resistance crisis. Global Public Health 2017;**13**(9):1158-68 doi: 10.1080/17441692.2017.1336248.

9. Fynbo L, Jensen CS. Antimicrobial stigmatization: public health concerns about conventional pig farming and pig farmers' experiences with stigmatization. Social science & medicine 2018;**201**:1-8 doi: 10.1016/j.socscimed.2018.01.036.

10. Elsevier B.V. Scopus. Secondary Scopus 2018. http://www.scopus.com/.

11. Research Councils UK. Gateway to research. Secondary Gateway to research 2018. http://gtr.rcuk.ac.uk/.

12. Kinh NV, Wertheim HFL, Thwaites GE, et al. Developing an antimicrobial resistance reference laboratory and surveillance programme in Vietnam. The Lancet Global Health 2017;**5**(12):e1186-e87 doi: 10.1016/S2214-109X(17)30370-4.

13. GARDP. Business plan 2017-2023: developing new antibiotic treatments and ensuring sustainable access for all. Geneva: Global Antibiotic Research and Development Partnership, 2017.

14. Paton J, Kresge N. Superbugs win another round as Big Pharma leaves antibiotics. Secondary Superbugs win another round as Big Pharma leaves antibiotics 2018. https://www.bloomberg.com/news/articles/2018-07-13/superbugs-win-another-round-as-big-pharma-leaves-antibiotics.

15. The Lancet. UK life science research: time to burst the biomedical bubble. The Lancet 2018;**392**(10143):187 doi: 10.1016/S0140-6736(18)31609-X.

For peer review only

16. Haenssgen MJ, Charoenboon N, Zanello G, et al. Antibiotics and activity spaces: protocol of an exploratory study of behaviour, marginalisation, and knowledge diffusion. BMJ Global Health 2018;**3**(e000621) doi: 10.1136/bmjgh-2017-000621.

17. Holloway KA, Batmanabane G, Puri M, et al. Antibiotic use in South East Asia and policies to promote appropriate use: reports from country situational analyses. Bmj 2017;**358**(Suppl1) doi: 10.1136/bmj.j2291.

18. Chereau F, Opatowski L, Tourdjman M, et al. Risk assessment for antibiotic resistance in South East Asia. Bmj 2017;**358**(Suppl1) doi: 10.1136/bmj.j3393.

19. Public Health England. UK case of Neisseria gonorrhoeae with high-level resistance to azithromycin and resistance to ceftriaxone acquired abroad. Health Protection Report Advanced Access Report 2018;**12**(11).

20. Klein EY, Van Boeckel TP, Martinez EM, et al. Global increase and geographic convergence in antibiotic consumption between 2000 and 2015 [epub ahead of print]. Proceedings of the National Academy of Sciences 2018 doi: 10.1073/pnas.1717295115.

21. Haenssgen MJ. Satellite-aided survey sampling and implementation in low- and middle-income contexts: a low-cost/low-tech alternative. Emerging Themes in Epidemiology 2015;**12**(20) doi: 10.1186/s12982-015-0041-8.

22. Wikimedia Commons. Locator map of Chiang Rai Province, Thailand, by NordNordWest; a map of Laos with Salavan province highlighted, by Infernoapple. Secondary Locator map of Chiang Rai Province, Thailand, by NordNordWest; a map of Laos with Salavan province highlighted, by Infernoapple 2018. Wikimedia Commons Web pages: http://en.wikipedia.org/wiki/File:Gansu_in_China_(%2Ball_claims_hatched).svg and https://commons.wikimedia.org/wiki/File:Rajasthan_in_India_(claimed_and_disputed_hatched).svg?uselang=en.

For peer review only

23. Willis GB. The practice of cross-cultural cognitive interviewing. Public Opinion Quarterly 2015;**79**(S1):359-95 doi: 10.1093/poq/nfu092.

24. Haenssgen MJ, Charoenboon N, Althaus T, et al. The social role of C-reactive protein point-of-care testing to guide antibiotic prescription in Northern Thailand. Social science & medicine 2018;**202**:1-12 doi: 10.1016/j.socscimed.2018.02.018.

25. Khine Zaw Y, Charoenboon N, Haenssgen MJ, et al. A comparison of patients' local conceptions of illness and medicines in the context of C-reactive protein biomarker testing in Chiang Rai and Yangon. American Journal of Tropical Medicine and Hygiene 2018;**98**(6):1661-760 doi: 10.4269/ajtmh.17-0906.

26. McCullough AR, Parekh S, Rathbone J, et al. A systematic review of the public's knowledge and beliefs about antibiotic resistance. Journal of Antimicrobial Chemotherapy 2016;**71**(1):27-33 doi: 10.1093/jac/dkv310.

27. Das J, Hammer J, Sánchez-Paramo C. The impact of recall periods on reported morbidity and health seeking behavior. Journal of Development Economics 2012;**98**(1):76-88 doi: http://dx.doi.org/10.1016/j.jdeveco.2011.07.001.

28. Fowler FJ. *Improving survey questions: design and evaluation*. Thousand Oaks, CA: Sage, 1995.

29. Yu M, Zhao G, Stålsby Lundborg C, et al. Knowledge, attitudes, and practices of parents in rural China on the use of antibiotics in children: a cross-sectional study. BMC Infectious Diseases 2014;**14**(1):112 doi: 10.1186/1471-2334-14-112.

30. Carrion ML. "You need to do your research": vaccines, contestable science, and maternal epistemology. Public Understanding of Science 2018;**27**(3):310-24 doi: 10.1177/0963662517728024.

31. Ribera JM, Hausmann-Muela S. The straw that breaks the camel's back: redirecting health-seeking behavior studies on malaria and vulnerability. Medical Anthropology Quarterly 2011;**25**(1):103-21 doi: 10.1111/j.1548-1387.2010.01139.x.

For peer review only

32. Ruel MT, Alderman H, The Maternal and Child Nutrition Study Group. Nutrition-sensitive interventions and programmes: How can they help to accelerate progress in improving maternal and child nutrition? The Lancet 2013;**382**(9891):536-51 doi: 10.1016/S0140-6736(13)60843-0.

For peer review only

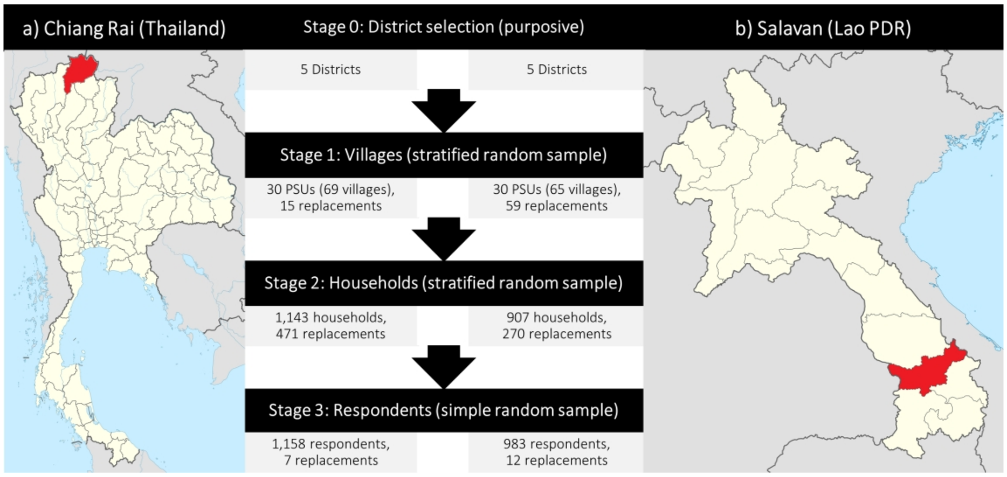

Survey sites and multi-stage sampling process. Source: Authors, adapted from Wikimedia Commons [22]. Notes: Unavailable selections at each sampling stage were substituted with a random replacement for the random samples of PSUs and household members, and with the nearest available neighbour for the interval sample of households. One PSU could contain more than one administrative village; if the first-chosen village contained less than 600 houses, then adjacent villages would be included. PSU=Primary Sampling Unit.

257x120mm (300 x 300 DPI)

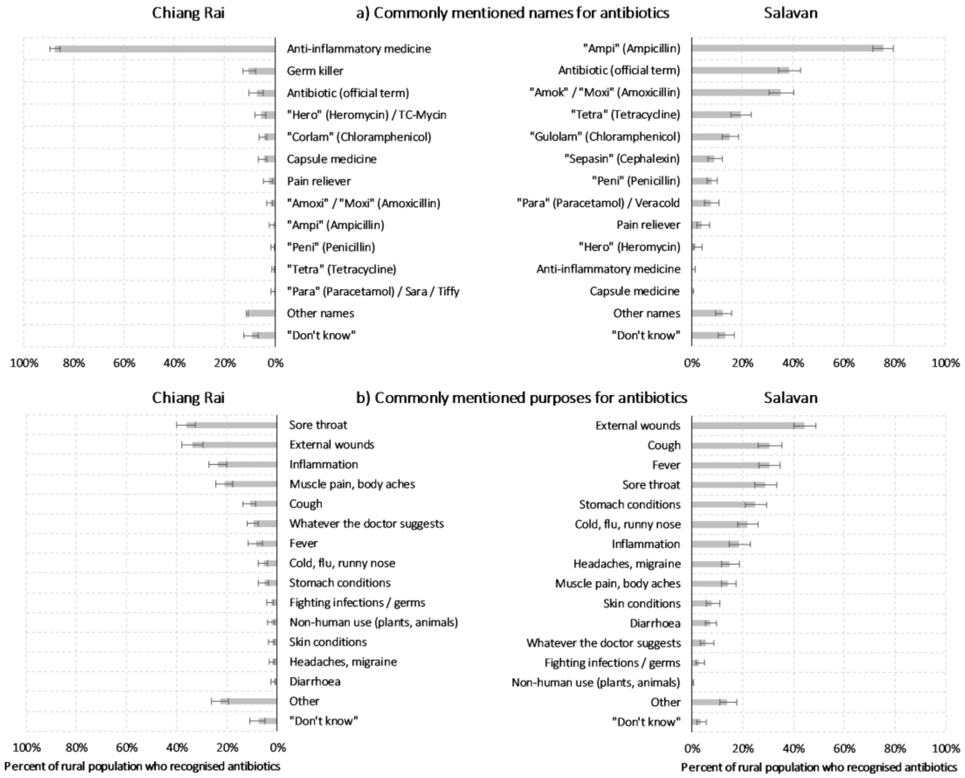

Common names and purposes for antibiotics. Source: Authors' analysis of survey data. Notes: Only including respondents who indicated that they had seen the presented medicine (i.e. common antibiotics) before. Chiang Rai: n = 1076; Salavan: n = 775. Population-weighted statistics, accounting for complex survey design. Multiple response permitted. Error bars indicate 95% confidence interval.

249x193mm (300 x 300 DPI)

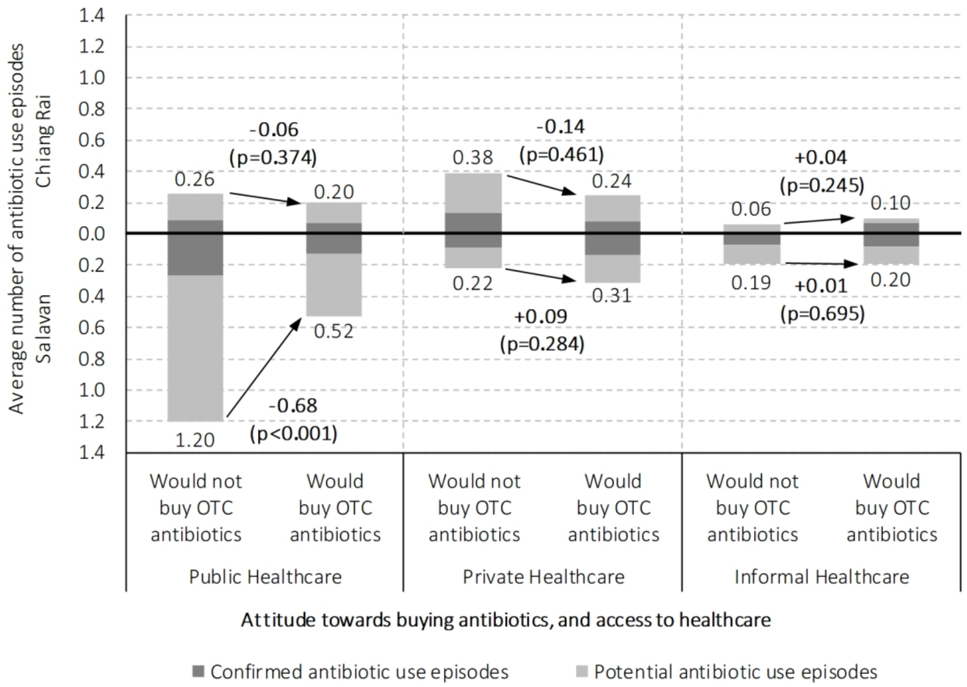

Antibiotic use episodes across field sites and channels of antibiotics access, by attitude towards buying over-the-counter antibiotics. Source: Authors' analysis of survey data. Notes: Including antibiotics and unclassified medicines that may be antibiotics. Illness-level data, including only completed illnesses experienced by respondent or a child under their supervision. Chiang Rai: n = 608; Salavan: n = 356. Population-weighted statistics, accounting for complex survey design. Multiple types of healthcare access per individual and illness episode possible. Group comparison using Wilcoxon rank-sum tests. Arrows illustrate differences and do not imply a causal relationship. OTC=over-the-counter.

139x100mm (300 x 300 DPI)

Appendix Figure A1. Trend of AMR-related social sciences and multidisciplinary publications, 1970–2017.

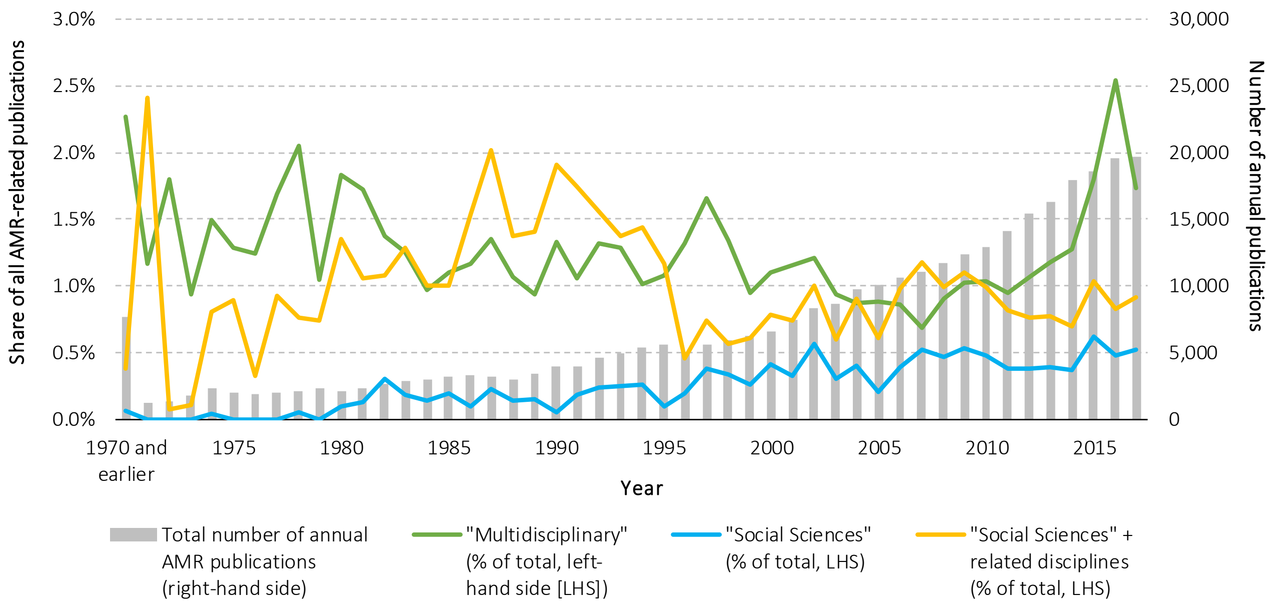

Source: Authors, based on Elsevier B.V. [15].

Notes: Data until 2018, as of 9 October 2018. Disciplines as reported by Scopus database, with "'Social Sciences' + related disciplines" providing upper bound of social science publications including Arts and Humanities;" "Business, Management and Accounting;" "Decision Sciences;" "Economics, Econometrics and Finance;" and "Psychology" alongside "Social Sciences." Based on search query [TITLE-ABS-KEY ("antibiotic resistance" OR "drug resistance" OR "antimicrobial resistance" OR "AMR")]. Total number of publications as of 9 October 2018 was 347,511, of which 66.0% arose from "Medicine;" 28.3% from "Biochemistry, Genetics and Molecular Biology;" and 18.2% from "Immunology and Microbiology" (multiple categories per publication possible.) "Social Sciences" average during the period was 0.4% (0.9% for "'Social Sciences' + related disciplines"). LHS=left-hand side.

Appendix Table A2. Variable descriptions.

	Variable	Description
Demographic attributes	Female	Binary variable: Sex of respondent (R); [1] if female.
	Age	Continuous variable: Age in years.
	Education	Continuous variable: Completed years of formal education.
	Speaking Thai / Lao	Binary variable: [1] if R reported ability to communicate in main language (irrespective of reading and writing).
	Wealth index	Continuous variable: Average of 17 household assets and amenities on scale from [0] to [1].
	Buddhist religion	Binary variable: [1] if R belongs to the majority religion (Buddhism in both sites).
	Thai/Lao nationality	Binary variable: [1] if R has Thai (Chiang Rai) or Lao (Salavan) nationality.
	Majority ethnic group	Binary variable: [1] if R belongs to the majority ethnic group Thai (Chiang Rai) or Lao Loum (Salavan).
Antibiotic knowledge / attitudes	Aware of antibiotics	Binary variable: [1] if R recognised images of antibiotic capsules that are common in the field site and, if not, the most common translation of antibiotics as "anti-inflammatory drug" ("ยาแก้อักเสบ" or "*yah kae ak seb*") in Thai and "germ resister" ("ຢາຕ້ານເຊື້ອ" or "*yah dtan suea*") in Lao. Additional categorical variables (coded ex ante and ex post) recorded the names and purposes that the respondent reported following recognition of the medicine.
	Aware of drug resistance	Binary variable: [1] if R recognised the local terms for "drug resistance." In Thai, "drug resistance" was translated as "ดื้อยา" ("*due yah*"). Lao has two translations of which "ດື້ຢາ" ("*due yah*") is the formal term and "ລື້ງ ຢາ" ("*lueng yah*") is a more colloquial but broader expression (both translations were asked separately). Additional categorical variables (coded ex ante and ex post) recorded the interpretations of each term.
	Would not buy antibiotics over the counter	Binary variable: [1] if answer to question "Is there any situation for which you would buy this medicine?" corresponded to FAO/OIE/WHO message "When using antibiotics: follow professional advice" (field coded based on survey training manual) [1].
	Prefers alternatives over antibiotics	Binary variable: [1] if answer to question "Do you prefer other remedies such as herbs or cough syrup to this medicine for sore throat?" corresponded to FAO/OIE/WHO message "Ensure medicines are only used when necessary" (field coded) [1].
	Does not keep antibiotics for future use	Binary variable: [1] if answer to question "If you were prescribed this medicine by a doctor and did not finish the course, would you keep it for future use?" corresponded to FAO/OIE/WHO message "When using antibiotics: never share medicines or use leftover drugs to treat a different illness" (field coded) [1].
	Knows that antibiotic resistance can spread	Binary variable: [1] if answer to question "Can your '*due yah*' (drug resistance) spread to other people, for example if you sneeze on them?" corresponded to FAO/OIE/WHO message "antimicrobial resistance can affect us all" (field coded) [1].
	Answer score	Continuous variable: Number of preceding answers ([0] to [4]) corresponding to FAO/OIE/WHO material [1].
Illness episodes	Self-rated severity	Ordinal variable: [1] if illness is reported as "mild;" [2] as "moderate;" [3] as "severe."
	Duration of illness episode	Continuous variable: Total duration of illness episode in days, calculated as sum of duration of individual steps in episode.
Treatment-seeking behaviour	Public healthcare provider	Binary variable: [1] if R reported accessing health centre or hospital during illness episode.
	Private healthcare provider	Binary variable: [1] if R reported accessing private clinic, hospital, or pharmacy.
	Informal healthcare provider	Binary variable: [1] if R reported accessing grocery store or traditional healer.
	Care from family or self-care	Binary variable: [1] if R reported self-treatment or care from family member or friend.
	Other types of healthcare access	Binary variable: [1] if R reported accessing other healthcare provider (e.g. village health volunteer).
	Medicines use episodes	Continuous variable: Number of reported medicine use episodes per illness, categorised into types of medicine (coded ex post into non-antibiotic medicine, antibiotics, and potential antibiotics) and sources of access (public, private, informal healthcare provider). Note that the actual amount of medicine used during each "medicine use episode" is likely to vary systematically across formal and informal healthcare providers, with "episodes" from the latter typically containing only a small number of pills and capsules for immediate treatment of symptoms.

Source: Authors.

For peer review only

Appendix Table A3. Characteristics of individuals who received antibiotics from public, private, and informal sources.

	Chiang Rai			p values[c]			Salavan			p values[c]		
	**Public antibiotic access**	**Private antibiotic access**	**Informal antibiotic access**	**Publ. vs. priv.**	**Publ. vs. inf.**	**Priv. vs. inf.**	**Public antibiotic access**	**Private antibiotic access**	**Informal antibiotic access**	**Publ. vs. priv.**	**Publ. vs. inf.**	**Priv. vs. inf.**
Number	93	115	35	200	110	132	157	38	41	179	178	77
**Demographics**												
Female	53.8% (40.3–66.8)	59.6% (46.6–71.4)	56.9% (34.2–77.1)	0.462	0.738	0.832	71.5% (63.8–78.2)	41.0% (23.5–61.1)	71.8% (51.2–86.1)	0.003	0.790	0.017
Age	43 (13)	48 (14)	40 (11)	0.158	0.584	>0.001	35 (19)	33 (12)	39 (18)	0.674	0.338	0.349
Education (years)	6.2 (4.9)	5.8 (5.2)	8.0 (3.8)	0.766	0.238	0.032	4.7 (6.3)	8.1 (5.9)	5.9 (5.5)	>0.001	0.419	0.139
Speaking Thai / Lao	92.3% (81.9–96.9)	85% (73.7–92)	98% (86.1–99.7)	0.157	0.227	0.036	92.7% (89.3–95.1)	100.0% (100.0–100.0)	100.0% (100.0–100.0)	0.007	0.029	..
Wealth index	0.6 (0.1)	0.7 (0.1)	0.7 (0.1)	0.015	>0.001	0.012	0.4 (0.2)	0.5 (0.1)	0.5 (0.2)	>0.001	0.389	0.205
Buddhist religion	78.5% (64.8–87.8)	77.2% (63.9–86.6)	89.0% (74.4–95.8)	0.871	0.236	0.149	66.5% (58.3–73.8)	78.9% (52.9–92.5)	79.2% (58.9–91.0)	0.310	0.110	0.976
Thai/Lao nationality	97.1% (89.8–99.2)	95.9% (88.8–98.5)	98% (86.1–99.7)	0.710	0.865	0.597	99% (92.9–99.9)	100.0% (100.0–100.0)	97.6% (84.0–99.7)	0.539	0.418	>0.001
Majority ethnic group (Thai/Lao Loum)	58.8% (45.3–71.1)	64.6% (52.3–75.3)	81.9% (65.8–91.4)	0.439	0.009	0.098	58.6% (48.9–67.7)	69.9% (45.6–86.6)	68.2% (44.4–85.2)	0.401	0.417	0.865
**Antibiotic knowledge / attitudes**												
Aware of antibiotics	97.5% (90.4–99.4)	95.7% (88.6–98.4)	93.3% (64.5–99.1)	0.519	0.107	0.629	88.7% (82.3–92.9)	92.7% (72.2–98.4)	100.0% (100.0–100.0)	0.276	0.022	0.142
Aware of drug resistance[a]	75.0% (62.5–84.3)	76.5% (64.9–85.1)	90.2% (76.4–96.4)	0.807	0.074	0.062	66.3% (57.8–73.8)	84.7% (61.1–95.1)	79.5% (61.3–90.5)	0.091	0.141	0.676
Links drug resistance to AMR concepts[b]	1.7% (0.3–9.5)	5.4% (2.0–13.6)	1.3% (0.2–9.0)	0.241	0.916	0.027	4.4% (2.0–9.3)	9.0% (1.9–33.5)	13.4% (5.6–28.7)	0.349	0.030	0.608
Would not buy antibiotics over the counter	62.0% (48.2–74.1)	62.1% (48.3–74.2)	42.0% (22.0–65.0)	0.963	0.046	0.040	44.5% (35.9–53.5)	18.3% (9.3–32.7)	23.3% (12.3–39.6)	>0.001	>0.001	0.441
Prefers antibiotics over alternatives	62.7% (49.6–74.2)	50.2% (36.8–63.6)	70.3% (46.2–86.7)	0.119	0.286	0.078	22.8% (16.8–30.2)	33.7% (19.5–51.7)	29.3% (13.7–52.0)	0.117	0.546	0.557
Does not keep antibiotics for future use	54.3% (41.2–66.8)	53.9% (41.7–65.6)	38.1% (18.9–62.0)	0.972	0.353	0.072	21.1% (14.2–30.2)	14.0% (4.6–35.4)	13.9% (5.2–32.1)	0.474	0.154	0.996
Knows that antibiotic resistance can spread	8.1% (3.9–16.2)	10.9% (5.7–19.9)	6.5% (1.6–23.2)	0.478	0.620	0.470	1.9% (0.5–6.6)	1.8% (0.2–12.4)	0.0% (0.0–0.0)	0.986	0.172	0.341
Answer score (0 to 4)	1.9 (0.9)	1.8 (0.9)	1.6 (0.8)	0.589	0.086	0.294	0.9 (1.1)	0.7 (0.7)	0.7 (0.6)	0.064	0.061	0.964

Source: Authors' analysis of survey data.

Notes: Including antibiotics and unconfirmed medicines that may include antibiotics. Illness-level data, including only completed illnesses experienced by respondent or a child under their supervision. Population-weighted statistics, accounting for complex survey design. Multiple types of healthcare access per individual and illness episode possible.

a. Comparing Thai "due yah" with the combined Lao "due yah" and "lueng yah."

b. Corresponding to interpretation of "drug resistance" as "Reference to antibiotics, drug-resistant germs" in Exhibit 6.

c. X2 and Wilcoxon rank-sum tests, omitting simultaneous antibiotic access from more than one type of healthcare provider, which was the case in 82/958 [8.6%] of all pairwise comparisons of antibiotic access.

For peer review only

1. Village Checklist (GPS coordinates of village and facilities) (to be completed by supervisor)

What kind of facility would you like to record?

A. District Number		[*code entered automatically*]
B. Village Number		[*code entered automatically*]
C. Village centre	a) Latitude	[*coordinates entered automatically*]
	b) Longitude	[*coordinates entered automatically*]
D. Village head's house	a) Latitude	[*coordinates entered automatically*]
	b) Longitude	[*coordinates entered automatically*]
E. Local shop	a) Latitude	[*coordinates entered automatically*]
	b) Longitude	[*coordinates entered automatically*]
F. Market	a) Latitude	[*coordinates entered automatically*]
	b) Longitude	[*coordinates entered automatically*]
G. Temple	a) Latitude	[*coordinates entered automatically*]
	b) Longitude	[*coordinates entered automatically*]
H. School	a) Latitude	[*coordinates entered automatically*]
	b) Longitude	[*coordinates entered automatically*]
I. Bus stop	a) Latitude	[*coordinates entered automatically*]
	b) Longitude	[*coordinates entered automatically*]
J. Health facility Specify (public, private, pharmacy, local store, traditional healer, etc.): ___________________	a) Latitude	[*coordinates entered automatically*]
	b) Longitude	[*coordinates entered automatically*]
	c) Who is staffing the facility?	Total staff: ___<br>Staff at time of visit: _________
	d) Does the provider have antibiotics available?	Yes ....1<br>No 0

For peer review only

Interview data [*Record observation*]

i. District Number		[*code entered automatically*]
ii. PSU Number		[*code entered automatically*]
iii. Household number		Number: ______
iv. Household coordinates	a) Latitude	[*coordinates entered automatically*]
	b) Longitude	[*coordinates entered automatically*]

v. What type is this house most similar to?

1...
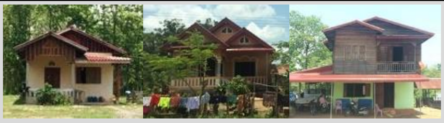

2...
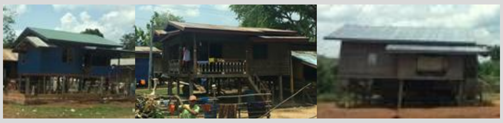

3...
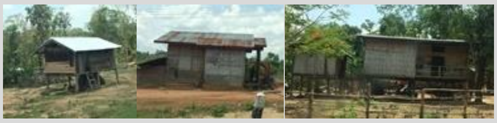

vi. Time of visit	a) First visit	[*time entered automatically*]
	b) Second visit	[*time entered automatically*]

List all persons aged 18+ years in household

Hello, I'm a researcher working for the Mahidol-Oxford Tropical Medicine Research Unit. We are interested in the lives and health behaviours of villagers across Thailand and Lao PDR. We are selecting participants randomly and would like to choose one or two members of your household. In order to choose and ask them to participate, could you please tell us who lives here? [provide PIS on request]

[*1 respondent per every 5 household members will be selected randomly from this list*]

Name	Nickname	Sex (M / F)	Age	Available for interview today? (Yes / No)

Statement of consent (Respondent will receive participant information sheet and verbal consent will be taken)
Thank you for participating. You will receive a small token of gratitude for your participation at the end of the interview.

vii.Date of interview	[*date entered automatically*]
viii.Time of interview begin	[*time entered automatically*]
ix.Respondent name	Respondent name: ______________________________
x.Interviewer code	[*code entered automatically*]

Part I: Personal and Household Characteristics
Let us begin with a few questions about yourself and your household.

1. [*record as observed*] Sex		Female........................ 1<br>Male 0
2. How old are you? [*in years*] [*If respondent cannot give exact age, ask for approximate age and code in range: 18-24, 25-34, 35-44, 45-59, 60 and older*]		Age in years: _______
3. Please indicate what kind of work you do. If you have more than one occupation at one time or throughout the year, please begin with the one in which you spend the most time and name up to three. If you do not have an occupation, please also mention whether you are still a student, retired, or unemployed.	a) Main occupation	Occupation: _________
	b) Side occupation	Occupation: _________
	c) Side occupation	Occupation: _________
4. What is your mother tongue?		Mother tongue: ________________
5. [In Thailand:] Can you speak Thai? [In Laos:] Can you speak Lao?		Yes .... 1<br>No 0
6. What is the highest grade of schooling that you completed?<br>[*excluding informal education and pre-school education such as nursery and kindergarten, but including grade school, high school, vocational training, tertiary education, etc.*]		Highest grade: ___
7. Are you the head of your household?		Yes ....1<br>No 0
7.1. [*if no*] What is the name of your household head?		Name: __________

For peer review only

8. What is your current marital status?	Never married ... 1<br>Currently married ... 2<br>Cohabiting ... 3<br>Separated / divorced ... 4<br>Widowed ... 5	
9. Are there any close family members of yours [children, spouse, siblings, parents] who live elsewhere? [*select "no" if not applicable*]	9.1. Do your parents live outside of this village? [*do not count parents-in-law*]	At least 1 person outside village .... 1<br>All inside village / not applicable.... 0
	9.2. Does your spouse live outside of this village?	At least 1 person outside village .... 1<br>All inside village / not applicable.... 0
	9.3. Do you have siblings who live outside of this village? [*do not count brothers-in-law and sisters-in-law*]	At least 1 person outside village .... 1<br>All inside village / not applicable.... 0
	9.4. Do you have children who live outside of this village?	At least 1 person outside village .... 1<br>All inside village / not applicable.... 0
Part II: Social Networks [for network census villages only]<br>I will now ask you some questions about your interactions with other people within and outside of your village.		
10. [Round I of network survey only] Where do you spend most of your time interacting with other people from your village?	a) Field: ___<br>b) Temple: ___<br>c) Local store: ___<br>d) Market: ___<br>e) Children's schools: ___<br>f) Home: _____<br>g) Workplace: ___<br>h) Village event/s: _______<br>i) Other site: ___	

For peer review only

11. [Round I of network survey only] Outside your household, with whom do you interact on a regular basis? (May be anyone from both inside and outside of the village, and through any platform which might not require a face-to-face interaction)

	a) What is the nickname of the person?	b) How is this person related to you? [*give examples if respondent is unsure about answer categories*]	c) What is the sex of this person?	d) Where does this person live?	e) What is the name of the household head of this person?	f) How often do you interact with this person?	g) How do you interact with this person? [*Mark all that apply*]	h) Do your conversations relate to health and well-being?
**11.1.** Contact 1	Nickname _____<br>Name _____	Spouse........ 1<br>Parent........ 2<br>Child........ 3<br>Sibling........ 4<br>Other relative........ 5<br>Neighbour........ 6<br>Friend (if not neighbour)........ 7<br>Other villager........ 8<br>Other (specify) _........ 9	Female...1<br>Male 0	In village .... 1<br>(specify: _____)<br>Outside village .. 2	Name of household head<br>________	Daily or more often ....4<br>Weekly or few times/week ....3<br>Monthly or few times/month ...2<br>Yearly or few times/year ....1<br>Less often or never 0	Face-to-face...1<br>Voice call........2<br>Messenger .....3<br>Other (specify)<br>_____ 4	Yes .... 1<br>No 0
**11.2.** Contact n	Nickname<br>Name	1 2 3 4 5 6 7 8 9	1 0	1 2	Name	0 1 2 3 4	1 2 3 4	1 0

**11a. [Round II of network survey only]** When we last visited you, you told us that you interact regularly with [*names*]. Has anything changed since last time?	Yes........1 → **[update social network question 11]**<br>No........0
**11i. [Round I of network survey only]** Is there anybody in your household with whom you talk about health and well-being? [*Mark all that apply*]	[*mark all names from household roster that apply*]

For peer review only

[*For network survey village respondents in Round 2*]<br>12. An education activity has recently taken place in your village.	
12.1. Did you participate in any of the activities?	Yes .... 1<br>Yes, but not throughout .... 2<br>No .... 3<br>Don't know / prefer not to say 4
12.2. Did you talk with anybody about the activity in your village?<br>[*"Talking" can involve any conversation including asking for information, informing about the educational activity, or discussing it (regardless of actual attendance)*]	a) Nickname 1: ______ b) Full name 1: ______ c) Relationship 1: 1 2 3 4 5 6 7<br>a) Nickname n: ______ b) Full name n: ______ c) Relationship n: 1 2 3 4 5 6 7<br>[*Relationship codes*]<br>Household member .... 1<br>Family member outside HH .... 2<br>Other relative .... 3<br>Neighbour .... 4<br>Friend other than neighbour .... 5<br>Other villager .... 6<br>Other (specify) _ 7
[*If respondent indicates conversation in Q 12.2*]<br>12.3. What subjects did you talk about in respect to the activity?<br>[*mark all that apply*]	Going to doctor when sick .... 1<br>Anti-inflammatories/antibiotics .... 2<br>Germs .... 3<br>Using medicines correctly .... 4<br>Activity in general .... 5<br>Games/awards .... 6<br>Song/Story/Play .... 7<br>Money/compensation .... 8<br>Other (specify) ____ 9

Part III: Healthcare Seeking Thank you for this. Now we come to a part where I will ask you some questions about health and health providers around here.

13. I would now like to ask you about the sources of health advice and medicine or other treatment that are available to you. Please think about all the places where you can go to get advice, treatment, or drugs if you (or your children) are sick.<br><br>Do you consider the following providers when you (or your children) feel unwell?<br><br>[*Mark all that apply*]	13.1. Drug dispensary, other local store selling medicine	Consultation .... 1<br>Medical advice .... 2<br>Access to medicine .... 3<br>Other reason(s) .... 4<br>Don't consider this provider .... 98<br>Don't know such a provider 99
	13.2. Traditional healer	Consultation .... 1<br>Medical advice .... 2<br>Access to medicine .... 3<br>Other reason(s) .... 4<br>Don't consider this provider .... 98<br>Don't know such a provider 99
	13.3. Pharmacist	Consultation .... 1<br>Medical advice .... 2<br>Access to medicine .... 3<br>Other reason(s) .... 4<br>Don't consider this provider .... 98<br>Don't know such a provider 99
	13.4. Private clinic	Consultation .... 1<br>Medical advice .... 2<br>Access to medicine .... 3<br>Other reason(s) .... 4<br>Don't consider this provider .... 98<br>Don't know such a provider 99
	13.5. Private hospital	Consultation .... 1<br>Medical advice .... 2<br>Access to medicine .... 3<br>Other reason(s) .... 4<br>Don't consider this provider .... 98<br>Don't know such a provider 99
	13.6. Health volunteer	Consultation .... 1<br>Medical advice .... 2<br>Access to medicine .... 3<br>Other reason(s) .... 4<br>Don't consider this provider .... 98<br>Don't know such a provider 99
	13.7. Public primary care unit	Consultation .... 1<br>Medical advice .... 2<br>Access to medicine .... 3<br>Other reason(s) .... 4<br>Don't consider this provider .... 98<br>Don't know such a provider 99

For peer review only - http://bmjopen.bmj.com/site/about/guidelines.xhtml

	**13.8.** Public hospital	Consultation .... 1<br>Medical advice........ 2<br>Access to medicine........ 3<br>Other reason(s) .... 4<br>Don't consider this provider........ 98<br>Don't know such a provider 99
	**13.9.** Other providers or Internet? Specify: __________	Consultation .... 1<br>Medical advice........ 2<br>Access to medicine........ 3<br>Other reason(s) .... 4<br>Don't consider this provider........ 98<br>Don't know such a provider 99

For peer review only

For peer review only - http://bmjopen.bmj.com/site/about/guidelines.xhtml

14. Now if you think again, is there anyone else with whom you talk about health?

	a) What is the nickname of the person?	b) What is the full name of the person?	c) How is this person related to you?<br><br>[*give examples if respondent is unsure about answer categories*]	d) What is the sex of this person?	e) Where does this person live?	f) What is the name of the household head of this person?	g) How often do you interact with this person?	h) How do you interact with this person?<br><br>[*Mark all thatapply*]
**14.1.**<br>Contact 1	Name ______	Name ______	Spouse.. 1<br>Parent .... 2<br>Child .... 3<br>Sibling... 4<br>Other relative .... 5<br>Neighbour .... 6<br>Friend (if not neighbour).................. 7<br>Other villager .... 8<br>Other (specify) _ 9	Female ..1<br>Male 0	In village .... 1<br>(specify: _____)<br><br>Outside village .. 2	Name of household head<br><br>_________	Daily or more often ....4<br>Weekly or few times/week ....3<br>Monthly or few times/month ...2<br>Yearly or few times/year ....1<br>Less often or never 0	Face-to-face.......................... 1<br>Voice call............................... 2<br>Messenger .... 3<br>Other (specify) _____ 4
**14.2.**<br>Contact n	Name	Name	1 2 3 4 5 6 7 8 9	1 0	1 2	Name	0 1 2 3 4	1 2 3 4

For peer review only - http://bmjopen.bmj.com/site/about/guidelines.xhtml

**15.** Did <u>you</u> or <u>a child in your household</u> have an acute illness (not a chronic, long-term condition that comes again and again) or an accident <u>in the last two</u> months? If yes, I will ask you about these illnesses one-by-one. [*if no, continue with Question 19*]	No........................0 ➔ [*Q 16*]<br>Yes......................1 ⬇
[*if yes*:]<br>**15.a** [Confirm if this episode is for respondent or child]	Respondent ....1 ➔ [*Q 15.1*]<br>Child 2
**15.b** How old is the child?	Age in years: _______
**15.c** Is the child female or male	Female ....1<br>Male 0
**15.1.** Can you please describe the symptoms or problem in your own words?	Description of condition:<br>____________________________________
**15.2.** Did [you / the child] receive a diagnosis of the illness from any medical provide, friend, or internet source?<br><br>If so, can you please describe the diagnosis of the illness if you received any and where [you / the child] received it? [*note: the diagnosis might be given by any medical provider including untrained and informal. Record all diagnoses if more than one.*]	a) Diagnosis 1: __________ b) Medical provider 1: 1 2 3 4 5 6 7 8<br>a) Diagnosis n: __________ b) Medical provider n: 1 2 3 4 5 6 7 8<br><br>[*Response codes*]<br>Drug dispensary, other local store selling medicine ....1<br>Traditional healer..........2<br>Pharmacist ....3<br>Private clinic ....4<br>Private hospital ....5<br>Primary care unit ....6<br>Public hospital..........7<br>Other providers or Internet? Specify: ________..........8
**15.3.** When did [you / the child] experience the accident/discomfort (for the first time)	Onset: ___ days / ______ weeks / _____ months ago
**15.4.** Would you describe the illness/accident as "mild," "moderate," or "severe"?	Mild..1<br>Moderate..2<br>Severe 3

15.5. Can you please explain the stages of the treatment? I will ask you step-by-step what you did, starting from the moment [you / the child] first experienced a discomfort.

	**15.5.1.** Step 1 (detection)	Step n
a) What kind of help or treatment did you get at this stage?<br>[*if unsure, specify*]	Ignored /did nothing ....1<br>Self-care (sleep, rest, medicine at home) ....2<br>Care from family and friends (full-time)..........3<br>Treated/consulted at a traditional healer ....4<br>Treated/cons. at a pharmacist..........5<br>Treated/cons. at shop selling drugs..........6<br>Treated/cons. at priv. clinic/hospital..........7<br>Treated/cons. at primary care unit..........8<br>Treated/cons. at a gvt. Hospital..........9<br>Other (specify) _____ 10	1<br>2<br>3<br>4<br>5<br>6<br>7<br>8<br>9<br>10
b) Where did this activity take place?	At home ....1<br>Less than 10 min. from home..........2<br>10 to 29 min. ....3<br>30 to 59 min. ....4<br>60 to 119 min. ....5<br>2 hours or more from home..........6	1<br>2<br>3<br>4<br>5<br>6
c) How did [you / the child] get to the place of the activity? [*select "at home" according to prior responses*]	At home .... 1<br>Walk .... 2<br>Own bicycle .... 3<br>Own motorcycle / Three-wheeler.......... 4<br>Own car / four-wheeler .... 5<br>Taxi or other hired ride.......... 6<br>Public transport.......... 7<br>Other (specify) ___ 8	1<br>2<br>3<br>4<br>5<br>6<br>7<br>8
d) How long did this stage last?<br>[*let respondent choose category; if <1 day, code "1" day*]	Duration:<br>___ days<br>___ weeks<br>___ months	___ days<br>___ weeks<br>__ months
e) Can you please name or describe all the medicines that you received or were prescribed during this step?<br><br>[*include medicine stored at home if "self-care at home"*] [*continue for all medicines received, then complete Questions g to k for each medicine individually*] 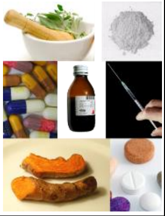	Medicine 1:<br>Name/description: _____<br><br>Medicine n:<br>Name/description: _____	Medicine 1<br><br>Medicine n
f) For how long did [you / the child] take the medicine?<br>[*let respondent choose category; if more than one repeated episode, indicate total duration*]<br>[*for each medicine individually*]	Duration:<br>___ days<br>___ weeks<br>___ months	___ days<br>___ weeks<br>__ months

For peer review only - http://bmjopen.bmj.com/site/about/guidelines.xhtml

g) How often per day did [you / the child] take the medicine?<br><br>[*calculate into daily use according to respondent's chosen frequency*]<br>[*for each medicine individually*]	Frequency: ___ times daily	___ times daily
h) What dosage did [you / the child] normally take?<br>[*let respondent choose category according to type of medicine*]<br>[*for each medicine individually*]	Dosage<br>___ tablets / capsules<br>___ drops (for liquid medicine)<br>___ spoons (for liquid medicine)<br>_____ shots/injections (for intravenous medicine)<br>per time administered	___ tablets<br>___ drops<br>___ spoons<br>___ shots
i) Did [you / the child] take the medicine exactly as it was recommended to you by the person who prescribed/sold them<br>[*for each medicine individually*]	Yes .... 1<br>No .... 0<br>Did not receive advice .... 9<br>Don't know 99	1<br>2<br>9<br>99
j) Did [you / the child] finish the medicine?<br>[*for each medicine individually*]	Yes .... 1<br>No 0	1<br>0
k) Did you or anybody else use a mobile phone during this stage <u>in connection with your condition</u>? [*if no, go to next step*]	Yes .... 1<br>No .... 0 ➔ [*next step*]	1<br>0
l) What was the purpose of using the mobile phone?<br><br>[*Mark all that apply*]	Ask for advice .... 1<br>Call for treatment .... 2<br>Arrange transport .... 3<br>Appointment .... 4<br>Reassure family/friends .... 5<br>Ask for money/supplies .... 6<br>Provider contacting me for information .... 7<br>Treatment reminder .... 8<br>Other (specify) _ 9	1<br>2<br>3<br>4<br>5<br>6<br>7<br>8<br>9
m) Which mobile phone functions did you or anybody else use?<br>[*Mark all that apply*]	Call .... 1<br>SMS .... 2<br>Internet, messenger .... 3<br>Alarm, calendar, reminder, etc. .... 4<br>Other (specify) _ 5	1<br>2<br>3<br>4<br>5
**15.6.** [Have you / has the child] now recovered from the illness/accident?	Yes .... 1<br>No 0	
**15.7.** Was anybody of your personal relationships involved in providing advice or help <u>during the illness</u>? [*record up to ten names*]	Yes .... 1<br>No 0	
[*For district survey*]<br>**15.7.b** How are these people related to you? [*Mark all that apply*]	Spouse .... 1<br>Parent .... 2<br>Child .... 3<br>Sibling .... 4<br>Other relative .... 5<br>Neighbour .... 6<br>Friend (if not neighbour) .... 7<br>Other villager .... 8<br>Other (specify) _ 9	
**15.7.c** What kind of support did they provide? [*Mark all that apply*]	Providing healthcare/attending .... 11<br>Providing advice .... 12<br>Providing medicine .... 13<br>Lending/granting money .... 21<br>Transportation/Lending vehicle .... 22<br>Contacting family/friends .... 23<br>Providing food .... 31<br>Helping with children/housework .... 32<br>Helping with jobs/agriculture work (feeding animals/tending crops/covering shifts, etc.) 33<br>Other (specify) _ 99	

[*For network survey*]	a) What is the name of the person?	b) How is this person related to you?	c) What kind of support was provided?<br>[*mark all that apply*]
**15.7.1.**<br>Contact 1	Name:<br>_______	Spouse .... 1<br>Parent .... 2<br>Child .... 3<br>Sibling .... 4<br>Other relative .... 5<br>Neighbour .... 6<br>Friend (if not neighbour) .... 7<br>Other villager .... 8<br>Other (specify) _ 9	Providing healthcare/attending .... 11<br>Providing advice .... 12<br>Providing medicine .... 13<br>Lending/granting money .... 21<br>Transportation/Lending vehicle .... 22<br>Contacting family/friends .... 23<br>Providing food .... 31<br>Helping with children/housework .... 32<br>Helping with jobs/agriculture work (feeding animals/tending crops/covering shifts, etc.) 33<br>Other (specify) _ 99
**15.7.2.**<br>Contact n	Name	1 2 3 4 5 6 7 8 9	11 12 13 21 22 23 31 32 33 99

For peer review only - http://bmjopen.bmj.com/site/about/guidelines.xhtml

15.8. Did <u>you</u> have another acute illness (not a chronic, long-term condition that comes again and again) or an accident <u>in the last two</u> months?<br>[*if yes, complete another sheet for Question 15*]	Yes .... 1 ➔ [*Q 15*]<br>No .... 0 🡻
16. I would now like to ask you your opinion about medicine. There are no right or wrong answers, I only want to understand what you think. Consider the following medicines:	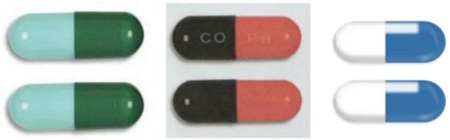
16.1. Have you seen these medicines before?	Yes.................... 1<br>No .... 0 ➔ [*Q 16.4*]

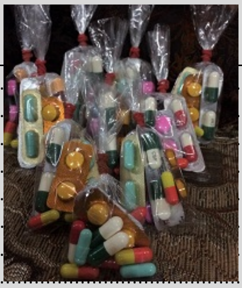

16.2. What do you call this medicine?	Antibiotics ท่านเรียกยานี้ว่าอะไร .... 11<br>Anti-inflammatory ยาแก้อักเสบ .... 12<br>Germ killer ยาฆ่าเชื้อ .... 13<br>Amoxy / Amoxicillin อะม็อกซี่/อะม็อกซี่ซิลลิน .... 14<br>Sore throat medicine ยาแก้เจ็บคอ .... 15<br>Cough medicine ยาแก้ไอ .... 16<br>Pain reliever ยาแก้ปวด .... 17<br>Fever reliever ยาแก้ไข้ .... 18<br>Other (specify: _____) อื่นๆ (โปรดระบุ) .... 98<br>Germ preventer / antibiotic ຢາຕ້ານເຊື້ອ .... 21<br>Amok ຢາຕ້ານເຊື້ອ .... 22<br>Ampi ຢາແອມປີ .... 23<br>Tetra ຢາເຕຕ້າ .... 24<br>Gulolam ກູໂລລາມ .... 25<br>Sepasin ເຊພາຊິນ .... 26<br>Other (specify: __) 99
16.3. What symptoms or illnesses would you use this medicine for?	Fever .... 1<br>Cough .... 2<br>Sore throat .... 3<br>Inflammation .... 4<br>Cold, flu, runny nose .... 5<br>Diarrhoea .... 6<br>Headache .... 7<br>Stomach ache .... 8<br>Muscle pain, other aches .... 9<br>Skin diseases, rashes, lumps .... 10<br>Wounds .... 11<br>Urinary tract infections .... 12<br>Every kind of sickness .... 13<br>Whatever the doctor suggests .... 14<br>Don't know / prefer not to say .... 98<br>Other (specify: __) 99
16.4. Is there any situation for which you would buy this medicine?	Desirable attitude/knowledge .... 1<br>Undesirable attitude/knowledge .... 0<br>No attitude / refuse to answer (respondent is aware, but doesn't reveal attitude) .... 97<br>Answer does not apply to question (respondent may be aware/unaware; satisficing) .... 98<br>Not aware of this medicine (awkward, cannot answer but does not try to satisfy) 99
16.5. Do you prefer other remedies such as herbs or cough syrup to this medicine for [sore throat]?	Desirable attitude/knowledge .... 1<br>Undesirable attitude/knowledge .... 0<br>No attitude / refuse to answer (respondent is aware, but doesn't reveal attitude) .... 97<br>Answer does not apply to question (respondent may be aware/unaware; satisficing) .... 98<br>Not aware of this medicine (awkward, cannot answer but does not try to satisfy) 99
16.6. If you were prescribed this medicine by a doctor and did not finish the course, would you keep it for future use?	Desirable attitude/knowledge .... 1<br>Undesirable attitude/knowledge .... 0<br>No attitude / refuse to answer (respondent is aware, but doesn't reveal attitude) .... 97<br>Answer does not apply to question (respondent may be aware/unaware; satisficing) .... 98<br>Not aware of this medicine (awkward, cannot answer but does not try to satisfy) 99
16.7. Have you heard about drug resistance?<br>(**16.7a** using alternative term "lueng yah" in Lao)	Yes .... 1<br>No 2
16.8. What do you think is drug resistance?<br>(**16.8a** using alternative term "lueng yah" in Lao)	Bacteria are resistant to medicine .... 1<br>Antibiotics become less effective if used wrongly/too much .... 2<br>Medicine in general becomes less effective if used wrongly/too much .... 3<br>Being stubborn to take medicine .... 4<br>Being addicted to medicine .... 5<br>Drug allergy .... 6<br>Lueng yah (drug resistance) .... 7<br>Answer does not relate to drug resistance .... 8<br>Other (specify) .... 98<br>"Don't know" 99
16.9. Can your drug resistance ("*due yah*") spread to other people, for example if you sneeze on them?	Desirable attitude/knowledge .... 1<br>Undesirable attitude/knowledge .... 0<br>No attitude / refuse to answer (respondent is aware, but doesn't reveal attitude) .... 97<br>Answer does not apply to question (respondent may be aware/unaware; satisficing) .... 98<br>Not aware of this medicine (awkward, cannot answer but does not try to satisfy) 99

For peer review only - http://bmjopen.bmj.com/site/about/guidelines.xhtml

Part IV: Household assets
We now come to the last part. Can you please provide me with some information about your household?

**17.** How many rooms does this house have apart from toilet and hallways?	Number of rooms: ________
**18.** What is the electricity situation in your household on a typical day?	Power at all times, no power cuts (90-100%) .... 1<br>Power most of the time, occasional power cuts (>50%) .... 2<br>Power sometimes, frequent power cuts (<50%) .... 3<br>No electricity 4
**19.** What kind of toilet does this house have and is it shared with other people in this community? *[if more than one, choose "best" toilet] [use show card to facilitate answers]*	Unshared flush toilet (e.g. piped sewer system, septic tank, pour flush toilet)........ 1<br>Shared (flush or non-flush) toilet with other community members or public toilet 2<br>No facility, Bush, Field, or others 3
**20.** What is the drinking water source of this house and is it shared with other people in this community? [*use show card to facilitate answers*]	Water piped into house or yard .... 1<br>Water not directly piped into house or yard (e.g. well, borehole, water from spring, rainwater, tanker truck, surface water including rivers, bottled water, etc.)2
**21.** What kind of fuel does this household use for cooking?	Improved fuel source (e.g. Electricity, gas stove, etc.) .... 1<br>Unimproved fuel source (e.g. Coal / Lignite, Charcoal, Wood, Straw / Shrubs / Grass, Animal dung, Agricultural crop residue) .... 2<br>No food cooked in household 3

**22.** I will now ask you for some items in your household. Please tell me…		Number of items in household:	
	22.1.	Have you got a *functioning* **radio** in your household? If so, how many?	__
	22.2.	Have you got a *functioning* **TV** in your household? If so, how many?	__
	22.3.	Have you got a *functioning* **rice cooker** in your household? If so, how many?	__
	22.4.	Have you got a *functioning* **landline telephone** in your household? If so, how many?	__
	22.5.	Have you got a *functioning* **mobile phone** in your household? If so, how many?	__
	22.6.	Have you got a *functioning* **computer** in your household? If so, how many?	__
	22.7.	Have you got a *functioning* **bicycle** in your household? If so, how many?	__
	22.8.	Have you got a *functioning* **scooter, motorcycle, or tricycle** in your household? If so, how many?	__
	22.9.	Have you got a *functioning* **car or truck** in your household? If so, how many?	__
	22.10.	Have you got a *functioning* **tractor** in your household? If so, how many?	__
	22.11.	Have you got a *functioning* **refrigerator or freezer** in your household? If so, how many?	__

**23.** How long does it normally take you to get to the following places?	**23.1.** How long does it take to get to the nearest market?	Less than 10 minutes .... 1<br>10 to 29 minutes .... 2<br>30 to 59 minutes .... 3<br>60 to 119 minutes .... 4<br>2 hours or more 5
	**23.2.** How long does it take to get to the village hall or the village head's house?	Less than 10 minutes .... 1<br>10 to 29 minutes .... 2<br>30 to 59 minutes .... 3<br>60 to 119 minutes .... 4<br>2 hours or more 5
	**23.3.** How long does it take to get to the nearest public or private doctor?	Less than 10 minutes .... 1<br>10 to 29 minutes .... 2<br>30 to 59 minutes .... 3<br>60 to 119 minutes .... 4<br>2 hours or more 5

**24.** What is your religion?	No religion .... 0<br>Buddhist .... 1<br>Christian .... 2<br>Muslim .... 3<br>Spirit (religious belief in Lao) .... 4<br>Other (Specify) __________ .... 5<br>Don't know 99
**25.** What is your nationality?	Thai .... 1<br>Lao .... 2<br>Myanmar/Burmese .... 3<br>Chinese .... 4<br>Other (Specify) __________ .... 9<br>Don't know 99

For peer review only - http://bmjopen.bmj.com/site/about/guidelines.xhtml

**26.** What is your ethnic background?	Thai ....1<br>Tai Yai ....2<br>Akha (E-Koh) ....3<br>Pakakeryor (Karen) ....4<br>Lahu (Muser) ....5<br>Lisu (Lisaw) ....6<br>Hmong (Meaw) ....7<br>Mien (Yao) ....8<br>Burmese ....9<br>Yunnan (Jin Haw) ....10<br>Tai Lue (Tai) ....11<br>Lao ....21<br>Kathuic ....22<br>Bahnaric Khmer ....23<br>Tai Thai ....24<br>Other (Specify) ________ ....30<br>Don't know 99
xi. Interview end time	[*time entered automatically*]
Thank you very much for participating in this survey. [*give gift to respondent*]	
Part V: Interviewer observations [to be completed by interviewer after interview]	
xii. Was the interview completed?	Yes ....1<br>Yes, with difficulties ....2<br>No 3
xiii. Was someone else present during the interview? [*mark all that apply*]	Survey supervisor ....1<br>Other household or family member ....2<br>Medical practitioner ....3<br>Government officer ....4<br>Other (specify) _____ ....5<br>No one 0
xiv. What is your evaluation of the accuracy and trustworthiness of the informant's answers?	Very good ....1<br>Satisfactory ....2<br>Doubtful ....3<br>Very low 4
xv. Were there any unusual circumstances during the interview?	Please describe: ________________________________

For peer review only

INTERVIEW GUIDES – COGNITIVE INTERVIEWS FOR SURVEY TESTING

บทสัมภาษณ์ – การสัมภาษณ์กระบวนการคิดเพื่อการทดสอบแบบสอบถาม

ບົດສໍາພາດ - ການສໍາພາດຂະບວນການຄິດເພື່ອການທົດສອບຄໍາຖາມ

Objective วัตถุประสงค์ ຈຸດປະສົງ

To understand how survey participants understand the survey questions and arrive at their answers. เพื่อเรียนรู้ว่าผู้เข้าร่วมงานวิจัยมีการทำความเข้าใจคำถามที่ใช้ในแบบสอบถามอย่างไร และมีความคิดอย่างไรก่อนตัดสินใจตอบคำถามแต่ละข้อ ເພື່ອຮຽນຮູ້ວ່າຜູ້ເຂົ້າຮ່ວມການຄົ້ນຄ້ວາມີຄວາມເຂົ້າໃຈຄໍາຖາມທີ່ໃຊ້ໃນແບບສອບຖາມແບບໃດ ແລະມີຄວາມຄິດແບບໃດກ່ອນຕັດສິນໃຈຕໍ່ຄໍາຖາມແຕ່ລະຂໍ້

Introduction บทนำ ບົດນໍາ

<Obtaining verbal informed consent in accordance with Oral Consent Script> <ดำเนินการขอคำยินยอมโดยใช้ข้อความสำหรับอธิบายงานวิจัยและขอคำยินยอมเข้าร่วมการวิจัยด้วยวาจา> <ດໍາເນີນການຂໍຄໍາຍິນຍອມໂດຍໃຊ້ຂໍ້ຄວາມສໍາລັບອະທິບາຍວຽກຄົ້ນຄ້ວາແລະຂໍຄໍາຍິນຍອມເຂົ້າຮ່ວມການຄົ້ນຄ້ວາດ້ວຍວາຈາ>

Thank you for agreeing to participate in this study. ขอบคุณท่านสำหรับการตกลงเข้าร่วมการวิจัยครั้งนี้ ຂອບໃຈທ່ານສໍາລັບການຕົກລົງເຂົ້າຮ່ວມການຄົ້ນຄ້ວາໃນຄັ້ງນີ້

Part I – About Yourself ส่วนที่ 1 – เกี่ยวกับตัวท่าน ສ່ວນທີ່ 1 - ກ່ຽວກັບຕົວທ່ານ

Let us start with a few questions about yourself. เราจะเริ่มโดยถามคำถามเกี่ยวกับข้อมูลส่วนตัวของท่าน ເຮົາຈະເລີ່ມໂດຍຖາມຄໍາຖາມກ່ຽວກັບຂໍ້ມູນສ່ວນຕົວຂອງທ່ານ

Guiding question: Who is my informant? คำถามนำ: ผู้ให้สัมภาษณ์คือใคร ຄໍາຖາມນໍາ: ຜູ້ໃຫ້ສໍາພາດແມ່ນໃຜ

1. How old are you? อายุของท่าน ອາຍຸທ່ານ
2. What is your level of education? การศึกษาสูงสุดของท่าน ການສຶກສາສູງສຸດຂອງທ່ານ
3. What is your ethnicity and nationality? เชื้อชาติ และ สัญชาติของท่าน ເຊື້ອຊາດແລະສັນຊາດຂອງທ່ານ

Part II – Practicing the "Think Aloud" Technique ส่วนที่ 2 – เทคนิคการคิดออกเสียง (การบรรยายกระบวนการคิด) ສ່ວນທີ່ 2 - ເທັກນິກການຄິດອອກສຽງ (ການບັນລະຍາຍຂະບວນການຄິດ)

Thank you. As we are trying to improve our survey, you can help us develop the questionnaire by participating in this interview. I would like to ask you to describe your thoughts as you think about the question and the answer as though you are talking to yourself. For example, what do you think as you receive the question? What does the question make you think of? In what way do you want answer to question? How do you arrive at your answer? Let me give you an example so you understand this type of interview better.

Let's say I was asked a question about "What do you see when you go from your home to the nearest temple?" If I was thinking out loud, I would say "[interviewer describing their own village]." So you can see how it works, though that was only one of many ways. The important thing is that there are no right or wrong answers. What really matters is your view and thought process. And this is what I would like you to share with me. So now it is your turn to practice.

ขอบคุณครับ/ค่ะ เนื่องจากเรากำลังอยู่ในช่วงปรับปรุงแบบสอบถาม ท่านสามารถช่วยเราพัฒนาแบบสอบถามนี้ได้โดยการเข้าร่วมการสัมภาษณ์นี้ ซึ่งเราจะขอให้ท่านบรรยายความคิดของท่านในขณะที่ท่านพิจารณาคำถามและคำตอบเสมือนกับท่านกำลังคุยกับตนเอง เช่น ท่านคิดอย่างไรเมื่อท่านได้รับคำถาม คำถามทำให้ท่านนึกถึงอะไรบ้าง ท่านต้องการตอบคำถามเหล่านี้อย่างไร ท่านได้คำตอบมาได้อย่างไร เราจะยกตัวอย่างให้ท่านหนึ่งตัวอย่างเพื่อให้ท่านสามารถเข้าใจการสัมภาษณ์ในลักษณะนี้มากขึ้น

ยกตัวอย่าง ถ้าเราต้องตอบคำถามที่ว่า "ท่านมองเห็นอะไรบ้างหากท่านเดินทางจากบ้านไปวัดที่ใกล้ที่สุด" และเราต้องบรรยายความคิดของเราให้ท่านฟัง เราจะพูดว่า "[ผู้สัมภาษณ์บรรยายการเดินทางในหมู่บ้านของตนเอง]" ซึ่งเป็นเพียงแค่หนึ่งในหลากหลายวิธีที่ท่านสามารถใช้ได้ สิ่งที่สำคัญที่สุดเกี่ยวกับการคิดออกเสียงของท่านคือคำตอบของท่านจะไม่ถูกตัดสินว่าถูกหรือผิด เพราะสิ่งที่เราต้องการเรียนรู้มากที่สุดคือมุมมองและวิธีการคิดของท่าน และนั่นคือสิ่งที่เราอยากให้ท่านแบ่งปันกับเรา ดังนั้นเราจึงขอให้ท่านลองคิดออกเสียงกับเราในคำถามต่อไปนี้

For peer review only - http://bmjopen.bmj.com/site/about/guidelines.xhtml

ຂອບໃຈ ເນື່ອງຈາກເຮົາກຳລັງຢູ່ໃນຊ່ວງປັບປຸງແບບສອບຖາມ ທ່ານສາມາດຊ່ວຍເຮົາພັດທະນາແບບສອບຖາມນີ້ໄດ້ໂດຍການເຂົ້າຮ່ວມການສຳພາດນີ້ ເຊິ່ງເຮົາຈະຂໍໃຫ້ທ່ານບັນລະຍາຍຄວາມຄິດຂອງທ່ານໃນຕອນທີ່ທ່ານກຳລັງພິຈາລະນາຄຳຖາມແລະຄຳຕອບຄືກັບທ່ານກຳລັງລົມກັບຕົນເອງ ເຊັ່ນ ທ່ານຄິດແນວໃດເມື່ອທ່ານໄດ້ຮັບຄຳຖາມ ຄຳຖາມເຮັດໃຫ້ເຮົາຄິດຫາຫຍັງແດ່ ທ່ານຕ້ອງການຕອບຄຳຖາມເຫຼົ່ານີ້ແນວໃດ ທ່ານໄດ້ຄຳຕອບມາໄດ້ແນວໃດ ເຮົາຈະຍົກຕົວຢ່າງໃຫ້ທ່ານໜຶ່ງຕົວຢ່າງເພື່ອໃຫ້ທ່ານສາມາດເຂົ້າໃຈການສຳພາດໃນລັກສະນະນີ້ຫຼາຍຂຶ້ນ.

ຍົກຕົວຢ່າງ ຖ້າເຮົາຕ້ອງຕອບຄຳຖາມວ່າ ທ່ານເບິ່ງເຫັນຫຍັງແດ່ຖ້າທ່ານເດີນທາງຈາກເຮືອນຫາວັດທີ່ໃກ້ທີ່ສຸດ ແລະເຮົາຕ້ອງບັນລະຍາຍຄວາມຄິດຂອງເຮົາໃຫ້ທ່ານຟັງເຮົາຈະເວົ້າວ່າ [illegible]ຜູ້ສຳພາດບັນຍາຍການເດີນທາງໃນໝູ່ບ້ານຂອງຕົນເອງ [illegible]ເຊິ່ງເປັນພຽງໜຶ່ງໃນຫຼາກຫຼາຍວິທີທີ່ທ່ານສາມາດໃຊ້ໄດ້ ສິ່ງທີ່ສຳຄັນທີ່ສຸດກ່ຽວກັບການຄິດອອກສຽງຂອງທ່ານຄືຄຳຕອບຂອງທ່ານຈະບໍ່ຖືກຕັດສິນວ່າຖືກຫຼືຜິດ ເພາະສິ່ງທີ່ເຮົາຕ້ອງການຮຽນຮູ້ຫຼາຍທີ່ສຸດມຸມມອງແລະວິທີການຄິດຂອງທ່ານ ແລະນັ້ນແມ່ນສິ່ງທີ່ເຮົາຢາກໃຫ້ທ່ານແບ່ງປັນກັບເຮົາ ດັ່ງນັ້ນເຮົາຈຶ່ງຂໍໃຫ້ທ່ານລອງຄິດອອກສຽງກັບເຮົາໃນຄຳຖາມຕໍ່ໄປນີ້

Guiding question: Is the respondent comfortable with the "think aloud" technique? คำถามนำ: ผู้ให้สัมภาษณ์สามารถใช้เทคนิคการคิดออกเสียงหรือไม่ ຄຳຖາມນຳ: ຜູ້ໃຫ້ສຳພາດໃຊ້ເຕັກນິກການຄິດອອກສຽງຫຼືບໍ່

4. I would like you to imagine your village. Could you please describe to me what you see when you go from your home to the nearest temple, in as much detail as you can? เราขอให้ท่านนึกภาพหมู่บ้านของท่านและบรรยายให้เราฟังว่าท่านเห็นอะไรเมื่อท่านเดินทางจากบ้านของท่านไปยังวัดที่ใกล้ที่สุด โดยใช้รายละเอียดมากที่สุดเท่าที่ท่านสามารถทำได้ ເຮົາຂໍໃຫ້ທ່ານນຶກພາບໝູ່ບ້ານຂອງທ່ານແລະບັນຍາຍໃຫ້ເຮົາຟັງວ່າທ່ານເຫັນຫຍັງເມື່ອທ່ານເດີນທາງຈາກເຮືອນຂອງທ່ານໄປຫາວັດທີ່ໃກ້ທີ່ສຸດ ໂດຍໃຊ້ລາຍລະອຽດຫຼາຍທີ່ສຸດທີ່ທ່ານສາມາດເຮັດໄດ້

Before we start, I'd like to give you another example that is more closely related to the actual questions from our questionnaire. Say you ask me to list all persons age ≥18 years in my household with their sex and age, I'd go [*interviewer answering question about adults in household*]. *ก่อนที่เราจะเริ่ม เราขอยกตัวอย่างที่ใกล้เคียงกับคำถามจริงกับแบบสอบถามของเราให้ท่านอีกหนึ่งครั้ง สมมติท่านขอให้เราบอกข้อมูลของสมาชิกอายุ 18 ปีหรือมากกว่า ที่อาศัยอยู่ในครัวเรือนนี้ทุกคน รวมถึงเพศและอายุ เราจะพูดว่า "[ผู้สัมภาษณ์ให้คำตอบเกี่ยวกับสมาชิกในครัวเรือนของตนเอง]." ກ່ອນທີ່ເຮົາຈະເລີ່ມເຮົາຂໍຍົກຕົວຢ່າງທີ່ຄ້າຍກັນກັບແບບສອບຖາມຂອງເຮົາ ສົມມຸດທ່ານບອກໃຫ້ເຮົາບອກຂໍ້ມູນຂອງຊະມະຊິກອາຍຸ 18 ປີຫຼືຫຼາຍກ່ວາ ທີ່ອາໄສຢູ່ໃນຄົວເຮືອນນີ້ທຸກຄົນ ລວມເຖິງເພດແລະອາຍຸ ເຮົາຈະເວົ້າວ່າ [[illegible]ຜູ້ສຳພາດໃຫ້ຄຳຕອບກ່ຽວກັບສະມາຊິກໃນຄົວເຮືອນຂອງຕົນເອງ] [illegible]*

List all persons aged 18+ years in household กรอกข้อมูลของสมาชิกอายุ **18** ปีหรือมากกว่า ที่อาศัยอยู่ในครัวเรือนนี้ **ໃສ່ຂໍ້ມູນຂອງຊະມະຊິກອາຍຸ 18 ປີຫຼືຫຼາຍກ່ວາ ທີ່ອາໃສຢູ່ຄົວເຮືອນນີ້**

[*1 respondent per every 5 household members will be selected randomly from this list*] [illegible] [*ຕົວແທນ 1 ຄົນຕໍ່ຊະມະຊິກຈຳນວນ 5 ຄົນ ຈະຖືກສຸ່ມເລືອກໃຫ້ເຂົ້າຮ່ວມການສຳພາດ*]

**Name** ชื่อ ຊື່	**Sex** เพศ ເພດ<br>**(M / F)** (ช/ญ) (ຊ/ຍ)	**Age** อายุ ອາຍຸ<br>**(in completed Years)** (จำนวนปีเต็ม) (ຈຳນວນປີເຕັມ)	**Available for interview today** ผู้เข้าร่วมสามารถให้สัมภาษณ์ในวันนี้หรือไม่ ຜູ້ເຂົ້າຮ່ວມສາມາດໃຫ້ສຳພາດໃນມື້ນີ້ໄດ້ຫຼືບໍ່?<br>**(Yes / No)** (ใช่/ไม่ใช่) (ແມ່ນ/ບໍ່ແມ່ນ)

Part III – Social-Network-Related Survey Questions ส่วนที่ 3 – คำถามเกี่ยวกับเครือข่ายสังคม ສ່ວນທີ່ 3 - ຄຳຖາມກ່ຽວກັບເຄືອຂ່າຍສັງຄົມ

I will now ask you some questions about your interactions with other people within and outside of your village. เราจะเริ่มโดยการถามคำถามท่านเกี่ยวกับปฏิสัมพันธ์ของท่านกับชาวบ้านคนอื่นๆ ทั้งในและนอกหมู่บ้าน ເຮົາຈະເລີ່ມໂດຍການຖາມຄຳຖາມທ່ານກ່ຽວກັບການພົວພັນຂອງທ່ານກັບຊາວບ້ານຄົນອື່ນໆ ທັງໃນແລະພາຍນອກຂອງໝູ່ບ້ານ

5. What are you thinking about when you hear the following question? ท่านมีความคิดอะไรบ้างเมื่อท่านได้ยินคำถามต่อไปนี้ ທ່ານມີຄວາມຄິດຫຍັງແດ່ເມື່ອທ່ານໄດ້ຍິນຄຳຖາມຕໍ່ໄປນີ້

For peer review only

Where do you spend most of your time interacting with other people from your village? ท่านใช้เวลาส่วนมากในการพบปะพูดคุยกับชาวบ้านในหมู่บ้านของท่านในสถานที่ใด ທ່ານໃຊ້ເວລາຫຼາຍທີ່ສຸດໃນການພົບປະໂອ້ລົມກັບຊາວບ້ານໃນໝູ່ບ້ານຂອງທ່ານໃນບ່ອນໃດແດ່<br><br>[*mark all that apply or select 99 if respondent indicates no noteworthy interaction*] [illegible]<br>[*ຕອບໄດ້ຫຼາຍກວ່າ 1 ຕົວເລືອກ ຫຼືເລືອກ 99 ຖ້າບໍ່ມີການພົວພັນທີ່ຊັດເຈນ*]	Field (specify) ไร่นา (โปรดระบุ) ໄຮ່ນາ (ກະລຸນາບອກ) ___ .... 1<br>Temple (specify) วัด (โปรดระบุ) ວັດ (ກະລຸນາບອກ) ___ .... 2<br>Local store (specify) ร้านค้า (โปรดระบุ) ຮ້ານຄ້າ (ກະລຸນາບອກ) ___ .... 3<br>Market (specify) ตลาด (โปรดระบุ) ຕະຫຼາດ (ກະລຸນາບອກ) ___ .... 4<br>Children s[illegible]schools (specify) โรงเรียนของบุตรหลาน (โปรดระบุ) ໂຮງຮຽນຂອງລູກ (ກະລຸນາບອກ) __ .... 5<br>Home (specify) ที่บ้าน (โปรดระบุ) ເຮືອນ (ກະລຸນາບອກ) ____ .... 6<br>Workplace (specify) ที่ทำงาน (โปรดระบุ) ບ່ອນເຮັດວຽກ (ກະລຸນາບອກ) ___ .... 7<br>Other site (specify) อื่นๆ (โปรดระบุ) ອື່ນໆ (ກະລຸນາບອກ) ___ .... 8<br><br>I don t[illegible]nteract notably with others ไม่มีปฏิสัมพันธ์ที่เด่นชัดกับชาวบ้านคนอื่นๆ ບໍ່ມີການພົວພັນທີ່ຊັດເຈນກັບຊາວບ້ານຄົນອື່ນໆ 99

a. How do you understand "interacting?" สำหรับท่าน ท่านคิดว่าคำว่า "พบปะพูดคุย" มีความหมายว่าอย่างไร ສໍາລັບທ່ານ ທ່ານຄິດວ່າ [illegible]ການພົບປະໂອ້ລົມ [illegible]ມີຄວາໝາຍວ່າແນວໃດ

b. What does "most of your time" mean to you? Which time frame did you have in mind? คำว่า "เวลาส่วนมาก" สำหรับท่านมีความหมายว่าอย่างไร ในขณะที่ท่านตอบคำถามในใจของท่านนึกถึงช่วงเวลาใด ຄໍາວ່າ [illegible]ເວລາຫຼາຍທີ່ສຸດ [illegible]ສໍາລັບທ່ານມີຄວາມໝາຍວ່າແບບໃດ ໃນຂະນະທີ່ທ່ານຕອບຄໍາຖາມໃນໃຈຂອງທ່ານນຶກເຖິງຊ່ວງເວລາໃດ

c. What are the possible places where people spend time together? มีที่ใดบ้างที่ชาวบ้านสามารถใช้เวลาร่วมกันได้ ມີບ່ອນໃດແດ່ທີ່ຊາວບ້ານສາມາດໃຊ້ເວລາຮ່ວມກັນໄດ້

d. Please ask us the same question in your own words. กรุณาถามคำถามนี้กับเราโดยใช้คำพูดในแบบของท่านเอง ກະລຸນາຖາມຄໍາຖາມນີ້ກັບເຮົາໂດຍໃຊ້ຄໍາເວົ້າໃນແບບຂອງທ່ານເອງ

e. What kind of things do you normally talk about when you interact with other villagers? โดยปกติเมื่อท่านพบปะกับชาวบ้านคนอื่นๆ ท่านสนทนากันเรื่องอะไรบ้าง ໂດຍປົກກະຕິທ່ານພົບປະກັບຊາວບ້ານຄົນອື່ນໆ ທ່ານລົມເລື່ອງຫຍັງແດ່

For peer review only

6. I will now ask you a set of questions, regarding the people you interact with. ชุดคำถามต่อไปจะถามถึงบุคคลที่ท่านพบปะพูดคุยด้วย ຊຸດຄຳຖາມຕໍ່ໄປຈະຖາມເຖີງບຸກຄົນທີ່ທ່ານພົບປະໂອ້ລົມນຳ

With whom do you interact on a regular basis? โดยปกติในชีวิตประจำวันท่านพบปะพูดคุยกับใครบ้าง ໂດຍປົກກະຕິໃນຊີວິດປະຈຳວັນທ່ານພົບປະໂອ້ລົມກັບໃຜແດ່

	a) What is the name of the person? ชื่อ ຊື່	b) How is this person related to you? ความสัมพันธ์กับท่าน ຄວາມສຳພັນກັບທ່ານ<br>[*give examples if respondent is unsure about answer categories*]<br>[illegible]<br>[*ຖ້າຜູ້ໃຫ້ສຳພາດບໍ່ໝັ້ນໃຈໃນໝວດຄຳຕອບ ໃຫ້ຍົກຕົວຢ່າງຄຳຕອບໄດ້*]	c) What is the sex of this person? เพศ ເພດ	d) Where does this person live? ที่อยู่ ທີ່ຢູ່	e) What is the name of the household head of this person? ใครคือหัวหน้าครัวเรือนของบุคคลนี้ ໃຜແມ່ນຫົວໜ້າຄົວເຮືອນຂອງບຸກຄົນນີ້	f) How often do you interact with this person? ท่านพบปะพูดคุยกับบุคคลนี้บ่อยเท่าใด ທ່ານພົບປະໂອ້ລົມກັບບຸກຄົນນີ້ບ່ອຍປານໃດ	g) How do you interact with this person? ท่านพบปะพูดคุยกับบุคคลนี้อย่างไร ທ່ານພົບປະໂອ້ລົມກັບບຸກຄົນນີ້ແນວໃດ<br>[*Mark all that apply*] [illegible] [*ຕອບໄດ້ຫຼາຍກ່ວາ 1 ຕົວເລືອກ*]	h) How often do your conversations relate to health and well-being? ท่านมีการสนทนากับบุคคลนี้เกี่ยวกับเรื่องสุขภาพบ่อยเท่าใด ທ່ານໂອ້ລົມກັບບຸກຄົນນີ້ເລື່ອງສຸຂະພາບບ່ອຍປານໃດ<br>[*If respondent does not discuss health, then move on to next contact after indicating "never"*] [illegible] [*ຖ້າຜູ້ໃຫ້ສຳພາດບໍ່ສະບາຍໃຈທີ່ຈະລົມເລື່ອງສຸຂະພາບໃຫ້ຜູ້ສຳພາດໃສ່ລະຫັດ 0 ແລະຂ້າມໄປຫາບຸກຄົນຕໍ່ໄປ*]	i) What health-related subjects do your conversations cover? หัวข้อการสนทนาเกี่ยวกับสุขภาพของท่านกับบุคคลนี้คือเรื่องอะไร ຫົວຂໍ້ສົນທະນາກ່ຽວກັບສຸຂະພາບຂອງທ່ານກັບບຸກຄົນນີ້ແມ່ນເລື່ອງຫຍັງ<br>[*Mark all that apply*] [illegible] [*ຕອບໄດ້ຫຼາຍກ່ວາ 1 ຕົວເລືອກ*]
Contact 1<br>คนที่ 1<br>ຄົນທີ່ 1	Name ชื่อ ຊື່ _____	Spouse คู่สมรส ຄູ່ແຕ່ງດອງ........ 1<br>Parent บิดามารดา ພໍ່ແມ່............. 2<br>Child บุตร ລູກ...................... 3<br>Sibling พี่น้อง ອ້າຍນ້ອງ .... 4<br>Other relative ญาติ ພີ່ນ້ອງ ...... 5<br>Neighbour เพื่อนบ้าน ເພື່ອນບ້ານ . 6<br>Friend (if not neighbour) เพื่อน (ที่ไม่ใช่เพื่อนบ้าน) ໝູ່ (ທີ່ບໍ່ແມ່ນເພື່ອນບ້ານ) .... 7<br>Other villager ชาวบ้านคนอื่นๆ ຊາວບ້ານຄົນອື່ນໆ.................... 8<br>Other (specify) อื่นๆ (โปรดระบุ) ອື່ນໆ (ກະລຸນາບອກ) _ 9	Female..1 หญิง ຍິງ<br>Male.....2 ชาย ຊາຍ	In village ....1 (specify: _____) ในหมู่บ้าน (โปรดระบุ: __) ໃນໝູ່ບ້ານ(ກະລຸນາບອກ: __)<br>Outside village ...2 (specify: _____) นอกหมู่บ้าน (โปรดระบุ: __) ນອກໝູ່ບ້ານ(ກະລຸນາບອກ: __)	Name of household head ชื่อหัวหน้าครัวเรือน: ຊື່ຫົວໜ້າຄົວເຮືອນ<br>_______	Daily or more often ทุกวันหรือบ่อยกว่า ທຸກມື້ຫຼືໜ້ອຍກ່ວາ.........4<br>Weekly or few times/week ทุกสัปดาห์หรือมากกว่าหนึ่งครั้งต่อสัปดาห์ ທຸກອາທິດຫຼືຫຼາຍກ່ວາໜຶ່ງເທື່ອຕໍ່ອາທິດ...............................3<br>Monthly or few times/month ทุกเดือนหรือมากกว่าหนึ่งครั้งต่อเดือน ທຸກເດືອນຫຼືຫຼາຍກ່ວາໜຶ່ງເທື່ອຕໍ່າເດືອນ................................2<br>Yearly or few times/year ทุกปีหรือมากกว่าหนึ่งครั้งต่อปี ທຸກປີຫຼືຫຼາຍກວ່າໜຶ່ງເທື່ອຕໍ່ປີ 1	Face-to-face ตัวต่อตัว ໂຕຕໍ່ໂຕ..........1<br>Voice call โทรศัพท์ ໂທລະສັບ ....2<br>Messenger ติดต่อสื่อสารโดยใช้อินเตอร์เน็ต ສື່ສານໂດຍໃຊ້ອິນເຕີເນັດ.......3<br>Other (specify) อื่นๆ (โปรดระบุ) ອື່ນໆ (ກະລຸນາບອກ)<br>______ 4	Always (100%) เป็นประจำ (100%) ເປັນປະຈຳ (100%) .. 5<br>Nearly always (<100%) เกือบเป็นประจำ (<100%) ເກືອບເປັນປະຈຳ (<100%) .... 4<br>Often (<90%) บ่อยครั้ง (<90%) ບ່ອຍ (<90%) .... 3<br>Sometimes (<50%) บางครั้ง (<50%) ບາງຄັ້ງ (<50%)........ 2<br>Hardly ever (<10%) แทบไม่เคย (<10%) ເກືອບບໍ່ເຄີຍ (<10%). 1<br>Never (0%) ไม่เคยเลย (0%).... ບໍ່ເຄີຍເລີຍ.......................... 0<br>➔ [*go to next contact*]<br>[illegible] [*ຂ້າມໄປບຸກຄົນຕໍ່ໄປ*]	Talk about general well-being ไต่ถามสารทุกข์สุกดิบทั่วไป ຖາມກ່ຽວກັບການເປັນຢູ່ທົ່ວໄປ ....1<br>Update on health knowledge/news อัพเดทเรื่องความรู้หรือข่าวสารสุขภาพใหม่ๆ ອັບເດດເລື່ອງຄວາມຮູ້ຫຼືຂ່າວສານສຸຂະພາບໃໝ່ໆ................2<br>Update on personal health status .... อัพเดทเรื่องสุขภาพส่วนตัว ອັບເດດເລື່ອງສຸຂະພາບສ່ວນຕົວ ....3<br>Advice on diagnosis ให้คำแนะนำเกี่ยวกับการวินิจฉัย ໃຫ້ຄຳແນະນຳກ່ຽວກັບການວິເຄາະພະຍາດ.......................4<br>Advice on doctors ให้คำแนะนำเกี่ยวกับแพทย์ ໃຫ້ຄຳແນະນຳກ່ຽວກັບໝໍ..5<br>Advice on treatment/medicine ให้คำแนะนำเกี่ยวกับวิธีการรักษา/ยา ໃຫ້ແນະນຳກ່ຽວກັບວິທີຮັກສາ/ຢາ 6

For peer review only - http://bmjopen.bmj.com/site/about/guidelines.xhtml

						Less often or never ไม่ค่อยบ่อย หรือไม่เคยเลย ບໍ່ເຄີຍເລີຍ 0			Emotional support/reassure ให้กำลังใจ ให้ความมั่นใจ ໃຫ້ກຳລັງໃຈ ໃຫ້ຄວາມໝັ້ນໃຈ ....7<br>Provide help during illness ให้ความช่วยเหลือระหว่างการป่วย ໃຫ້ຄວາມຊ່ວຍເຫຼືອຕອນບໍ່ສະບາຍ ....8<br>Other (specify) อื่นๆ (โปรดระบุ) ອື່ນໆ (ກະລຸນາບອກ)___ .9
Contact 2 คนที่ 2 ຄົນທີ່ 2	Name ชื่อ ຊື່	1 2 3 4 5 6 7 8 9	1 2	1 2	Name ชื่อ ຊື່	0 1 2 3 4	1 2 3 4	0 1 2 3 4 5	1 2 3 4 5 6 7 8 9
[*continue until Contact x maximum 10 contacts*] [[illegible] n [illegible] 10 [illegible]] [*ບັນທຶກບຸກຄົນທີ່ມີຄວາມກ່ຽວຂ້ອງລະຫ່ວາງອາການເຈັບປ່ວຍທຸກຄົນ ບໍ່ຫຼາຍກ່ວາ 10 ຄົນ*]	Name ชื่อ ຊື່	1 2 3 4 5 6 7 8 9	1 2	1 2	Name ชื่อ ຊື່	0 1 2 3 4	1 2 3 4	0 1 2 3 4 5	1 2 3 4 5 6 7 8 9

a. How did you go about answering these questions? ท่านมีวิธีการตอบคำถามเหล่านี้อย่างไร ທ່ານມີວິທີການຕອບຄຳຖາມເຫຼົ່ານີ້ແນວໃດ

b. What did you imagine while answering the question, e.g. the “interaction setting” that you had in mind? ในขณะที่ท่านตอบคำถามเหล่านี้ท่านมีภาพอะไรอยู่ในใจ เช่น ลักษณะของสถานที่ ฯลฯ ໃນຂະນະທີ່ທ່ານຕອບຄຳຖາມເຫຼົ່ານີ້ທ່ານມີພາບຫຍັງຢູ່ໃນໃຈ ເຊັ່ນ ລັກຊະນະຂອງສະຖານທີ່ ແລະອື່ນໆ

c. What time period were you thinking of? จากคำตอบที่ท่านให้เราเกี่ยวกับบุคคลที่ท่านมีปฏิสัมพันธ์ด้วย ท่านนึกถึงช่วงเวลาใด ຈາກຄຳຕອບທີ່ທ່ານໃຫ້ເຮົາກ່ຽວກັບບຸກຄົນທີ່ທ່ານມີປະຕິສຳພັນນຳ ທ່ານນຶກເຖິງຊ່ວງເວລາໃດ

d. How did you come up with the order of people? Why did you think of these people first rather than others? ท่านมีการเรียงลำดับบุคคลเหล่านี้อย่างไร เพราะเหตุใดท่านจึงบอกชื่อบุคคลเหล่านี้กับเราเป็นลำดับแรกๆ หากเปรียบเทียบกับบุคคลอื่น ທ່ານມີການລຽງລຳດັບບຸກຄົນເຫຼົ່ານີ້ແບບໃດ ຍ້ອນຫຍັງທ່ານຈຶ່ງບອກຊື່ບຸກຄົນເຫຼົ່ານີ້ກັບກ່ອນຄົນອື່ນ

e. What is the best way we can find these people from your interaction circle? How would you describe where they live? หากเราต้องการพูดคุยกับบุคคลที่ท่านกล่าวถึงเหล่านี้ เราสามารถพบพวกเขาได้อย่างไร ท่านจะสามารถอธิบายที่อยู่ของพวกเขาได้อย่างไร ຖ້າເຮົາຕ້ອງການລົມກັບບຸກຄົນທີ່ທ່ານກ່າວເຖິງເຫຼົ່ານີ້ ເຮົາສາມາດພົບພົບພວກເຂົາໄດ້ແນວໃດ ທ່ານສາມາດອະທິບາຍທີ່ຢູ່ຂອງພວກເຂົາໄດ້ແນວໃດ

For peer review

Part IV – Health-Related Survey Questions ส่วนที่ 4 – คำถามเกี่ยวกับสุขภาพ ສ່ວນທີ່ 4 - ຄຳຖາມກ່ຽວກັບສຸຂະພາບ

Thank you for this. Now we come to a part where I will ask you some questions about health and health providers around here. ขอบคุณครับ/ค่ะ คำถามช่วงต่อไปจะเป็นเรื่องสุขภาพและสถานบริการสุขภาพในพื้นที่นี้ ຂອບໃຈ ຄຳຖາມຕໍ່ໄປນີ້ຈະເປັນຄຳຖາມກ່ຽວກັບສຸຂະພາບ ແລະສະຖານບໍລິການສຸຂະພາບໃນພື້ນທີ່ນີ້

7. For the following question, can you please “think out loud” about your answers? เราขอให้ท่าน “คิดออกเสียง” ในระหว่างที่ท่านตอบคำถามต่อไปนี้ ເຮົາຂໍໃຫ້ທ່ານ ☒ິດອອກສຽງ ☒ີນລະຫ່ວາງການຕອບຄຳຖາມຕໍ່ໄປນີ້

I would now like to ask you about the sources of health advice and medicine or other treatment that are available to you. Please think about all the places where you can go to get advice, treatment, or drugs if you (or your children) are sick. คำถามต่อไปเป็นคำถามเกี่ยวกับสถานที่หรือบุคคลที่ท่านสามารถรับคำปรึกษา รับยา หรือ รับการรักษาอื่นๆ ได้ กรุณานึกถึงสถานที่ทุกแห่งหรือคนทุกคนที่ท่านสามารถรับคำปรึกษา รับการรักษา หรือรับยา หากท่านหรือเด็กในปกครองของท่านป่วย ຄຳຖາມຕໍ່ໄປເປັນຄຳຖາມກ່ຽວກັບສະຖານທີ່ຫຼືບຸກຄົນທີ່ທ່ານສາມາດຮັບຄຳປຶກສາ ຮັບຢາ ຫຼືຮັບການຮັກສາອື່ນໆ ໄດ້ ກະລຸນານຶກເຖິງສະຖານທີ່ທຸກແຫ່ງຫຼືບຸກຄົນທຸກຄົນທີ່ທ່ານສາມາດຮັບຄຳປຶກສາ ຮັບການຮັກສາ ຫຼືຮັບຢາ ຖ້າຫາກທ່ານຫຼືເດັກທີ່ທ່ານດູແລບໍ່ສະບາຍ<br><br>Which of the following options do you consider for medical treatment, advice, or to get medicine? ท่านเคยได้รับคำปรึกษา รับยา หรือ รับ	Drug dispensary, other local store selling medicine ร้านขายของชำหรือร้านค้าทั่วไปที่มีการจำหน่ายยา ຮ້ານທົ່ວໄປທີ່ມີການຂາຍຢາ	Medical treatment เพื่อการรักษา ສຳລັບການປິ່ນປົວ .... 1<br>Medical advice เพื่อคำแนะนำในการรักษา ສຳລັບຄຳແນະນຳໃນການປິ່ນປົວ .... 2<br>Access to medicine เพื่อซื้อ/รับยา ສຳລັບຊື້/ຮັບຢາ .... 3<br>Don t☒consider this provider ไม่มีการใช้บริการประเภทนี้ ບໍ່ມີການໃຊ້ບໍລິການປະເພດນີ້..0<br>Don t☒know such a provider ไม่รู้จักผู้ให้บริการประเภทนี้ ບໍ່ຮູ້ຈັກຜູ້ໃຫ້ບໍລິການປະເພດນີ້.99
	Traditional healer หมอชาวบ้าน ໝໍຊາວບ້ານ	Medical treatment เพื่อการรักษา ສຳລັບການປິ່ນປົວ .... 1<br>Medical advice เพื่อคำแนะนำในการรักษา ສຳລັບຄຳແນະນຳໃນການປິ່ນປົວ .... 2<br>Access to medicine เพื่อซื้อ/รับยา ສຳລັບຊື້/ຮັບຢາ .... 3<br>Don t☒consider this provider ไม่มีการใช้บริการประเภทนี้ ບໍ່ມີການໃຊ້ບໍລິການປະເພດນີ້..0<br>Don t☒know such a provider ไม่รู้จักผู้ให้บริการประเภทนี้ ບໍ່ຮູ້ຈັກຜູ້ໃຫ້ບໍລິການປະເພດນີ້.99
	Pharmacist เภสัชกร ຜູ້ຂາຍຢາ	Medical treatment เพื่อการรักษา ສຳລັບການປິ່ນປົວ .... 1<br>Medical advice เพื่อคำแนะนำในการรักษา ສຳລັບຄຳແນະນຳໃນການປິ່ນປົວ .... 2<br>Access to medicine เพื่อซื้อ/รับยา ສຳລັບຊື້/ຮັບຢາ .... 3<br>Don t☒consider this provider ไม่มีการใช้บริการประเภทนี้ ບໍ່ມີການໃຊ້ບໍລິການປະເພດນີ້..0<br>Don t☒know such a provider ไม่รู้จักผู้ให้บริการประเภทนี้ ບໍ່ຮູ້ຈັກຜູ້ໃຫ້ບໍລິການປະເພດນີ້.99
	Private clinic คลินิกเอกชน ຄລີນິກເອກະຊົນ	Medical treatment เพื่อการรักษา ສຳລັບການປິ່ນປົວ .... 1<br>Medical advice เพื่อคำแนะนำในการรักษา ສຳລັບຄຳແນະນຳໃນການປິ່ນປົວ .... 2<br>Access to medicine เพื่อซื้อ/รับยา ສຳລັບຊື້/ຮັບຢາ .... 3<br>Don t☒consider this provider ไม่มีการใช้บริการประเภทนี้ ບໍ່ມີການໃຊ້ບໍລິການປະເພດນີ້..0<br>Don t☒know such a provider ไม่รู้จักผู้ให้บริการประเภทนี้ ບໍ່ຮູ້ຈັກຜູ້ໃຫ້ບໍລິການປະເພດນີ້.99

For peer review only - http://bmjopen.bmj.com/site/about/guidelines.xhtml

การรักษา จากใครบ้าง ທ່ານເຄີຍໄດ້ຮັບຄໍາປຶກສາ ຮັບຢາ ຫຼືຮັບການຮັກສາອື່ນຈາກໃຜແດ່<br><br>[*Mark all that apply*] [illegible] [*ຕອບໄດ້ຫຼາຍກ່ວາ 1 ຕົວເລືອກ*]	Private hospital โรงพยาบาลเอกชน ໂຮງໝໍເອກະຊົນ	Medical treatment เพื่อการรักษา ສໍາລັບການປິ່ນປົວ .... 1<br>Medical advice เพื่อคำแนะนำในการรักษา ສໍາລັບຄໍາແນະນໍາໃນການປິ່ນປົວ .... 2<br>Access to medicine เพื่อซื้อ/รับยา ສໍາລັບຊື້/ຮັບຢາ .... 3<br>Don't consider this provider ไม่มีการใช้บริการประเภทนี้ ບໍ່ມີການໃຊ້ບໍລິການປະເພດນີ້.. 0<br>Don't know such a provider ไม่รู้จักผู้ให้บริการประเภทนี้ ບໍ່ຮູ້ຈັກຜູ້ໃຫ້ບໍລິການປະເພດນີ້. 99
	Primary care unit สถานีอนามัยของรัฐ ສະຖານີອານາໄມຂອງລັດ	Medical treatment เพื่อการรักษา ສໍາລັບການປິ່ນປົວ .... 1<br>Medical advice เพื่อคำแนะนำในการรักษา ສໍາລັບຄໍາແນະນໍາໃນການປິ່ນປົວ .... 2<br>Access to medicine เพื่อซื้อ/รับยา ສໍາລັບຊື້/ຮັບຢາ .... 3<br>Don't consider this provider ไม่มีการใช้บริการประเภทนี้ ບໍ່ມີການໃຊ້ບໍລິການປະເພດນີ້.. 0<br>Don't know such a provider ไม่รู้จักผู้ให้บริการประเภทนี้ ບໍ່ຮູ້ຈັກຜູ້ໃຫ້ບໍລິການປະເພດນີ້. 99
	Public hospital โรงพยาบาลรัฐ ໂຮງໝໍລັດ	Medical treatment เพื่อการรักษา ສໍາລັບການປິ່ນປົວ .... 1<br>Medical advice เพื่อคำแนะนำในการรักษา ສໍາລັບຄໍາແນະນໍາໃນການປິ່ນປົວ .... 2<br>Access to medicine เพื่อซื้อ/รับยา ສໍາລັບຊື້/ຮັບຢາ .... 3<br>Don't consider this provider ไม่มีการใช้บริการประเภทนี้ ບໍ່ມີການໃຊ້ບໍລິການປະເພດນີ້.. 0<br>Don't know such a provider ไม่รู้จักผู้ให้บริการประเภทนี้ ບໍ່ຮູ້ຈັກຜູ້ໃຫ້ບໍລິການປະເພດນີ້. 99
	Other providers or Internet? Specify: ผู้ให้บริการรูปแบบอื่นๆ หรือ อินเตอร์เน็ต (โปรดระบุ) ຜູ້ບໍລິການໃນຮູບແບບອື່ນໆຫຼືອິນເຕີເນັດ (ກະລຸນາບອກ) __________	Medical treatment เพื่อการรักษา ສໍາລັບການປິ່ນປົວ .... 1<br>Medical advice เพื่อคำแนะนำในการรักษา ສໍາລັບຄໍາແນະນໍາໃນການປິ່ນປົວ .... 2<br>Access to medicine เพื่อซื้อ/รับยา ສໍາລັບຊື້/ຮັບຢາ .... 3<br>Don't consider this provider ไม่มีการใช้บริการประเภทนี้ ບໍ່ມີການໃຊ້ບໍລິການປະເພດນີ້.. 0<br>Don't know such a provider ไม่รู้จักผู้ให้บริการประเภทนี้ ບໍ່ຮູ້ຈັກຜູ້ໃຫ້ບໍລິການປະເພດນີ້. 99

a. Do you choose any of these providers for specific symptoms or illnesses? ท่านมีการเลือกสถานบริการสุขภาพแห่งใดแห่งหนึ่งสำหรับรักษาโรคใดโดยเฉพาะหรือไม่ ທ່ານມີການເລືອກສະຖານສຸຂະພາບທີ່ໃດທີ່ໜຶ່ງສໍາລັບກວດພະຍາດໃດໂດຍສະເພາະຫຼືບໍ່

b. Do you think these questions are too hard? ท่านรู้สึกว่าคำถามเหล่านี้ยากเกินไปหรือไม่ ທ່ານຮູ້ສຶກວ່າຄໍາຖາມເຫຼົ່ານີ້ຍາກເກີນໄປຫຼືບໍ່

c. How big of an area do you think of when you answered these questions? E.g. within the village, the province, the surrounding provinces ในขณะที่ท่านตอบคำถามเหล่านี้ ท่านนึกถึงสถานบริการสุขภาพภายในบริเวณใด เช่น ภายในหมู่บ้าน ภายในจังหวัด หรือรวมจังหวัดรอบนอกด้วย ຕອນທີ່ທ່ານຕອບຄໍາຖາມເຫຼົ່ານີ້ ທ່ານນຶກເຖິງສະຖານທີ່ບໍລິການສຸຂະພາບພາຍໃນບໍລິເວນໃດ ເຊັ່ນ ພາຍໃນໝູ່ບ້ານ ພາຍໃນແຂວງ ຫຼືແຂວງຮອບນອກ

d. Are there other forms of health provider that you could use but we have not talked about? มีสถานบริการสุขภาพลักษณะอื่นที่ท่านสามารถใช้บริการได้แต่เรายังไม่ได้พูดถึงอีกหรือไม่ ມີສະຖານທີ່ບໍລິການສຸຂະພາບລັກຊະນະອື່ນທີ່ທ່ານສາມາດໃຊ້ບໍລິການໄດ້ແຕ່ເຮົາຍັງບໍ່ໄດ້ເວົ້າເຖິງອີກຫຼືບໍ່

8. I will now ask you another series of questions regarding a recent illness. ต่อไปเราจะถามคำถามเกี่ยวกับอาการป่วยครั้งล่าสุดของท่าน ຕໍ່ໄປນີ້ເຮົາຈະຖາມຄໍາຖາມກ່ຽວກັບການເຈັບເປັນຄັ້ງລ່າສຸດຂອງທ່ານ

Did you have an acute illness (not a chronic, long-term condition that comes again and again) or an accident in the last two months? ในช่วงสองเดือนที่ผ่านมา ท่านมีอาการป่วย (ไม่รวมโรคเรื้อรังหรือโรคที่เป็นๆ หายๆ) หรือประสบอุบัติเหตุ หรือไม่ ໃນຊ່ວງສອງເດືອນທີ່ຜ່ານມາ ທ່ານມີອາການເຈັບເປັນ (ບໍ່ລວມພະຍາດຊໍາເຮື້ອຫຼືພະຍາດທີ່ເປັນຕໍ່ເນື່ອງ) ຫຼືອຸປະຕິເຫດຫຼືບໍ່?	No ไม่ใช่ ບໍ່ແມ່ນ .... 2<br>Yes ใช่ ແມ່ນ............. 1
[*if yes:*] [illegible] [*ຖ້າແມ່ນ:*]	Description of condition: คำอธิบายอาการ ຄໍາອະທິບາຍອາການ ______________________________

For peer review only - http://bmjopen.bmj.com/site/about/guidelines.xhtml

Can you please describe the symptoms or problem in your own words? กรุณาอธิบายอาการหรือปัญหาในแบบที่ท่านเข้าใจ ກະລຸນາອະທິບາຍອາການຫຼືບັນຫາໃນແບບທີ່ທ່ານເຂົ້າໃຈ	___________________________________

For peer review only

Can you please describe the diagnosis of the illness if you received any and where you received it?<br>[*note: the diagnosis might be given by any medical provider including untrained and informal. Record all diagnoses if more than one.*] หากท่านได้รับการวินิจฉัยอาการ กรุณาอธิบายการวินิจฉัยนั้นและระบุแหล่งที่มา [หมายเหตุ: ผู้ให้สัมภาษณ์อาจได้รับการวินิจฉัยจากผู้ให้บริการทางสุขภาพลักษณะใดก็ได้ ซึ่งรวมถึงผู้ให้บริการที่ไม่รับการศึกษาด้านการแพทย์และผู้ให้บริการนอกระบบ กรอกข้อมูลการวินิจฉัยทั้งหมด หากผู้ให้สัมภาษณ์ได้รับการวินิจฉัยมากกว่า 1 ครั้ง] ຖ້າຫາກທ່ານໄດ້ຮັບການວິເຄາະພະຍາດ ກະລຸນາອະທິບາຍການວິເຄາະພະຍາດເຫຼື່ອນັ້ນແລະລະບຸແຫຼ່ງຂໍ້ມູນ [ຫມາຍເຫດ: ຜູ້ໃຫ້ສໍາພາດອາດໄດ້ຮັບການວິເຄາະພະຍາດຈາກຜູ້ໃຫ້ບໍລິການສຸຂະພາບປະເພດໃດກໍ່ໄດ້ ເຊິ່ງຮວມເຖິງຜູ້ໃຫ້ບໍລິການສຸຂະພາບທີ່ບໍ່ມີການສຶກສາທາງການແພດແລະຜູ້ໃຫ້ບໍລິການນອກລະບົບ ຕື່ມໃສ່ຂໍ້ມູນການວິເຄາະພະຍາດທັງຫມົດ ຖ້າຜູ້ໃຫ້ສໍາພາດໄດ້ຮັບການວິເຄາະພະຍາດຫຼາຍກ່ວາ 1 ຄັ້ງ]<br>[*Response codes* รหัสคำตอบ ລະຫັດຄໍາຕອບ]<br>Drug dispensary, other local store selling medicine<br>ร้านขายของชำหรือร้านค้าทั่วไปที่มีการจำหน่ายยา ຮ້ານທົ່ວໄປທີ່ມີການຂາຍຢາ ....1<br>Traditional healer หมอชาวบ้าน ໝໍຊາວບ້ານ ....2<br>Pharmacist เภสัชกร ຜູ້ຂາຍຢາ ....3<br>Private clinic คลินิกเอกชน ຄລີນິກເອກະຊົນ ....4<br>Private hospital โรงพยาบาลเอกชน ໂຮງໝໍເອກະຊົນ ....5<br>Primary care unit สถานีอนามัยของรัฐ ສະຖານີອານາໄມຂອງລັດ......................6<br>Public hospital โรงพยาบาลรัฐ ໂຮງໝໍລັດ ....7<br>Other providers or Internet? Specify: ผู้ให้บริการรูปแบบอื่นๆ หรืออินเตอร์เน็ต (โปรดระบุ) ຜູ້ບໍລິການໃນຮູບແບບອື່ນໆຫຼືອິນເຕີເນັດ (ກະລຸນາບອກ) ________ 8	Diagnosis 1 การวินิจฉัยโรค 1 ການວິເຄາະພະຍາດ 1:<br>____________________________________<br>Medical provider 1 ผู้ให้บริการทางสุขภาพ 1 ຜູ້ໃຫ້ບໍລິການສຸຂະພາບ 1:<br>1 2 3 4 5 6 7 8<br>Diagnosis 2 การวินิจฉัยโรค 2 ການວິເຄາະພະຍາດ 2:<br>____________________________________<br>Medical provider 2 ผู้ให้บริการทางสุขภาพ 2 ຜູ້ໃຫ້ບໍລິການສຸຂະພາບ 2:<br>1 2 3 4 5 6 7 8<br>Diagnosis 3 การวินิจฉัยโรค 3 ການວິເຄາະພະຍາດ 3:<br>____________________________________<br>Medical provider 3 ผู้ให้บริการทางสุขภาพ 3 ຜູ້ໃຫ້ບໍລິການສຸຂະພາບ 3:<br>1 2 3 4 5 6 7 8
When did you experience the accident/discomfort (for the first time) ท่านเริ่มมีอาการป่วยหรือประสบอุบัติเหตุตั้งแต่เมื่อใด ທ່ານເລີ່ມມີອາການເຈັບເປັນຫຼືຖືກອຸບັດຕິເຫດຕັ້ງແຕ່ເມື່ອໃດ?	Onset: ___ days / ______ weeks / _____ months ago<br>เริ่มอาการป่วย/อุบัติเหตุ: _______ วัน / _______ สัปดาห์ / ______ เดือนที่แล้ว<br>ເລີ່ມມີອາການບໍ່ສະບາຍຫຼືອຸບັດຕິເຫດ: ___ ມື້ / ___ ອາທິດ / ___ ເດືອນກ່ອນ
[illegible] moderate, or severe [illegible] ท่านคิดว่าการป่วยหรืออุบัติเหตุครั้งนั้นอยู่ในขั้นน้อย ปานกลาง หรือหนัก ທ່ານຄິດວ່າອາການເຈັບເປັນຫຼືອຸບັດຕະເຫດຄັ້ງນັ້ນຢູ່ຂັ້ນນ້ອຍ, ປານກາງ, ຫຼືໜັກ	Mild น้อย ນ້ອຍ.................................1<br>Moderate ปานกลาง ປານກາງ ....2<br>Severe หนัก ໜັກ.............................3

a. Did you feel comfortable talking about your health? ท่านรู้สึกสบายใจ (หรืออึดอัดใจ) ในการพูดคุยเรื่องสุขภาพหรือไม่ ທ່ານຮູ້ສຶກສະບາຍໃຈ (ຫຼືອຶດອັດໃຈ) ໃນການລົມເລື່ອງສຸຂະພາບຫຼືບໍ່

b. Do you think these questions are too hard? ท่านรู้สึกว่าคำถามเหล่านี้ยากเกินไปหรือไม่ ທ່ານຮູ້ສຶກວ່າຄໍາຖາມເຫຼົ່ານີ້ຍາກເກີນໄປຫຼືບໍ່

c. How do you rank mild, moderate, and severe? What do those words mean to you? ท่านใช้อะไรเป็นเครื่องบ่งบอกว่าอาการป่วยของท่านน้อย ปานกลาง หรือหนัก, ในความคิดของท่าน น้อย ปานกลาง และหนัก หมายความว่าอย่างไร ທ່ານໃຊ້ຫຍັງເປັນເຄື່ອງບົ່ງບອກອາການເຈັບເປັນຂອງທ່ານ ນ້ອຍ ປານກາງ ຫຼືໜັກ, ໃນຄວາມຄິດຂອງທ່ານ ໜ້ອຍ ປານກາງແລະໜັກ ໝາຍຄວາມວ່າແນວໃດ

9. The next set of questions still relates to the same illness. คำถามชุดต่อไปยังคงเกี่ยวกับอาการป่วยครั้งเดียวกับคำถามก่อนหน้า ຄໍາຖາມຊຸດຕໍ່ໄປຍັງກ່ຽວກັບອາການບໍ່ສະບາຍເຊັ່ນດຽວກັບຄໍາຖາມກ່ອນໜ້ານີ້

For peer review only

Can you please explain the stages of the treatment? I will ask you step-by-step what you did, starting from the moment you first detected the condition. กรุณาอธิบายขั้นตอนการรักษาทุกขั้นตอนระหว่างการป่วยครั้งนี้ เราขอให้ท่านเล่าให้เราฟังทีละขั้นตอนตั้งแต่ท่านเริ่มรู้สึกมีอาการป่วย ກະລຸນາອະທິບາຍຂັ້ນຕອນການປິ່ນປົວທຸກຂັ້ນຕອນລະຫວ່າງການເຈັບເປັນຄັ້ງນີ້ ເຮົາຂໍໃຫ້ທ່ານເລົ່າໃຫ້ເຮົາຟັງເທື່ອລະຂັ້ນຕອນຕັ້ງແຕ່ຕອນທີ່ທ່ານເລີ່ມມີອາການບໍ່ສະບາຍ

[*ask respondent what he or she did first, then code answer and continue. After each column, ask: "What did you do next?" Only one activity per step. If e.g. medical treatment and then home care, first step is medical treatment, second step is home care. Repeat until respondent was cured or today otherwise.*] [[illegible]] *[ຖາມຄຳຖາມກ່ຽວກັບຂັ້ນຕອນການປິ່ນປົວຂັ້ນທຳອິດ [illegible] ຖາມຄຳຖາມຕໍ່ໄປ ຫຼັງຈາກຂຽນຂໍ້ມູນໃນແຕ່ລະຫ້ອງ ໃຫ້ຖາມ [illegible]ທ່ານເຮັດຫຍັງຕໍ່ໄປຫຼັງຈາກນັ້ນ [illegible]ຂຽນຂໍ້ມູນຂອງການປິ່ນປົວແຕ່ລະຊະນິດຕໍ່ໜຶ່ງຂັ້ນຕອນເທົ່ານັ້ນ ຕົວຢ່າງ: [illegible]ເຂົ້າພົບໝໍແລ້ວປິ່ນປົວອາການຢູ່ເຮືອນ [illegible]ໃຫ້ຂຽນຂັ້ນທີ່ 1 ເຂົ້າພົບໝໍ ແລະຂັ້ນທີ່ 2 ປົວອາການຢູ່ເຮືອນ ບັນທຶກຂັ້ນຕອນທຸກຂັ້ນຕອນແບບນີ້ຈົນເຖິງຂັ້ນທີ່ດີຈາກອາການຫຼືຈົນຮອດມື້ເກັບຂໍ້ມູນ]*

	Step 1 (detection)<br>ขั้นที่ 1 (เมื่อเริ่มรู้สึกป่วย)<br>ຂັ້ນທີ່ 1 (ເມື່ອເລີ່ມບໍ່ສະບາຍ)	Step 2<br>ขั้นที่ 2<br>ຂັ້ນທີ່ 2	[*continue until Step x when discomfort was cured or until today*] [[illegible]] *[ບັນທຶກຂັ້ນຕອນທຸກຂັ້ນຕອນໃນຕາຕະລາງນີ້ຕັ້ງແຕ່ເລີ່ມເຈັບເປັນຈົນເຖິງຫາຍດີຫຼືຈົນຮອດມື້ເກັບຂໍ້ມູນ]*
a) What kind of help or treatment did you get at this stage? ท่านมีการรักษาในขั้นตอนนี้อย่างไร ທ່ານມີການປິ່ນປົວໃນຂັ້ນຕອນນີ້ແບບໃດ<br><br>[*if unsure, specify*] [[illegible]] *[ຖ້າບໍ່ໝັ້ນໃຈໃຫ້ຂຽນລາຍລະອຽດໃນໝວດ [illegible]ອື່ນໆ]*	Ignored /did nothing ละเลย/ไม่ทำอะไร ບໍ່ສົນໃຈ/ບໍ່ເຮັດຫຍັງເລີຍ. 1<br>Self-care (sleep, rest, medicine at home) รักษาตัวเอง (นอนหลับ, พักผ่อน, ใช้ยาที่มีที่บ้าน) ປົວຕົນເອງ (ນອນ, ພັກຜ່ອນ, ໃຊ້ຢາທີ່ມີຢູ່ເຮືອນ) ....2<br>Care from family and friends (full-time) ได้รับการรักษาจากครอบครัวหรือเพื่อน (ตลอดเวลา) ໄດ້ຮັບການປິ່ນປົວຈາກຄອບຄົວຫຼືໝູ່ (ຕະຫຼອດເວລາ)....................3<br>Treated/consulted at a pharmacist รับการรักษา/คำปรึกษาจากเภสัชกร ຮັບການປິ່ນປົວ/ຄຳປຶກສາຈາກຜູ້ຂາຍຢາ....................4<br>Treated/consulted at shop selling drugs รับการรักษา/คำปรึกษาจากร้านขายของชำหรือร้านค้าทั่วไปที่มีการจำหน่ายยา ຮັບການປິ່ນປົວ/ຄຳປຶກສາຈາກຮ້ານທົ່ວໄປທີ່ມີການຂາຍຢາ ....5<br>Treated/consulted at priv. clinic/hospital รับการรักษา/คำปรึกษาจากคลินิก/โรงพยาบาลเอกชน ຮັບການປິ່ນປົວ/ຄຳປຶກສາຈາກຈາກຄລີນິກ/ໂຮງໝໍເອກະຊົນ ....6<br>Treated/consulted at primary care unit รับการรักษา/คำปรึกษาจากสถานีอนามัยของรัฐ ຮັບການປິ່ນປົວ/ຄຳປຶກສາຈາກສະຖານີອານາໄມຂອງລັດ....................7<br>Treated/consulted at a gvt. Hospital รับการรักษา/คำปรึกษาจากโรงพยาบาลของรัฐ ຮັບການປິ່ນປົວ/ຄຳປຶກສາຈາກຈາກໂຮງໝໍຂອງລັດ ...8<br>Other (specify) อื่นๆ (โปรดระบุ) ອື່ນໆ (ກະລຸນາບອກ) _ 9	1<br>2<br>3<br>4<br>5<br>6<br>7<br>8<br>9	1<br>2<br>3<br>4<br>5<br>6<br>7<br>8<br>9

b) Where did this activity take place? ขั้นตอนการรักษานี้เกิดขึ้นที่ไหน ຂັ້ນຕອນການປິ່ນປົວນີ້ເກີດຂຶ້ນຢູ່ໃສ	At home ที่บ้าน ຢູ່ເຮືອນ....1<br>Less than 10 min. from home น้อยกว่า 10 นาทีจากบ้าน ນ້ອຍກ່ວາ 10 ນາທີຈາກເຮືອນ....2<br>10 to 29 min. 10 ถึง 29 นาทีจากบ้าน 10 ຫາ 29 ນາທີຈາກເຮືອນ....3<br>30 to 59 min. 30 ถึง 59 นาทีจากบ้าน 30 ຫາ 59 ນາທີຈາກເຮືອນ....4<br>60 to 119 min. 60 ถึง 119 นาทีจากบ้าน 60 ຫາ 119 ນາທີຈາກເຮືອນ....5<br>2 hours or more from home 2 ชั่วโมงหรือมากกว่านั้น ສອງຊົ່ວໂມງຫຼືຫຼາຍກ່ວາ....6	1<br>2<br>3<br>4<br>5<br>6	1<br>2<br>3<br>4<br>5<br>6
c) How did you get to the place of the activity? ท่านเดินทางไปสถานที่ที่ท่านทำการรักษาในขั้นตอนนี้อย่างไร ທ່ານເດີນທາງໄປສະຖານທີ່ທີ່ ປິ່ນປົວໃນຂັ້ນຕອນນີ້ແນວໃດ<br>*[[illegible] according to prior responses [illegible]][ເລືອກ ຢູ່ເຮືອນ ຖ້າ [illegible] ສອດຄ່ອງ ກັບຄຳຕອບກ່ອນໜ້າຫາກາງວຂ້ອງກັນ]*	At home ที่บ้าน ຢູ່ເຮືອນ....1<br>Walk เดินเท้า ຍ່າງ....2<br>Own bicycle จักรยานของท่าน ລົດຖີບຂອງທ່ານ....3<br>Own motorcycle / Three-wheeler รถจักรยานยนต์/รถสามล้อของท่าน ລົດຈັກ/ລົດສາມລໍ້ຂອງທ່ານ....4<br>Own car / four-wheeler รถยนต์/ขับเคลื่อนสี่ล้อของท่าน ລົດຍົນ/ລົດໃຫຍ່ສີ່ລໍ້ຂອງທ່ານ....5<br>Taxi or other hired ride รถแท็กซี่/รถรับจ้าง ລົດແທັກຊີ/ລົດຮັບຈ້າງ....6<br>Public transport ขนส่งสาธารณะ ລົດຂົນສົ່ງສາທາລະນະ....7<br>Other (specify) อื่นๆ (โปรดระบุ) ອື່ນໆ (ກະລຸນາບອກ) _ 8	1<br>2<br>3<br>4<br>5<br>6<br>7<br>8	1<br>2<br>3<br>4<br>5<br>6<br>7<br>8
d) How long did this stage last? ท่านรักษาในขั้นตอนนี้เป็นระยะเวลาเท่าใด ທ່ານປິ່ນປົວໃນຂັ້ນຕອນນີ້ເປັນໄລຍະເວລາເທົ່າໃດ<br>*[let respondent choose category; if less than one day, code [illegible] day] [illegible]*<br>*[ໃຫ້ຜູ້ໃຫ້ສຳພາດເລືອກໜ່ວຍເວລາເອງ ຖ້ານ້ອຍກວ່າໜຶ່ງມື້ໃຫ້ຂຽນ [illegible] ມື້]*	Duration ระยะเวลา ໄລຍະເວລາ:<br>___ days วัน ມື້<br>___ weeks สัปดาห์ ອາທິດ<br>___ months เดือน ເດືອນ	___ days วัน ມື້<br>___ weeks สัปดาห์ ອາທິດ<br>___ months เดือน ເດືອນ	___ days วัน ມື້<br>___ weeks สัปดาห์ ອາທິດ<br>___ months เดือน ເດືອນ
e) Why did you seek this advice/treatment? เพราะเหตุใดท่านจึงเลือกรักษา/รับคำแนะนำนี้ ເປັນຫຍັງທ່ານຈຶ່ງເລືອກການປິ່ນປົວ/ຮັບຄຳແນະນຳນີ້<br>*[Mark all that apply] [[illegible]]*<br>*[ຕອບໄດ້ຫຼາຍກ່ວາ 1 ຕົວເລືອກ]*	This is the normal choice เป็นสิ่งที่ทำอยู่ปกติ ເປັນແນວປະຕິບັດປົກກະຕິ.... 1<br>Advice from others เป็นคำแนะนำจากคนอื่น ເປັນຄຳແນະນຳຈາກຄົນອື່ນ.... 2<br>Too busy for alternatives ไม่มีตัวเลือกอื่นเพราะไม่ว่าง ບໍ່ມີທາງເລືອກອື່ນເນື່ອງຈາກບໍ່ມີເວລາ.... 3<br>No access to money/transportation มีปัญหาเรื่องเงินหรือการเดินทาง ມີບັນຫາກ່ຽວກັບເງິນຫຼືການເດີນທາງ.... 4<br>Ran out of medicines ยาที่ใช้อยู่หมด ຢາທີ່ໃຊ້ຢູ່ໝົດ.... 5<br>Not feeling better พยายามรักษาด้วยวิธีอื่นแล้วไม่หาย ປິ່ນປົວໂດຍວິທີອື່ນແລ້ວບໍ່ດີຂຶ້ນ.... 6<br>New/more serious symptoms อาการหนักลงกว่าเก่าหรือมีอาการอื่นปรากฏ ອາການໜັກລົງກວ່າເກົ່າຫຼືມີອາການອື່ນປາກົດເພີ່ມຂຶ້ນ.... 7<br>Other (specify) อื่นๆ (โปรดระบุ) ອື່ນໆ (ກະລຸນາບອກ) _ 8	1<br>2<br>3<br>4<br>5<br>6<br>7<br>8	1<br>2<br>3<br>4<br>5<br>6<br>7<br>8

f) Can you please name or describe all the medicines that you received or were prescribed during this step? กรุณาระบุชื่อหรืออธิบายลักษณะของยาทั้งหมดที่ท่านได้รับจากการรักษาขั้นตอนนี้ ກະລຸນາບອກຊື່ຫຼືອະທິບາຍລັກສະນະຂອງຢາທີ່ທ່ານໄດ້ຮັບຈາກການປິ່ນປົວຂັ້ນຕອນນີ້<br>[*leave blank if no medicine received*] [*include medicine stored at home if self-care at home*] [*continue for all medicines received, then complete Questions g to k for each medicine individually*] [illegible] [*ຈື່ຊ່ອງຫວ່າງຖ້າຜູ້ໃຫ້ສຳພາດບໍ່ໄດ້ຮັບຢາຈາກການປິ່ນປົວໃນຂັ້ນຕອນນີ້*] [*ຮວມຢາທີ່ມີເກັບຢູ່ເຮືອນດ້ວຍ ຖ້າຫາກຜູ້ໃຫ້ສຳພາດປົວຕົນເອງຢູ່ເຮືອນ*] [*ບັນທຶກຢາທຸກຊະນິດທີ່ຜູ້ໃຫ້ສຳພາດໄດ້ຮັບ ຈາກນັ້ນຕື່ມຂໍ້ມູນໃສ່ໃນຄຳຖາມ g ເຖິງ k ສຳລັບຢາແຕ່ລະຊະນິດ*]	Medicine 1 ยาชนิดที่ 1 ຢາຊະນິດທີ່ 1:<br>Name/description ชื่อ/คำอธิบาย ຊື່/ຄຳອະທິບາຍ:<br>______________<br>Medicine 2 ยาชนิดที่ 2 ຢາຊະນິດທີ່ 2:<br>Name/description ชื่อ/คำอธิบาย ຊື່/ຄຳອະທິບາຍ:<br>______________<br>Medicine 3 ยาชนิดที่ 3 ຢາຊະນິດທີ່ 3:<br>Name/description ชื่อ/คำอธิบาย ຊື່/ຄຳອະທິບາຍ:<br>______________	Medicine 1 ยาชนิดที่ 1 ຢາຊະນິດທີ່ 1<br>Medicine 2 ยาชนิดที่ 2 ຢາຊະນິດທີ່ 2<br>Medicine 3 ยาชนิดที่ 3 ຢາຊະນິດທີ່ 3	Medicine 1 ยาชนิดที่ 1 ຢາຊະນິດທີ່ 1<br>Medicine 2 ยาชนิดที่ 2 ຢາຊະນິດທີ່ 2<br>Medicine 3 ยาชนิดที่ 3 ຢາຊະນິດທີ່ 3
[*for each medicine individually*] [illegible] [*ສຳລັບຢາແຕ່ລະຊະນິດ*]<br>g) For how long did you take the medicine? ท่านใช้ยาชนิดนี้อยู่นานเท่าใด ທ່ານໃຊ້ຢາຊະນິດນີ້ດົນປານໃດ<br>[*let respondent choose category; if more than one repeated episode, indicate total duration*] [illegible] [*ໃຫ້ຜູ້ໃຫ້ສຳພາດເລືອກໜ່ວຍເວລາເອງ ຖ້າມີການໃຊ້ຕົວຢາຊະນິດດຽວກັນຫຼາຍເທື່ອໃຫ້ຜູ້ສຳພາດລວມໄລຍະເວລາທັງໝົດ*]	Duration ระยะเวลา ໄລຍະເວລາ:<br>___ days วัน ມື້<br>___ weeks สัปดาห์ ອາທິດ<br>___ months เดือน ເດືອນ	___ days วัน ມື້<br>___ weeks สัปดาห์ ອາທິດ<br>___ months เดือน ເດືອນ	___ days วัน ມື້<br>___ weeks สัปดาห์ ອາທິດ<br>___ months เดือน ເດືອນ
h) How often per day did you take the medicine? ท่านใช้ยาชนิดนี้วันละกี่ครั้ง ທ່ານໃຊ້ຢາຊະນິດນີ້ມືລະຈັກເທື່ອ<br>[*calculate into daily use according to respondent's chosen frequency*] [illegible] [*ຄິດໄລ່ເປັນຈຳນວນເທື່ອຕໍ່ມື້ຈາກຄຳຕອບຂອງຜູ້ໃຫ້ສຳພາດ*]	Frequency ความถี่ ຄວາມຖີ່: ___ times daily ครั้งต่อวัน ເທື່ອຕໍ່ມື້	___ times daily ครั้งต่อวัน ເທື່ອຕໍ່ມື້	___ times daily ครั้งต่อวัน ເທື່ອຕໍ່ມື້

i) What dosage did you normally take? ท่านใช้ยาชนิดนี้ในปริมาณเท่าใดต่อหนึ่งครั้ง ທ່ານໃຊ້ຢາຊະນິດນີ້ປະລິມານເທົ່າໃດຕໍ່ໜຶ່ງເທື່ອ<br>[*let respondent choose category according to type of medicine*] [⊠ ⊠ ⊠ - ⊠ ⊠ ⊠ ⊠ ⊠ ⊠ r ⊠ ⊠ ⊠ ⊠ ⊠ ⊠ ⊠ ⊠ ⊠ ⊠ ⊠<br>[*ໃຫ້ຜູ້ໃຫ້ສຳພາດເລືອກໜ່ວຍປະລິມານຢາເອງ ໂດຍຂຶ້ນຢູ່ກັບຊະນິດຂອງຢາ*]	Dosage ปริมาณ ຄວາມຖີ່:<br>___ tablets / capsules<br>เม็ด/แคปซูล ເມັດ/ແຄບຊູນ<br>___ drops (for liquid medicine)<br>หยด (สำหรับยาน้ำ) ຢອດ (ສຳລັບຢານ້ຳ)<br>___ spoons (for liquid medicine)<br>ช้อน (สำหรับยาน้ำ) ບ່ວງ (ສຳລັບຢານ້ຳ)<br>per time administered ต่อครั้งที่ใช้ ຕໍ່ເທື່ອທີ່ໃຊ້	___ tablets เม็ด ເມັດ<br>___ drops หยด ຢອດ<br>___ spoons ช้อน ບ່ວງ	___ tablets เม็ด ເມັດ<br>___ drops หยด ຢອດ<br>___ spoons ช้อน ບ່ວງ
j) Did you take the medicine exactly as it was recommended to you by the person who prescribed/sold them? ท่านใช้ยาชนิดนี้ตามคำแนะนำของผู้สั่งจ่าย/จำหน่ายหรือไม่ ທ່ານໃຊ້ຢາຊະນິດນີ້ຕາມຄຳແນະນຳຈາກທ່ານໝໍ/ຜູ້ຂາຍຢາຫຼືບໍ່	Yes ใช่ ແມ່ນ.....1<br>No ไม่ใช่ ບໍ່ແມ່ນ.....2<br>Did not receive advice ไม่ได้รับคำแนะนำ ບໍ່ໄດ້ຮັບຄຳແນະນຳ.....9<br>Don t⊠know ไม่ใช่ ບໍ່ແມ່ນ.....99	1<br>2<br>9<br>99	1<br>2<br>9<br>99
k) Did you finish the medicine? ท่านใช้ยาชนิดนี้จนหมดหรือไม่ ທ່ານໃຊ້ຢາຊະນິດນີ້ຈົນໝົດຫຼືບໍ່	Yes ใช่ ແມ່ນ..... 1<br>No ไม่ใช่ ບໍ່ແມ່ນ..... 2	1<br>2	1<br>2

Have you now recovered from the illness/accident? ท่านฟื้นตัวจากอาการป่วย/อุบัติเหตุแล้วหรือไม่ ທ່ານດີຈາກອາການເຈັບເປັນ/ອຸບັດຕິເຫດແລ້ວບໍ່?	Yes ใช่ ແມ່ນ.....1<br>No ไม่ใช่ ບໍ່ແມ່ນ.....2

Was anybody of your personal relationships involved in providing advice or help during the illness? ท่านได้รับคำปรึกษาหรือความช่วยเหลือในด้านใดๆ ก็ตามจากบุคคลที่เกี่ยวข้องกับท่านระหว่างการป่วยครั้งนี้หรือไม่ ທ່ານໄດ້ຮັບຄຳປຶກສາຫຼືຄວາມຊ່ວຍເຫຼືອໃນດ້ານໃດໆ ກໍ່ຕາມຈາກບຸກຄົນທີ່ກ່ຽວຂ້ອງກັບທ່ານລະຫ່ວາງການເຈັບເປັນຄັ້ງນີ້ຫຼືບໍ່

[*record up to ten names*] [⊠ ⊠ ⊠ ⊠ ⊠ ⊠ ⊠ ⊠ ⊠ ⊠ ⊠ ⊠ ⊠ ⊠ ⊠ ⊠ ⊠ *10* ⊠ ⊠][*ບັນທຶກຊື່ຂອງບຸກຄົນທີ່ມີຄວາມກ່ຽວຂ້ອງກັບທ່ານລະຫ່ວາງການເຈັບປ່ວຍຫຼາຍສຸດ 10 ຄົນ*]

	a) What is the name of the person? ชื่อ ຊື່	b) How is this person related to you? ความสัมพันธ์กับท่าน ຄວາມສຳພັນກັບທ່ານ?<br>[*give examples if respondent is unsure about answer categories*] [⊠ ⊠ ⊠ ⊠ ⊠ o - ⊠ ⊠ ⊠ ⊠ ⊠ r ⊠ ⊠ ⊠ ⊠ ⊠] [*ຖ້າຜູ້ໃຫ້ສຳພາດບໍ່ໝັ້ນໃຈໃນໝວດຄຳຕອບ ໃຫ້ຍົກຕົວຢ່າງຄຳຕອບໄດ້*]	c) What kind of support was provided? ⊠ n ⊠ ⊠ ⊠ n ⊠ ⊠ ⊠ ⊠ ⊠ ທ່ານໄດ້ຮັບການຊ່ວຍເຫຼືອຈາກບຸກຄົນເຫຼົ່ານີ້ແນວໃດ?<br>[*mark all that apply*] [⊠ ⊠ ⊠ ⊠ *1* ⊠ ⊠][*ຕອບໄດ້ຫຼາຍກວ່າ 1 ຕົວເລືອກ*]

For peer review only

Contact 1 คนที่ 1 ຄົນທີ່ 1	Name ชื่อ ຊື່:<br>________	Spouse คู่สมรส ຄູ່ແຕ່ງດອງ ....1<br>Parent บิดามารดา ພໍ່ແມ່ ....2<br>Child บุตร ລູກ...............................3<br>Sibling พี่น้อง ອ້າຍນ້ອງ.......................4<br>Other relative ญาติ ພີ່ນ້ອງອື່ນໆ...........5<br>Neighbour เพื่อนบ้าน ເພື່ອນບ້ານ............6<br>Friend (if not neighbour) เพื่อน (ที่ไม่ใช่เพื่อนบ้าน) ໝູ່ (ທີ່ບໍ່ແມ່ນເພື່ອນບ້ານ)..........7<br>Other villager ชาวบ้านคนอื่นๆ ຊາວບ້ານຄົນອື່ນໆ...8<br>Other (specify) อื่นๆ (โปรดระบุ) ອື່ນໆ (ກະລຸນາບອກ) _ 9	Providing healthcare/attending ให้การรักษา/การดูแล ໃຫ້ການເບິ່ງແຍງ...11<br>Providing advice ให้คำปรึกษา ໃຫ້ຄຳປຶກສາ ....12<br>Providing medicine ให้ยา ໃຫ້ຢາ ....13<br>Lending/granting money ให้หรือให้ยืมเงิน ໃຫ້ຫຼືໃຫ້ຢືມເງິນ...21<br>Transportation/Lending vehicle อำนวยความสะดวกในด้านการเดินทาง/ให้ยืมพาหนะ ສ້າງຄວາມສະດວກດ້ານການເດີນທາງ/ໃຫ້ຢືມຍານພາຫະນະ ....22<br>Contacting family/friends ติดต่อครอบครัว/เพื่อน ຕິດຕໍ່ຄອບຄົວ/ໝູ່ ....23<br>Providing food สนับสนุนอาหาร ສະໜັບສະໜູນອາຫານ ....31<br>Helping with children/housework ช่วยดูแลลูกหลาน/งานบ้าน ຊ່ວຍເບິ່ງແຍງລູກຫລານຫຼືເດັກນ້ອຍ/ວຽກບ້ານ ....32<br>Helping with jobs/agriculture work (feeding animals/tending crops/covering shifts, etc.) ช่วยเหลือเกี่ยวกับอาชีพ/การเกษตร (ช่วยดูแลสัตว์เลี้ยง/งานเพาะปลูก/ทำงานแทน) ຊ່ວຍເຮັດວຽກ/ການກະສິກຳ (ຊ່ວຍເບິ່ງແຍງສັດລ້ຽງ/ເຮັດໄຮ່ເຮັດນາ/ເຮັດວຽກແທນ)...33<br>Other (specify) อื่นๆ (โปรดระบุ) ອື່ນໆ (ກະລຸນາບອກ) _ ..99
Contact 2 คนที่ 2 ຄົນທີ່ 2	Name ชื่อ ຊື່	1 2 3 4 5 6 7 8 9	11 12 13 21 22 23 31 32 33 99
[*continue until Contact x* [illegible] *maximum 10 contacts*] [[illegible]] *[ບັນທຶກບຸກຄົນທີ່ມີຄວາມກ່ຽວຂ້ອງລະຫ່ວາງອາການເຈັບປ່ວຍທຸກຄົນ ບໍ່ຫຼາຍກ່ວາ 10ຄົນ]*	Name ชื่อ ຊື່	1 2 3 4 5 6 7 8 9	11 12 13 21 22 23 31 32 33 99

a. Do you think these questions are too hard? ท่านรู้สึกว่าคำถามเหล่านี้ยากเกินไปหรือไม่ ທ່ານຮູ້ສຶກວ່າຄຳຖາມເຫຼົ່ານີ້ຍາກເກີນໄປຫຼືບໍ່

b. Did you find it tedious to go through the illness in such detail? เป็นเรื่องน่าเบื่อหรือไม่ที่ท่านต้องอธิบายอาการป่วยของท่านโดยละเอียด ເປັນເລື່ອງໜ້າເບື່ອຫຼືບໍ່ທີ່ທ່ານຕ້ອງມາອະທິບາຍອາການບໍ່ສະບາຍຂອງທ່ານຢ່າງລະອຽດ

c. How well do you remember the medicines that you used during that illness? ท่านสามารถจดจำยาที่ท่านใช้ระหว่างการป่วยได้ดีเพียงใด ທ່ານສາມາດຈື່ຢາທີ່ທ່ານໃຊ້ລະຫ່ວາງຕອນເຈັບປ່ວຍ ໄດ້ດີພຽງໃດ

d. Did you think about anything in particular to help you recall the medicines? ที่ท่านนึกถึงอะไรเพื่อเป็นตัวช่วยในการทวนความจำเกี่ยวกับยาเหล่านี้ ທ່ານນຶກເຖິງຫຍັງເປັນຕົວຊ່ວຍໃນການຈື່ກ່ຽວກັບຢາເຫຼົ່ານີ້

e. How do you indicate that the symptoms are cured? Would you stop taking medicines then? What if the doctor’s instruction is to complete the dose? ท่านสามารถบอกได้อย่างไรว่าอาการป่วยของท่านหายแล้ว หากอาการป่วยของท่านหายท่านจะหยุดรับประทานยาทันทีหรือไม่ หากแพทย์แนะนำให้ท่านใช้ยาจนหมดแต่อาการของท่านหายแล้วท่านยังจะปฏิบัติตามคำสั่งแพทย์หรือไม่ ທ່ານສາມາດບອກໄດ້ແນວໃດວ່າອາການເຈັບເປັນຂອງທ່ານດີແລ້ວ ຖ້າອາການເຈັບເປັນຂອງທ່ານດີ ທ່ານຈະຢຸດກິນຢາທັນທີຫຼືບໍ່ ຖ້າໝໍແນະນຳໃຫ້ທ່ານໃຊ້ຢາຈົນໝົດແຕ່ອາການຂອງທ່ານດີແລ້ວທ່ານຍັງຈະປະຕິບັດຕາມຄຳສັ່ງໝໍຫຼືບໍ່

10. The next question deals with medicine. Could you please tell me what is going through your mind while you are answering the question? คำถามต่อไปจะเป็นคำถามเกี่ยวกับยารักษาโรค เราขอให้ท่านบอกเราว่าท่านนึกถึงอะไรระหว่างการตอบคำถามเหล่านี้ ຄຳຖາມຕໍ່ໄປຈະເປັນຄຳຖາມກ່ຽວກັບຢາປົວພະຍາດ ເຮົາຂໍໃຫ້ທ່ານບອກເຮົາວ່າທ່ານນຶກເຖິງຫຍັງລະຫ່ວາງການຕອບຄຳຖາມເຫຼົ່ານີ້

For peer review only - http://bmjopen.bmj.com/site/about/guidelines.xhtml

I would now like to ask you your opinion about medicine. There are no right or wrong answers, I only want to understand what you think. เราอยากทราบความคิดเห็นของท่านเกี่ยวกับการใช้ยา คำถามเหล่านี้ไม่มีคำตอบที่ถูกหรือผิด เราเพียงต้องการรู้ว่าท่านคิดอย่างไร ເຮົາຢາກຮູ້ຄວາມຄິດເຫັນຂອງທ່ານກ່ຽວກັບການໃຊ້ຢາ ຄຳຖາມເຫຼົ່ານີ້ບໍ່ມີຄຳຕອບຖືກຫຼືຜິດ ເຮົາພຽງຕ້ອງການຮູ້ວ່າທ່ານຄິດແນວໃດ

Consider the following medicines ⊠they are commonly referred to as ⊠ntibiotics. ⊠

พิจารณายาเหล่านี้ซึ่งเป็นยาที่ถูกรู้จักในนาม "ยาปฏิชีวนะ" ພິຈາລະນາຢາເຫຼົ່ານີ້ເຊິ່ງເປັນຢາທີ່ຮູ້ຈັກກັນໃນນາມ ⊠ຕ້ານເຊື້ອ ⊠

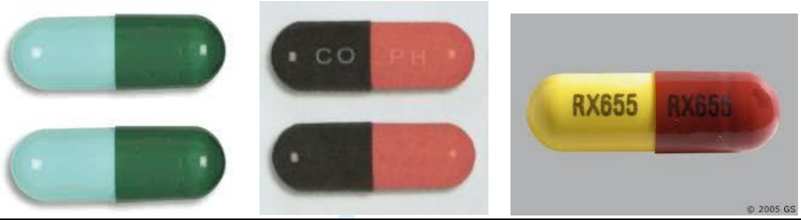

Would you buy this medicine if you think you need it but cannot get it prescribed by a doctor? หากท่านไม่ได้รับยานี้หลังจากเข้าพบแพทย์แม้ท่านรู้สึกว่าท่านควรรับประทานยานี้ ท่านจะซื้อยานี้จากแหล่งอื่นหรือไม่ ຖ້າທ່ານບໍ່ໄດ້ຮັບຢານີ້ຫຼັງຈາກເຂົ້າພົບໝໍເຖິງທ່ານຈະຮູ້ສຶກວ່າທ່ານຄວນກິນຢານີ້ ທ່ານຈະຊື້ຢານີ້ຈາກແຫຼ່ງອື່ນຫຼືບໍ່	Yes ใช่ ແມ່ນ .... 1<br>No ไม่ใช่ ບໍ່ແມ່ນ.................................... 2<br>I don t⊠know ไม่ทราบ ບໍ່ຮູ້ 99
Would you try other remedies such as sponge bath, herbs, or paracetamol before using this medicine? ท่านจะลองใช้วิธีการรักษาอื่น เช่น เช็ดตัว สมุนไพร หรือ พาราเซตามอลก่อนใช้ยานี้หรือไม่ ທ່ານຈະພະຍາຍາມປິ່ນປົວໂດຍວິທີການອື່ນ ເຊັ່ນ ເຊັດຕົວ ຢາຮາກໄມ້ ຫຼືພາຣາເຊຕາມອນກ່ອນໃຊ້ຢານີ້ຫຼືບໍ່	Yes ใช่ ແມ່ນ .... 1<br>No ไม่ใช่ ບໍ່ແມ່ນ.................................... 2<br>I don t⊠know ไม่ทราบ ບໍ່ຮູ້ 99
If you had to take this medicine and your symptoms disappear after 2 days, would you finish a complete course of 7 days or stop taking it immediately? หากท่านจำเป็นต้องรับประทานยานี้และอาการของท่านดีขึ้นภายใน 2 วัน ท่านจะรับประทานยาต่อจนครบ 7 วัน หรือหยุดรับประทานทันที ຖ້າຫາກທ່ານມີຄວາມຈຳເປັນຕ້ອງກິນຢານີ້ເພື່ອການປິ່ນປົວ ແລະອາການຂອງທ່ານດີຂຶ້ນພາຍໃນສອງມື້ ທ່ານຈະກິນຢາຕໍ່ຈົນສຸດ 7 ມື້ ຫຼືຢຸດກິນທັນທີ	Finish course รับประทานต่อจนหมด ກິນຢາຕໍ່ຈົນສຸດ .... 1<br>Stop taking หยุดรับประทาน ຢຸດກິນທັນທີ........ 2<br>I don t⊠know ไม่ทราบ ບໍ່ຮູ້ 99
Do you think the way other people use this medicine affects its efficacy on you? ท่านคิดว่าการใช้ยานี้ของคนอื่นมีผลกระทบต่อผลลัพธ์ของการใช้ยานี้ของท่านหรือไม่ ທ່ານຄິດວ່າການໃຊ້ຢານີ້ຂອງຄົນອື່ນມີຜົນກະທົບກັບສະມັດຖະພາບຂອງການໃຊ້ຢານີ້ຂອງທ່ານຫຼືບໍ່	Yes ใช่ ແມ່ນ .... 1<br>No ไม่ใช่ ບໍ່ແມ່ນ.................................... 2<br>I don t⊠know ไม่ทราบ ບໍ່ຮູ້ 99

a. As I have said before, these survey questions are not intended to measure your knowledge. Did you feel tested or pressured being asked these questions at all? เราได้บอกท่านข้างต้นเกี่ยวกับคำถามเหล่านี้ที่ไม่ได้ถูกออกแบบเพื่อเป็นข้อสอบวัดความรู้ ท่านรู้สึกถูกทดสอบหรือกดดันในการตอบคำถามเหล่านี้หรือไม่ ເຮົາໄດ້ບອກທ່ານຂັ້ນຕົ້ນກ່ຽວກັບຄຳຖາມເຫຼົ່ານີ້ທີ່ບໍ່ໄດ້ອອກແບບເພື່ອເປັນບົດເສັງວັດແທກຄວາມຮູ້ ທ່ານຮູ້ສຶກຖືກທົດສອບຫຼືກົດດັນໃນການຕອບຄຳຖາມເຫຼົ່ານີ້ຫຼືບໍ່

b. Have you heard of antibiotics? ท่านเคยได้ยินเกี่ยวกับยาปฏิชีวนะหรือไม่ ທ່ານເຄີຍໄດ້ຍິນກ່ຽວກັບຢາຕ້ານເຊື້ອຫຼືບໍ່

c. Other than the term "antibiotics," how do you normally call these medicines? นอกจากคำว่า "ยาปฏิชีวนะ" ท่านเรียกยาชนิดนี้ได้ว่าอะไรอีกบ้าง ນອກຈາກຄຳວ່າ ຢາຕ້ານເຊື້ອ ທ່ານເອີ້ນຢາຊະນິດນີ້ໄດ້ວ່າຫຍັງອີກແດ່

d. Do you think other villagers will associate the pictures with other types of medicine? ท่านคิดว่าชาวบ้านคนอื่นๆ จะนึกถึงยาชนิดอื่นหรือไม่เมื่อเห็นภาพเหล่านี้ ທ່ານຄິດວ່າຊາວບ້ານຄົນອື່ນໆ ຈະນຶກເຖິງຢາຊະນິດອື່ນຫຼືບໍ່ເມື່ອເຫັນພາບເຫຼົ່ານີ້

For peer review only - http://bmjopen.bmj.com/site/about/guidelines.xhtml

Part V – Household-Related Survey Questions ส่วนที่ 5 – คำถามเกี่ยวกับครัวเรือน ສ່ວນທີ່ 5 - ຄຳຖາມກ່ຽວກັບຄົວເຮືອນ

Thank you. We now come to the last part. ขอบคุณครับ/ค่ะ ส่วนต่อไปเป็นส่วนสุดท้ายของการสัมภาษณ์ ຂອບໃຈ ສ່ວນຕໍ່ເປັນເປັນສ່ວນສຸດທ້າຍຂອງການສຳພາດ

11. The following questions relate to your household. คำถามต่อไปนี้เป็นคำถามเกี่ยวกับครัวเรือนของท่าน ຄຳຖາມຕໍ່ໄປນີ້ເປັນຄຳຖາມກ່ຽວກັບຄົວເຮືອນຂອງທ່ານ

I will now ask you for some items in your household. Please tell me ต่อไปเราจะถามท่านเกี่ยวกับสิ่งของที่ท่านครอบครองในครัวเรือน ຕໍ່ໄປເຮົາຈະຖາມກ່ຽວກັບສິ່ງຂອງທີ່ທ່ານຄອບຄອງໃນຄົວເຮືອນ		Number of items in household นวนของสิ่งของที่มีภายในครัวเรือน ຈຳນວນຂອງສິ່ງຂອງທີ່ມີພາຍໃນຄົວເຮືອນ:
	Have you got a *functioning* **radio** in your household? If so, how many? ท่านมี**วิทยุ**ที่ใช้งานได้อยู่ในครัวเรือนของท่านหรือไม่ ถ้ามี กี่เครื่อง ທ່ານມີ**ວິທະຍຸ**ທີ່ໃຊ້ງານໄດ້ໃນຄົວເຮືອນຂອງທ່ານຫຼືບໍ່ ຖ້າມີ ຈັກອັນ	________
	Have you got a *functioning* **TV** in your household? If so, how many? ท่านมี**โทรทัศน์**ที่ใช้งานได้อยู่ในครัวเรือนของท่านหรือไม่ ถ้ามี กี่เครื่อง ທ່ານມີ**ທໍລະທັດ**ທີ່ໃຊ້ງານໄດ້ຢູ່ໃນຄົວເຮືອນຂອງທ່ານຫຼືບໍ່ ຖ້າມີ ຈັກອັນ	________
	Have you got a *functioning* **rice cooker** in your household? If so, how many? ท่านมี**หม้อหุงข้าว**ที่ใช้งานได้อยู่ในครัวเรือนของท่านหรือไม่ ถ้ามี กี่เครื่อง ທ່ານມີ**ໝໍ້ຫຸງເຂົ້າ**ທີ່ໃຊ້ງານໄດ້ຢູ່ໃນຄົວເຮືອນຂອງທ່ານຫຼືບໍ່ ຖ້າມີ ຈັກອັນ	________
	Have you got a *functioning* **landline telephone** in your household? If so, how many? ท่านมี**โทรศัพท์บ้าน**ที่ใช้งานได้อยู่ในครัวเรือนของท่านหรือไม่ ถ้ามี กี่เครื่อง ທ່ານມີ**ໂທລະສັບຕັ້ງໂຕະ**ທີ່ໃຊ້ງານໄດ້ຢູ່ໃນຄົວເຮືອນຂອງທ່ານຫຼືບໍ່ ຖ້າມີ ຈັກອັນ	________
	Have you got a *functioning* **mobile phone** in your household? If so, how many? ท่านมี**โทรศัพท์มือถือ**ที่ใช้งานได้อยู่ในครัวเรือนของท่านหรือไม่ ถ้ามี กี่เครื่อง ທ່ານມີ**ໂທລະສັບມືຖື**ທີ່ໃຊ້ງານໄດ້ຢູ່ໃນຄົວເຮືອນຂອງທ່ານຫຼືບໍ່ ຖ້າມີ ຈັກອັນ	________
	Have you got a *functioning* **computer** in your household? If so, how many? ท่านมี**คอมพิวเตอร์**ที่ใช้งานได้อยู่ในครัวเรือนของท่านหรือไม่ ถ้ามี กี่เครื่อง ທ່ານມີ**ຄອມພິວເຕີ**ທີ່ໃຊ້ງານໄດ້ຢູ່ໃນຄົວເຮືອນຂອງທ່ານຫຼືບໍ່ ຖ້າມີ ຈັກອັນ	________
	Have you got a *functioning* **bicycle** in your household? If so, how many? ท่านมี**รถจักรยาน**ที่ใช้งานได้อยู่ในครัวเรือนของท่านหรือไม่ ถ้ามี กี่คัน ທ່ານມີ**ລົດຖີບ**ທີ່ໃຊ້ງານໄດ້ຢູ່ໃນຄົວເຮືອນຂອງທ່ານຫຼືບໍ່ ຖ້າມີ ຈັກຄັນ	________
	Have you got a *functioning* **scooter, motorcycle, or tricycle** in your household? If so, how many? ท่านมี**จักรยานยนต์หรือรถสามล้อ**ที่ใช้งานได้อยู่ในครัวเรือนของท่านหรือไม่ ถ้ามี กี่คัน ທ່ານມີ**ລົດຈັກຫຼືລົດສາມລໍ້**ທີ່ໃຊ້ງານໄດ້ຢູ່ໃນຄົວເຮືອນຂອງທ່ານຫຼືບໍ່ ຖ້າມີ ຈັກຄັນ	________
	Have you got a *functioning* **car or truck** in your household? If so, how many? ท่านมี**รถยนต์ รถกระบะ หรือรถบรรทุก**ที่ใช้งานได้อยู่ในครัวเรือนของท่านหรือไม่ ถ้ามี กี่คัน ທ່ານມີ**ລົດໃຫຍ່ ລົດກະບະ ຫຼືລົດບັນທຸກ**ທີ່ໃຊ້ງານໄດ້ຢູ່ໃນຄົວເຮືອນຂອງທ່ານຫຼືບໍ່ ຖ້າມີ ຈັກຄັນ	________
	Have you got a *functioning* **tractor** in your household? If so, how many? ท่านมี**รถแทรกเตอร์หรือรถไถนา**ที่ใช้งานได้อยู่ในครัวเรือนของท่านหรือไม่ ถ้ามี กี่คัน ທ່ານມີ**ລົດໄຖນາ**ທີ່ໃຊ້ງານໄດ້ຢູ່ໃນຄົວເຮືອນຂອງທ່ານຫຼືບໍ່ ຖ້າມີ ຈັກຄັນ	________
	Have you got a *functioning* **refrigerator or freezer** in your household? If so, how many? ท่านมี**ตู้เย็นหรือตู้แช่เข็ง**ที่ใช้งานได้อยู่ในครัวเรือนของท่านหรือไม่ ถ้ามี กี่ตู้ ທ່ານມີ**ຕູ້ເຢັນຫຼືຕູ້ແຊ່ແຂງ**ທີ່ໃຊ້ງານໄດ້ຢູ່ໃນຄົວເຮືອນຂອງທ່ານຫຼືບໍ່ ຖ້າມີ ຈັກຕູ້	________

a. Do you feel comfortable (or uncomfortable) recalling these points for your household? ท่านรู้สึกสบายใจ (หรืออึดอัดใจ) ในการนึกถึงข้าวของเครื่องใช้เหล่านี้ในครัวเรือนของท่านหรือไม่ ທ່ານຮູ້ສຶກສະບາຍໃຈ (ຫຼືອຶດອັດໃຈ) ໃນການນຶກເຖິງເຄື່ອງໃຊ້ເຫຼົ່ານີ້ໃນຄົວເຮືອນຂອງທ່ານຫຼືບໍ່

b. Are there some common household items that you normally see in your village? ข้าวของเครื่องใช้ที่เรากล่าวมาข้างต้นเป็นสิ่งที่คนในหมู่บ้านนี้ใช้กันทั่วไปหรือไม่ ເຄື່ອງໃຊ້ທີ່ເຮົາກ່າວມາໃນຂັ້ນຕົ້ນເປັນສິ່ງທີ່ຄົນໃນໝູ່ບ້ານນີ້ໃຊ້ທົ່ວໄປຫຼືບໍ່

c. How would you distinguish whether somebody in your village is wealthy or not?

For peer review only

ในหมู่บ้านของท่าน ท่านสามารถแยกได้อย่างไรว่าใครรวยหรือจน ໃນໝູ່ບ້ານຂອງທ່ານ ທ່ານສາມາດແຍກໄດ້ແນວໃດວ່າໃຜລວຍຫຼືທຸກ

d. If you had to group these houses into 2 categories, rich and poor, how would you do that? [Sample pictures will be provided] หากท่านต้องจัดกลุ่มบ้านในภาพเหล่านี้ออกเป็นสองกลุ่ม ได้แก่กลุ่มรวยและกลุ่มจน ท่านจะจัดอย่างไร [แสดงบัตรภาพให้ผู้ให้สัมภาษณ์จัดกลุ่ม] ຫາກທ່ານຕ້ອງຈັດກຸ່ມເຮືອນໃນຮູບເຫຼົ່ານີ້ອອກເປັນສອງກຸ່ມໄດ້ແກ່ກຸ່ມລວຍແລະກຸ່ມທຸກ ທ່ານຈະຈັດແນວໃດ [ສະແດງບັດພາບໃຫ້ຜູ້ສຳພາດຈັດກຸ່ມ]

e. What kinds of "meals" did you have in mind when you answered the question? ตอนที่ท่านตอบคำถามเราเรื่องมื้ออาหารที่ท่านรับประทานสัปดาห์ที่แล้ว ท่านจินตนาการภาพถึงมื้ออาหารลักษณะใด ຕອນທ່ານຕອບຄຳຖາມເຮົາເລື່ອງຄາບອາຫານທີ່ທ່ານກິນໃນອາທິດທີ່ແລ້ວ ທ່ານຈິນຕະນາການການພາບເຖິງຄາບອາຫານລັກຊະນະໃດ

12. And these are the last two questions. I would like to ask you once more to share your thoughts as you answer these questions. และคำถามสองคำถามสุดท้าย เราต้องการขอให้ท่านเล่าให้เราฟังอีกครั้งว่าท่านคิดอะไรบ้างระหว่างการตอบคำถามต่อไปนี้ ແລະຄຳຖາມສອງຄຳຖາມສຸດທ້າຍ ເຮົາຂໍໃຫ້ທ່ານເລົ່າໃຫ້ເຮົາຟັງອີກເທື່ອໜຶ່ງວ່າທ່ານຄິດຫຍັງແດ່ລະຫວ່າງການຕອບຄຳຖາມເຫຼົ່ານີ້

And finally the last two questions: Have you heard about drug resistance? คำถามสองข้อสุดท้าย: ท่านเคยได้ยินเกี่ยวกับการดื้อยาหรือไม่ ຄຳຖາມສອງຂໍ້ສຸດທ້າຍ: ທ່ານເຄີຍໄດ້ຍິນກ່ຽວກັບການດື້ຢາຫຼືບໍ່	Yes เคย ເຄີຍ.. 1<br>No ไม่เคย ບໍ່ເຄີຍ 2
What do you think is drug resistance? ท่านคิดว่าการดื้อยาคืออะไร ທ່ານຄິດວ່າການດື້ຢາແມ່ນຫຍັງ	Answer relates to antibiotic or antimicrobial resistance คำตอบสอดคล้องกับการดื้อยาต้านจุลชีพ ຄຳຕອບສອດຄ່ອງກັບການດື້ຢາຕ້ານຈຸລະຊີບ...1<br>Answer relates to resistance to other types of drugs คำตอบสอดคล้องกับการดื้อยาชนิดอื่นๆ ຄຳຕອບສອງຄ່ອງກັບການດື້ຢາຊະນິດອື່ນໆ ....2<br>Answer does not relate to drug resistance คำตอบไม่สอดคล้องกับการดื้อยา ຄຳຕອບບໍ່ສອດຄ່ອງກັບການດື້ຢາ..3<br>☒on t☒know ☒ไม่ทราบ ບໍ່ຮູ້ 99

a. What your intuitive understanding of "drug resistance?" เมื่อพูดถึง "การดื้อยา" ท่านเข้าใจความหมายของคำคำนี้อย่างไร ເມື່ອເວົ້າເຖິງ ☒ການດື້ຢາ ☒ທ່ານເຂົ້າໃຈຄວາມໝາຍຂອງຄຳນີ້ແນວໃດ

b. Do you think other villagers will translate the meaning of "drug resistance" differently from you? ท่านคิดว่าชาวบ้านคนอื่นๆ จะแปลความหมายของคำว่า "ดื้อยา" แตกต่างจากท่านหรือไม่ ທ່ານຄິດວ່າຊາວບ້ານຄົນອື່ນ ຈະແປຄວາມໝາຍຂອງຄຳວ່າ ☒ດື້ຢາ ☒ແຕກຕ່າງຈາກທ່ານຫຼືບໍ່

c. Did you feel pressured answering these questions? ท่านรู้สึกกดดันในการตอบคำถามนี้หรือไม่ ທ່ານຮູ້ສຶກກົດດັນໃນການຕອບຄຳຖາມເຫຼົ່ານີ້ຫຼືບໍ່

We are coming to the end of our interview. Thank you so much for sharing your views with me. Your responses have been very helpful. *การสัมภาษณ์นี้สิ้นสุดลงแล้ว คำตอบที่ท่านให้กับเราเป็นประโยชน์กับการวิจัยของเราอย่างมาก ขอบคุณสำหรับความคิดเห็นที่ท่านแบ่งปันกับเรา* ການສຳພາດນີ້ສິ້ນສຸດແລ້ວ ຄຳຕອບທີ່ທ່ານໃຫ້ກັບເຮົາຈະເປັນປະໂຫຍດກັບການຄົ້ນຄ້ວາຂອງເຮົາຫຼາຍ ຂອບໃຈສຳລັບຄຳຄິດເຫັນທີ່ທ່ານແບ່ງປັນກັບເຮົາ

For peer review only

For peer review only - http://bmjopen.bmj.com/site/about/guidelines.xhtml

No	Topic	Item	Check	Comment
1a	Title & Abstract	(*a*) Indicate the study's design with a commonly used term in the title or the abstract	☑	Page 1
1b	Title & Abstract	(*b*) Provide in the abstract an informative and balanced summary of what was done and what was found	☑	Pages 2-3
**Introduction**				
2	Background/ rationale	Explain the scientific background and rationale for the investigation being reported	☑	See introduction, esp. page 4
3	Objectives	State specific objectives, including any prespecified hypotheses	☑	See introduction, page 4
**Methods**				
4	Study design	Present key elements of study design early in the paper	☑	Research design at beginning of methods section, pages 5-6
5	Setting	Describe the setting, locations, and relevant dates, including periods of recruitment, exposure, follow-up, and data collection	☑	See research design section, pages 5-6; exposure and follow-up not applicable
6	Participants	Cross-sectional study—Give the eligibility criteria, and the sources and methods of selection of participants	☑	See research design and study population sections, pages 5-6
7	Variables	Clearly define all outcomes, exposures, predictors, potential confounders, and effect modifiers. Give diagnostic criteria, if applicable	☑	Key outcomes described in data collection section, pages 7-8; All variables described in Appendix Table A2; diagnostic criteria not applicable
8	Data sources/ measurement	For each variable of interest, give sources of data and details of methods of assessment (measurement). Describe comparability of assessment methods if there is more than one group	☑	All variables described in Appendix Table A2, data collection and translation process for the two sites described in data collection section, page 7
9	Bias	Describe any efforts to address potential sources of bias	☑	Recall biases mitigated through elicitation of behavioural sequences (pages 15-16)
10	Study size	Explain how the study size was arrived at	☑	See research design section, pages 5-6
11	Quantitative variables	Explain how quantitative variables were handled in the analyses. If applicable, describe which groupings were chosen and why	☑	All variables described in Appendix Table A2 (incl. variable construction)
12a	Statistical methods	(*a*) Describe all statistical methods, including those used to control for confounding	☑	Data analysis section, pages 8-9
12b	Statistical methods	(*b*) Describe any methods used to examine subgroups and interactions	☑	Data analysis section, pages 8-9
12c	Statistical methods	(*c*) Explain how missing data were addressed	☑	Not applicable (data complete; sub-group analysis of illness episodes indicated in data analysis section, page 9)
12d	Statistical methods	Cross-sectional study—If applicable, describe analytical methods taking account of sampling strategy	☑	Page 8: SVY suite of commands in Stata 15
12e	Statistical methods	Describe any sensitivity analyses	☑	Inclusion of "confirmed" and "potential" antibiotic use, data collection section, pages 7-8
**Results**				
13a	Participants	(a) Report numbers of individuals at each stage of study—eg numbers potentially eligible, examined for eligibility, confirmed eligible, included in the study, completing follow-up, and analysed	☑	See flow diagram of sampling strategy in Figure 1, page 6
13b	Participants	(b) Give reasons for non-participation at each stage	☑	Research design section: Non-availability of random selection, information on random replacement procedures in pages 5-6 and selection data in Figure 1
13c	Participants	(c) Consider use of a flow diagram	☑	See flow diagram of sampling strategy in Figure 1, page 6
14a	Descriptive data	(a) Give characteristics of study participants (eg demographic, clinical, social) and information on exposures and potential confounders	☑	Results section, Table 1, page 11
14b	Descriptive data	(b) Indicate number of participants with missing data for each variable of interest	☑	Not applicable (data complete)
15	Outcome data	Cross-sectional study—Report numbers of outcome events or summary measures	☑	Results section, Table 1, page 11
16a	Main results	(*a*) Give unadjusted estimates and, if applicable, confounder-adjusted estimates and their precision (eg, 95% confidence interval). Make clear which confounders were adjusted for and why they were included	☑	Not applicable: descriptive population estimates of knowledge, attitudes, behaviours
16b	Main results	(*b*) Report category boundaries when continuous variables were categorized	☑	Not applicable
16c	Main results	(*c*) If relevant, consider translating estimates of relative risk into absolute risk for a meaningful time period	☑	Not applicable
17	Other analyses	Report other analyses done—eg analyses of subgroups and interactions, and sensitivity analyses	☑	Throughout results section, pages 9-14, e.g. sensitivity analysis of antibiotic classifications in Footnote iv
**Discussion**				
18	Key results	Summarise key results with reference to study objectives	☑	Discussion/conclusion section, pages 14-15
19	Limitations	Discuss limitations of the study, taking into account sources of potential bias or imprecision. Discuss both direction and magnitude of any potential bias	☑	Discussion/conclusion section, pages 15-16
20	Interpretation	Give a cautious overall interpretation of results considering objectives, limitations, multiplicity of analyses, results from similar studies, and other relevant evidence	☑	Discussion/conclusion section, lines 16-17
21	Generalisability	Discuss the generalisability (external validity) of the study results	☑	Discussion/conclusion section, page 16
**Other information**				
22	Funding	Give the source of funding and the role of the funders for the present study and, if applicable, for the original study on which the present article is based	☑	See funding declaration, page 19

BMJ Open

Antibiotic Knowledge, Attitudes, and Practices: New Insights from Cross-Sectional Rural Health Behaviour Surveys in Low- and Middle-Income Southeast Asia

Journal:	*BMJ Open*
Manuscript ID	bmjopen-2018-028224.R1
Article Type:	Research
Date Submitted by the Author:	20-May-2019
Complete List of Authors:	Haenssgen, Marco; University of Oxford Centre for Tropical Medicine, Center for Tropical Medicine and Global Health; University of Warwick, School of Cross Faculty Studies<br>Charoenboon, Nutcha; Mahidol Oxford Tropical Medicine Research Unit<br>Zanello, Giacomo; University of Reading, School of Agriculture, Policy and Development; Leverhulme Centre for Integrative Research on Agriculture and Health<br>Mayxay, Mayfong; Lao-Oxford-Mahosot Hospital-Wellcome Trust Research Unit; Faculty of Postgraduate Studies, University of Health Sciences<br>Reed-Tsochas, Felix; University of Oxford Said Business School, Said Business School; University of Oxford, Oxford Martin School<br>Lubell, Yoel ; University of Oxford Centre for Tropical Medicine, Center for Tropical Medicine and Global Health; Mahidol Oxford Tropical Medicine Research Unit<br>Wertheim, Heiman; Radbuod University, Medical Microbiology Department; Oxford University Clinical Research Unit<br>Lienert, Jeffrey; University of Oxford Said Business School, ; National Human Genome Research Institute,<br>Xayavong, Thipphaphone; University for Peace, Department of Peace and Conflict Studies; Ateneo de Manila University, Department of Political Science<br>Khine Zaw, Yuzana; London School of Hygiene and Tropical Medicine, Department of Global Health and Development<br>Thepkhamkong, Amphayvone; Lao-Oxford-Mahosot Hospital-Wellcome Trust Research Unit<br>Sithongdeng, Nicksan; Lao-Oxford-Mahosot Hospital-Wellcome Trust Research Unit<br>Khamsoukthavong, Nid; Lao-Oxford-Mahosot Hospital-Wellcome Trust Research Unit<br>Phanthavong, Chanthasone; Lao-Oxford-Mahosot Hospital-Wellcome Trust Research Unit<br>Boualaiseng, Somsanith; Lao-Oxford-Mahosot Hospital-Wellcome Trust Research Unit<br>Vongsavang, Souksakhone; Lao-Oxford-Mahosot Hospital-Wellcome Trust Research Unit<br>Wibunjak, Kanokporn; Mahidol Oxford Tropical Medicine Research Unit<br>Chai-in, Poowadon; Mahidol Oxford Tropical Medicine Research Unit,<br>Thavethanutthanawin, Patthanan; Mahidol Oxford Tropical Medicine

Research Unit
Althaus, Thomas; University of Oxford Centre for Tropical Medicine, Center for Tropical Medicine and Global Health; Mahidol Oxford Tropical Medicine Research Unit
Greer, Rachel; Mahidol Oxford Tropical Medicine Research Unit, Faculty of Tropical Medicine Mahidol University; Chiangrai Regional Hospital, Chiangrai Clinical Research Unit
Nedsuwan, Supalert; Chiangrai Regional Hospital, Primary Care Department
Wangrangsimakul, Tri; Mahidol Oxford Tropical Medicine Research Unit; Chiangrai Regional Hospital, Chiangrai Clinical Research Unit
Limmathurotsakul, Direk; Mahidol Oxford Tropical Medicine Research Unit,
Elliott, Elizabeth; University College London, UCL Anthropology; Institut de recherche pour le développement
Ariana, Proochista; University of Oxford Centre for Tropical Medicine, Center for Tropical Medicine and Global Health

**Primary Subject Heading**:	Global health
Secondary Subject Heading:	Health policy, Infectious diseases, Public health, Sociology
Keywords:	Health policy < HEALTH SERVICES ADMINISTRATION & MANAGEMENT, TROPICAL MEDICINE, PUBLIC HEALTH

SCHOLARONE™
Manuscripts

Antibiotic Knowledge, Attitudes, and Practices: New Insights from Cross-Sectional Rural Health Behaviour Surveys in Low- and Middle-Income Southeast Asia

Marco J HAENSSGEN [a, b, c, d], Nutcha CHAROENBOON [d], Giacomo ZANELLO [e, f], Mayfong MAYXAY [a, g, h], Felix REED-TSOCHAS [b, c, i], Yoel LUBELL [a, d], Heiman FL WERTHEIM [j, k], Jeffrey LIENERT [b, c, l], Thipphaphone XAYAVONG [m, n], Yuzana KHINE ZAW [o], Amphayvone THEPKHAMKONG [g], Nicksan SITHONGDENG [g], Nid KHAMSOUKTHAVONG [g], Chanthasone PHANTHAVONG [g], Somsanith BOUALAISENG [g], Souksakhone VONGSAVANG [g], Kanokporn WIBUNJAK [d], Poowadon CHAI-IN [d], Patthanan THAVETHANUTTHANAWIN [d], Thomas ALTHAUS [a, d], Rachel C GREER [a, d], Supalert NEDSUWAN [p], Tri WANGRANGSIMAKUL [a, d], Direk LIMMATHUROTSAKUL [d], Elizabeth ELLIOTT [q, r], Proochista ARIANA [a]

[a.] Centre for Tropical Medicine and Global Health, Nuffield Department of Clinical Medicine, University of Oxford, Oxford, UK
[b.] CABDyN Complexity Centre, Saïd Business School, University of Oxford, Oxford, UK
[c.] Green Templeton College, Oxford, UK
[d.] Mahidol Oxford Tropical Medicine Research Unit, Faculty of Tropical Medicine, Mahidol University, Bangkok, Thailand
[e.] School of Agriculture, Policy and Development, University of Reading, Reading, UK
[f.] Leverhulme Centre for Integrative Research on Agriculture and Health, London, UK
[g.] Lao Oxford Mahosot Wellcome Trust Research Unit (LOMWRU), Mahidol Oxford Tropical Medicine Research Unit (MORU), Faculty of Tropical Medicine, Mahidol University, Bangkok, Thailand
[h.] Institute of Research and Education Development, University of Health Sciences, Vientiane, Lao PDR
[i.] Oxford Martin School, University of Oxford, Oxford, UK
[j.] Oxford University Clinical Research Unit (OUCRU), Ho Chi Minh City, Viet Nam
[k.] Medical Microbiology Department, Radboudumc, Nijmegen, Netherlands
[l.] National Human Genome Research Institute, National Institutes of Health, Bethesda, MD, US
[m.] Department of Peace and Conflict Studies, University for Peace, Costa Rica
[n.] Department of Political Science, School of Social Science, Ateneo de Manila University, Manila, Philippines
[o.] Department of Global Health and Development, London School of Hygiene & Tropical Medicine, London, UK
[p.] Primary Care Department, Chiangrai Prachanukroh Hospital, Chiang Rai, Thailand
[q.] UCL Anthropology, University College London, London, UK
[r.] Institut de recherche pour le développement, Vientiane, Lao PDR

Corresponding author: Marco J HAENSSGEN, Centre for Tropical Medicine and Global Health, Nuffield Department of Medicine, University of Oxford, Old Road Campus, Roosevelt Drive, Oxford OX3 7FZ, UK; phone: +66(0)951906543; email: marco.haenssgen@warwick.ac.uk

WORD COUNT

4,371 (main text, excl. references, figures and tables)

KEYWORDS

Antimicrobial resistance, antibiotics, awareness, treatment-seeking behaviour, survey, social sciences, development studies, interdisciplinary research, Thailand, Lao PDR

For peer review only

ABSTRACT

Introduction: Low- and middle-income countries (LMICs) are crucial in the global response to antimicrobial resistance (AMR), but diverse health systems, healthcare practices, and cultural conceptions of medicine can complicate global education and awareness-raising campaigns. Social research can help understand LMIC contexts but remains underrepresented in AMR research.

Objective: To (1) describe antibiotic-related knowledge, attitudes, and practices of the general population in two LMICs and to (2) assess the role of antibiotic-related knowledge and attitudes on antibiotic access from different types of healthcare providers.

Design: Observational study: cross-sectional rural health behaviour survey, representative on the population level.

Setting: General rural population in Chiang Rai (Thailand) and Salavan (Lao PDR), surveyed between November 2017 and May 2018.

Participants: 2141 adult members (≥18 years) of the general rural population, representing 712,000 villagers.

Outcome measures: Antibiotic-related knowledge, attitudes, and practices across sites and healthcare access channels.

Findings: Villagers were aware of antibiotics (Chiang Rai: 95.7%; Salavan: 86.4%; $p<0.001$) and drug resistance (Chiang Rai: 74.8%; Salavan: 62.5%; $p<0.001$), but the usage of technical concepts for antibiotics was dwarfed by local expressions like “anti-inflammatory medicine” in Chiang Rai (87.6%; 95% confidence interval [CI]: 84.9–90.0) and “*ampi*” in Salavan (75.6%; 95% CI: 71.4–79.4). Multivariate linear regression suggested that attitudes against over-the-counter antibiotics were linked to 0.12 additional antibiotic use episodes from public healthcare providers in Chiang Rai (95% CI: 0.01 – 0.23) and 0.53 in Salavan (95% CI: 0.16 – 0.90).

For peer review only

Conclusions: Locally specific conceptions and counter-intuitive practices around antimicrobials can complicate AMR communication efforts and entail unforeseen consequences. Overcoming "knowledge deficits" alone will therefore be insufficient for global AMR behaviour change. We call for an expansion of behavioural AMR strategies towards "AMR-sensitive interventions" that address context-specific upstream drivers of antimicrobial use (e.g. unemployment insurance) and complement education and awareness campaigns.

Registration: clinicaltrials.gov identifier NCT03241316

STRENGTH AND LIMITATIONS OF THIS STUDY

- Provincial-level representative survey using three-stage stratified cluster random sampling design
- Survey based on preceding qualitative research on antibiotic use in Southeast Asia
- Inclusion of general population enables insights into formal and informal healthcare utilisation
- Cross-sectional analysis of rural health behaviours excludes seasonal change and urban settings
- Two-month recall period enabled greater inclusion but may bias responses towards better educated population groups

MAIN TEXT

Introduction

Antimicrobial resistance (AMR) threatens modern medicine by rendering antimicrobial drugs ineffective. Multi-faceted global strategies target human, animal, and plant health alongside the environment and food production and safety to respond to this "superbug crisis" [1]. In human health, supply-sided responses include incentives to stimulate drug research and development; action on the demand side intends to limit and target antimicrobial use for instance through new diagnostic

For peer review only

technologies, public health intervention to improve vaccine coverage and hygiene, and other antimicrobial stewardship activities like restricted dispensing of antibiotics and prescriber feedback [2-4]. As an interdisciplinary field, the social dimensions of the problem are being recognised in global AMR policy, which are typically addressed via education and awareness-raising activities aimed at governmental staff, healthcare workers, and the general public [2].

Low- and middle-income countries (LMICs) play an important role in the global response to AMR. However, diverse health systems, healthcare practices, and conceptions related to the use of antimicrobials require social research to understand local contexts and the complexity of human behaviour in LMIC settings. For example, with a focus on the health behaviour of the general public, the anthropological literature suggests that social factors like precarity and discrimination can influence medicine use independently of awareness [5]; psychology and behavioural economics indicate that health decision-making processes interact with the social environment and contextual change to create adverse behavioural biases [6 7]; and communication studies research points at interferences between awareness campaigns and local contexts that can entail unforeseen consequences like politicisation, stigmatisation, or accidentally encouraging the behaviours they try to discourage [8 9]. Such examples underline the possible contribution of the social sciences to AMR, but they remain persistently underrepresented with less than 2% of all AMR-related publications (see Figure A1 in supplementary file 1for a time trend) [10]. This is problematic for at least three reasons:

- We currently have an insufficient social science knowledge base for behavioural interventions in AMR—a global health priority that has attracted more than £600 million of AMR expenditure and future commitments [11-13].
- The recent withdrawal of large pharmaceutical companies from antimicrobial research and development [14] threatens the AMR supply-side response, requiring yet more effective action on the demand side.

For peer review only

- More extensive social sciences work can yield novel social innovations as a benefit of disciplinary diversification [15].

Using social research methods, the objectives of this paper were to (1) describe antibiotic-related knowledge, attitudes, and practices of the general population in two LMICs and to (2) assess the role of antibiotic-related knowledge and attitudes on antibiotic access from different types of healthcare providers. We report findings from a provincial-level representative survey of rural health behaviours across 69 villages in northern Thailand and 65 villages in southern Lao PDR as part of the interdisciplinary "Antibiotics and Activity Spaces" project [16].[i] We implemented the study in Southeast Asia, which is characterised as a region "at high risk of the emergence and spread of antibiotic resistance in humans" [17 18]. With more than 9% of global air passengers and more than 110 million international tourist arrivals in 2016 [19], the potential of cross-border spread of drug-resistant microbes also gives AMR research in Southeast Asia a global relevance—as the recent importation of multi-drug-resistant *Neisseria gonorrhoeae* to the UK showed [20]. Within Southeast Asia, Thailand and Lao PDR lent themselves for a comparative analysis because of their physical and cultural proximity, and Chiang Rai (Thailand) and Salavan (Lao PDR) in particular had similarly varied terrain and large and ethnically diverse rural populations. The main field site differences were Thailand's more advanced economic and health system context and more established AMR action plan [21]. For example, Thailand maintains a national strategic plan on antimicrobial resistance (2017-2021) [22]. In addition, according to World Bank data, Thailand's public health expenditure per capita in 2016 were nearly ten times higher than Lao's (USD 496.2 vs. USD 50.1 in purchasing power parity), and Thailand had 2.3 nurses per 1,000 people in 2015, compared to 1.0 per 1,000 people in Lao PDR in 2014 [19].

[i] This paper contributes to the project's research question, "*What are the manifestations and determinants of problematic antibiotic use in patients' healthcare-seeking pathways?*" [16].

For peer review only

Methods

Multistage Survey Design

Our study design was a three-stage stratified cluster random survey (Figure 1): Following the purposive selection of five districts per province, we selected a random sample of 30 primary sampling units (PSUs) per province (six per district), stratified by distance to the nearest district headquarters. The second stage was the selection of an interval sample of 5% but at least 30 of all households in the PSU, which we approximated as residential structures on satellite maps [23]. Participants were sampled in the third and final stage. This process involved the random selection of available household members (one for every five members). At each sampling stage, we substituted unavailable selections (1) with a stratified random replacement for the random PSU sample, (2) with the nearest available neighbour for the interval sample of households, and (3) with a simple random replacement for the random household member sample (replacement numbers indicated in Figure 1). The cross-sectional data collection took place between November 2017 and May 2018.

[Insert Figure 1 about here]

Figure 1. Survey sites and multi-stage sampling process.

Source: Authors, adapted from Wikimedia Commons [24].
Notes: Unavailable selections at each sampling stage were substituted with a random replacement for the random samples of PSUs and household members, and with the nearest available neighbour for the interval sample of households. One PSU could contain more than one administrative village; if the first-chosen village contained less than 600 houses, then adjacent villages would be included. PSU=Primary Sampling Unit.

Study Population

Our study population was the general adult population of rural Chiang Rai and Salavan (522,000 in Chiang Rai and 190,000 in Salavan as per census data), from whom we drew a representative sample of 1158 villagers in Chiang Rai and 983 in Salavan. We did not specifically sample patients, but we

For peer review only

recorded any acute illness episode or accident-related injury if one occurred within the last two months of the interview, both for the respondents and any children under their supervision.

Patient and Public Involvement

This study did not sample patients but only adult members of the general public. The survey instrument was based on preceding qualitative research in Southeast Asia [25 26], in which patients, healthcare providers, and healthy adults participated, but patients or members of the public were not directly involved in the design or conception of the study. This preceding research prompted the research interest in treatment-seeking behaviours and conceptions of medicine and illness among the broader rural population in Southeast Asia. We will disseminate our findings through outreach to policy stakeholders and local development organisations, through public engagement activities like the World Antibiotic Awareness Week, and though our local network of collaborators in the field sites.

Data Collection

Our survey instrument was a 45-minute face-to-face questionnaire (see supplementary file 2). It was administered on tablets running the survey software SurveyCTO (Dobility Inc., Cambridge, MA, USA) by locally recruited survey teams comprising seven enumerators and two survey supervisors per country, who received five days of full-time classroom and field training. The original English questionnaire was co-developed with, and translated into Thai and Lao by, the local research team,[ii] and local translators were recruited for the 228 instances where we encountered language barriers. The

[ii] We refrained from additional back-translation as the local-language versions of the questionnaire were based on qualitative research material that we had previously used in the region, aided further by field pilots and cognitive interviewing.

For peer review only

questionnaires were piloted in rural Chiang Rai and Salavan, with 50 cognitive interviews supporting the questionnaire development and revision as well as the contextualisation of the survey data (not reported here; interview guide in supplementary file 3) [27].

The questionnaire covered basic demographic and socio-economic information, antibiotic-related knowledge and attitudes, and treatment-seeking behaviour during acute illnesses and accident-related injuries. When measuring people's awareness of antibiotics, we could not simply ask villagers whether they knew what "antibiotics" are, considering that (a) a variety of local terms related to antibiotics existed, (b) people may be familiar with specific antibiotic brands but not aware of their antibiotic attributes, and (c) the understanding of technical language was uncommon (see Results section for evidence on this point). We therefore asked respondents first if they recognised images of common antibiotics in the field site.[iii] In the 108/1974 (5.5%) of cases where the respondents did not mention "antibiotics," its colloquial equivalents, or the names of specific antibiotic types, we asked them if they had heard about "anti-inflammatory drugs" ("ยาแก้อักเสบ" or "*yah kae ak seb*") in Thai and "germ resisters" ("ຢາຕ້ານເຊື້ອ" or "*yah dtan suea*") in Lao as common local notions of "antibiotics."

We next asked about the purposes for which the respondent would use these antibiotics, which served as information alongside inputs from local pharmacists to triangulate in later parts of the questionnaire whether the respondent received antibiotics during an illness. However, 752/2986 (25.2%) medicine use episodes could not be confirmed as either antibiotic or non-antibiotic (e.g. "white powder" or "green capsule"). We included these uncertain cases as "potential" antibiotic use episodes to capture behaviour more comprehensively.

[iii] Three images on the survey tablet in Chiang Rai and a bag with seven local antibiotics in Salavan (considering the wider range of terms and medicines in circulation). See questionnaire in supplementary file 2.

For peer review only

Data Analysis

In order to inform the current global health agenda on antibiotic education and awareness raising, we used descriptive statistical analysis and regression analysis to describe the patterns of knowledge and attitudes – and their role in determining antibiotic use – across the two field sites, using the variables described in Table A1 in supplementary file 1. If the common policy narrative holds, then we would expect rural populations in Chiang Rai and Salavan to exhibit:

- low degrees of antibiotic-related knowledge,
- generally high levels of antibiotic consumption especially from informal sources (e.g. unregistered shops selling antibiotics over the counter), and
- lower general antibiotic use and a higher share of supervised antibiotic use from formal healthcare providers among people whose attitudes correspond to awareness-raising messages for AMR (based on FAO/OIE/WHO material, see Table A1 in supplementary file 1) [1].

We stratified and compared the samples by field site (i.e. province) to account for the systemic influence of the health system configuration on people's health knowledge and behaviour in Chiang Rai and Salavan. We estimated provincially representative patterns using post-stratification weights based on census data (considering village size and district-specific age and gender composition) and adjusting the descriptive and regression analysis results for the multi-stage sampling design with the help of the SVY suite of commands in Stata 15 (StataCorp, College Station, TX, USA). We separately analysed the full sample and the subset of respondents who reported a recent illness, whereby we tested differences in knowledge, attitudes, and behaviours across provinces and across antibiotic access channels with X^2 tests for binary and Wilcoxon rank-sum tests (two-sided) for non-normally distributed variables. We further carried out multivariate analysis of the determinants of antibiotic use from public (public hospitals and primary care units), private (private hospitals, clinics, and pharmacies), and informal sources (grocery stores selling medicine, traditional healers). The multivariate analysis used linear regression models adjusted by the complex survey design (sampling clusters and survey

For peer review only

weights), which we compared across the two country samples, using the Chow test to ascertain systematic differences in the determinants of antibiotic use across the two field sites [28].[iv] We indicated significance levels below 0.1, 0.05, and 0.01 with *, **, and ***, respectively.

Results

Representative statistical data of Chiang Rai and Salavan are presented in Appendix Table 1. In terms of socio-demographic characteristics of the rural population in the two provinces, Chiang Rai villagers were older on average ($p<0.001$), tended to have received more formal education ($p<0.001$), and had higher asset wealth ($p<0.001$), while fewer Salavan villagers belonged to the local majority ethnicity ($p=0.030$).

Respondents' recognition of antibiotics in Chiang Rai was significantly higher than in Salavan, but overall high in both sites (Chiang Rai: 95.7%; Salavan: 86.4%; $p<0.001$). Recognition of the phrase "drug resistance" was high as well, whereby 74.8% recognised the term in Chiang Rai and 62.5%

[iv] Although the dependent variables were not normally distributed, the otherwise preferable functional form of Poisson regression did not converge in most cases owing to the relatively small sample sizes. However, where they did converge, the linear regressions yielded more conservative estimates (likewise, the linear regressions adjusted by the complex survey design yielded more conservative results than linear multilevel models that take the hierarchical structure of the data into account). We therefore present the linear regression results in this article. For improved model fitness and to reduce the influence of outliers, we further substituted the duration of the illness with its log. To test for multicollinearity in the cross-sectional survey data, we analysed the pairwise correlations between all independent variables stratified by field site, whereby the largest correlation coefficients in Chiang Rai were +0.59 (ethnicity/religion) and –0.50 (education/age), and in Salavan +0.76 (ethnicity/religion) and +0.62 (religion/wealth) (see Table A3 in supplementary file 1). The largest variance inflation factors (VIFs) were for the dummy variables of religion (VIF = 3.12 in the Salavan sample) and ethnicity (VIF = 2.01 in the Chiang Rai sample), the exclusion of which from the regression models did not produce meaningful differences in parameter estimates or significance levels of the other independent variables. We therefore presented the full regression models to not omit independent variables selectively.

For peer review only

(p<0.001) recognised either of the two common variations in Salavan. Appendix Table 1 further indicates that antibiotic-related knowledge and attitudes aligned more closely with FAO/OIE/WHO messages in Chiang Rai than in Salavan (p<0.001 for all four questions). Across the four questions, respondents in rural Chiang Rai had an average answer score of 1.8 as opposed to rural Salavan with 0.7 (p<0.001).

Figure 2, Panel a, demonstrates the ways in which people related to "antibiotics." In Chiang Rai, respondents commonly referred to antibiotics as "anti-inflammatory drug," representing 87.6% of all responses ("ยาแก้อักเสบ" or "*yah kae ak seb*;" a vernacular notion specific to antibiotics).[v] Only 7.2% used the official term for "antibiotic" ("ยาปฏิชีวนะ" or "*yah pa ti chee wa na*") alongside "*germ killer*" and specific antibiotic types like "*corlam*" (chloramphenicol; 4.6%). In rural Salavan, a larger portion of 38.6% used the official term for antibiotics ("ຢາຕ້ານເຊື້ອ" or "*yah dtan suea*," translated as "germ resister"), but Salavan respondents were also more likely to use various colloquial expressions for specific types of antibiotics, like "*Ampi*" with 75.6% and "*Amok*" with 35.3%.

Reported purposes of antibiotic use were yet more varied and are displayed in Figure 2, Panel b. The overall most common use was the treatment of external wounds (Chiang Rai: 33.7%; Salavan: 44.4%; p<0.001). Other frequently reported uses in Salavan included coughs (30.5%; Chiang Rai: 10.9%; p<0.001) and fevers (30.5%; Chiang Rai: 8.3%; p<0.001). Thai respondents further indicated common use of antibiotics for sore throats (Chiang Rai: 36.3%, where it was the single most common use; Salavan: 28.9%; p=0.016) and for the more general idea of an "inflammation" of the body (Chiang Rai: 23.5%; Salavan: 18.6%; p=0.083). Thai respondents would also more often limit their use to whatever a healthcare worker would recommend (Chiang Rai: 9.5%; Salavan: 5.4%; p=0.037), while 2.3% indicated that they would treat their plants or animals (dogs and chickens) with antibiotics

[v] Actual anti-inflammatory medicine like ibuprofen would usually be referred to by its brand names.

For peer review only

(Salavan: 0.1%; p<0.001). Using antibiotics to treat infections or to fight bacteria and germs was only mentioned by a small minority of the rural populations (Chiang Rai: 2.4%; Salavan: 2.8%; p=0.243).

[Insert Figure 2 about here]

Figure 2. Common names and purposes for antibiotics.

Source: Authors' analysis of survey data.
Notes: Only including respondents who indicated that they had seen the presented medicine (i.e. common antibiotics) before. Chiang Rai: *n* = 1076; Salavan: *n* = 775. Population-weighted statistics, accounting for complex survey design. Multiple response permitted. Error bars indicate 95% confidence interval.

Table 1 indicates that—though people typically recognised the term "drug resistance"—the responses to the question "*What do you think is drug resistance?*" only rarely corresponded to clinical definitions, and the coexistence of two common translations of the term in Lao PDR complicated the picture further. In Chiang Rai, 10.6% of the interpretations related to antibiotics and/or drug-resistant germs. Lao respondents linked the official term "*due yah*" to clinical definitions in 7.7% of all interpretations, and the colloquial term "*lueng yah*" in 9.6% of all interpretations. Not unlike other high- and low-income countries [29], drug resistance was typically interpreted as a growing tolerance of the body towards medicine as a result of repeated use (not limited to antibiotics). Other common interpretations in Chiang Rai were the incorrect or erratic use of medicine (12.5%), and an understanding of drug resistance as side-effects of or allergic reactions to medicine in general (4.2%). In Salavan, "*due yah*" was often interpreted as a refusal or "stubbornness" to take medicine (21.8%; possibly due to its literal translation into "*stubborn* [to the effect of] *medicine*"), while its vernacular equivalent "*lueng yah*" was often interpreted in the opposite way as a psychological dependence or addiction to medicine (24.9%).

Table 1. Awareness and interpretations of "drug resistance."

For peer review only

	Chiang Rai		Salavan			
	"due yah"		***"due yah"***		***"lueng yah"***	
Awareness in rural population	72.9% (67.4–77.8)		27.1% (22.2–32.6)		58.8% (54.6–63.0)	
**Top 5 interpretations**						
Rank 1	Body becomes tolerant to medicine	54.1% (49.3–58.9)	Body becomes tolerant to medicine	38.1% (30.4–46.4)	Body becomes tolerant to medicine	50.9% (44.7–57.1)
Rank 2	Taking medicine incorrectly	12.5% (10.2–15.3)	Patient is "stubborn," refuses medicine	21.8% (14.8–31.0)	Addicted to / preference for medicine	24.9% (20.2–30.2)
Rank 3	**Reference to antibiotics, drug-resistant germs**	**10.6% (8.1–13.8)**	Side-effects, drug allergy	9.2% (5.2–15.8)	**Reference to antibiotics, drug-resistant germs**	**9.6% (6.9–13.1)**
Rank 4	Don't know	6.3% (4.6–8.7)	**Reference to antibiotics, drug-resistant germs**	**7.7% (4.7–12.5)**	Don't know	4.0% (2.3–6.8)
Rank 5	Side-effects, drug allergy	4.2% (2.6–6.7)	Addicted to / preference for medicine	7.1% (3.5–13.8)	Sickness is "stubborn" / unresponsive	2.9% (1.3–6.2)

Source: Authors' analysis of survey data.
Notes: Ranking percentages only include respondents who indicated that they had heard the respective term "drug resistance" before. 95% confidence intervals in parentheses. Chiang Rai: *n* = 871; Salavan (*due yah*): *n* = 206; Salavan (*lueng yah*): *n* = 470. Population-weighted statistics, accounting for complex survey design. Only single response permitted. In Salavan, the common response "*due yah* means *lueng yah*" (24.8% [18.4–32.6]) was recoded to incorporate respondent's definition of *lueng yah*.

According to our survey data, 99.9% of the rural population in Chiang Rai and 91.6% of the rural Salavan population had a public primary healthcare centre within a 10km radius. Private sources were more varied, as 93.0% and 34% of the rural Chiang Rai population had a private clinic and a pharmacy within a 10km radius, respectively (37.8% and 47.4% in Salavan, respectively), whereas informal healthcare through shops and informal healers was nearly universally available within the survey villages (>97.8% in all cases).

Among our 2141 respondents, we captured 608 illness episodes in Chiang Rai and 356 in Salavan (Appendix Table 1). Healthcare utilisation during these episodes varied slightly across the two field sites. Chiang Rai respondents accessed a narrower spectrum of healthcare providers and were significantly less likely to access public and "other" healthcare providers ($p<0.001$ in both cases). Both

For peer review only

sites also exhibited a high level of medicine access, with 2.2 and 2.5 medicine use episodes during an illness Chiang Rai and Salavan, respectively (p=0.050). Respondents in Chiang Rai thereby indicated higher use of non-antibiotic medicine (Chiang Rai: 1.6; Salavan: 1.3; p=0.048). In contrast, respondents in Salavan had more episodes of antibiotic use per illness (Chiang Rai: 0.2; Salavan: 0.4; p<0.001), and more usage of medicines that could potentially include antibiotics (Chiang Rai: 0.4; Salavan: 0.9; p<0.001). The pattern of antibiotic access was similar for informal sources, but generally lower in Chiang Rai: confirmed antibiotic use from informal channels represented 1.6% of all medicine use episodes in Chiang Rai and 3.3% in Salavan; and 3.6% in Chiang Rai and 7.9% in Salavan if unconfirmed but potential antibiotic use episodes are included.

Comparing the bivariate differences between individuals who accessed antibiotics from public, private, and informal sources (see Table A2 in supplementary file 1) indicated that, contrary to intuition, patients receiving antibiotics from informal sources had no less wealth or formal education than users of public healthcare. Indeed, wealthier and more educated individuals in Chiang Rai were significantly associated with receiving antibiotics from informal sources (wealth: p=0.012; education: p=0.032). Similarly, awareness of drug resistance was not significantly lower among patients who received antibiotics from informal sources, while the share of respondents who linked drug resistance to biomedical notions of AMR in Salavan was significantly higher among individuals accessing antibiotics through informal channels compared to public channels (13.4% vs. 4.4%, p=0.030). Patients who accessed antibiotics through informal channels were nevertheless significantly more inclined towards buying over-the-counter antibiotics than public antibiotic users (Chiang Rai: p=0.040; Salavan: p<0.001).

The results of the multivariate analysis are presented in Table 2. The dependent variables were the number of confirmed antibiotic use episodes from public, private, and informal sources; as a sensitivity check, we also included the more inclusive definition of “confirmed and potential” antibiotic use episodes. The relationship between antibiotic-related knowledge and attitudes and antibiotic use was

For peer review only

mixed. Among the most common knowledge- and attitude-related predictors of antibiotic use was the respondents' inclination to buy antibiotics. Again contrary to expectations, the attitude to not buy antibiotics over the counter was linked to disproportionate consumption of antibiotics from public sources. For example, patients who would not buy antibiotics over the counter would have 0.12 additional potential antibiotic use episodes in Chiang Rai (Model 7; 95% CI: 0.01 – 0.23) and up to 0.53 in Salavan (Model 8; 95% CI: 0.16 – 0.90), *ceteris paribus*. Also knowledge of antibiotics was positively associated with antibiotic consumption from private sources in Chiang Rai (Model 3) and from public and informal sources in Salavan (Models 2 and 6). In contrast, other antibiotic-related attitudes and knowledge linked negatively to antibiotic consumption. For example, the knowledge that antibiotic resistance can spread was linked to 0.25 fewer potential antibiotic use episodes from informal sources (Model 12, 95% CI: -0.39 – -0.10) and Chiang Rai patients who preferred alternatives over antibiotics had 0.12 fewer confirmed antibiotic use episodes from private sources (Model 3; 95% CI: -0.22 – -0.03).

Among other covariates, wealthier patients had lower consumption of antibiotics from public (Chiang Rai and Salavan, Models 7 and 8) but also higher consumption from private (Salavan, Model 4) and informal healthcare providers (Chiang Rai, Models 5 and 11) – presumably enabled by their higher purchasing power. Speakers of the majority language were also more likely to consume more antibiotics from public sources, which resonates with anecdotes encountered during the field research according to which speakers of minority languages tended to be less assertive in the patient-doctor encounter (the link to education was less clear). Not surprisingly, longer illness episodes were also associated with more antibiotic use episodes from public and private sources.

The Chow test indicated that the determinants of confirmed antibiotic use from private sources were significantly different in Chiang Rai and Salavan (Models 3 and 4; p=0.011), as were all sources of potential and confirmed antibiotic use (Models 7 to 12; p=0.056, p=0.015, and p=0.083 for public, private, and informal antibiotic use, respectively).

For peer review only

Table 2. Regression results of determinants of antibiotic use from public, private, and informal sources.

	Confirmed antibiotic use episodes						Confirmed and potential antibiotic use episodes					
	Public providers		Private providers		Informal providers		Public providers		Private providers		Informal providers	
Site	CR	SAL	CR	SAL	CR	SAL	CR	SAL	CR	SAL	CR	SAL
Model number	(1)	(2)	(3)	(4)	(5)	(6)	(7)	(8)	(9)	(10)	(11)	(12)
**Independent variables**												
Would not buy antibiotics over the counter	0.045* (-0.01 - 0.10)	0.168** (0.02 - 0.32)	0.074 (-0.02 - 0.16)	-0.040 (-0.13 - 0.05)	-0.050 (-0.11 - 0.01)	-0.008 (-0.09 - 0.08)	0.117** (0.01 - 0.23)	0.530*** (0.16 - 0.90)	0.151* (-0.03 - 0.33)	-0.095 (-0.33 - 0.14)	-0.031 (-0.10 - 0.04)	0.021 (-0.14 - 0.18)
Prefers antibiotics over alternatives	-0.023 (-0.08 - 0.03)	-0.017 (-0.12 - 0.08)	-0.124** (-0.22 - - 0.03)	-0.009 (-0.10 - 0.08)	0.033 (-0.02 - 0.08)	-0.014 (-0.09 - 0.07)	-0.026 (-0.17 - 0.12)	-0.222* (-0.44 - 0.00)	-0.161* (-0.35 - 0.03)	0.054 (-0.18 - 0.28)	0.005 (-0.07 - 0.08)	0.044 (-0.10 - 0.19)
Does not keep antibiotics for future use	-0.049* (-0.10 - 0.00)	-0.008 (-0.11 - 0.09)	-0.060 (-0.14 - 0.02)	-0.013 (-0.12 - 0.09)	-0.024 (-0.08 - 0.03)	0.037 (-0.07 - 0.15)	-0.073 (-0.20 - 0.05)	0.185 (-0.18 - 0.55)	-0.056 (-0.25 - 0.14)	0.056 (-0.26 - 0.37)	-0.049 (-0.12 - 0.02)	-0.050 (-0.21 - 0.11)
Knows that antibiotic resistance can spread	0.041 (-0.05 - 0.13)	-0.088 (-0.35 - 0.17)	0.030 (-0.11 - 0.17)	-0.051 (-0.34 - 0.24)	0.035 (-0.04 - 0.11)	-0.155*** (-0.25 - - 0.06)	0.068 (-0.15 - 0.29)	-0.355 (-1.53 - 0.82)	-0.060 (-0.25 - 0.13)	-0.138 (-0.73 - 0.46)	0.013 (-0.09 - 0.11)	-0.247*** (-0.39 - -0.10)
Aware of antibiotics	-0.028 (-0.12 - 0.07)	0.101* (-0.00 - 0.20)	0.109** (0.01 - 0.21)	0.017 (-0.08 - 0.11)	-0.135 (-0.38 - 0.10)	0.064** (0.01 - 0.12)	0.093 (-0.20 - 0.39)	0.185 (-0.28 - 0.65)	-0.197 (-0.78 - 0.39)	-0.346 (-0.94 - 0.25)	-0.073 (-0.33 - 0.18)	0.130* (-0.00 - 0.26)
Aware of drug resistance[a]	-0.041 (-0.11 - 0.03)	0.056 (-0.08 - 0.20)	0.056 (-0.03 - 0.14)	0.086 (-0.04 - 0.21)	0.016 (-0.01 - 0.05)	0.014 (-0.09 - 0.12)	-0.143* (-0.29 - 0.01)	-0.041 (-0.39 - 0.31)	-0.063 (-0.33 - 0.20)	0.113 (-0.16 - 0.38)	-0.024 (-0.09 - 0.04)	-0.027 (-0.20 - 0.14)
Female	-0.005 (-0.06 - 0.05)	0.086* (-0.01 - 0.18)	-0.005 (-0.09 - 0.08)	-0.064 (-0.17 - 0.04)	0.035 (-0.02 - 0.09)	0.049 (-0.03 - 0.13)	-0.122 (-0.27 - 0.03)	0.168 (-0.09 - 0.42)	0.001 (-0.19 - 0.20)	-0.296* (-0.59 - 0.00)	0.028 (-0.04 - 0.09)	0.181** (0.03 - 0.33)
Age	-0.001 (-0.00 - 0.00)	-0.001 (-0.00 - 0.00)	0.003* (-0.00 - 0.01)	-0.001 (-0.00 - 0.00)	-0.002* (-0.00 - 0.00)	0.002 (-0.00 - 0.00)	0.001 (-0.00 - 0.01)	-0.001 (-0.01 - 0.01)	0.009** (0.00 - 0.02)	-0.004 (-0.01 - 0.00)	-0.001 (-0.00 - 0.00)	0.007** (0.00 - 0.01)
Education (years)	-0.002 (-0.01 - 0.01)	-0.012* (-0.02 - 0.00)	0.004 (-0.01 - 0.02)	0.012 (-0.00 - 0.03)	-0.006** (-0.01 - - 0.00)	0.010 (-0.00 - 0.02)	0.001 (-0.01 - 0.02)	-0.001 (-0.05 - 0.05)	0.015 (-0.01 - 0.04)	0.018 (-0.01 - 0.05)	-0.001 (-0.01 - 0.01)	0.010 (-0.01 - 0.03)
Speaking Thai / Lao	0.161*** (0.07 - 0.25)	0.251*** (0.10 - 0.40)	0.005 (-0.12 - 0.13)	-0.010 (-0.12 - 0.10)	0.019 (-0.02 - 0.06)	0.058 (-0.01 - 0.13)	-0.016 (-0.46 - 0.43)	0.161 (-0.27 - 0.59)	-0.336 (-0.79 - 0.12)	-0.005 (-0.30 - 0.29)	0.033 (-0.05 - 0.12)	0.152** (0.01 - 0.30)
Wealth index	-0.179 (-0.42 - 0.06)	-0.105 (-0.47 - 0.26)	-0.001 (-0.28 - 0.28)	0.340* (-0.01 - 0.69)	0.158** (0.01 - 0.31)	-0.121 (-0.35 - 0.10)	-0.417* (-0.90 - 0.06)	-1.073* (-2.26 - 0.12)	0.323 (-0.30 - 0.95)	0.505 (-0.30 - 1.31)	0.241** (0.02 - 0.46)	0.065 (-0.73 - 0.86)
Buddhist religion	-0.016 (-0.11 - 0.08)	-0.065 (-0.29 - 0.16)	-0.074 (-0.21 - 0.06)	-0.031 (-0.18 - 0.12)	0.010 (-0.02 - 0.04)	0.028 (-0.06 - 0.12)	-0.033 (-0.22 - 0.16)	0.037 (-0.40 - 0.47)	-0.122 (-0.44 - 0.19)	-0.031 (-0.35 - 0.29)	0.017 (-0.05 - 0.08)	-0.021 (-0.23 - 0.19)
Thai/Lao nationality	-0.030 (-0.13 - 0.07)	-0.140 (-0.53 - 0.25)	-0.069 (-0.24 - 0.10)	0.013 (-0.11 - 0.13)	-0.003 (-0.06 - 0.06)	0.088 (-0.03 - 0.21)	0.009 (-0.37 - 0.39)	0.003 (-0.43 - 0.44)	0.005 (-0.52 - 0.53)	-0.118 (-0.46 - 0.22)	-0.078 (-0.24 - 0.09)	-0.603 (-1.95 - 0.74)
Majority ethnic group (Thai/Lao Loum)	0.033 (-0.04 - 0.11)	0.169* (-0.02 - 0.36)	0.065 (-0.04 - 0.17)	-0.059 (-0.19 - 0.07)	0.050** (0.01 - 0.09)	-0.039 (-0.14 - 0.06)	0.049 (-0.11 - 0.21)	0.360* (-0.04 - 0.76)	-0.044 (-0.27 - 0.18)	0.118 (-0.20 - 0.44)	0.035 (-0.03 - 0.10)	0.030 (-0.17 - 0.23)
Self-rated severity (1=mild, 2=medium, 3=severe)	0.063*** (0.02 - 0.10)	0.009 (-0.08 - 0.09)	0.002 (-0.05 - 0.05)	0.062 (-0.02 - 0.14)	0.009 (-0.02 - 0.04)	0.023 (-0.03 - 0.08)	0.077 (-0.02 - 0.17)	0.254** (0.01 - 0.50)	0.068 (-0.06 - 0.20)	0.107 (-0.17 - 0.39)	0.065** (0.01 - 0.12)	-0.038 (-0.20 - 0.12)
Log of duration of illness episode (days)	0.060*** (0.02 - 0.10)	0.036 (-0.01 - 0.08)	0.076*** (0.03 - 0.12)	0.046 (-0.01 - 0.10)	0.003 (-0.02 - 0.02)	-0.025 (-0.07 - 0.02)	0.275*** (0.16 - 0.39)	0.507*** (0.27 - 0.74)	0.257*** (0.10 - 0.41)	0.234** (0.05 - 0.42)	0.005 (-0.03 - 0.04)	-0.012 (-0.13 - 0.10)
Constant	-0.007 (-0.23 - 0.21)	-0.123 (-0.55 - 0.30)	-0.152 (-0.38 - 0.08)	-0.198 (-0.46 - 0.07)	0.117 (-0.12 - 0.35)	-0.207* (-0.43 - 0.02)	-0.022 (-0.61 - 0.56)	-0.767 (-1.82 - 0.29)	-0.181 (-1.08 - 0.72)	0.029 (-1.17 - 1.23)	-0.006 (-0.37 - 0.35)	0.199 (-1.18 - 1.58)
**Model Statistics**												
Number	604	356	604	356	604	356	604	356	604	356	604	356
R^2	0.102	0.106	0.080	0.133	0.104	0.046	0.143	0.218	0.126	0.136	0.052	0.095
F statistic	2.29***	3.34***	1.89**	1.79**	0.89	1.48	2.82***	4.57***	2.41***	1.30	1.18	2.27***
**Chow Test**	**(2) – (1) = 0**		**(4) – (3) = 0**		**(6) – (5) = 0**		**(8) – (7) = 0**		**(10) – (9) = 0**		**(12) – (11) = 0**	
F statistic	1.33		2.05**		1.08		1.65*		1.97**		1.55*	

Source: Authors' analysis of survey data.

Notes: Illness episode level, including completed illnesses experienced by respondent or child under their supervision, excluding incomplete episodes. Population-weighted statistics, accounting for complex survey design. 95% confidence intervals in parentheses. CR = Chiang Rai. SAL = Salavan.

For peer review only

*p < 0.1, **p < 0.05, ***p < 0.01.
a. Comparing Thai "due yah" with the combined Lao "due yah" and "lueng yah."

Discussion and Conclusion

Our paper aimed at understanding AMR-related general population behaviour in LMICs through a description of antibiotic-related knowledge, attitudes, and practices in rural Thailand and Lao PDR, and an assessment of the role of antibiotic-related knowledge and attitudes on antibiotic access from different types of healthcare providers. We demonstrated that rural populations exhibited:

- mixed but surprisingly high levels of awareness and attitudes corresponding to AMR awareness-raising material, although only a minority of villagers were familiar with technical notions of antibiotics and drug resistance;
- relatively low levels of antibiotic access from informal sources; and
- surprisingly counter-intuitive links between informal antibiotic use, socio-economic status, and their attitudes—especially among villagers in Salavan, who had disproportionately high antibiotic use if their attitude showed a disinclination against over-the-counter antibiotics.

Our survey data also revealed profound differences between the two field sites despite their cultural and geographical proximity. For example, villagers referred to antibiotics with wide-ranging and locally specific vernacular expressions (only a minority adopted technical language in either site), and "drug resistance" was typically understood as a general tolerance of the body to medicine but local interpretations ranged from patients refusing medicine to patients being addicted to medicine. Some of these differences could be explained by the local health system configuration. The better endowed and more regulated health system as well as the more extensive public health campaigns in Chiang Rai arguably contributed to the higher rates of public awareness and the comparatively lower rates of antibiotic use, whereas the Salavan health system faced more pressing trade-offs between ensuring access to and preventing the overuse of antibiotics. Alas, as the analysis has shown, antibiotic-related

For peer review only

awareness and attitudes appeared to have little bearing on people's antibiotic consumption when controlling for other determinants of medicine use.

The surveys were implemented after the Monsoon season to reduce accessibility barriers like landslides, floods, and farm work. This temporal focus meant that our survey was not able to capture internal migration or seasonal change affecting the epidemiological environment. The rural survey is also unable to speak for urban health behaviour or behavioural patterns outside rural Thailand and Lao PDR, or for awareness and behaviour among healthcare staff and policy makers (with which awareness-raising activities for the general public may interact). Lastly, our focus on health behaviour and our 60-day recall period could introduce recall and social desirability biases. Most LMIC health behaviour research uses 14-30-day recall periods; longer recall periods can lead to underrepresentation of lower educated groups [30]. However, for a survey of behaviour rather than of epidemiological patterns, 14-day recall would have truncated the sample to an impractical size (omitting 540/964 [56.0%] of all responses) and neglected that illness episodes often extended beyond a fortnight (as was the case for 91/964 [8.7%] of the recorded illnesses). In response, we conducted regular review sessions with our survey team to identify and alleviate social desirability; we excluded chronic illnesses; and our questionnaire asked our respondents to walk through the sequence of events, which improves recall [31]. While we cannot rule out a residual risk of social desirability and recall bias, it is not clear *a priori* whether and how any remaining bias would affect our comparison of antibiotic uses across different healthcare providers.

By virtue of being a representative rural survey in northern Thailand and southern Lao PDR, the specific notions and behavioural patterns around antibiotic use are not generalisable beyond the study context, although similar interpretations of antibiotics as "anti-inflammatory medicine" exist elsewhere (e.g. in China) [32], and even the documented practice of using antibiotics in plant cultivation has historical antecedents [33]. Other study findings like the widespread use of antibiotics for external (and often allegedly "internal") wounds have few documented equivalents in other settings

For peer review only

and deserve further research. However, the findings of our study have a broader relevance insofar as they expose the complexity of local knowledge and its relationship to AMR-related behaviour. On the one hand, our work underlines the challenges facing public awareness campaigns as the current principal strategy to change AMR-related population behaviour. For example, if not mindful of the local context, the slogan of the 2017 World Antibiotic Awareness Week to "use antibiotics wisely to combat rising drug resistance" could plausibly entail *increased* antibiotic use or the use of stronger medicine if people understand drug resistance as stubbornness of patients or as a problem applying to all types of medicines.

On the other hand, our study also demonstrated that the link between knowledge, attitudes, and antibiotic-related behaviour may be weak in LMIC contexts. This disjunction is not new [5], but the counter-intuitive link between awareness, antibiotic-related attitudes, and antibiotic use from informal sources suggests that AMR-related information can easily entail unintended consequences—knowledge and awareness empower, but people themselves decide how they will use this new "power" in their daily lives [34]. For instance, villagers may not necessarily buy antibiotics from private clinics and unregulated corner shops because of ignorance, but because they become more assertive about their health (and increased wealth may enable patients to exercise this assertiveness).

Considering potential misunderstandings in AMR communication on the one hand and contextual determinants of behaviour beyond knowledge deficits on the other, we call for an expansion of behavioural AMR strategies to address structural factors of behavioural change. For example, vulnerability and adversity may drive people into seemingly irrational antimicrobial use [35]. A sick labourer or factory worker may take antimicrobials desperately to maintain their job and to sustain their families, in which case it would be futile trying to convince them that their hardship is secondary to the global health goal of tackling AMR. Yet, it may be possible to alleviate their pressure to consume antimicrobials through paid sick leave and unemployment insurance. We propose the exploration of such "AMR-sensitive interventions" to address upstream drivers of antimicrobial use and to

For peer review only - http://bmjopen.bmj.com/site/about/guidelines.xhtml

complement education and awareness campaigns—similar to nutrition-sensitive interventions that target the determinants of mal- and undernutrition through upstream interventions like social safety nets (rather than e.g. providing supplements directly to people) [36]. AMR-sensitive interventions require us to venture out of health policy terrain into broader development policy. There is yet little evidence whether and how such context-oriented approaches bear fruit. Greater involvement of the social sciences is necessary to uncover this gap and to find constructive solutions that address the social factors of which AMR is a symptom.

ACKNOWLEDGEMENTS

We thank our collaborators Caroline OH Jones, Romyen Kosaikanont, Pollavat Praphattong, Pathompong Manohan, Paul N Newton, Sommay Keomany, Penporn Warapikuptanun, Daranee Intralawan, Patchapoom U-Thong, Patipat Benjaroon, Narinnira Sangkham, Sirirat Chailert, Krittanon Promsutt, Maipheth Keovilayvanh, and Phaengnitta Phanthasomchit for guidance during the study design and for field survey implementation assistance. We further thank Ernest R Guevarra and Eric Ohuma for helpful discussions, feedback, and support during the survey design phase; Edward Gibbs, Lisa White, the Centre for Tropical Medicine and Global Health, the CABDyN Complexity Centre, and the Mahidol-Oxford Tropical Medicine Research Unit / Wellcome Trust Strategic Award for institutional support; Zoë Doran and the Clinical Trials Support Group at the Mahidol Oxford Tropical Medicine Research Unit for quality assurance during the protocol development process; Alison Brindle, Claire-Lise Kessler, and John Bleho for engaging communication work; and Karen Valentine, Holly Blades, and Kanchana Pongsaswat for indispensable operational and logistical assistance.

AUTHOR CONTRIBUTIONS

Study conceptualisation, design, and theoretical framing: MJH

For peer review only

Study design: MJH, NC, GZ, MM, FR-T, YL, HFLW, JL, TX, YKZ, AT, NS, NK, CP, SB, SV, KW, PC-I, PT, TA, RCG, SN, TW, DL, EE, PA

Survey instrument development: MJH, GZ, NC

Study protocol development: MJH, NC

Data cleaning and coding: MJH, NC

Data analysis and manuscript draft: MJH

Manuscript review and approval: MJH, NC, GZ, MM, FR-T, YL, HFLW, JL, TX, YKZ, AT, NS, NK, CP, SB, SV, KW, PC-I, PT, TA, RCG, SN, TW, DL, EE, PA

COMPETING INTERESTS

We declare that no conflict of interest – financial or otherwise – exists.

DATA SHARING

The data set will be made publicly available in June 2019 on the UK Data Service and equivalent repositories in Thailand and Lao PDR.

ETHICS APPROVAL

The research was reviewed and approved by the University of Oxford Tropical Research Ethics Committee (Ref. OxTREC 528-17), and it received local ethical approval in Thailand from the Mae Fah Luang University Research Ethics Committee on Human Research (Ref. REH 60099), and in Lao PDR from the National Ethics Committee for Health Research (Ref. NEHCR 074). Participation in the survey was voluntary and we obtained informed verbal consent from all participants, which was audio recorded and documented by the survey field investigators with a written record of oral consent for each participant.

For peer review only

FUNDING SOURCES

This project is funded by the Antimicrobial Resistance Cross Council Initiative supported by the seven research councils in partnership with the Department of Health and Department for Environment Food & Rural Affairs (grant ref. ES/P00511X/1, administered by the UK Economic and Social Research Council). YKZ was further supported by internal research placement funding by the MSc International Health and Tropical Medicine (University of Oxford). The funders had no involvement in the design and implementation of the project.

For peer review only

REFERENCES

1. FAO, OIE, WHO. Handle antimicrobials with care: Tripartite brochure [infographic]. Geneva: World Health Organization, 2017.

2. The Review on Antimicrobial Resistance. Tackling drug-resistant infections globally: final report and recommendations. London: The UK Prime Minister, 2016.

3. WHO. Global action plan on antimicrobial resistance. Geneva: World Health Organization, 2015.

4. MacDougall C, Polk RE. Antimicrobial stewardship programs in health care systems. Clinical Microbiology Reviews 2005;**18**(4):638-56 doi: 10.1128/CMR.18.4.638-656.2005.

5. Chandler CIR, Hutchinson E, Hutchison C. Addressing antimicrobial resistance through social theory: an anthropologically oriented report. London: London School of Hygiene & Tropical Medicine, 2016.

6. Cawley J, Ruhm CJ. The economics of risky health behaviors. In: Pauly MV, Mcguire TG, Barros PP, eds. Handbook of health economics. Amsterdam: Elsevier, 2011:95-199.

7. Sheehy-Skeffington J, Rea J. How poverty affects people's decision-making processes. York: Joseph Rowntree Foundation, 2017.

8. Davis M, Whittaker A, Lindgren M, et al. Understanding media publics and the antimicrobial resistance crisis. Global Public Health 2017;**13**(9):1158-68 doi: 10.1080/17441692.2017.1336248.

9. Fynbo L, Jensen CS. Antimicrobial stigmatization: public health concerns about conventional pig farming and pig farmers' experiences with stigmatization. Social science & medicine 2018;**201**:1-8 doi: 10.1016/j.socscimed.2018.01.036.

10. Elsevier B.V. Scopus. Secondary Scopus 2018. http://www.scopus.com/.

11. Research Councils UK. Gateway to research. Secondary Gateway to research 2018. http://gtr.rcuk.ac.uk/.

12. Kinh NV, Wertheim HFL, Thwaites GE, et al. Developing an antimicrobial resistance reference laboratory and surveillance programme in Vietnam. The Lancet Global Health 2017;**5**(12):e1186-e87 doi: 10.1016/S2214-109X(17)30370-4.

13. GARDP. Business plan 2017-2023: developing new antibiotic treatments and ensuring sustainable access for all. Geneva: Global Antibiotic Research and Development Partnership, 2017.

14. Paton J, Kresge N. Superbugs win another round as Big Pharma leaves antibiotics. Secondary Superbugs win another round as Big Pharma leaves antibiotics 2018. https://www.bloomberg.com/news/articles/2018-07-13/superbugs-win-another-round-as-big-pharma-leaves-antibiotics.

15. The Lancet. UK life science research: time to burst the biomedical bubble. The Lancet 2018;**392**(10143):187 doi: 10.1016/S0140-6736(18)31609-X.

16. Haenssgen MJ, Charoenboon N, Zanello G, et al. Antibiotics and activity spaces: protocol of an exploratory study of behaviour, marginalisation, and knowledge diffusion. BMJ Global Health 2018;**3**(e000621) doi: 10.1136/bmjgh-2017-000621.

For peer review only

17. Holloway KA, Batmanabane G, Puri M, et al. Antibiotic use in South East Asia and policies to promote appropriate use: reports from country situational analyses. Bmj 2017;**358**(Suppl1) doi: 10.1136/bmj.j2291.

18. Chereau F, Opatowski L, Tourdjman M, et al. Risk assessment for antibiotic resistance in South East Asia. Bmj 2017;**358**(Suppl1) doi: 10.1136/bmj.j3393.

19. World Bank. World databank. Secondary World databank 2018. http://databank.worldbank.org/data/home.aspx.

20. Public Health England. UK case of Neisseria gonorrhoeae with high-level resistance to azithromycin and resistance to ceftriaxone acquired abroad. Health Protection Report Advanced Access Report 2018;**12**(11).

21. Klein EY, Van Boeckel TP, Martinez EM, et al. Global increase and geographic convergence in antibiotic consumption between 2000 and 2015. Proceedings of the National Academy of Sciences 2018;**115**(15):E3463-E70 doi: 10.1073/pnas.1717295115.

22. Sumpradit N, Wongkongkathep S, Poonpolsup S, et al. New chapter in tackling antimicrobial resistance in Thailand. Bmj 2017;**358**(Suppl1) doi: 10.1136/bmj.j2423.

23. Haenssgen MJ. Satellite-aided survey sampling and implementation in low- and middle-income contexts: a low-cost/low-tech alternative. Emerging Themes in Epidemiology 2015;**12**(20) doi: 10.1186/s12982-015-0041-8.

24. Wikimedia Commons. Locator map of Chiang Rai Province, Thailand, by NordNordWest; a map of Laos with Salavan province highlighted, by Infernoapple. Secondary Locator map of Chiang Rai Province, Thailand, by NordNordWest; a map of Laos with Salavan province highlighted, by Infernoapple 2018. Wikimedia Commons Web pages: http://en.wikipedia.org/wiki/File:Gansu_in_China_(%2Ball_claims_hatched).svg and https://commons.wikimedia.org/wiki/File:Rajasthan_in_India_(claimed_and_disputed_hatched).svg?uselang=en.

For peer review only

25. Haenssgen MJ, Charoenboon N, Althaus T, et al. The social role of C-reactive protein point-of-care testing to guide antibiotic prescription in Northern Thailand. Social science & medicine 2018;**202**:1-12 doi: 10.1016/j.socscimed.2018.02.018.

26. Khine Zaw Y, Charoenboon N, Haenssgen MJ, et al. A comparison of patients' local conceptions of illness and medicines in the context of C-reactive protein biomarker testing in Chiang Rai and Yangon. American Journal of Tropical Medicine and Hygiene 2018;**98**(6):1661-760 doi: 10.4269/ajtmh.17-0906.

27. Willis GB. The practice of cross-cultural cognitive interviewing. Public Opinion Quarterly 2015;**79**(S1):359-95 doi: 10.1093/poq/nfu092.

28. Chow GC. Tests of equality between sets of coefficients in two linear regressions. Econometrica 1960;**28**(3):591-605 doi: 10.2307/1910133.

29. McCullough AR, Parekh S, Rathbone J, et al. A systematic review of the public's knowledge and beliefs about antibiotic resistance. Journal of Antimicrobial Chemotherapy 2016;**71**(1):27-33 doi: 10.1093/jac/dkv310.

30. Das J, Hammer J, Sánchez-Paramo C. The impact of recall periods on reported morbidity and health seeking behavior. Journal of Development Economics 2012;**98**(1):76-88 doi: 10.1016/j.jdeveco.2011.07.001.

31. Fowler FJ. *Improving survey questions: design and evaluation*. Thousand Oaks, CA: Sage, 1995.

32. Yu M, Zhao G, Stålsby Lundborg C, et al. Knowledge, attitudes, and practices of parents in rural China on the use of antibiotics in children: a cross-sectional study. BMC Infectious Diseases 2014;**14**(1):112 doi: 10.1186/1471-2334-14-112.

33. Kirchhelle C. Pharming animals: a global history of antibiotics in food production (1935–2017). Palgrave Communications 2018;**4**(96) doi: 10.1057/s41599-018-0152-2.

For peer review only

34. Carrion ML. "You need to do your research": vaccines, contestable science, and maternal epistemology. Public Understanding of Science 2018;**27**(3):310-24 doi: 10.1177/0963662517728024.

35. Ribera JM, Hausmann-Muela S. The straw that breaks the camel's back: redirecting health-seeking behavior studies on malaria and vulnerability. Medical Anthropology Quarterly 2011;**25**(1):103-21 doi: 10.1111/j.1548-1387.2010.01139.x.

36. Ruel MT, Alderman H, The Maternal and Child Nutrition Study Group. Nutrition-sensitive interventions and programmes: How can they help to accelerate progress in improving maternal and child nutrition? The Lancet 2013;**382**(9891):536-51 doi: 10.1016/S0140-6736(13)60843-0.

For peer review only

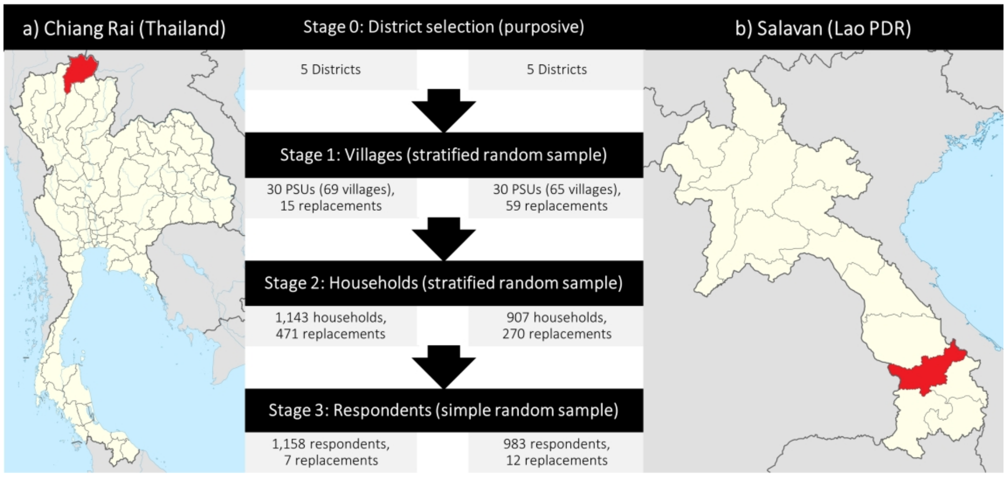

Survey sites and multi-stage sampling process. Source: Authors, adapted from Wikimedia Commons [22]. Notes: Unavailable selections at each sampling stage were substituted with a random replacement for the random samples of PSUs and household members, and with the nearest available neighbour for the interval sample of households. One PSU could contain more than one administrative village; if the first-chosen village contained less than 600 houses, then adjacent villages would be included. PSU=Primary Sampling Unit.

257x120mm (300 x 300 DPI)

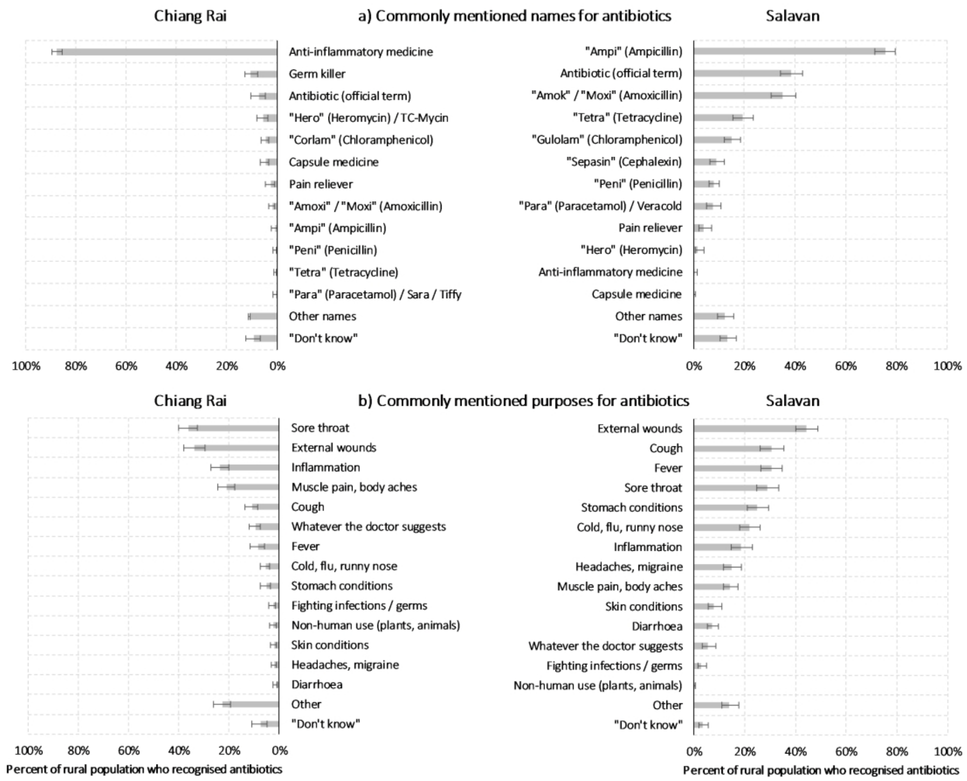

Common names and purposes for antibiotics. Source: Authors' analysis of survey data. Notes: Only including respondents who indicated that they had seen the presented medicine (i.e. common antibiotics) before. Chiang Rai: n = 1076; Salavan: n = 775. Population-weighted statistics, accounting for complex survey design. Multiple response permitted. Error bars indicate 95% confidence interval.

249x193mm (300 x 300 DPI)

Appendix

Appendix Table 1. Provincial-level estimates of rural surveys in Chiang Rai and Salavan.

	Chiang Rai	Salavan	X^2 / z-score
**Demographics**			
Number	1158	983	..
Female[a]	51.3% (44.9–57.6)	50.9% (47.0–54.9)	0.01
Age[a]	46 (13)	37 (20)	10.83***
Education (years)	6.3 (4.5)	4.4 (5.5)	2.10**
Speaking Thai / Lao	92.4% (89.9–94.2)	93.6% (91.9–94.9)	0.88
Wealth index	0.7 (0.1)	0.4 (0.2)	13.12***
Buddhist religion	81.9% (77.4–85.7)	67.5% (61.7–72.9)	16.85***
Thai/Lao nationality	95.0% (93.1–96.4)	98.8% (97.7–99.4)	18.50***
Majority ethnic group (Thai/Lao Loum)	65.2% (59.6–70.4)	56.2% (49.9–62.2)	4.73**
**Antibiotic knowledge / attitudes**			
Number	1158	983	..
Aware of antibiotics	95.7% (94.0–96.9)	86.4% (83.6–88.7)	41.47***
Aware of drug resistance[b]	74.8% (71.1–78.2)	62.5% (58.1–66.7)	18.80***
Would not buy antibiotics over the counter	57.0% (52.7–61.1)	27.7% (24.6–31.0)	115.03***
Prefers antibiotics over alternatives	61.8% (57.9–65.5)	24.8% (21.1–29.0)	151.35***
Does not keep antibiotics for future use	57.1% (53.1–61.0)	16.2% (13.2–19.8)	201.30***
Knows that antibiotic resistance can spread	9.1% (7.2–11.5)	3.4% (2.1–5.5)	15.09***
Answer score (0 to 4)	1.8 (0.9)	0.7 (1.0)	17.13***
**Illness episodes[c]**			
Number	608	356	..
Self-rated severity (1=mild, 2=medium, 3=severe)	1.6 (0.7)	1.8 (0.8)	2.43**
Duration of illness episode (days)	6.8 (7.1)	6.5 (7.9)	0.82
**Treatment-seeking behaviour[c]**			
Number	608	356	..
Public healthcare provider	29.0% (24.8–33.7)	44.8% (37.8–52.0)	14.17***
Private healthcare providers	25.0% (20.5–30.1)	23.8% (17.8–31.0)	0.08
Informal healthcare provider	8.5% (6.1–11.8)	6.9% (3.9–11.8)	0.45
Care from family or self-care	88.8% (84.3–92.2)	93.2% (88.3–96.1)	2.27
Other types of healthcare access	0.3% (0.1–1.4)	6.0% (2.8–12.3)	23.43***
**Medicine use episodes per illness[c]**			
Number	608	356	..
Medicine use episodes	2.2 (1.7)	2.5 (2.3)	1.96**
Non-antibiotic medicine use episodes	1.6 (1.2)	1.3 (1.4)	1.98**
Antibiotic use episodes	0.2 (0.5)	0.4 (0.7)	3.51***
Potential antibiotic use episodes	0.4 (0.9)	0.9 (1.8)	4.32***
Antibiotic use episodes per illness from public sources	0.1 (0.3)	0.1 (0.4)	0.78
Antibiotic use episodes per illness from private sources	0.1 (0.3)	0.2 (0.5)	1.65*
Antibiotic use episodes per illness from informal sources	0.0 (0.2)	0.1 (0.4)	1.69*
Antibiotic / potential antibiotic use episodes from public sources	0.3 (0.8)	0.3 (1.1)	0.57
Antibiotic / potential antibiotic use episodes from private sources	0.2 (0.6)	0.7 (1.5)	3.11***
Antibiotic / potential antibiotic use episodes from informal sources	0.1 (0.3)	0.2 (0.7)	2.33**

Source: Authors' analysis of survey data.

Notes: Population-weighted statistics, accounting for complex survey design. Not applicable categories indicated with ".." Group comparison using X^2 tests for binary and Wilcoxon rank-sum tests for non-normally distributed variables.

*p < 0.1, **p < 0.05, ***p < 0.01.

a. Due to population weighting, samples reflect the same sex and age profiles as the respective censuses.

b. Comparing Thai "due yah" with the combined Lao "due yah" and "lueng yah."

c. Completed illnesses experienced by respondent or child under their supervision, excluding incomplete episodes.

For peer review only

Supplemental Material

Figure A1. Trend of AMR-related social sciences and multidisciplinary publications, 1970–2017.

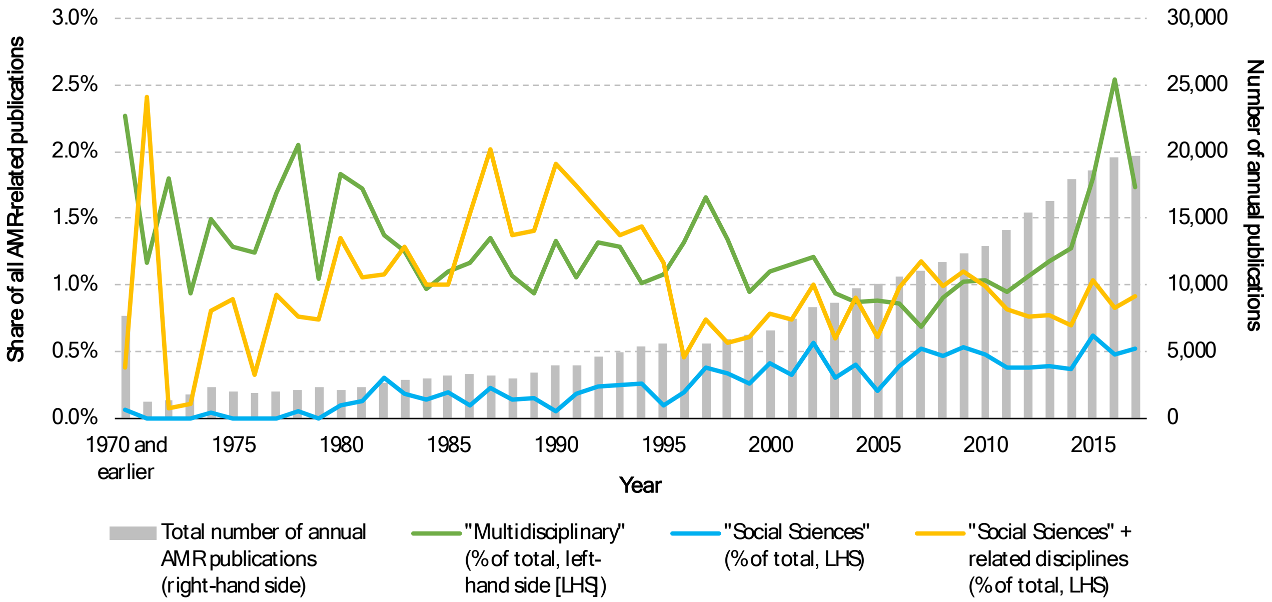

Source: Authors, based on Elsevier B.V. [15].

Notes: Data until 2018, as of 9 October 2018. Disciplines as reported by Scopus database, with "'Social Sciences' + related disciplines" providing upper bound of social science publications including Arts and Humanities;" "Business, Management and Accounting;" "Decision Sciences;" "Economics, Econometrics and Finance;" and "Psychology" alongside "Social Sciences." Based on search query [TITLE-ABS-KEY ("antibiotic resistance" OR "drug resistance" OR "antimicrobial resistance" OR "AMR")]. Total number of publications as of 9 October 2018 was 347,511, of which 66.0% arose from "Medicine;" 28.3% from "Biochemistry, Genetics and Molecular Biology;" and 18.2% from "Immunology and Microbiology" (multiple categories per publication possible.) "Social Sciences" average during the period was 0.4% (0.9% for "'Social Sciences' + related disciplines"). LHS=left-hand side.

Table A1. Variable descriptions.

	Variable	Description
**Demographic attributes**	Female	Binary variable: Sex of respondent (R); [1] if female.
	Age	Continuous variable: Age in years.
	Education	Continuous variable: Completed years of formal education.
	Speaking Thai / Lao	Binary variable: [1] if R reported ability to communicate in main language (irrespective of reading and writing).
	Wealth index	Continuous variable: Average of 17 household assets and amenities on scale from [0] to [1].
	Buddhist religion	Binary variable: [1] if R belongs to the majority religion (Buddhism in both sites).
	Thai/Lao nationality	Binary variable: [1] if R has Thai (Chiang Rai) or Lao (Salavan) nationality.
	Majority ethnic group	Binary variable: [1] if R belongs to the majority ethnic group Thai (Chiang Rai) or Lao Loum (Salavan).
**Antibiotic knowledge / attitudes**	Aware of antibiotics	Binary variable: [1] if R recognised images of antibiotic capsules that are common in the field site and, if not, the most common translation of antibiotics as "anti-inflammatory drug" ("ยาแก้อักเสบ" or "*yah kae ak seb*") in Thai and "germ resister" ("ຢາຕ້ານເຊື້ອ" or "*yah dtan suea*") in Lao. Additional categorical variables (coded ex ante and ex post) recorded the names and purposes that the respondent reported following recognition of the medicine.
	Aware of drug resistance	Binary variable: [1] if R recognised the local terms for "drug resistance." In Thai, "drug resistance" was translated as "ดื้อยา" ("*due yah*"). Lao has two translations of which "ດື້ຢາ" ("*due yah*") is the formal term and "ລື້ງຢາ" ("*lueng yah*") is a more colloquial but broader expression (both translations were asked separately). Additional categorical variables (coded ex ante and ex post) recorded the interpretations of each term.
	Would not buy antibiotics over the counter	Binary variable: [1] if answer to question "Is there any situation for which you would buy this medicine?" corresponded to FAO/OIE/WHO message "When using antibiotics: follow professional advice" (field coded based on survey training manual) [1].
	Prefers alternatives over antibiotics	Binary variable: [1] if answer to question "Do you prefer other remedies such as herbs or cough syrup to this medicine for sore throat?" corresponded to FAO/OIE/WHO message "Ensure medicines are only used when necessary" (field coded) [1].
	Does not keep antibiotics for future use	Binary variable: [1] if answer to question "If you were prescribed this medicine by a doctor and did not finish the course, would you keep it for future use?" corresponded to FAO/OIE/WHO message "When using antibiotics: never share medicines or use leftover drugs to treat a different illness" (field coded) [1].
	Knows that antibiotic resistance can spread	Binary variable: [1] if answer to question "Can your '*due yah*' (drug resistance) spread to other people, for example if you sneeze on them?" corresponded to FAO/OIE/WHO message "antimicrobial resistance can affect us all" (field coded) [1].
	Answer score	Continuous variable: Number of preceding answers ([0] to [4]) corresponding to FAO/OIE/WHO material [1].
**Illness episodes**	Self-rated severity	Ordinal variable: [1] if illness is reported as "mild;" [2] as "moderate;" [3] as "severe."
	Duration of illness episode	Continuous variable: Total duration of illness episode in days, calculated as sum of duration of individual steps in episode.
**Treatment-seeking behaviour**	Public healthcare provider	Binary variable: [1] if R reported accessing health centre or hospital during illness episode.
	Private healthcare provider	Binary variable: [1] if R reported accessing private clinic, hospital, or pharmacy.
	Informal healthcare provider	Binary variable: [1] if R reported accessing grocery store or traditional healer.
	Care from family or self-care	Binary variable: [1] if R reported self-treatment or care from family member or friend.
	Other types of healthcare access	Binary variable: [1] if R reported accessing other healthcare provider (e.g. village health volunteer).
	Medicines use episodes	Continuous variable: Number of reported medicine use episodes per illness, categorised into types of medicine (coded ex post into non-antibiotic medicine, antibiotics, and potential antibiotics) and sources of access (public, private, informal healthcare provider). Note that the actual amount of medicine used during each "medicine use episode" is likely to vary systematically across formal and informal healthcare providers, with "episodes" from the latter typically containing only a small number of pills and capsules for immediate treatment of symptoms.

Source: Authors.

For peer review only

Table A2. Correlation matrixes of independent variables in regression models.

	Would not buy antibiotics over the counter	Prefers antibiotics over alternatives	Does not keep antibiotics for future use	Knows that antibiotic resistance can spread	Aware of antibiotics	Aware of drug resistance	Female	Age	Education (years)	Speaking Thai / Lao	Wealth index	Buddhist religion	Thai/Lao nationality	Majority ethnic group (Thai/Lao Loum)	Self-rated severity	Log of duration of illness episode (days)
**Chiang Rai**																
**Would not buy antibiotics over the counter**	1.00															
**Prefers antibiotics over alternatives**	0.05	1.00														
**Does not keep antibiotics for future use**	0.25***	0.16**	1.00													
**Knows that antibiotic resistance can spread**	0.01	-0.03	0.02	1.00												
**Aware of antibiotics**	0.03	0.05	0.00	0.07	1.00											
**Aware of drug resistance**	-0.02	0.02	-0.06	0.09	0.17***	1.00										
**Female**	0.13	-0.02	-0.02	-0.02	-0.03	-0.01	1.00									
**Age**	0.00	-0.03	0.10	0.03	-0.09	-0.11	-0.03	1.00								
**Education (years)**	0.02	0.10	-0.01	0.03	0.13	0.37***	-0.18***	-0.50***	1.00							
**Speaking Thai / Lao**	0.04	0.04	0.04	0.02	0.23***	0.32***	-0.06	-0.08	0.26***	1.00						
**Wealth index**	0.05	0.09	-0.03	0.04	0.07	0.28***	-0.08	0.12	0.29***	0.33***	1.00					
**Buddhist religion**	-0.04	0.02	-0.03	0.08	0.02	0.26***	0.00	0.17***	0.19***	0.19***	0.27***	1.00				
**Thai/Lao nationality**	0.04	0.03	0.01	0.04	0.12	0.16**	-0.06	0.02	0.15**	0.32***	0.22***	0.24***	1.00			
**Majority ethnic group (Thai/Lao Loum)**	-0.02	0.04	0.00	0.04	0.12	0.33***	-0.03	0.21***	0.26***	0.32***	0.43***	0.59***	0.27***	1.00		
**Self-rated severity**	0.04	0.09	0.02	-0.01	0.03	-0.01	0.00	-0.02	-0.01	-0.03	-0.01	-0.14*	0.05	-0.15**	1.00	
**Log of duration of illness episode (days)**	0.02	0.06	-0.03	-0.04	0.03	0.05	0.09	0.04	-0.07	-0.04	0.00	-0.02	0.02	-0.06	0.36***	1.00
**Salavan**																
**Would not buy antibiotics over the counter**	1.00															
**Prefers antibiotics over alternatives**	-0.16	1.00														
**Does not keep antibiotics for future use**	-0.01	0.01	1.00													
**Knows that antibiotic resistance can spread**	0.00	0.03	0.10	1.00												
**Aware of antibiotics**	-0.23***	0.04	-0.07	0.01	1.00											
**Aware of drug resistance**	-0.19**	0.13	-0.03	0.14	0.30***	1.00										
**Female**	-0.01	-0.08	0.02	0.06	-0.06	0.06	1.00									
**Age**	-0.03	0.03	0.02	0.03	0.13	0.15	-0.09	1.00								
**Education (years)**	-0.13	0.13	-0.02	0.14	0.20*	0.36***	-0.23***	-0.19**	1.00							
**Speaking Thai / Lao**	-0.27***	0.15	0.03	0.01	0.26***	0.25***	-0.28***	0.11	0.34***	1.00						
**Wealth index**	-0.33***	0.16	0.04	-0.06	0.28***	0.38***	-0.02	0.08	0.40***	0.36***	1.00					
**Buddhist religion**	-0.42***	0.17	0.02	0.00	0.29***	0.48***	0.05	0.15	0.26***	0.44***	0.62***	1.00				
**Thai/Lao nationality**	0.05	-0.07	0.07	0.02	-0.05	-0.02	-0.02	-0.19**	0.11	-0.05	-0.03	-0.07	1.00			
**Majority ethnic group (Thai/Lao Loum)**	-0.32***	0.16	0.03	0.03	0.30***	0.53***	0.07	0.16	0.32***	0.36***	0.55***	0.76***	0.03	1.00		
**Self-rated severity**	0.19**	0.02	0.07	0.01	-0.12	-0.02	0.11	-0.01	-0.02	-0.08	-0.05	-0.13	0.10	-0.08	1.00	
**Log of duration of illness episode (days)**	0.13	0.07	0.04	0.04	-0.02	-0.04	0.11	0.07	-0.08	-0.07	0.02	-0.05	0.10	-0.09	0.21***	1.00

Source: Authors' analysis of survey data.
Notes: Unweighted statistics.
*p < 0.1, **p < 0.05, ***p < 0.01.

For peer review only

Table A3. Characteristics of individuals who received antibiotics from public, private, and informal sources.

	Chiang Rai			X² / z-score[c]			Salavan			X² / z-score[c]		
	**Public antibiotic access**	**Private antibiotic access**	**Informal antibiotic access**	**Publ. vs. priv.**	**Publ. vs. inf.**	**Priv. vs. inf.**	**Public antibiotic access**	**Private antibiotic access**	**Informal antibiotic access**	**Publ. vs. priv.**	**Publ. vs. inf.**	**Priv. vs. inf.**
Number	93	115	35	200	110	132	157	38	41	179	178	77
**Demographics**												
Female	53.8% (40.3–66.8)	59.6% (46.6–71.4)	56.9% (34.2–77.1)	0.54	0.11	0.05	71.5% (63.8–78.2)	41.0% (23.5–61.1)	71.8% (51.2–86.1)	9.13***	0.07	6.63**
Age	43 (13)	48 (14)	40 (11)	1.41	0.55	3.54***	35 (19)	33 (12)	39 (18)	0.42	0.96	0.94
Education (years)	6.2 (4.9)	5.8 (5.2)	8.0 (3.8)	0.30	1.18	2.14**	4.7 (6.3)	8.1 (5.9)	5.9 (5.5)	4.75***	0.81	1.48
Speaking Thai / Lao	92.3% (81.9–96.9)	85% (73.7–92)	98% (86.1–99.7)	2.04	1.50	4.63**	92.7% (89.3–95.1)	100.0% (100.0–100.0)	100.0% (100.0–100.0)	7.80***	4.96**	..
Wealth index	0.6 (0.1)	0.7 (0.1)	0.7 (0.1)	2.42**	3.48***	2.52**	0.4 (0.2)	0.5 (0.1)	0.5 (0.2)	3.86***	0.86	1.27
Buddhist religion	78.5% (64.8–87.8)	77.2% (63.9–86.6)	89.0% (74.4–95.8)	0.03	1.44	2.14	66.5% (58.3–73.8)	78.9% (52.9–92.5)	79.2% (58.9–91.0)	1.05	2.61	<0.01
Thai/Lao nationality	97.1% (89.8–99.2)	95.9% (88.8–98.5)	98% (86.1–99.7)	0.14	0.03	0.28	99% (92.9–99.9)	100.0% (100.0–100.0)	97.6% (84.0–99.7)	0.38	0.66	24.41***
Majority ethnic group (Thai/Lao Loum)	58.8% (45.3–71.1)	64.6% (52.3–75.3)	81.9% (65.8–91.4)	0.60	7.36***	2.84*	58.6% (48.9–67.7)	69.9% (45.6–86.6)	68.2% (44.4–85.2)	0.71	0.67	0.03
**Antibiotic knowledge / attitudes**												
Aware of antibiotics	97.5% (90.4–99.4)	95.7% (88.6–98.4)	93.3% (64.5–99.1)	0.42	2.70	0.24	88.7% (82.3–92.9)	92.7% (72.2–98.4)	100.0% (100.0–100.0)	1.21	5.50**	2.32
Aware of drug resistance[a]	75.0% (62.5–84.3)	76.5% (64.9–85.1)	90.2% (76.4–96.4)	0.06	3.35*	3.63*	66.3% (57.8–73.8)	84.7% (61.1–95.1)	79.5% (61.3–90.5)	2.94*	2.22	0.18
Links drug resistance to AMR concepts[b]	1.7% (0.3–9.5)	5.4% (2.0–13.6)	1.3% (0.2–9.0)	1.39	0.01	5.17**	4.4% (2.0–9.3)	9.0% (1.9–33.5)	13.4% (5.6–28.7)	0.89	4.92**	0.27
Would not buy antibiotics over the counter	62.0% (48.2–74.1)	62.1% (48.3–74.2)	42.0% (22.0–65.0)	<0.01	4.19**	4.44**	44.5% (35.9–53.5)	18.3% (9.3–32.7)	23.3% (12.3–39.6)	13.92***	24.99***	0.62
Prefers antibiotics over alternatives	62.7% (49.6–74.2)	50.2% (36.8–63.6)	70.3% (46.2–86.7)	2.48	1.17	3.21*	22.8% (16.8–30.2)	33.7% (19.5–51.7)	29.3% (13.7–52.0)	2.52	0.37	0.36
Does not keep antibiotics for future use	54.3% (41.2–66.8)	53.9% (41.7–65.6)	38.1% (18.9–62.0)	<0.01	0.88	3.35*	21.1% (14.2–30.2)	14.0% (4.6–35.4)	13.9% (5.2–32.1)	0.52	2.07	<0.01
Knows that antibiotic resistance can spread	8.1% (3.9–16.2)	10.9% (5.7–19.9)	6.5% (1.6–23.2)	0.51	0.25	0.53	1.9% (0.5–6.6)	1.8% (0.2–12.4)	0.0% (0.0–0.0)	<0.01	1.90	0.95
Answer score (0 to 4)	1.9 (0.9)	1.8 (0.9)	1.6 (0.8)	0.54	1.72*	1.05	0.9 (1.1)	0.7 (0.7)	0.7 (0.6)	1.86*	1.87*	0.05

Source: Authors' analysis of survey data.

Notes: Including antibiotics and unconfirmed medicines that may include antibiotics. Illness-level data, including only completed illnesses experienced by respondent or a child under their supervision. Population-weighted statistics, accounting for complex survey design. Multiple types of healthcare access per individual and illness episode possible.

a. Comparing Thai "due yah" with the combined Lao "due yah" and "lueng yah."

b. Corresponding to interpretation of "drug resistance" as "Reference to antibiotics, drug-resistant germs" in Exhibit 6.

c. X^2 and Wilcoxon rank-sum tests, omitting simultaneous antibiotic access from more than one type of healthcare provider, which was the case in 82/958 [8.6%] of all pairwise comparisons of antibiotic access.

For peer review only

1. Village Checklist (GPS coordinates of village and facilities) (to be completed by supervisor)

What kind of facility would you like to record?

A. District Number		[*code entered automatically*]
B. Village Number		[*code entered automatically*]
C. Village centre	a) Latitude	[*coordinates entered automatically*]
	b) Longitude	[*coordinates entered automatically*]
D. Village head's house	a) Latitude	[*coordinates entered automatically*]
	b) Longitude	[*coordinates entered automatically*]
E. Local shop	a) Latitude	[*coordinates entered automatically*]
	b) Longitude	[*coordinates entered automatically*]
F. Market	a) Latitude	[*coordinates entered automatically*]
	b) Longitude	[*coordinates entered automatically*]
G. Temple	a) Latitude	[*coordinates entered automatically*]
	b) Longitude	[*coordinates entered automatically*]
H. School	a) Latitude	[*coordinates entered automatically*]
	b) Longitude	[*coordinates entered automatically*]
I. Bus stop	a) Latitude	[*coordinates entered automatically*]
	b) Longitude	[*coordinates entered automatically*]
J. Health facility<br>Specify (public, private, pharmacy, local store, traditional healer, etc.):<br>_________________	a) Latitude	[*coordinates entered automatically*]
	b) Longitude	[*coordinates entered automatically*]
	c) Who is staffing the facility?	Total staff: ___<br>Staff at time of visit: ________
	d) Does the provider have antibiotics available?	Yes ....1<br>No 0

For peer review only

Interview data [*Record observation*]

i. District Number		[*code entered automatically*]
ii. PSU Number		[*code entered automatically*]
iii. Household number		Number: ______
iv. Household coordinates	a) Latitude	[*coordinates entered automatically*]
	b) Longitude	[*coordinates entered automatically*]

v. What type is this house most similar to?

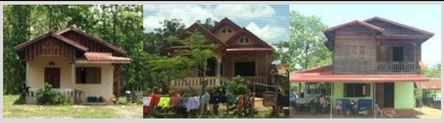
1…

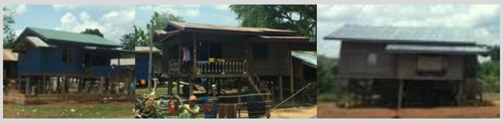
2…

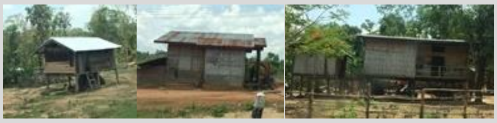
3…

vi. Time of visit	a) First visit	[*time entered automatically*]
	b) Second visit	[*time entered automatically*]

List all persons aged 18+ years in household

Hello, I'm a researcher working for the Mahidol-Oxford Tropical Medicine Research Unit. We are interested in the lives and health behaviours of villagers across Thailand and Lao PDR. We are selecting participants randomly and would like to choose one or two members of your household. In order to choose and ask them to participate, could you please tell us who lives here? [provide PIS on request]

[*1 respondent per every 5 household members will be selected randomly from this list*]

Name	Nickname	Sex (M / F)	Age	Available for interview today? (Yes / No)

Statement of consent (Respondent will receive participant information sheet and verbal consent will be taken)
Thank you for participating. You will receive a small token of gratitude for your participation at the end of the interview.

vii.Date of interview	[*date entered automatically*]
viii.Time of interview begin	[*time entered automatically*]
ix.Respondent name	Respondent name: ______________________________
x.Interviewer code	[*code entered automatically*]

Part I: Personal and Household Characteristics
Let us begin with a few questions about yourself and your household.

1. [*record as observed*] Sex		Female........................ 1<br>Male 0
2. How old are you? [*in years*] [*If respondent cannot give exact age, ask for approximate age and code in range: 18-24, 25-34, 35-44, 45-59, 60 and older*]		Age in years: _______
3. Please indicate what kind of work you do. If you have more than one occupation at one time or throughout the year, please begin with the one in which you spend the most time and name up to three. If you do not have an occupation, please also mention whether you are still a student, retired, or unemployed.	a) Main occupation	Occupation: _________
	b) Side occupation	Occupation: _________
	c) Side occupation	Occupation: _________
4. What is your mother tongue?		Mother tongue: ________________
5. [In Thailand:] Can you speak Thai? [In Laos:] Can you speak Lao?		Yes .... 1<br>No 0
6. What is the highest grade of schooling that you completed?<br>[*excluding informal education and pre-school education such as nursery and kindergarten, but including grade school, high school, vocational training, tertiary education, etc.*]		Highest grade: ___
7. Are you the head of your household?		Yes ....1<br>No 0
7.1. [*if no*] What is the name of your household head?		Name: __________

8. What is your current marital status?	Never married........ 1<br>Currently married........ 2<br>Cohabiting........ 3<br>Separated / divorced........ 4<br>Widowed........ 5	
9. Are there any close family members of yours [children, spouse, siblings, parents] who live elsewhere? [*select "no" if not applicable*]	9.1. Do your parents live outside of this village? [*do not count parents-in-law*]	At least 1 person outside village .... 1<br>All inside village / not applicable.... 0
	9.2. Does your spouse live outside of this village?	At least 1 person outside village .... 1<br>All inside village / not applicable.... 0
	9.3. Do you have siblings who live outside of this village? [*do not count brothers-in-law and sisters-in-law*]	At least 1 person outside village .... 1<br>All inside village / not applicable.... 0
	9.4. Do you have children who live outside of this village?	At least 1 person outside village .... 1<br>All inside village / not applicable.... 0
Part II: Social Networks [for network census villages only]<br>I will now ask you some questions about your interactions with other people within and outside of your village.		
10. [Round I of network survey only] Where do you spend most of your time interacting with other people from your village?	a) Field: ___<br>b) Temple: ___<br>c) Local store: ___<br>d) Market: ___<br>e) Children's schools: ___<br>f) Home: _____<br>g) Workplace: ___<br>h) Village event/s: _______<br>i) Other site: ___	

For peer review only

11. **[Round I of network survey only]** Outside your household, with whom do you interact on a regular basis? (May be anyone from both inside and outside of the village, and through any platform which might not require a face-to-face interaction)

	a) What is the nickname of the person?	b) How is this person related to you? [*give examples if respondent is unsure about answer categories*]	c) What is the sex of this person?	d) Where does this person live?	e) What is the name of the household head of this person?	f) How often do you interact with this person?	g) How do you interact with this person? [*Mark all that apply*]	h) Do your conversations relate to health and well-being?
**11.1.** Contact 1	Nickname _____<br>Name _____	Spouse................ 1<br>Parent................ 2<br>Child .... 3<br>Sibling................ 4<br>Other relative .... 5<br>Neighbour................ 6<br>Friend (if not neighbour)................ 7<br>Other villager................ 8<br>Other (specify) _................ 9	Female...1<br>Male 0	In village .... 1<br>(specify: _____)<br>Outside village .. 2	Name of household head<br>________	Daily or more often ....4<br>Weekly or few times/week ....3<br>Monthly or few times/month ...2<br>Yearly or few times/year ....1<br>Less often or never 0	Face-to-face...1<br>Voice call........2<br>Messenger .....3<br>Other (specify) _____ 4	Yes .... 1<br>No 0
**11.2.** Contact n	Nickname<br>Name	1 2 3 4 5 6 7 8 9	1 0	1 2	Name	0 1 2 3 4	1 2 3 4	1 0

**11a. [Round II of network survey only]** When we last visited you, you told us that you interact regularly with [*names*]. Has anything changed since last time?	Yes........................1 → **[update social network question 11]**<br>No.........................0
**11i. [Round I of network survey only]** Is there anybody in your household with whom you talk about health and well-being? [*Mark all that apply*]	[*mark all names from household roster that apply*]

For peer review only - http://bmjopen.bmj.com/site/about/guidelines.xhtml

[*For network survey village respondents in Round 2*]<br>**12.** An education activity has recently taken place in your village.	
**12.1.** Did you participate in any of the activities?	Yes .... 1<br>Yes, but not throughout .... 2<br>No .... 3<br>Don't know / prefer not to say 4
**12.2.** Did you talk with anybody about the activity in your village?<br>[*"Talking" can involve any conversation including asking for information, informing about the educational activity, or discussing it (regardless of actual attendance)*]	a) Nickname 1: ______ b) Full name 1: ______ c) Relationship 1: 1 2 3 4 5 6 7<br>a) Nickname n: ______ b) Full name n: ______ c) Relationship n: 1 2 3 4 5 6 7<br>[*Relationship codes*]<br>Household member .... 1<br>Family member outside HH .... 2<br>Other relative .... 3<br>Neighbour .... 4<br>Friend other than neighbour .... 5<br>Other villager .... 6<br>Other (specify) _ 7
[*If respondent indicates conversation in Q 12.2*]<br>**12.3.** What subjects did you talk about in respect to the activity?<br>[*mark all that apply*]	Going to doctor when sick .... 1<br>Anti-inflammatories/antibiotics .... 2<br>Germs .... 3<br>Using medicines correctly .... 4<br>Activity in general .... 5<br>Games/awards .... 6<br>Song/Story/Play .... 7<br>Money/compensation .... 8<br>Other (specify) ____ 9

Part III: Healthcare Seeking Thank you for this. Now we come to a part where I will ask you some questions about health and health providers around here.

**13.** I would now like to ask you about the sources of health advice and medicine or other treatment that are available to you. Please think about all the places where you can go to get advice, treatment, or drugs if you (or your children) are sick.<br><br>Do you consider the following providers when you (or your children) feel unwell?<br><br>[*Mark all that apply*]	**13.1.** Drug dispensary, other local store selling medicine	Consultation .... 1<br>Medical advice .... 2<br>Access to medicine .... 3<br>Other reason(s) .... 4<br>Don't consider this provider .... 98<br>Don't know such a provider 99
	**13.2.** Traditional healer	Consultation .... 1<br>Medical advice .... 2<br>Access to medicine .... 3<br>Other reason(s) .... 4<br>Don't consider this provider .... 98<br>Don't know such a provider 99
	**13.3.** Pharmacist	Consultation .... 1<br>Medical advice .... 2<br>Access to medicine .... 3<br>Other reason(s) .... 4<br>Don't consider this provider .... 98<br>Don't know such a provider 99
	**13.4.** Private clinic	Consultation .... 1<br>Medical advice .... 2<br>Access to medicine .... 3<br>Other reason(s) .... 4<br>Don't consider this provider .... 98<br>Don't know such a provider 99
	**13.5.** Private hospital	Consultation .... 1<br>Medical advice .... 2<br>Access to medicine .... 3<br>Other reason(s) .... 4<br>Don't consider this provider .... 98<br>Don't know such a provider 99
	**13.6.** Health volunteer	Consultation .... 1<br>Medical advice .... 2<br>Access to medicine .... 3<br>Other reason(s) .... 4<br>Don't consider this provider .... 98<br>Don't know such a provider 99
	**13.7.** Public primary care unit	Consultation .... 1<br>Medical advice .... 2<br>Access to medicine .... 3<br>Other reason(s) .... 4<br>Don't consider this provider .... 98<br>Don't know such a provider 99

For peer review only - http://bmjopen.bmj.com/site/about/guidelines.xhtml

	**13.8.** Public hospital	Consultation .... 1<br>Medical advice........ 2<br>Access to medicine........ 3<br>Other reason(s) .... 4<br>Don't consider this provider........ 98<br>Don't know such a provider 99
	**13.9.** Other providers or Internet? Specify: __________	Consultation .... 1<br>Medical advice........ 2<br>Access to medicine........ 3<br>Other reason(s) .... 4<br>Don't consider this provider........ 98<br>Don't know such a provider 99

For peer review only

For peer review only - http://bmjopen.bmj.com/site/about/guidelines.xhtml

14. Now if you think again, is there anyone else with whom you talk about health?								
	a) What is the nickname of the person?	b) What is the full name of the person?	c) How is this person related to you?<br><br>[*give examples if respondent is unsure about answer categories*]	d) What is the sex of this person?	e) Where does this person live?	f) What is the name of the household head of this person?	g) How often do you interact with this person?	h) How do you interact with this person?<br><br>[*Mark all thatapply*]
**14.1.** Contact 1	Name ______	Name ______	Spouse.......... 1<br>Parent .... 2<br>Child .... 3<br>Sibling.......... 4<br>Other relative .... 5<br>Neighbour.......... 6<br>Friend (if not neighbour).......... 7<br>Other villager.......... 8<br>Other (specify) _.......... 9	Female ..1<br>Male 0	In village .... 1<br>(specify: _____)<br><br>Outside village .. 2	Name of household head<br>________	Daily or more often ....4<br>Weekly or few times/week ....3<br>Monthly or few times/month ...2<br>Yearly or few times/year ....1<br>Less often or never..........0	Face-to-face.......... 1<br>Voice call.......... 2<br>Messenger .... 3<br>Other (specify) _____ 4
**14.2.** Contact n	Name	Name	1 2 3 4 5 6 7 8 9	1 0	1 2	Name	0 1 2 3 4	1 2 3 4

For peer review only

**15.** Did you or a child in your household have an acute illness (not a chronic, long-term condition that comes again and again) or an accident in the last two months? If yes, I will ask you about these illnesses one-by-one. [*if no, continue with Question 19*]	No........................0 ➔ [*Q 16*]<br>Yes......................1 ⬇
[*if yes*:]<br>**15.a** [Confirm if this episode is for respondent or child]	Respondent ....1 ➔ [*Q 15.1*]<br>Child 2
**15.b** How old is the child?	Age in years: _______
**15.c** Is the child female or male	Female ....1<br>Male 0
**15.1.** Can you please describe the symptoms or problem in your own words?	Description of condition:<br>________________________
**15.2.** Did [you / the child] receive a diagnosis of the illness from any medical provide, friend, or internet source?<br><br>If so, can you please describe the diagnosis of the illness if you received any and where [you / the child] received it? [***note: the diagnosis might be given by any medical provider including untrained and informal. Record all diagnoses if more than one.***]	a) Diagnosis 1: ___________ b) Medical provider 1: 1 2 3 4 5 6 7 8<br>a) Diagnosis n: ___________ b) Medical provider n: 1 2 3 4 5 6 7 8<br><br>[*Response codes*]<br>Drug dispensary, other local store selling medicine ....1<br>Traditional healer....................2<br>Pharmacist ....3<br>Private clinic ....4<br>Private hospital ....5<br>Primary care unit ....6<br>Public hospital....................7<br>Other providers or Internet? Specify: ________....................8
**15.3.** When did [you / the child] experience the accident/discomfort (for the first time)	Onset: ___ days / ______ weeks / _____ months ago
**15.4.** Would you describe the illness/accident as "mild," "moderate," or "severe"?	Mild....................1<br>Moderate....................2<br>Severe 3

15.5. Can you please explain the stages of the treatment? I will ask you step-by-step what you did, starting from the moment [you / the child] first experienced a discomfort.

	**15.5.1.** Step 1 (detection)	Step n
a) What kind of help or treatment did you get at this stage?<br>[*if unsure, specify*]	Ignored /did nothing ....1<br>Self-care (sleep, rest, medicine at home) ....2<br>Care from family and friends (full-time)....................3<br>Treated/consulted at a traditional healer ....4<br>Treated/cons. at a pharmacist....................5<br>Treated/cons. at shop selling drugs....................6<br>Treated/cons. at priv. clinic/hospital ....7<br>Treated/cons. at primary care unit....................8<br>Treated/cons. at a gvt. Hospital....................9<br>Other (specify) _____ 10	1<br>2<br>3<br>4<br>5<br>6<br>7<br>8<br>9<br>10
b) Where did this activity take place?	At home ....1<br>Less than 10 min. from home....................2<br>10 to 29 min. ....3<br>30 to 59 min. ....4<br>60 to 119 min. ....5<br>2 hours or more from home....................6	1<br>2<br>3<br>4<br>5<br>6
c) How did [you / the child] get to the place of the activity? [*select "at home" according to prior responses*]	At home .... 1<br>Walk .... 2<br>Own bicycle .... 3<br>Own motorcycle / Three-wheeler.................... 4<br>Own car / four-wheeler .... 5<br>Taxi or other hired ride.................... 6<br>Public transport.................... 7<br>Other (specify) ___ 8	1<br>2<br>3<br>4<br>5<br>6<br>7<br>8
d) How long did this stage last?<br>[*let respondent choose category; if <1 day, code "1" day*]	Duration:<br>___ days<br>___ weeks<br>___ months	___ days<br>___ weeks<br>__ months
e) Can you please name or describe all the medicines that you received or were prescribed during this step?<br><br>[*include medicine stored at home if "self-care at home"*] [*continue for all medicines received, then complete Questions g to k for each medicine individually*] 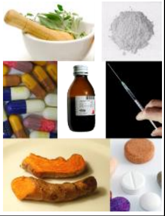	Medicine 1:<br>Name/description: _____<br><br>Medicine n:<br>Name/description: _____	Medicine 1<br><br>Medicine n
f) For how long did [you / the child] take the medicine?<br>[*let respondent choose category; if more than one repeated episode, indicate total duration*]<br>[*for each medicine individually*]	Duration:<br>___ days<br>___ weeks<br>___ months	___ days<br>___ weeks<br>__ months

For peer review only - http://bmjopen.bmj.com/site/about/guidelines.xhtml

g) How often per day did [you / the child] take the medicine?<br><br>[*calculate into daily use according to respondent's chosen frequency*]<br>[*for each medicine individually*]	Frequency: ___ times daily	___ times daily
h) What dosage did [you / the child] normally take?<br>[*let respondent choose category according to type of medicine*]<br>[*for each medicine individually*]	Dosage<br>___ tablets / capsules<br>___ drops (for liquid medicine)<br>___ spoons (for liquid medicine)<br>_____ shots/injections (for intravenous medicine)<br>per time administered	___ tablets<br>___ drops<br>___ spoons<br>___ shots
i) Did [you / the child] take the medicine exactly as it was recommended to you by the person who prescribed/sold them<br>[*for each medicine individually*]	Yes....1<br>No....0<br>Did not receive advice....9<br>Don't know....99	1<br>2<br>9<br>99
j) Did [you / the child] finish the medicine?<br>[*for each medicine individually*]	Yes....1<br>No....0	1<br>0
k) Did you or anybody else use a mobile phone during this stage <u>in connection with your condition</u>? [*if no, go to next step*]	Yes....1<br>No....0 ➔ [*next step*]	1<br>0
l) What was the purpose of using the mobile phone?<br><br>[*Mark all that apply*]	Ask for advice....1<br>Call for treatment....2<br>Arrange transport....3<br>Appointment....4<br>Reassure family/friends....5<br>Ask for money/supplies....6<br>Provider contacting me for information....7<br>Treatment reminder....8<br>Other (specify) _ 9	1<br>2<br>3<br>4<br>5<br>6<br>7<br>8<br>9
m) Which mobile phone functions did you or anybody else use?<br>[*Mark all that apply*]	Call....1<br>SMS....2<br>Internet, messenger....3<br>Alarm, calendar, reminder, etc. ....4<br>Other (specify) _ 5	1<br>2<br>3<br>4<br>5

**15.6.** [Have you / has the child] now recovered from the illness/accident?	Yes....1<br>No....0
**15.7.** Was anybody of your personal relationships involved in providing advice or help <u>during the illness</u>? [*record up to ten names*]	Yes....1<br>No....0
[*For district survey*]<br>**15.7.b** How are these people related to you? [*Mark all that apply*]	Spouse.... 1<br>Parent.... 2<br>Child.... 3<br>Sibling.... 4<br>Other relative.... 5<br>Neighbour.... 6<br>Friend (if not neighbour).... 7<br>Other villager.... 8<br>Other (specify) _.... 9
**15.7.c** What kind of support did they provide? [*Mark all that apply*]	Providing healthcare/attending.... 11<br>Providing advice.... 12<br>Providing medicine.... 13<br>Lending/granting money.... 21<br>Transportation/Lending vehicle.... 22<br>Contacting family/friends.... 23<br>Providing food.... 31<br>Helping with children/housework.... 32<br>Helping with jobs/agriculture work (feeding animals/tending crops/covering shifts, etc.) 33<br>Other (specify) _.... 99

[*For network survey*]	a) What is the name of the person?	b) How is this person related to you?	c) What kind of support was provided?<br>[*mark all that apply*]
**15.7.1.**<br>Contact 1	Name:<br>_______	Spouse....1<br>Parent....2<br>Child....3<br>Sibling....4<br>Other relative....5<br>Neighbour....6<br>Friend (if not neighbour)....7<br>Other villager....8<br>Other (specify) _....9	Providing healthcare/attending.... 11<br>Providing advice.... 12<br>Providing medicine.... 13<br>Lending/granting money.... 21<br>Transportation/Lending vehicle.... 22<br>Contacting family/friends.... 23<br>Providing food.... 31<br>Helping with children/housework.... 32<br>Helping with jobs/agriculture work (feeding animals/tending crops/covering shifts, etc.) 33<br>Other (specify) _.... 99
**15.7.2.**<br>Contact n	Name	1 2 3 4 5 6 7 8 9	11 12 13 21 22 23 31 32 33 99

**15.8.** Did you have another acute illness (not a chronic, long-term condition that comes again and again) or an accident in the last two months?<br>[*if yes, complete another sheet for Question 15*]	Yes .... 1 ➔ [*Q 15*]<br>No .... 0 ⬇
**16.** I would now like to ask you your opinion about medicine. There are no right or wrong answers, I only want to understand what you think. Consider the following medicines:	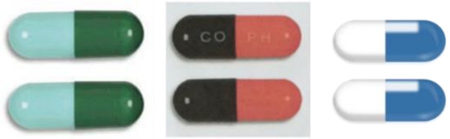
**16.1.** Have you seen these medicines before?	Yes........ 1<br>No .... 0 ➔ [*Q 16.4*]
**16.2.** What do you call this medicine?	Antibiotics ท่านเรียกยานี้ว่าอะไร........ 11<br>Anti-inflammatory ยาแก้อักเสบ........ 12<br>Germ killer ยาฆ่าเชื้อ .... 13<br>Amoxy / Amoxicillin อะม็อกซี่/อะม็อกซี่ซิลลิน .... 14<br>Sore throat medicine ยาแก้เจ็บคอ .... 15<br>Cough medicine ยาแก้ไอ .... 16<br>Pain reliever ยาแก้ปวด .... 17<br>Fever reliever ยาแก้ไข้ .... 18<br>Other (specify: _____) อื่นๆ (โปรดระบุ)........ 98<br>Germ preventer / antibiotic ຢາຕ້ານເຊື້ອ........ 21<br>Amok ຢາຕ້ານເຊື້ອ .... 22<br>Ampi ຢາແອມປີ .... 23<br>Tetra ຢາເຕຕ້າ .... 24<br>Gulolam ກູໂລລາມ........ 25<br>Sepasin ເຊພາຊິນ........ 26<br>Other (specify: __)........ 99
**16.3.** What symptoms or illnesses would you use this medicine for?	Fever .... 1<br>Cough .... 2<br>Sore throat........ 3<br>Inflammation .... 4<br>Cold, flu, runny nose .... 5<br>Diarrhoea .... 6<br>Headache .... 7<br>Stomach ache .... 8<br>Muscle pain, other aches........ 9<br>Skin diseases, rashes, lumps .... 10<br>Wounds........ 11<br>Urinary tract infections .... 12<br>Every kind of sickness........ 13<br>Whatever the doctor suggests .... 14<br>Don't know / prefer not to say .... 98<br>Other (specify: __)........ 99
**16.4.** Is there any situation for which you would buy this medicine?	Desirable attitude/knowledge .... 1<br>Undesirable attitude/knowledge .... 0<br>No attitude / refuse to answer (respondent is aware, but doesn't reveal attitude)........ 97<br>Answer does not apply to question (respondent may be aware/unaware; satisficing)...... 98<br>Not aware of this medicine (awkward, cannot answer but does not try to satisfy) 99
**16.5.** Do you prefer other remedies such as herbs or cough syrup to this medicine for [sore throat]?	Desirable attitude/knowledge .... 1<br>Undesirable attitude/knowledge .... 0<br>No attitude / refuse to answer (respondent is aware, but doesn't reveal attitude)........ 97<br>Answer does not apply to question (respondent may be aware/unaware; satisficing)...... 98<br>Not aware of this medicine (awkward, cannot answer but does not try to satisfy) 99
**16.6.** If you were prescribed this medicine by a doctor and did not finish the course, would you keep it for future use?	Desirable attitude/knowledge .... 1<br>Undesirable attitude/knowledge .... 0<br>No attitude / refuse to answer (respondent is aware, but doesn't reveal attitude)........ 97<br>Answer does not apply to question (respondent may be aware/unaware; satisficing)...... 98<br>Not aware of this medicine (awkward, cannot answer but does not try to satisfy) 99
**16.7.** Have you heard about drug resistance?<br>(**16.7a** using alternative term "lueng yah" in Lao)	Yes .... 1<br>No 2
**16.8.** What do you think is drug resistance?<br>(**16.8a** using alternative term "lueng yah" in Lao)	Bacteria are resistant to medicine .... 1<br>Antibiotics become less effective if used wrongly/too much........ 2<br>Medicine in general becomes less effective if used wrongly/too much........ 3<br>Being stubborn to take medicine .... 4<br>Being addicted to medicine........ 5<br>Drug allergy .... 6<br>Lueng yah (drug resistance) .... 7<br>Answer does not relate to drug resistance........ 8<br>Other (specify)........ 98<br>"Don't know" 99
**16.9.** Can your drug resistance ("*due yah*") spread to other people, for example if you sneeze on them?	Desirable attitude/knowledge .... 1<br>Undesirable attitude/knowledge .... 0<br>No attitude / refuse to answer (respondent is aware, but doesn't reveal attitude)........ 97<br>Answer does not apply to question (respondent may be aware/unaware; satisficing)...... 98<br>Not aware of this medicine (awkward, cannot answer but does not try to satisfy) 99

For peer review only - http://bmjopen.bmj.com/site/about/guidelines.xhtml

Part IV: Household assets
We now come to the last part. Can you please provide me with some information about your household?

Question	Response
**17.** How many rooms does this house have apart from toilet and hallways?	Number of rooms: ________
**18.** What is the electricity situation in your household on a typical day?	Power at all times, no power cuts (90-100%) .... 1<br>Power most of the time, occasional power cuts (>50%) .... 2<br>Power sometimes, frequent power cuts (<50%) .... 3<br>No electricity 4
**19.** What kind of toilet does this house have and is it shared with other people in this community? *[if more than one, choose "best" toilet] [use show card to facilitate answers]*	Unshared flush toilet (e.g. piped sewer system, septic tank, pour flush toilet) .... 1<br>Shared (flush or non-flush) toilet with other community members or public toilet 2<br>No facility, Bush, Field, or others 3
**20.** What is the drinking water source of this house and is it shared with other people in this community? [*use show card to facilitate answers*]	Water piped into house or yard .... 1<br>Water not directly piped into house or yard (e.g. well, borehole, water from spring, rainwater, tanker truck, surface water including rivers, bottled water, etc.)2
**21.** What kind of fuel does this household use for cooking?	Improved fuel source (e.g. Electricity, gas stove, etc.) .... 1<br>Unimproved fuel source (e.g. Coal / Lignite, Charcoal, Wood, Straw / Shrubs / Grass, Animal dung, Agricultural crop residue) .... 2<br>No food cooked in household 3

22. I will now ask you for some items in your household. Please tell me…

Number of items in household:		
22.1.	Have you got a *functioning* **radio** in your household? If so, how many?	__
22.2.	Have you got a *functioning* **TV** in your household? If so, how many?	__
22.3.	Have you got a *functioning* **rice cooker** in your household? If so, how many?	__
22.4.	Have you got a *functioning* **landline telephone** in your household? If so, how many?	__
22.5.	Have you got a *functioning* **mobile phone** in your household? If so, how many?	__
22.6.	Have you got a *functioning* **computer** in your household? If so, how many?	__
22.7.	Have you got a *functioning* **bicycle** in your household? If so, how many?	__
22.8.	Have you got a *functioning* **scooter, motorcycle, or tricycle** in your household? If so, how many?	__
22.9.	Have you got a *functioning* **car or truck** in your household? If so, how many?	__
22.10.	Have you got a *functioning* **tractor** in your household? If so, how many?	__
22.11.	Have you got a *functioning* **refrigerator or freezer** in your household? If so, how many?	__

23. How long does it normally take you to get to the following places?

Place	Response
23.1. How long does it take to get to the nearest market?	Less than 10 minutes .... 1<br>10 to 29 minutes .... 2<br>30 to 59 minutes .... 3<br>60 to 119 minutes .... 4<br>2 hours or more 5
23.2. How long does it take to get to the village hall or the village head's house?	Less than 10 minutes .... 1<br>10 to 29 minutes .... 2<br>30 to 59 minutes .... 3<br>60 to 119 minutes .... 4<br>2 hours or more 5
23.3. How long does it take to get to the nearest public or private doctor?	Less than 10 minutes .... 1<br>10 to 29 minutes .... 2<br>30 to 59 minutes .... 3<br>60 to 119 minutes .... 4<br>2 hours or more 5

Question	Response
**24.** What is your religion?	No religion .... 0<br>Buddhist .... 1<br>Christian .... 2<br>Muslim .... 3<br>Spirit (religious belief in Lao) .... 4<br>Other (Specify) __________ .... 5<br>Don't know 99
**25.** What is your nationality?	Thai .... 1<br>Lao .... 2<br>Myanmar/Burmese .... 3<br>Chinese .... 4<br>Other (Specify) __________ .... 9<br>Don't know 99

For peer review

**26.** What is your ethnic background?	Thai ....1<br>Tai Yai ....2<br>Akha (E-Koh) ....3<br>Pakakeryor (Karen) ....4<br>Lahu (Muser) ....5<br>Lisu (Lisaw) ....6<br>Hmong (Meaw) ....7<br>Mien (Yao) ....8<br>Burmese ....9<br>Yunnan (Jin Haw) ....10<br>Tai Lue (Tai) ....11<br><br>Lao ....21<br>Kathuic ....22<br>Bahnaric Khmer ....23<br>Tai Thai ....24<br><br>Other (Specify) ________ ....30<br>Don't know 99
xi. Interview end time	[*time entered automatically*]
Thank you very much for participating in this survey. [*give gift to respondent*]	
**Part V: Interviewer observations [to be completed by interviewer after interview]**	
xii. Was the interview completed?	Yes ....1<br>Yes, with difficulties ....2<br>No 3
xiii. Was someone else present during the interview? [*mark all that apply*]	Survey supervisor ....1<br>Other household or family member ....2<br>Medical practitioner ....3<br>Government officer ....4<br>Other (specify) _____ ....5<br>No one 0
xiv. What is your evaluation of the accuracy and trustworthiness of the informant's answers?	Very good ....1<br>Satisfactory ....2<br>Doubtful ....3<br>Very low 4
xv. Were there any unusual circumstances during the interview?	Please describe: ______________________________

For peer review only

For peer review only - http://bmjopen.bmj.com/site/about/guidelines.xhtml

INTERVIEW GUIDES – COGNITIVE INTERVIEWS FOR SURVEY TESTING

บทสัมภาษณ์ – การสัมภาษณ์กระบวนการคิดเพื่อการทดสอบแบบสอบถาม

ບົດສຳພາດ - ການສຳພາດຂະບວນການຄິດເພື່ອການທົດສອບຄຳຖາມ

Objective วัตถุประสงค์ ຈຸດປະສົງ

To understand how survey participants understand the survey questions and arrive at their answers. เพื่อเรียนรู้ว่าผู้เข้าร่วมงานวิจัยมีการทำความเข้าใจคำถามที่ใช้ในแบบสอบถามอย่างไร และมีความคิดอย่างไรก่อนตัดสินใจตอบคำถามแต่ละข้อ ເພື່ອຮຽນຮູ້ວ່າຜູ້ເຂົ້າຮ່ວມການຄົ້ນຄ້ວາມີຄວາມເຂົ້າໃຈຄຳຖາມທີ່ໃຊ້ໃນແບບສອບຖາມແບບໃດ ແລະມີຄວາມຄິດແບບໃດກ່ອນຕັດສິນໃຈຕໍ່ຄຳຖາມແຕ່ລະຂໍ້

Introduction บทนำ ບົດນຳ

<Obtaining verbal informed consent in accordance with Oral Consent Script> <ดำเนินการขอคำยินยอมโดยใช้ข้อความสำหรับอธิบายงานวิจัยและขอคำยินยอมเข้าร่วมการวิจัยด้วยวาจา> <ດຳເນີນການຂໍຄຳຍິນຍອມໂດຍໃຊ້ຂໍ້ຄວາມສຳລັບອະທິບາຍວຽກຄົ້ນຄ້ວາແລະຂໍຄຳຍິນຍອມເຂົ້າຮ່ວມການຄົ້ນຄ້ວາດ້ວຍວາຈາ>

Thank you for agreeing to participate in this study. ขอบคุณท่านสำหรับการตกลงเข้าร่วมการวิจัยครั้งนี้ ຂອບໃຈທ່ານສຳລັບການຕົກລົງເຂົ້າຮ່ວມການຄົ້ນຄ້ວາໃນຄັ້ງນີ້

Part I – About Yourself ส่วนที่ 1 – เกี่ยวกับตัวท่าน ສ່ວນທີ່ 1 - ກ່ຽວກັບຕົວທ່ານ

Let us start with a few questions about yourself. เราจะเริ่มโดยถามคำถามเกี่ยวกับข้อมูลส่วนตัวของท่าน ເຮົາຈະເລີ່ມໂດຍຖາມຄຳຖາມກ່ຽວກັບຂໍ້ມູນສ່ວນຕົວຂອງທ່ານ

Guiding question: Who is my informant? คำถามนำ: ผู้ให้สัมภาษณ์คือใคร ຄຳຖາມນຳ: ຜູ້ໃຫ້ສຳພາດແມ່ນໃຜ

1. How old are you? อายุของท่าน ອາຍຸທ່ານ
2. What is your level of education? การศึกษาสูงสุดของท่าน ການສຶກສາສູງສຸດຂອງທ່ານ
3. What is your ethnicity and nationality? เชื้อชาติ และ สัญชาติของท่าน ເຊື້ອຊາດແລະສັນຊາດຂອງທ່ານ

Part II – Practicing the "Think Aloud" Technique ส่วนที่ 2 – เทคนิคการคิดออกเสียง (การบรรยายกระบวนการคิด) ສ່ວນທີ່ 2 - ເທັກນິກການຄິດອອກສຽງ (ການບັນລະຍາຍຂະບວນການຄິດ)

Thank you. As we are trying to improve our survey, you can help us develop the questionnaire by participating in this interview. I would like to ask you to describe your thoughts as you think about the question and the answer as though you are talking to yourself. For example, what do you think as you receive the question? What does the question make you think of? In what way do you want answer to question? How do you arrive at your answer? Let me give you an example so you understand this type of interview better.

Let's say I was asked a question about "What do you see when you go from your home to the nearest temple?" If I was thinking out loud, I would say "[interviewer describing their own village]." So you can see how it works, though that was only one of many ways. The important thing is that there are no right or wrong answers. What really matters is your view and thought process. And this is what I would like you to share with me. So now it is your turn to practice.

ขอบคุณครับ/ค่ะ เนื่องจากเรากำลังอยู่ในช่วงปรับปรุงแบบสอบถาม ท่านสามารถช่วยเราพัฒนาแบบสอบถามนี้ได้โดยการเข้าร่วมการสัมภาษณ์นี้ ซึ่งเราจะขอให้ท่านบรรยายความคิดของท่านในขณะที่ท่านพิจารณาคำถามและคำตอบเสมือนกับท่านกำลังคุยกับตนเอง เช่น ท่านคิดอย่างไรเมื่อท่านได้รับคำถาม คำถามทำให้ท่านนึกถึงอะไรบ้าง ท่านต้องการตอบคำถามเหล่านี้อย่างไร ท่านได้คำตอบมาได้อย่างไร เราจะยกตัวอย่างให้ท่านหนึ่งตัวอย่างเพื่อให้ท่านสามารถเข้าใจการสัมภาษณ์ในลักษณะนี้มากขึ้น

ยกตัวอย่าง ถ้าเราต้องตอบคำถามที่ว่า "ท่านมองเห็นอะไรบ้างหากท่านเดินทางจากบ้านไปวัดที่ใกล้ที่สุด" และเราต้องบรรยายความคิดของเราให้ท่านฟัง เราจะพูดว่า "[ผู้สัมภาษณ์บรรยายการเดินทางในหมู่บ้านของตนเอง]" ซึ่งเป็นเพียงแค่หนึ่งในหลากหลายวิธีที่ท่านสามารถใช้ได้ สิ่งที่สำคัญที่สุดเกี่ยวกับการคิดออกเสียงของท่านคือคำตอบของท่านจะไม่ถูกตัดสินว่าถูกหรือผิด เพราะสิ่งที่เราต้องการเรียนรู้มากที่สุดคือมุมมองและวิธีการคิดของท่าน และนั่นคือสิ่งที่เราอยากให้ท่านแบ่งปันกับเรา ดังนั้นเราจึงขอให้ท่านลองคิดออกเสียงกับเราในคำถามต่อไปนี้

For peer review only - http://bmjopen.bmj.com/site/about/guidelines.xhtml

ຂອບໃຈ ເນື່ອງຈາກເຮົາກຳລັງຢູ່ໃນຊ່ວງປັບປຸງແບບສອບຖາມ ທ່ານສາມາດຊ່ວຍເຮົາພັດທະນາແບບສອບຖາມນີ້ໄດ້ໂດຍການເຂົ້າຮ່ວມການສຳພາດນີ້ ເຊິ່ງເຮົາຈະຂໍໃຫ້ທ່ານບັນລະຍາຍຄວາມຄິດຂອງທ່ານໃນຕອນທີ່ທ່ານກຳລັງພິຈາລະນາຄຳຖາມແລະຄຳຕອບຄືກັບທ່ານກຳລັງລົມກັບຕົນເອງ ເຊັ່ນ ທ່ານຄິດແນວໃດເມື່ອທ່ານໄດ້ຮັບຄຳຖາມ ຄຳຖາມເຮັດໃຫ້ເຮົາຄິດຫາຫຍັງແດ່ ທ່ານຕ້ອງການຕອບຄຳຖາມເຫຼົ່ານີ້ແນວໃດ ທ່ານໄດ້ຄຳຕອບມາໄດ້ແນວໃດ ເຮົາຈະຍົກຕົວຢ່າງໃຫ້ທ່ານໜຶ່ງຕົວຢ່າງເພື່ອໃຫ້ທ່ານສາມາດເຂົ້າໃຈການສຳພາດໃນລັກຊະນະນີ້ຫຼາຍຂຶ້ນ.

ຍົກຕົວຢ່າງ ຖ້າເຮົາຕ້ອງຕອບຄຳຖາມວ່າ ທ່ານເບິ່ງເຫັນຫຍັງແດ່ຖ້າທ່ານເດີນທາງຈາກເຮືອນຫາວັດທີ່ໃກ້ທີ່ສຸດ ແລະເຮົາຕ້ອງບັນລະຍາຍຄວາມຄິດຂອງເຮົາໃຫ້ທ່ານຟັງເຮົາຈະເວົ້າວ່າ "[ຜູ້ສຳພາດບັນຍາຍການເດີນທາງໃນໝູ່ບ້ານຂອງຕົນເອງ]" ເຊິ່ງເປັນພຽງໜຶ່ງໃນຫຼາກຫຼາຍວິທີທີ່ທ່ານສາມາດໃຊ້ໄດ້ ສິ່ງທີ່ສຳຄັນທີ່ສຸດກ່ຽວກັບການຄິດອອກສຽງຂອງທ່ານຄືຄຳຕອບຂອງທ່ານຈະບໍ່ຖືກຕັດສິນວ່າຖືກຫຼືຜິດ ເພາະສິ່ງທີ່ເຮົາຕ້ອງການຮຽນຮູ້ຫຼາຍທີ່ສຸດມຸມມອງແລະວິທີການຄິດຂອງທ່ານ ແລະນັ້ນແມ່ນສິ່ງທີ່ເຮົາຢາກໃຫ້ທ່ານແບ່ງປັນກັບເຮົາ ດັ່ງນັ້ນເຮົາຈຶ່ງຂໍໃຫ້ທ່ານລອງຄິດອອກສຽງກັບເຮົາໃນຄຳຖາມຕໍ່ໄປນີ້

Guiding question: Is the respondent comfortable with the "think aloud" technique? คำถามนำ: ผู้ให้สัมภาษณ์สามารถใช้เทคนิคการคิดออกเสียงหรือไม่ ຄຳຖາມນຳ: ຜູ້ໃຫ້ສຳພາດໃຊ້ເຕັກນິກການຄິດອອກສຽງຫຼືບໍ່

4. I would like you to imagine your village. Could you please describe to me what you see when you go from your home to the nearest temple, in as much detail as you can? เราขอให้ท่านนึกภาพหมู่บ้านของท่านและบรรยายให้เราฟังว่าท่านเห็นอะไรเมื่อท่านเดินทางจากบ้านของท่านไปยังวัดที่ใกล้ที่สุด โดยใช้รายละเอียดมากที่สุดเท่าที่ท่านสามารถทำได้ ເຮົາຂໍໃຫ້ທ່ານນຶກພາບໝູ່ບ້ານຂອງທ່ານແລະບັນຍາຍໃຫ້ເຮົາຟັງວ່າທ່ານເຫັນຫຍັງເມື່ອທ່ານເດີນທາງຈາກເຮືອນຂອງທ່ານໄປຫາວັດທີ່ໃກ້ທີ່ສຸດ ໂດຍໃຊ້ລາຍລະອຽດຫຼາຍທີ່ສຸດທີ່ທ່ານສາມາດເຮັດໄດ້

Before we start, I'd like to give you another example that is more closely related to the actual questions from our questionnaire. Say you ask me to list all persons age ≥18 years in my household with their sex and age, I'd go [*interviewer answering question about adults in household*]. *ก่อนที่เราจะเริ่ม เราขอยกตัวอย่างที่ใกล้เคียงกับคำถามจริงกับแบบสอบถามของเราให้ท่านอีกหนึ่งครั้ง สมมติท่านขอให้เราบอกข้อมูลของสมาชิกอายุ 18 ปีหรือมากกว่า ที่อาศัยอยู่ในครัวเรือนนี้ทุกคน รวมถึงเพศและอายุ เราจะพูดว่า "[ผู้สัมภาษณ์ให้คำตอบเกี่ยวกับสมาชิกในครัวเรือนของตนเอง]." ກ່ອນທີ່ເຮົາຈະເລີ່ມເຮົາຂໍຍົກຕົວຢ່າງທີ່ຄ້າຍກັນກັບແບບສອບຖາມຂອງເຮົາ ສົມມຸດທ່ານບອກໃຫ້ເຮົາບອກຂໍ້ມູນຂອງຊະມະຊິກອາຍຸ 18 ປີຫຼືຫຼາຍກ່ວາ ທີ່ອາໄສຢູ່ໃນຄົວເຮືອນນີ້ທຸກຄົນ ລວມເຖິງເພດແລະອາຍຸ ເຮົາຈະເວົ້າວ່າ "[ຜູ້ສຳພາດໃຫ້ຄຳຕອບກ່ຽວກັບສະມາຊິກໃນຄົວເຮືອນຂອງຕົນເອງ]"*

List all persons aged 18+ years in household กรอกข้อมูลของสมาชิกอายุ 18 ปีหรือมากกว่า ที่อาศัยอยู่ในครัวเรือนนี้ ໃສ່ຂໍ້ມູນຂອງຊະມະຊິກອາຍຸ 18 ປີຫຼືຫຼາຍກ່ວາ ທີ່ອາໄສຢູ່ຄົວເຮືອນນີ້

[*1 respondent per every 5 household members will be selected randomly from this list*] [*ตัวแทน 1 คนต่อสมาชิกจำนวน 5 คน จะถูกสุ่มเลือกให้เข้าร่วมการสัมภาษณ์*] [*ຕົວແທນ 1 ຄົນຕໍ່ຊະມະຊິກຈຳນວນ 5 ຄົນ ຈະຖືກສຸ່ມເລືອກໃຫ້ເຂົ້າຮ່ວມການສຳພາດ*]

**Name** ชื่อ ຊື່	**Sex** เพศ ເພດ<br>**(M / F)** (ช/ญ) (ຊ/ຍ)	**Age** อายุ ອາຍຸ<br>**(in completed Years)** (จำนวนปีเต็ม) (ຈຳນວນປີເຕັມ)	**Available for interview today** ผู้เข้าร่วมสามารถให้สัมภาษณ์ในวันนี้หรือไม่ ຜູ້ເຂົ້າຮ່ວມສາມາດໃຫ້ສຳພາດໃນມື້ນີ້ໄດ້ຫຼືບໍ່?<br>**(Yes / No)** (ใช่/ไม่ใช่) (ແມ່ນ/ບໍ່ແມ່ນ)

Part III – Social-Network-Related Survey Questions ส่วนที่ 3 – คำถามเกี่ยวกับเครือข่ายสังคม ສ່ວນທີ່ 3 - ຄຳຖາມກ່ຽວກັບເຄືອຂ່າຍສັງຄົມ

I will now ask you some questions about your interactions with other people within and outside of your village. เราจะเริ่มโดยการถามคำถามท่านเกี่ยวกับปฏิสัมพันธ์ของท่านกับชาวบ้านคนอื่นๆ ทั้งในและนอกหมู่บ้าน ເຮົາຈະເລີ່ມໂດຍການຖາມຄຳຖາມທ່ານກ່ຽວກັບການພົວພັນຂອງທ່ານກັບຊາວບ້ານຄົນອື່ນໆ ທັງໃນແລະພາຍນອກຂອງໝູ່ບ້ານ

5. What are you thinking about when you hear the following question? ท่านมีความคิดอะไรบ้างเมื่อท่านได้ยินคำถามต่อไปนี้ ທ່ານມີຄວາມຄິດຫຍັງແດ່ເມື່ອທ່ານໄດ້ຍິນຄຳຖາມຕໍ່ໄປນີ້

For peer review only - http://bmjopen.bmj.com/site/about/guidelines.xhtml

Where do you spend most of your time interacting with other people from your village? ท่านใช้เวลาส่วนมากในการพบปะพูดคุยกับชาวบ้านในหมู่บ้านของท่านในสถานที่ใด ທ່ານໃຊ້ເວລາຫຼາຍທີ່ສຸດໃນການພົບປະໂອ້ລົມກັບຊາວບ້ານໃນໝູ່ບ້ານຂອງທ່ານໃນບ່ອນໃດແດ່<br><br>[*mark all that apply or select 99 if respondent indicates no noteworthy interaction*] [*ตอบได้มากกว่า 1 ตัวเลือก หรือเลือก 99 หากไม่มีปฏิสัมพันธ์ที่เด่นชัดปรากฏ*] [*ຕອບໄດ້ຫຼາຍກວ່າ 1 ຕົວເລືອກ ຫຼືເລືອກ 99 ຖ້າບໍ່ມີການພົວພັນທີ່ຊັດເຈນ*]	Field (specify) ไร่นา (โปรดระบุ) ໄຮ່ນາ (ກະລຸນາບອກ) ___ .... 1<br>Temple (specify) วัด (โปรดระบุ) ວັດ (ກະລຸນາບອກ) ___ .... 2<br>Local store (specify) ร้านค้า (โปรดระบุ) ຮ້ານຄ້າ (ກະລຸນາບອກ) ___ .... 3<br>Market (specify) ตลาด (โปรดระบุ) ຕະຫຼາດ (ກະລຸນາບອກ) ___........ 4<br>Children's schools (specify) โรงเรียนของบุตรหลาน (โปรดระบุ) ໂຮງຮຽນຂອງລູກ (ກະລຸນາບອກ) __ .... 5<br>Home (specify) ที่บ้าน (โปรดระบุ) ເຮືອນ (ກະລຸນາບອກ) ____ .... 6<br>Workplace (specify) ที่ทำงาน (โปรดระบุ) ບ່ອນເຮັດວຽກ (ກະລຸນາບອກ) ___........ 7<br>Other site (specify) อื่นๆ (โปรดระบุ) ອື່ນໆ (ກະລຸນາບອກ) ___........ 8<br><br>I don't interact notably with others ไม่มีปฏิสัมพันธ์ที่เด่นชัดกับชาวบ้านคนอื่นๆ ບໍ່ມີການພົວພັນທີ່ຊັດເຈນກັບຊາວບ້ານຄົນອື່ນໆ........ 99

a. How do you understand "interacting?" สำหรับท่าน ท่านคิดว่าคำว่า "พบปะพูดคุย" มีความหมายว่าอย่างไร ສຳລັບທ່ານ ທ່ານຄິດວ່າ "ການພົບປະໂອ້ລົມ" ມີຄວາໝາຍວ່າແນວໃດ

b. What does "most of your time" mean to you? Which time frame did you have in mind? คำว่า "เวลาส่วนมาก" สำหรับท่านมีความหมายว่าอย่างไร ในขณะที่ท่านตอบคำถามในใจของท่านนึกถึงช่วงเวลาใด ຄຳວ່າ "ເວລາຫຼາຍທີ່ສຸດ" ສຳລັບທ່ານມີຄວາມໝາຍວ່າແບບໃດ ໃນຂະນະທີ່ທ່ານຕອບຄຳຖາມໃນໃຈຂອງທ່ານນຶກເຖິງຊ່ວງເວລາໃດ

c. What are the possible places where people spend time together? มีที่ใดบ้างที่ชาวบ้านสามารถใช้เวลาร่วมกันได้ ມີບ່ອນໃດແດ່ທີ່ຊາວບ້ານສາມາດໃຊ້ເວລາຮ່ວມກັນໄດ້

d. Please ask us the same question in your own words. กรุณาถามคำถามนี้กับเราโดยใช้คำพูดในแบบของท่านเอง ກະລຸນາຖາມຄຳຖາມນີ້ກັບເຮົາໂດຍໃຊ້ຄຳເວົ້າໃນແບບຂອງທ່ານເອງ

e. What kind of things do you normally talk about when you interact with other villagers? โดยปกติเมื่อท่านพบปะกับชาวบ้านคนอื่นๆ ท่านสนทนากันเรื่องอะไรบ้าง ໂດຍປົກກະຕິທ່ານພົບປະກັບຊາວບ້ານຄົນອື່ນໆ ທ່ານລົມເລື່ອງຫຍັງແດ່

For peer review only

6. I will now ask you a set of questions, regarding the people you interact with. ชุดคำถามต่อไปจะถามถึงบุคคลที่ท่านพบปะพูดคุยด้วย ຊຸດຄຳຖາມຕໍ່ໄປຈະຖາມເຖິງບຸກຄົນທີ່ທ່ານພົບປະໂອ້ລົມນຳ

With whom do you interact on a regular basis? โดยปกติในชีวิตประจำวันท่านพบปะพูดคุยกับใครบ้าง ໂດຍປົກກະຕິໃນຊີວິດປະຈຳວັນທ່ານພົບປະໂອ້ລົມກັບໃຜແດ່

	a) What is the name of the person? ชื่อ ຊື່	b) How is this person related to you? ความสัมพันธ์กับท่าน ຄວາມສຳພັນກັບທ່ານ [*give examples if respondent is unsure about answer categories*] [*หากผู้ให้สัมภาษณ์ไม่มั่นใจในหมวดคำตอบ ให้ยกตัวอย่างคำตอบได้*] [*ຖ້າຜູ້ໃຫ້ສຳພາດບໍ່ໝັ້ນໃຈໃນໝວດຄຳຕອບ ໃຫ້ຍົກຕົວຢ່າງຄຳຕອບໄດ້*]	c) What is the sex of this person? เพศ ເພດ	d) Where does this person live? ที่อยู่ ທີ່ຢູ່	e) What is the name of the household head of this person? ใครคือหัวหน้าครัวเรือนของบุคคลนี้ ໃຜແມ່ນຫົວໜ້າຄົວເຮືອນຂອງບຸກຄົນນີ້	f) How often do you interact with this person? ท่านพบปะพูดคุยกับบุคคลนี้บ่อยเท่าใด ທ່ານພົບປະໂອ້ລົມກັບບຸກຄົນນີ້ບ່ອຍປານໃດ	g) How do you interact with this person? ท่านพบปะพูดคุยกับบุคคลนี้อย่างไร ທ່ານພົບປະໂອ້ລົມກັບບຸກຄົນນີ້ແນວໃດ [*Mark all that apply*] [*ตอบได้มากกว่า 1 ตัวเลือก*] [*ຕອບໄດ້ຫຼາຍກ່ວາ 1 ຕົວເລືອກ*]	h) How often do your conversations relate to health and well-being? ท่านมีการสนทนากับบุคคลนี้เกี่ยวกับเรื่องสุขภาพบ่อยเท่าใด ທ່ານໂອ້ລົມກັບບຸກຄົນນີ້ເລື່ອງສຸຂະພາບບ່ອຍປານໃດ [*If respondent does not discuss health, then move on to next contact after indicating "never"*] [*หากผู้ให้ไม่สบายใจกับการพูดคุยเรื่องสุขภาพ ให้ผู้สัมภาษณ์ใส่รหัส 0 และข้ามไปที่บุคคลต่อไป*] [*ຖ້າຜູ້ໃຫ້ສຳພາດບໍ່ສະບາຍໃຈທີ່ຈະລົມເລື່ອງສຸຂະພາບໃຫ້ຜູ້ສຳພາດໃສ່ລະຫັດ 0 ແລະຂ້າມໄປຫາບຸກຄົນຕໍ່ໄປ*]	i) What health-related subjects do your conversations cover? หัวข้อการสนทนาเกี่ยวกับสุขภาพของท่านกับบุคคลนี้คือเรื่องอะไร ຫົວຂໍ້ສົນທະນາກ່ຽວກັບສຸຂະພາບຂອງທ່ານກັບບຸກຄົນນີ້ແມ່ນເລື່ອງຫຍັງ [*Mark all that apply*] [*ตอบได้มากกว่า 1 ตัวเลือก*] [*ຕອບໄດ້ຫຼາຍກ່ວາ 1 ຕົວເລືອກ*]
Contact 1 คนที่ 1 ຄົນທີ່ 1	Name ชื่อ ຊື່ ____	Spouse คู่สมรส ຄູ່ແຕ່ງດອງ........ 1<br>Parent บิดามารดา ພໍ່ແມ່............. 2<br>Child บุตร ລູກ...................... 3<br>Sibling พี่น้อง ອ້າຍນ້ອງ .... 4<br>Other relative ญาติ ພີ່ນ້ອງ ...... 5<br>Neighbour เพื่อนบ้าน ເພື່ອນບ້ານ . 6<br>Friend (if not neighbour) เพื่อน (ที่ไม่ใช่เพื่อนบ้าน) ໝູ່ (ທີ່ບໍ່ແມ່ນເພື່ອນບ້ານ) .... 7<br>Other villager ชาวบ้านคนอื่นๆ ຊາວບ້ານຄົນອື່ນໆ.................... 8<br>Other (specify) อื่นๆ (โปรดระบุ) ອື່ນໆ (ກະລຸນາບອກ) _ 9	Female..1 หญิง ຍິງ<br>Male.....2 ชาย ຊາຍ	In village ....1 (specify: _____) ในหมู่บ้าน (โปรดระบุ: __) ໃນໝູ່ບ້ານ(ກະລຸນາບອກ: __)<br>Outside village ...2 (specify: _____) นอกหมู่บ้าน (โปรดระบุ: __) ນອກໝູ່ບ້ານ(ກະລຸນາບອກ: __)	Name of household head ชื่อหัวหน้าครัวเรือน: ຊື່ຫົວໜ້າຄົວເຮືອນ ______	Daily or more often ทุกวันหรือบ่อยกว่า ທຸກມື້ຫຼືໜ້ອຍກ່ວາ.........4<br>Weekly or few times/week ทุกสัปดาห์หรือมากกว่าหนึ่งครั้งต่อสัปดาห์ ທຸກອາທິດຫຼືຫຼາຍກ່ວາໜຶ່ງເທື່ອຕໍ່ອາທິດ..............................3<br>Monthly or few times/month ทุกเดือนหรือมากกว่าหนึ่งครั้งต่อเดือน ທຸກເດືອນຫຼືຫຼາຍກ່ວາໜຶ່ງເທື່ອຕໍ່າເດືອນ..............................2<br>Yearly or few times/year ทุกปีหรือมากกว่าหนึ่งครั้งต่อปี ທຸກປີຫຼືຫຼາຍກວ່າໜຶ່ງເທື່ອຕໍ່ປີ 1	Face-to-face ตัวต่อตัว ໂຕຕໍ່ໂຕ..........1<br>Voice call โทรศัพท์ ໂທລະສັບ ....2<br>Messenger ติดต่อสื่อสารโดยใช้อินเตอร์เน็ต ສື່ສານໂດຍໃຊ້ອິນເຕີເນັດ.......3<br>Other (specify) อื่นๆ (โปรดระบุ) ອື່ນໆ (ກະລຸນາບອກ) ______ 4	Always (100%) เป็นประจำ (100%) ເປັນປະຈຳ (100%) .. 5<br>Nearly always (<100%) เกือบเป็นประจำ (<100%) ເກືອບເປັນປະຈຳ (<100%) .... 4<br>Often (<90%) บ่อยครั้ง (<90%) ບ່ອຍ (<90%) .... 3<br>Sometimes (<50%) บางครั้ง (<50%) ບາງຄັ້ງ (<50%)........ 2<br>Hardly ever (<10%) แทบไม่เคย (<10%) ເກືອບບໍ່ເຄີຍ (<10%). 1<br>Never (0%) ไม่เคยเลย (0%).... ບໍ່ເຄີຍເລີຍ.......................... 0<br>➔ [*go to next contact*] [*ข้ามไปบุคคลต่อไป*] [*ຂ້າມໄປບຸກຄົນຕໍ່ໄປ*]	Talk about general well-being ไถ่ถามสารทุกข์สุกดิบทั่วไป ຖາມກ່ຽວກັບການເປັນຢູ່ທົ່ວໄປ ....1<br>Update on health knowledge/news อัพเดทเรื่องความรู้หรือข่าวสารสุขภาพใหม่ๆ ອັບເດດເລື່ອງຄວາມຮູ້ຫຼືຂ່າວສານສຸຂະພາບໃໝ່ໆ................2<br>Update on personal health status .... อัพเดทเรื่องสุขภาพส่วนตัว ອັບເດດເລື່ອງສຸຂະພາບສ່ວນຕົວ ....3<br>Advice on diagnosis ให้คำแนะนำเกี่ยวกับการวินิจฉัย ໃຫ້ຄຳແນະນຳກ່ຽວກັບການວິເຄາະພະຍາດ.......................4<br>Advice on doctors ให้คำแนะนำเกี่ยวกับแพทย์ ໃຫ້ຄຳແນະນຳກ່ຽວກັບໝໍ...5<br>Advice on treatment/medicine ให้คำแนะนำเกี่ยวกับวิธีการรักษา/ยา ໃຫ້ແນະນຳກ່ຽວກັບວິທີຮັກສາ/ຢາ 6

						Less often or never ไม่ค่อยบ่อยหรือไม่เคยเลย ບໍ່ເຄີຍເລີຍ 0			Emotional support/reassure ให้กำลังใจ ให้ความมั่นใจ ໃຫ້ກຳລັງໃຈ ໃຫ້ຄວາມໝັ້ນໃຈ ....7<br>Provide help during illness ให้ความช่วยเหลือระหว่างการป่วย ໃຫ້ຄວາມຊ່ວຍເຫຼືອຕອນບໍ່ສະບາຍ ....8<br>Other (specify) อื่นๆ (โปรดระบุ) ອື່ນໆ (ກະລຸນາບອກ)___ .9
Contact 2 คนที่ 2 ຄົນທີ່ 2	Name ชื่อ ຊື່	1 2 3 4 5 6 7 8 9	1 2	1 2	Name ชื่อ ຊື່	0 1 2 3 4	1 2 3 4	0 1 2 3 4 5	1 2 3 4 5 6 7 8 9
[*continue until Contact x – maximum 10 contacts*] [*บันทึกบุคคลที่มีความเกี่ยวข้องระหว่างการเจ็บป่วยทุกคน ไม่เกิน 10 คน*] [*ບັນທຶກບຸກຄົນທີ່ມີຄວາມກ່ຽວຂ້ອງລະຫ່ວາງອາການເຈັບປ່ວຍທຸກຄົນ ບໍ່ຫຼາຍກ່ວາ 10 ຄົນ*]	Name ชื่อ ຊື່	1 2 3 4 5 6 7 8 9	1 2	1 2	Name ชื่อ ຊື່	0 1 2 3 4	1 2 3 4	0 1 2 3 4 5	1 2 3 4 5 6 7 8 9

For peer review only - http://bmjopen.bmj.com/site/about/guidelines.xhtml

a. How did you go about answering these questions? ท่านมีวิธีการตอบคำถามเหล่านี้อย่างไร ທ່ານມີວິທີການຕອບຄໍາຖາມເຫຼົ່ານີ້ແນວໃດ
b. What did you imagine while answering the question, e.g. the “interaction setting” that you had in mind? ในขณะที่ท่านตอบคำถามเหล่านี้ท่านมีภาพอะไรอยู่ในใจ เช่น ลักษณะของสถานที่ ฯลฯ ໃນຂະນະທີ່ທ່ານຕອບຄໍາຖາມເຫຼົ່ານີ້ທ່ານມີພາບຫຍັງຢູ່ໃນໃຈ ເຊັ່ນ ລັກຊະນະຂອງສະຖານທີ່ ແລະອື່ນໆ
c. What time period were you thinking of? จากคำตอบที่ท่านให้เราเกี่ยวกับบุคคลที่ท่านมีปฏิสัมพันธ์ด้วย ท่านนึกถึงช่วงเวลาใด ຈາກຄໍາຕອບທີ່ທ່ານໃຫ້ເຮົາກ່ຽວກັບບຸກຄົນທີ່ທ່ານມີປະຕິສໍາພັນນໍາ ທ່ານນຶກເຖິງຊ່ວງເວລາໃດ
d. How did you come up with the order of people? Why did you think of these people first rather than others? ท่านมีการเรียงลำดับบุคคลเหล่านี้อย่างไร เพราะเหตุใดท่านจึงบอกชื่อบุคคลเหล่านี้กับเราเป็นลำดับแรกๆ หากเปรียบเทียบกับบุคคลอื่น ທ່ານມີການລຽງລໍາດັບບຸກຄົນເຫຼົ່ານີ້ແບບໃດ ຍ້ອນຫຍັງທ່ານຈຶ່ງບອກຊື່ບຸກຄົນເຫຼົ່ານີ້ກັບກ່ອນຄົນອື່ນ
e. What is the best way we can find these people from your interaction circle? How would you describe where they live? หากเราต้องการพูดคุยกับบุคคลที่ท่านกล่าวถึงเหล่านี้ เราสามารถพบพวกเขาได้อย่างไร ท่านจะสามารถอธิบายที่อยู่ของพวกเขาได้อย่างไร ຖ້າເຮົາຕ້ອງການລົມກັບບຸກຄົນທີ່ທ່ານກ່າວເຖິງເຫຼົ່ານີ້ ເຮົາສາມາດພົບພົບພວກເຂົາໄດ້ແນວໃດ ທ່ານສາມາດອະທິບາຍທີ່ຢູ່ຂອງພວກເຂົາໄດ້ແນວໃດ

For peer review

Part IV – Health-Related Survey Questions ส่วนที่ 4 – คำถามเกี่ยวกับสุขภาพ ສ່ວນທີ່ 4 - ຄໍາຖາມກ່ຽວກັບສຸຂະພາບ

Thank you for this. Now we come to a part where I will ask you some questions about health and health providers around here. ขอบคุณครับ/ค่ะ คำถามช่วงต่อไปจะเป็นเรื่องสุขภาพและสถานบริการสุขภาพในพื้นที่นี้ ຂອບໃຈ ຄໍາຖາມຕໍ່ໄປນີ້ຈະເປັນຄໍາຖາມກ່ຽວກັບສຸຂະພາບ ແລະສະຖານບໍລິການສຸຂະພາບໃນພື້ນທີ່ນີ້

7. For the following question, can you please “think out loud” about your answers? เราขอให้ท่าน “คิดออกเสียง” ในระหว่างที่ท่านตอบคำถามต่อไปนี้ ເຮົາຂໍໃຫ້ທ່ານ “ຄິດອອກສຽງ” ໃນລະຫວ່າງການຕອບຄໍາຖາມຕໍ່ໄປນີ້

I would now like to ask you about the sources of health advice and medicine or other treatment that are available to you. Please think about all the places where you can go to get advice, treatment, or drugs if you (or your children) are sick. คำถามต่อไปเป็นคำถามเกี่ยวกับสถานที่หรือบุคคลที่ท่านสามารถรับคำปรึกษา รับยา หรือ รับการรักษาอื่นๆ ได้ กรุณานึกถึงสถานที่ทุกแห่งหรือคนทุกคนที่ท่านสามารถรับคำปรึกษา รับการรักษา หรือรับยา หากท่านหรือเด็กในปกครองของท่านป่วย ຄໍາຖາມຕໍ່ໄປເປັນຄໍາຖາມກ່ຽວກັບສະຖານທີ່ຫຼືບຸກຄົນທີ່ທ່ານສາມາດຮັບຄໍາປຶກສາ ຮັບຢາ ຫຼືຮັບການຮັກສາອື່ນໆ ໄດ້ ກະລຸນານຶກເຖິງສະຖານທີ່ທຸກແຫ່ງຫຼືບຸກຄົນທຸກຄົນທີ່ທ່ານສາມາດຮັບຄໍາປຶກສາ ຮັບການຮັກສາ ຫຼືຮັບຢາ ຖ້າຫາກທ່ານຫຼືເດັກທີ່ທ່ານດູແລບໍ່ສະບາຍ<br><br>Which of the following options do you consider for medical treatment, advice, or to get medicine? ท่านเคยได้รับคำปรึกษา รับยา หรือ รับ	Drug dispensary, other local store selling medicine ร้านขายของชำหรือร้านค้าทั่วไปที่มีการจำหน่ายยา ຮ້ານທົ່ວໄປທີ່ມີການຂາຍຢາ	Medical treatment เพื่อการรักษา ສໍາລັບການປິ່ນປົວ .... 1<br>Medical advice เพื่อคำแนะนำในการรักษา ສໍາລັບຄໍາແນະນໍາໃນການປິ່ນປົວ .... 2<br>Access to medicine เพื่อซื้อ/รับยา ສໍາລັບຊື້/ຮັບຢາ .... 3<br>Don’t consider this provider ไม่มีการใช้บริการประเภทนี้ ບໍ່ມີການໃຊ້ບໍລິການປະເພດນີ້..0<br>Don’t know such a provider ไม่รู้จักผู้ให้บริการประเภทนี้ ບໍ່ຮູ້ຈັກຜູ້ໃຫ້ບໍລິການປະເພດນີ້.99
	Traditional healer หมอชาวบ้าน ໝໍຊາວບ້ານ	Medical treatment เพื่อการรักษา ສໍາລັບການປິ່ນປົວ .... 1<br>Medical advice เพื่อคำแนะนำในการรักษา ສໍາລັບຄໍາແນະນໍາໃນການປິ່ນປົວ .... 2<br>Access to medicine เพื่อซื้อ/รับยา ສໍາລັບຊື້/ຮັບຢາ .... 3<br>Don’t consider this provider ไม่มีการใช้บริการประเภทนี้ ບໍ່ມີການໃຊ້ບໍລິການປະເພດນີ້..0<br>Don’t know such a provider ไม่รู้จักผู้ให้บริการประเภทนี้ ບໍ່ຮູ້ຈັກຜູ້ໃຫ້ບໍລິການປະເພດນີ້.99
	Pharmacist เภสัชกร ຜູ້ຂາຍຢາ	Medical treatment เพื่อการรักษา ສໍາລັບການປິ່ນປົວ .... 1<br>Medical advice เพื่อคำแนะนำในการรักษา ສໍາລັບຄໍາແນະນໍາໃນການປິ່ນປົວ .... 2<br>Access to medicine เพื่อซื้อ/รับยา ສໍາລັບຊື້/ຮັບຢາ .... 3<br>Don’t consider this provider ไม่มีการใช้บริการประเภทนี้ ບໍ່ມີການໃຊ້ບໍລິການປະເພດນີ້..0<br>Don’t know such a provider ไม่รู้จักผู้ให้บริการประเภทนี้ ບໍ່ຮູ້ຈັກຜູ້ໃຫ້ບໍລິການປະເພດນີ້.99
	Private clinic คลินิกเอกชน ຄລີນິກເອກະຊົນ	Medical treatment เพื่อการรักษา ສໍາລັບການປິ່ນປົວ .... 1<br>Medical advice เพื่อคำแนะนำในการรักษา ສໍາລັບຄໍາແນະນໍາໃນການປິ່ນປົວ .... 2<br>Access to medicine เพื่อซื้อ/รับยา ສໍາລັບຊື້/ຮັບຢາ .... 3<br>Don’t consider this provider ไม่มีการใช้บริการประเภทนี้ ບໍ່ມີການໃຊ້ບໍລິການປະເພດນີ້..0<br>Don’t know such a provider ไม่รู้จักผู้ให้บริการประเภทนี้ ບໍ່ຮູ້ຈັກຜູ້ໃຫ້ບໍລິການປະເພດນີ້.99

For peer review only - http://bmjopen.bmj.com/site/about/guidelines.xhtml

การรักษา จากใครบ้าง ທ່ານເຄີຍໄດ້ຮັບຄຳປຶກສາ ຮັບຢາ ຫຼືຮັບການຮັກສາອື່ນຈາກໃຜແດ່<br><br>[*Mark all that apply*] [*ตอบได้มากกว่า 1 ตัวเลือก*] [*ຕອບໄດ້ຫຼາຍກ່ວາ 1 ຕົວເລືອກ*]	Private hospital โรงพยาบาลเอกชน ໂຮງໝໍເອກະຊົນ	Medical treatment เพื่อการรักษา ສຳລັບການປິ່ນປົວ .... 1<br>Medical advice เพื่อคำแนะนำในการรักษา ສຳລັບຄຳແນະນຳໃນການປິ່ນປົວ .... 2<br>Access to medicine เพื่อซื้อ/รับยา ສຳລັບຊື້/ຮັບຢາ .... 3<br>Don't consider this provider ไม่มีการใช้บริการประเภทนี้ ບໍ່ມີການໃຊ້ບໍລິການປະເພດນີ້..0<br>Don't know such a provider ไม่รู้จักผู้ให้บริการประเภทนี้ ບໍ່ຮູ້ຈັກຜູ້ໃຫ້ບໍລິການປະເພດນີ້.99
	Primary care unit สถานีอนามัยของรัฐ ສະຖານີອານາໄມຂອງລັດ	Medical treatment เพื่อการรักษา ສຳລັບການປິ່ນປົວ .... 1<br>Medical advice เพื่อคำแนะนำในการรักษา ສຳລັບຄຳແນະນຳໃນການປິ່ນປົວ .... 2<br>Access to medicine เพื่อซื้อ/รับยา ສຳລັບຊື້/ຮັບຢາ .... 3<br>Don't consider this provider ไม่มีการใช้บริการประเภทนี้ ບໍ່ມີການໃຊ້ບໍລິການປະເພດນີ້..0<br>Don't know such a provider ไม่รู้จักผู้ให้บริการประเภทนี้ ບໍ່ຮູ້ຈັກຜູ້ໃຫ້ບໍລິການປະເພດນີ້.99
	Public hospital โรงพยาบาลรัฐ ໂຮງໝໍລັດ	Medical treatment เพื่อการรักษา ສຳລັບການປິ່ນປົວ .... 1<br>Medical advice เพื่อคำแนะนำในการรักษา ສຳລັບຄຳແນະນຳໃນການປິ່ນປົວ .... 2<br>Access to medicine เพื่อซื้อ/รับยา ສຳລັບຊື້/ຮັບຢາ .... 3<br>Don't consider this provider ไม่มีการใช้บริการประเภทนี้ ບໍ່ມີການໃຊ້ບໍລິການປະເພດນີ້..0<br>Don't know such a provider ไม่รู้จักผู้ให้บริการประเภทนี้ ບໍ່ຮູ້ຈັກຜູ້ໃຫ້ບໍລິການປະເພດນີ້.99
	Other providers or Internet? Specify: ผู้ให้บริการรูปแบบอื่นๆ หรืออินเตอร์เน็ต (โปรดระบุ) ຜູ້ບໍລິການໃນຮູບແບບອື່ນໆຫຼືອິນເຕີເນັດ (ກະລຸນາບອກ)<br>__________	Medical treatment เพื่อการรักษา ສຳລັບການປິ່ນປົວ .... 1<br>Medical advice เพื่อคำแนะนำในการรักษา ສຳລັບຄຳແນະນຳໃນການປິ່ນປົວ .... 2<br>Access to medicine เพื่อซื้อ/รับยา ສຳລັບຊື້/ຮັບຢາ .... 3<br>Don't consider this provider ไม่มีการใช้บริการประเภทนี้ ບໍ່ມີການໃຊ້ບໍລິການປະເພດນີ້..0<br>Don't know such a provider ไม่รู้จักผู้ให้บริการประเภทนี้ ບໍ່ຮູ້ຈັກຜູ້ໃຫ້ບໍລິການປະເພດນີ້.99

a. Do you choose any of these providers for specific symptoms or illnesses? ท่านมีการเลือกสถานบริการสุขภาพแห่งใดแห่งหนึ่งสำหรับรักษาโรคใดโดยเฉพาะหรือไม่ ທ່ານມີການເລືອກສະຖານສຸຂະພາບທີ່ໃດທີ່ໜຶ່ງສຳລັບກວດພະຍາດໃດໂດຍສະເພາະຫຼືບໍ່

b. Do you think these questions are too hard? ท่านรู้สึกว่าคำถามเหล่านี้ยากเกินไปหรือไม่ ທ່ານຮູ້ສຶກວ່າຄຳຖາມເຫຼົ່ານີ້ຍາກເກີນໄປຫຼືບໍ່

c. How big of an area do you think of when you answered these questions? E.g. within the village, the province, the surrounding provinces ในขณะที่ท่านตอบคำถามเหล่านี้ ท่านนึกถึงสถานบริการสุขภาพภายในบริเวณใด เช่น ภายในหมู่บ้าน ภายในจังหวัด หรือรวมจังหวัดรอบนอกด้วย ຕອນທີ່ທ່ານຕອບຄຳຖາມເຫຼົ່ານີ້ ທ່ານນຶກເຖິງສະຖານທີ່ບໍລິການສຸຂະພາບພາຍໃນບໍລິເວນໃດ ເຊັ່ນ ພາຍໃນໝູ່ບ້ານ ພາຍໃນແຂວງ ຫຼືແຂວງຮອບນອກ

d. Are there other forms of health provider that you could use but we have not talked about? มีสถานบริการสุขภาพลักษณะอื่นที่ท่านสามารถใช้บริการได้แต่เรายังไม่ได้พูดถึงอีกหรือไม่ ມີສະຖານທີ່ບໍລິການສຸຂະພາບລັກຊະນະອື່ນທີ່ທ່ານສາມາດໃຊ້ບໍລິການໄດ້ແຕ່ເຮົາຍັງບໍ່ໄດ້ເວົ້າເຖິງອີກຫຼືບໍ່

8. I will now ask you another series of questions regarding a recent illness. ต่อไปเราจะถามคำถามเกี่ยวกับอาการป่วยครั้งล่าสุดของท่าน ຕໍ່ໄປນີ້ເຮົາຈະຖາມຄຳຖາມກ່ຽວກັບການເຈັບເປັນຄັ້ງລ່າສຸດຂອງທ່ານ

Did you have an acute illness (not a chronic, long-term condition that comes again and again) or an accident in the last two months? ในช่วงสองเดือนที่ผ่านมา ท่านมีอาการป่วย (ไม่รวมโรคเรื้อรังหรือโรคที่เป็นๆ หายๆ) หรือประสบอุบัติเหตุ หรือไม่ ໃນຊ່ວງສອງເດືອນທີ່ຜ່ານມາ ທ່ານມີອາການເຈັບເປັນ (ບໍ່ລວມພະຍາດຊຳເຮື້ອຫຼືພະຍາດທີ່ເປັນຕໍ່ເນື່ອງ) ຫຼືອຸປະຕິເຫດຫຼືບໍ່?	No ไม่ใช่ ບໍ່ແມ່ນ .... 2<br>Yes ใช่ ແມ່ນ............ 1
[*if yes*:] [*ถ้าใช่:*] [*ຖ້າແມ່ນ:*]	Description of condition: คำอธิบายอาการ ຄຳອະທິບາຍອາການ<br>______________________________

For peer review only - http://bmjopen.bmj.com/site/about/guidelines.xhtml

Can you please describe the symptoms or problem in your own words? กรุณาอธิบายอาการหรือปัญหาในแบบที่ท่านเข้าใจ ກະລຸນາອະທິບາຍອາການຫຼືບັນຫາໃນແບບທີ່ທ່ານເຂົ້າໃຈ	________________________________

For peer review only

<table>
<tr><td>Can you please describe the diagnosis of the illness if you received any and where you received it?

[note: the diagnosis might be given by any medical provider including untrained and informal. Record all diagnoses if more than one.] หากท่านได้รับการวินิจฉัยอาการ กรุณาอธิบายการวินิจฉัยนั้นและระบุแหล่งที่มา [หมายเหตุ: ผู้ให้สัมภาษณ์อาจได้รับการวินิจฉัยจากผู้ให้บริการทางสุขภาพลักษณะใดก็ได้ ซึ่งรวมถึงผู้ให้บริการที่ไม่รับการศึกษาด้านการแพทย์และผู้ให้บริการนอกระบบ กรอกข้อมูลการวินิจฉัยทั้งหมด หากผู้ให้สัมภาษณ์ได้รับการวินิจฉัยมากกว่า 1 ครั้ง] ຖ້າຫາກທ່ານໄດ້ຮັບການວິເຄາະພະຍາດ ກະລຸນາອະທິບາຍການວິເຄາະພະຍາດເຫື່ອນັ້ນແລະລະບຸແຫຼ່ງຂໍ້ມູນ [ຫມາຍເຫດ: ຜູ້ໃຫ້ສຳພາດອາດໄດ້ຮັບການວິເຄາະພະຍາດຈາກຜູ້ໃຫ້ບໍລິການສຸຂະພາບປະເພດໃດກໍ່ໄດ້ ເຊິ່ງຮວມເຖິງຜູ້ໃຫ້ບໍລິການສຸຂະພາບທີ່ບໍ່ມີການສຶກສາທາງການແພດແລະຜູ້ໃຫ້ບໍລິການນອກລະບົບ ຕື່ມໃສ່ຂໍ້ມູນການວິເຄາະພະຍາດທັງຫມົດ ຖ້າຜູ້ໃຫ້ສຳພາດໄດ້ຮັບການວິເຄາະພະຍາດຫຼາຍກ່ວາ 1 ຄັ້ງ]

[Response codes รหัสคำตอบ ລະຫັດຄຳຕອບ]
Drug dispensary, other local store selling medicine
ร้านขายของชำหรือร้านค้าทั่วไปที่มีการจำหน่ายยา ຮ້ານທົ່ວໄປທີ່ມີການຂາຍຢາ 1
Traditional healer หมอชาวบ้าน ໝໍຊາວບ້ານ 2
Pharmacist เภสัชกร ຜູ້ຂາຍຢາ 3
Private clinic คลินิกเอกชน ຄລີນິກເອກະຊົນ 4
Private hospital โรงพยาบาลเอกชน ໂຮງໝໍເອກະຊົນ 5
Primary care unit สถานีอนามัยของรัฐ ສະຖານີອານາໄມຂອງລັດ....................6
Public hospital โรงพยาบาลรัฐ ໂຮງໝໍລັດ 7
Other providers or Internet? Specify: ผู้ให้บริการรูปแบบอื่นๆ หรืออินเตอร์เน็ต (โปรดระบุ) ຜູ້ບໍລິການໃນຮູບແບບອື່ນໆຫຼືອິນເຕີເນັດ (ກະລຸນາບອກ) _________ ....8</td>
<td>Diagnosis 1 การวินิจฉัยโรค 1 ການວິເຄາະພະຍາດ 1:
____

Medical provider 1 ผู้ให้บริการทางสุขภาพ 1 ຜູ້ໃຫ້ບໍລິການສຸຂະພາບ 1:
1 2 3 4 5 6 7 8

Diagnosis 2 การวินิจฉัยโรค 2 ການວິເຄາະພະຍາດ 2:
____

Medical provider 2 ผู้ให้บริการทางสุขภาพ 2 ຜູ້ໃຫ້ບໍລິການສຸຂະພາບ 2:
1 2 3 4 5 6 7 8

Diagnosis 3 การวินิจฉัยโรค 3 ການວິເຄາະພະຍາດ 3:
____

Medical provider 3 ผู้ให้บริการทางสุขภาพ 3 ຜູ້ໃຫ້ບໍລິການສຸຂະພາບ 3:
1 2 3 4 5 6 7 8</td></tr>
<tr><td>When did you experience the accident/discomfort (for the first time) ท่านเริ่มมีอาการป่วยหรือประสบอุบัติเหตุตั้งแต่เมื่อใด ທ່ານເລີ່ມມີອາການເຈັບເປັນຫຼືຖືກອຸບັດຕິເຫດຕັ້ງແຕ່ເມື່ອໃດ?</td>
<td>Onset: ___ days / ______ weeks / _____ months ago
เริ่มอาการป่วย/อุบัติเหตุ: _______ วัน / _______ สัปดาห์ / ______ เดือนที่แล้ว
ເລີ່ມມີອາການບໍ່ສະບາຍຫຼືອຸບັດຕິເຫດ: ___ ມື້ / ___ ອາທິດ / ___ ເດືອນກ່ອນ</td></tr>
<tr><td>Would you describe the illness/accident as “mild,” “moderate,” or “severe”? ท่านคิดว่าการป่วยหรืออุบัติเหตุครั้งนั้นอยู่ในขั้นน้อย ปานกลาง หรือหนัก ທ່ານຄິດວ່າອາການເຈັບເປັນຫຼືອຸບັດຕະເຫດຄັ້ງນັ້ນຢູ່ຂັ້ນນ້ອຍ, ປານກາງ, ຫຼືໜັກ</td>
<td>Mild น้อย ນ້ອຍ....................1
Moderate ปานกลาง ປານກາງ 2
Severe หนัก ໜັກ....................3</td></tr>
</table>

a. Did you feel comfortable talking about your health? ท่านรู้สึกสบายใจ (หรืออึดอัดใจ) ในการพูดคุยเรื่องสุขภาพหรือไม่ ທ່ານຮູ້ສຶກສະບາຍໃຈ (ຫຼືອຶດອັດໃຈ) ໃນການລົມເລື່ອງສຸຂະພາບຫຼືບໍ່

b. Do you think these questions are too hard? ท่านรู้สึกว่าคำถามเหล่านี้ยากเกินไปหรือไม่ ທ່ານຮູ້ສຶກວ່າຄຳຖາມເຫຼົ່ານີ້ຍາກເກີນໄປຫຼືບໍ່

c. How do you rank mild, moderate, and severe? What do those words mean to you? ท่านใช้อะไรเป็นเครื่องบ่งบอกว่าอาการป่วยของท่านน้อย ปานกลาง หรือหนัก, ในความคิดของท่าน น้อย ปานกลาง และหนัก หมายความว่าอย่างไร ທ່ານໃຊ້ຫຍັງເປັນເຄື່ອງບົ່ງບອກອາການເຈັບເປັນຂອງທ່ານ ນ້ອຍ ປານກາງ ຫຼືໜັກ, ໃນຄວາມຄິດຂອງທ່ານ ໜ້ອຍ ປານກາງແລະໜັກ ໝາຍຄວາມວ່າແນວໃດ

9. The next set of questions still relates to the same illness. คำถามชุดต่อไปยังคงเกี่ยวกับอาการป่วยครั้งเดียวกับคำถามก่อนหน้า ຄຳຖາມຊຸດຕໍ່ໄປຍັງກ່ຽວກັບອາການບໍ່ສະບາຍເຊັ່ນດຽວກັບຄຳຖາມກ່ອນໜ້ານີ້

For peer review only

Can you please explain the stages of the treatment? I will ask you step-by-step what you did, starting from the moment you first detected the condition. กรุณาอธิบายขั้นตอนการรักษาทุกขั้นตอนระหว่างการป่วยครั้งนี้ เราขอให้ท่านเล่าให้เราฟังทีละขั้นตอนตั้งแต่ท่านเริ่มรู้สึกมีอาการป่วย ກະລຸນາອະທິບາຍຂັ້ນຕອນການປິ່ນປົວທຸກຂັ້ນຕອນລະຫ່ວາງການເຈັບເປັນຄັ້ງນີ້ ເຮົາຂໍໃຫ້ທ່ານເລົ່າໃຫ້ເຮົາຟັງເທື່ອລະຂັ້ນຕອນຕັ້ງແຕ່ຕອນທີ່ທ່ານເລີ່ມມີອາການບໍ່ສະບາຍ

[*ask respondent what he or she did first, then code answer and continue. After each column, ask: "What did you do next?" Only one activity per step. If e.g. medical treatment and then home care, first step is medical treatment, second step is home care. Repeat until respondent was cured or today otherwise.*] [*ถามคำถามเกี่ยวกับขั้นตอนการรักษาขั้นแรกสุด จากนั้นกรอกรหัสคำตอบ แล้วจึงถามคำถามต่อไป หลังจากกรอกข้อมูลในแต่ละคอลัมน์ ให้ถาม "ท่านทำอะไรต่อไปหลังจากนั้น" กรอกข้อมูลของการรักษาแต่ละชนิดต่อหนึ่งขั้นตอนเท่านั้น ตัวอย่าง: "เข้าพบแพทย์แล้วรักษาอาการที่บ้าน" ให้กรอก ขั้นที่ 1 เข้าพบแพทย์ และขั้นที่ 2 รักษาอาการที่บ้าน บันทึกขั้นตอนทุกขั้นตอนเช่นนี้จนถึงขั้นที่หายจากอาการหรือจนถึงวันที่ทำการเก็บข้อมูล*] [*ຖາມຄຳຖາມກ່ຽວກັບຂັ້ນຕອນການປິ່ນປົວຂັ້ນທຳອິດ ຈາກນັ້ນຂຽນລະຫັດຄຳຕອບ ແລ້ວຈຶ່ງຖາມຄຳຖາມຕໍ່ໄປ ຫຼັງຈາກຂຽນຂໍ້ມູນໃນແຕ່ລະຫ້ອງ ໃຫ້ຖາມ "ທ່ານເຮັດຫຍັງຕໍ່ໄປຫຼັງຈາກນັ້ນ" ຂຽນຂໍ້ມູນຂອງການປິ່ນປົວແຕ່ລະຊະນິດຕໍ່ໜຶ່ງຂັ້ນຕອນເທົ່ານັ້ນ ຕົວຢ່າງ: "ເຂົ້າພົບໝໍແລ້ວປິ່ນປົວອາການຢູ່ເຮືອນ" ໃຫ້ຂຽນຂັ້ນທີ່ 1 ເຂົ້າພົບໝໍ ແລະຂັ້ນທີ່ 2 ປົວອາການຢູ່ເຮືອນ ບັນທຶກຂັ້ນຕອນທຸກຂັ້ນຕອນແບບນີ້ຈົນເຖິງຂັ້ນທີ່ດີຈາກອາການຫຼືຈົນຮອດມື້ເກັບຂໍ້ມູນ*]

	Step 1 (detection)<br>ขั้นที่ 1 (เมื่อเริ่มรู้สึกป่วย)<br>ຂັ້ນທີ່ 1 (ເມື່ອເລີ່ມບໍ່ສະບາຍ)	Step 2<br>ขั้นที่ 2<br>ຂັ້ນທີ່ 2	[*continue until Step x when discomfort was cured or until today*] [บันทึกขั้นตอนทุกขั้นตอนในตารางนี้ตั้งแต่เริ่มป่วยจนถึงขั้นที่หายจากอาการหรือจนถึงวันที่ทำการเก็บข้อมูล] [*ບັນທຶກຂັ້ນຕອນທຸກຂັ້ນຕອນໃນຕາຕະລາງນີ້ຕັ້ງແຕ່ເລີ່ມຈັບເປັນຈົນເຖິງຫາຍດີຫຼືຈົນຮອດມື້ເກັບຂໍ້ມູນ*]
a) What kind of help or treatment did you get at this stage? ท่านมีการรักษาในขั้นตอนนี้อย่างไร ທ່ານມີການປິ່ນປົວໃນຂັ້ນຕອນນີ້ແບບໃດ<br><br>[*if unsure, specify*] [หากไม่มั่นใจ ให้กรอกรายละเอียดในหมวด "อื่นๆ"] [*ຖ້າບໍ່ໝັ້ນໃຈໃຫ້ຂຽນລາຍລະອຽດໃນໝວດ "ອື່ນໆ"*]	Ignored /did nothing ละเลย/ไม่ทำอะไร ບໍ່ສົນໃຈ/ບໍ່ເຮັດຫຍັງເລີຍ. 1<br>Self-care (sleep, rest, medicine at home) รักษาตัวเอง (นอนหลับ, พักผ่อน, ใช้ยาที่มีที่บ้าน) ປົວຕົນເອງ (ນອນ, ພັກຜ່ອນ, ໃຊ້ຢາທີ່ມີຢູ່ເຮືອນ) .... 2<br>Care from family and friends (full-time) ได้รับการรักษาจากครอบครัวหรือเพื่อน (ตลอดเวลา) ໄດ້ຮັບການປິ່ນປົວຈາກຄອບຄົວຫຼືໝູ່ (ຕະຫຼອດເວລາ)........ 3<br>Treated/consulted at a pharmacist รับการรักษา/คำปรึกษาจากเภสัชกร ຮັບການປິ່ນປົວ/ຄຳປຶກສາຈາກຜູ້ຂາຍຢາ........ 4<br>Treated/consulted at shop selling drugs รับการรักษา/คำปรึกษาจากร้านขายของชำหรือร้านค้าทั่วไปที่มีการจำหน่ายยา ຮັບການປິ່ນປົວ/ຄຳປຶກສາຈາກຮ້ານທົ່ວໄປທີ່ມີການຂາຍຢາ .... 5<br>Treated/consulted at priv. clinic/hospital รับการรักษา/คำปรึกษาจากคลินิก/โรงพยาบาลเอกชน ຮັບການປິ່ນປົວ/ຄຳປຶກສາຈາກຈາກຄລີນິກ/ໂຮງໝໍເອກະຊົນ .... 6<br>Treated/consulted at primary care unit รับการรักษา/คำปรึกษาจากสถานีอนามัยของรัฐ ຮັບການປິ່ນປົວ/ຄຳປຶກສາຈາກສະຖານີອານາໄມຂອງລັດ........ 7<br>Treated/consulted at a gvt. Hospital รับการรักษา/คำปรึกษาจากโรงพยาบาลของรัฐ ຮັບການປິ່ນປົວ/ຄຳປຶກສາຈາກຈາກໂຮງໝໍຂອງລັດ ... 8<br>Other (specify) อื่นๆ (โปรดระบุ) ອື່ນໆ (ກະລຸນາບອກ) _ 9	1<br>2<br>3<br>4<br>5<br>6<br>7<br>8<br>9	1<br>2<br>3<br>4<br>5<br>6<br>7<br>8<br>9

For peer review only - http://bmjopen.bmj.com/site/about/guidelines.xhtml

b) Where did this activity take place? ขั้นตอนการรักษานี้เกิดขึ้นที่ไหน ຂັ້ນຕອນການປິ່ນປົວນີ້ເກີດຂຶ້ນຢູ່ໃສ	At home ที่บ้าน ຢູ່ເຮືອນ....................1<br>Less than 10 min. from home น้อยกว่า 10 นาทีจากบ้าน ນ້ອຍກ່ວາ 10 ນາທີຈາກເຮືອນ....................2<br>10 to 29 min. 10 ถึง 29 นาทีจากบ้าน 10 ຫາ 29 ນາທີຈາກເຮືອນ....................3<br>30 to 59 min. 30 ถึง 59 นาทีจากบ้าน 30 ຫາ 59 ນາທີຈາກເຮືອນ....................4<br>60 to 119 min. 60 ถึง 119 นาทีจากบ้าน 60 ຫາ 119 ນາທີຈາກເຮືອນ....................5<br>2 hours or more from home 2 ชั่วโมงหรือมากกว่านั้น ສອງຊົ່ວໂມງຫຼືຫຼາຍກ່ວາ....................6	1<br>2<br>3<br>4<br>5<br>6	1<br>2<br>3<br>4<br>5<br>6
c) How did you get to the place of the activity? ท่านเดินทางไปสถานที่ที่ท่านทำการรักษาในขั้นตอนนี้อย่างไร ທ່ານເດີນທາງໄປສະຖານທີ່ທີ່ ປິ່ນປົວໃນຂັ້ນຕອນນີ້ແນວໃດ<br>[*select "at home" according to prior responses* เลือก "ที่บ้าน" ให้สอดคล้องกับคำตอบก่อนหน้าหากเข้าข่ายกรณีนั้น] [*ເລືອກ "ຢູ່ເຮືອນ" ໃຫ້ສອດຄ່ອງ ກັບຄໍາຕອບກ່ອນໜ້າຫາກກ່ຽວຂ້ອງກັນ*]	At home ที่บ้าน ຢູ່ເຮືອນ....................1<br>Walk เดินเท้า ຍ່າງ....................2<br>Own bicycle จักรยานของท่าน ລົດຖີບຂອງທ່ານ....................3<br>Own motorcycle / Three-wheeler รถจักรยานยนต์/รถสามล้อของท่าน ລົດຈັກ/ລົດສາມລໍ້ຂອງທ່ານ....................4<br>Own car / four-wheeler รถยนต์/ขับเคลื่อนสี่ล้อของท่าน ລົດຍົນ/ລົດໃຫຍ່ສີ່ລໍ້ຂອງທ່ານ....................5<br>Taxi or other hired ride รถแท็กซี่/รถรับจ้าง ລົດເທັກຊີ/ລົດຮັບຈ້າງ....................6<br>Public transport ขนส่งสาธารณะ ລົດຂົນສົ່ງສາທາລະນະ....................7<br>Other (specify) อื่นๆ (โปรดระบุ) ອື່ນໆ (ກະລຸນາບອກ) _ 8	1<br>2<br>3<br>4<br>5<br>6<br>7<br>8	1<br>2<br>3<br>4<br>5<br>6<br>7<br>8
d) How long did this stage last? ท่านรักษาในขั้นตอนนี้เป็นระยะเวลาเท่าใด ທ່ານປິ່ນປົວໃນຂັ້ນຕອນນີ້ເປັນໄລຍະເວລາເທົ່າໃດ<br>[*let respondent choose category; if less than one day, code "1" day*] [*ให้ผู้ให้สัมภาษณ์เลือกหน่วยเวลาเอง หากน้อยกว่าหนึ่งวันให้กรอก "1" วัน*] [*ໃຫ້ຜູ້ໃຫ້ສໍາພາດເລືອກໜ່ວຍເວລາເອງ ຖ້ານ້ອຍກ່ວາໜຶ່ງມື້ໃຫ້ຂຽນ "1" ມື້*]	Duration ระยะเวลา ໄລຍະເວລາ:<br>___ days วัน ມື້<br>___ weeks สัปดาห์ ອາທິດ<br>___ months เดือน ເດືອນ	___ days วัน ມື້<br>___ weeks สัปดาห์ ອາທິດ<br>___ months เดือน ເດືອນ	___ days วัน ມື້<br>___ weeks สัปดาห์ ອາທິດ<br>___ months เดือน ເດືອນ
e) Why did you seek this advice/treatment? เพราะเหตุใดท่านจึงเลือกรักษา/รับคำแนะนำนี้ ເປັນຫຍັງທ່ານຈຶ່ງເລືອກການປິ່ນປົວ/ຮັບຄໍາແນະນໍານີ້<br>[*Mark all that apply*] [*ตอบได้มากกว่า 1 ตัวเลือก*] [*ຕອບໄດ້ຫຼາຍກ່ວາ 1 ຕົວເລືອກ*]	This is the normal choice เป็นสิ่งที่ทำอยู่ปกติ ເປັນແນວປະຕິບັດປົກກະຕິ....................1<br>Advice from others เป็นคำแนะนำจากคนอื่น ເປັນຄໍາແນະນໍາຈາກຄົນອື່ນ....................2<br>Too busy for alternatives ไม่มีตัวเลือกอื่นเพราะไม่ว่าง ບໍ່ມີທາງເລືອກອື່ນເນື່ອງຈາກບໍ່ມີເວລາ....................3<br>No access to money/transportation มีปัญหาเรื่องเงินหรือการเดินทาง ມີບັນຫາກ່ຽວກັບເງິນຫຼືການເດີນທາງ....................4<br>Ran out of medicines ยาที่ใช้อยู่หมด ຢາທີ່ໃຊ້ຢູ່ຫມົດ....................5<br>Not feeling better พยายามรักษาด้วยวิธีอื่นแล้วไม่หาย ປິ່ນປົວໂດຍວິທີອື່ນແລ້ວບໍ່ດີຂຶ້ນ....................6<br>New/more serious symptoms อาการหนักลงกว่าเก่าหรือมีอาการอื่นปรากฏ ອາການໜັກລົງກວ່າເກົ່າຫຼືມີອາການອື່ນປາກົດເພີ່ມຂຶ້ນ....................7<br>Other (specify) อื่นๆ (โปรดระบุ) ອື່ນໆ (ກະລຸນາບອກ) _ 8	1<br>2<br>3<br>4<br>5<br>6<br>7<br>8	1<br>2<br>3<br>4<br>5<br>6<br>7<br>8

f) Can you please name or describe all the medicines that you received or were prescribed during this step? กรุณาระบุชื่อหรืออธิบายลักษณะของยาทั้งหมดที่ท่านได้รับจากการรักษาขั้นตอนนี้ ກະລຸນາບອກຊື່ຫຼືອະທິບາຍລັກສະນະຂອງຢາທີ່ທ່ານໄດ້ຮັບຈາກການປິ່ນປົວຂັ້ນຕອນນີ້<br>[*leave blank if no medicine received*] [*include medicine stored at home if "self-care at home"*] [*continue for all medicines received, then complete Questions g to k for each medicine individually*] *[เว้นช่องว่างหากผู้ให้สัมภาษณ์ไม่ได้รับยาจากการรักษาในขั้นตอนนี้] [รวมถึงยาที่มีเก็บไว้ที่บ้านด้วยหากผู้ให้สัมภาษณ์รักษาตัวเองอยู่ที่บ้าน] [บันทึกยาทุกชนิดที่ผู้ให้สัมภาษณ์ได้รับ จากนั้นกรอกข้อมูลในคำถาม g ถึง k สำหรับยาแต่ละชนิด] [ຈື່ງຊ່ອງຫ່ວາງຖ້າຜູ້ໃຫ້ສໍາພາດບໍ່ໄດ້ຮັບຢາຈາກການປິ່ນປົວໃນຂັ້ນຕອນນີ້] [ຣວມຢາທີ່ມີເກັບຢູ່ເຮືອນດ້ວຍ ຖ້າຫາກຜູ້ໃຫ້ສໍາພາດປົວຕົນເອງຢູ່ເຮືອນ] [ບັນທຶກຢາທຸກຊະນິດທີ່ຜູ້ໃຫ້ສໍາພາດໄດ້ຮັບ ຈາກນັ້ນຕື່ມຂໍ້ມູນໃສ່ໃນຄໍາຖາມ g ເຖິງ k ສໍາລັບຢາແຕ່ລະຊະນິດ]*	Medicine 1 ยาชนิดที่ 1 ຢາຊະນິດທີ່ 1:<br>Name/description ชื่อ/คำอธิบาย ຊື່/ຄໍາອະທິບາຍ:<br>_______________<br>Medicine 2 ยาชนิดที่ 2 ຢາຊະນິດທີ່ 2:<br>Name/description ชื่อ/คำอธิบาย ຊື່/ຄໍາອະທິບາຍ:<br>_______________<br>Medicine 3 ยาชนิดที่ 3 ຢາຊະນິດທີ່ 3:<br>Name/description ชื่อ/คำอธิบาย ຊື່/ຄໍາອະທິບາຍ:<br>_______________	Medicine 1 ยาชนิดที่ 1 ຢາຊະນິດທີ່ 1<br>Medicine 2 ยาชนิดที่ 2 ຢາຊະນິດທີ່ 2<br>Medicine 3 ยาชนิดที่ 3 ຢາຊະນິດທີ່ 3	Medicine 1 ยาชนิดที่ 1 ຢາຊະນິດທີ່ 1<br>Medicine 2 ยาชนิดที่ 2 ຢາຊະນິດທີ່ 2<br>Medicine 3 ยาชนิดที่ 3 ຢາຊະນິດທີ່ 3
[*for each medicine individually*] [สำหรับยาแต่ละชนิด] *[ສໍາລັບຢາແຕ່ລະຊະນິດ]*<br>g) For how long did you take the medicine? ท่านใช้ยาชนิดนี้อยู่นานเท่าใด ທ່ານໃຊ້ຢາຊະນິດນີ້ດົນປານໃດ<br>[*let respondent choose category; if more than one repeated episode, indicate total duration*] *[ให้ผู้ให้สัมภาษณ์เลือกหน่วยเวลาเอง หากมีการใช้ยาตัวเดียวกันหลายครั้ง ให้ผู้สัมภาษณ์รวมระยะเวลาทั้งหมด] [ໃຫ້ຜູ້ໃຫ້ສໍາພາດເລືອກໜ່ວຍເວລາເອງ ຖ້າມີການໃຊ້ຕົວຢາຊະນິດດຽວກັນຫຼາຍເທື່ອໃຫ້ຜູ້ສໍາພາດລວມໄລຍະເວລາທັງໝົດ]*	Duration ระยะเวลา ໄລຍະເວລາ:<br>___ days วัน ມື້<br>___ weeks สัปดาห์ ອາທິດ<br>___ months เดือน ເດືອນ	___ days วัน ມື້<br>___ weeks สัปดาห์ ອາທິດ<br>___ months เดือน ເດືອນ	___ days วัน ມື້<br>___ weeks สัปดาห์ ອາທິດ<br>___ months เดือน ເດືອນ
h) How often per day did you take the medicine? ท่านใช้ยาชนิดนี้วันละกี่ครั้ง ທ່ານໃຊ້ຢາຊະນິດນີ້ມືລະຈັກເທື່ອ<br>[*calculate into daily use according to respondent's chosen frequency*]<br>[คำนวณเป็นจำนวนครั้งต่อวันจากคำตอบของผู้ให้สัมภาษณ์]<br>*[ຄິດໄລ່ເປັນຈໍານວນເທື່ອຕໍ່ມື້ຈາກຄໍາຕອບຂອງຜູ້ໃຫ້ສໍາພາດ]*	Frequency ความถี่ ຄວາມຖີ່: ___ times daily ครั้งต่อวัน ເທື່ອຕໍ່ມື້	___ times daily ครั้งต่อวัน ເທື່ອຕໍ່ມື້	___ times daily ครั้งต่อวัน ເທື່ອຕໍ່ມື້

i) What dosage did you normally take? ท่านใช้ยาชนิดนี้ในปริมาณเท่าใดต่อหนึ่งครั้ง ທ່ານໃຊ້ຢາຊະນິດນີ້ປະລິມານເທົ່າໃດຕໍ່ໜຶ່ງເທື່ອ<br>[*let respondent choose category according to type of medicine*] [*ให้ผู้ให้สัมภาษณ์เลือกหน่วยปริมาณยาเอง โดยขึ้นอยู่กับชนิดของยา*] [*ໃຫ້ຜູ້ໃຫ້ສໍາພາດເລືອກໜ່ວຍປະລິມານຢາເອງ ໂດຍຂຶ້ນຢູ່ກັບຊະນິດຂອງຢາ*]	Dosage ปริมาณ ຄວາມຖີ່:<br>___ tablets / capsules<br>เม็ด/แคปซูล ເມັດ/ແຄບຊຸນ<br>___ drops (for liquid medicine)<br>หยด (สำหรับยาน้ำ) ຢອດ (ສໍາລັບຢານ້ຳ)<br>___ spoons (for liquid medicine)<br>ช้อน (สำหรับยาน้ำ) ບ່ວງ (ສໍາລັບຢານ້ຳ)<br>per time administered ต่อครั้งที่ใช้ ຕໍ່ເທື່ອທີ່ໃຊ້	___ tablets เม็ด ເມັດ<br>___ drops หยด ຢອດ<br>___ spoons ช้อน ບ່ວງ	___ tablets เม็ด ເມັດ<br>___ drops หยด ຢອດ<br>___ spoons ช้อน ບ່ວງ
j) Did you take the medicine exactly as it was recommended to you by the person who prescribed/sold them? ท่านใช้ยาชนิดนี้ตามคำแนะนำของผู้สั่งจ่าย/จำหน่ายหรือไม่ ທ່ານໃຊ້ຢາຊະນິດນີ້ຕາມຄໍາແນະນໍາຈາກໝໍ/ຜູ້ຂາຍຢາຫຼືບໍ່	Yes ใช่ ແມ່ນ....1<br>No ไม่ใช่ ບໍ່ແມ່ນ....2<br>Did not receive advice ไม่ได้รับคำแนะนำ ບໍ່ໄດ້ຮັບຄໍາແນະນໍາ....9<br>Don't know ไม่ใช่ ບໍ່ແມ່ນ....99	1<br>2<br>9<br>99	1<br>2<br>9<br>99
k) Did you finish the medicine? ท่านใช้ยาชนิดนี้จนหมดหรือไม่ ທ່ານໃຊ້ຢາຊະນິດນີ້ຈົນໝົດຫຼືບໍ່	Yes ใช่ ແມ່ນ.... 1<br>No ไม่ใช่ ບໍ່ແມ່ນ.... 2	1<br>2	1<br>2

Have you now recovered from the illness/accident? ท่านฟื้นตัวจากอาการป่วย/อุบัติเหตุแล้วหรือไม่ ທ່ານດີຈາກອາການເຈັບເປັນ/ອຸບັດຕິເຫດແລ້ວບໍ່?	Yes ใช่ ແມ່ນ....1<br>No ไม่ใช่ ບໍ່ແມ່ນ....2

Was anybody of your personal relationships involved in providing advice or help during the illness? ท่านได้รับคำปรึกษาหรือความช่วยเหลือในด้านใดๆ ก็ตามจากบุคคลที่เกี่ยวข้องกับท่านระหว่างการป่วยครั้งนี้หรือไม่ ທ່ານໄດ້ຮັບຄໍາປຶກສາຫຼືຄວາມຊ່ວຍເຫຼືອໃນດ້ານໃດໆ ກໍ່ຕາມຈາກບຸກຄົນທີ່ກ່ຽວຂ້ອງກັບທ່ານລະຫ່ວາງການເຈັບເປັນຄັ້ງນີ້ຫຼືບໍ່

[*record up to ten names*] [*กรอกชื่อของบุคคลที่มีความเกี่ยวข้องระหว่างอาการป่วยไม่เกิน 10 คน*] [*ຂຽນຊື່ຂອງບຸກຄົນທີ່ມີຄວາມກ່ຽວຂ້ອງລະຫ່ວາງອາການເຈັບປ່ວຍບໍ່ຫຼາຍກ່ວາ 10 ຄົນ*]

	a) What is the name of the person? ชื่อ ຊື່	b) How is this person related to you? ความสัมพันธ์กับท่าน ຄວາມສໍາພັນກັບທ່ານ?<br>[*give examples if respondent is unsure about answer categories*] [*หากผู้ให้สัมภาษณ์ไม่มั่นใจในหมวดคำตอบ ให้ยกตัวอย่างคำตอบได้*] [*ຖ້າຜູ້ໃຫ້ສໍາພາດບໍ່ໝັ້ນໃຈໃນໝວດຄໍາຕອບ ໃຫ້ຍົກຕົວຢ່າງຄໍາຕອບໄດ້*]	c) What kind of support was provided? *ท่านได้รับความช่วยเหลือจากบุคคลนี้อย่างไร* ທ່ານໄດ້ຮັບການຊ່ວຍເຫຼືອຈາກບຸກຄົນເຫຼົ່ານີ້ແນວໃດ?<br>[*mark all that apply*] [*ตอบได้มากกว่า 1 ตัวเลือก*] [*ຕອບໄດ້ຫຼາຍກ່ວາ 1 ຕົວເລືອກ*]

For peer review only

Contact 1 คนที่ 1 ຄົນທີ່ 1	Name ชื่อ ຊື່:<br>_______	Spouse คู่สมรส ຄູ່ແຕ່ງດອງ ....1<br>Parent บิดามารดา ພໍ່ແມ່ ....2<br>Child บุตร ລູກ.................................3<br>Sibling พี่น้อง ອ້າຍນ້ອງ.......................4<br>Other relative ญาติ ພີ່ນ້ອງອື່ນໆ...........5<br>Neighbour เพื่อนบ้าน ເພື່ອນບ້ານ............6<br>Friend (if not neighbour) เพื่อน (ที่ไม่ใช่เพื่อนบ้าน) ໝູ່ (ທີ່ບໍ່ແມ່ນເພື່ອນບ້ານ)..........7<br>Other villager ชาวบ้านคนอื่นๆ ຊາວບ້ານຄົນອື່ນໆ...8<br>Other (specify) อื่นๆ (โปรดระบุ) ອື່ນໆ (ກະລຸນາບອກ) _ 9	Providing healthcare/attending ให้การรักษา/การดูแล ໃຫ້ການເບິ່ງແຍງ...11<br>Providing advice ให้คำปรึกษา ໃຫ້ຄໍາປຶກສາ ....12<br>Providing medicine ให้ยา ໃຫ້ຢາ ....13<br>Lending/granting money ให้หรือให้ยืมเงิน ໃຫ້ຫຼືໃຫ້ຢືມເງິນ...21<br>Transportation/Lending vehicle อำนวยความสะดวกในด้านการเดินทาง/ให้ยืมพาหนะ ສ້າງຄວາມສະດວກດ້ານການເດີນທາງ/ໃຫ້ຢືມຍານພາຫະນະ...22<br>Contacting family/friends ติดต่อครอบครัว/เพื่อน ຕິດຕໍ່ຄອບຄົວ/ໝູ່...23<br>Providing food สนับสนุนอาหาร ສະໜັບສະໜູນອາຫານ ....31<br>Helping with children/housework ช่วยดูแลลูกหลาน/งานบ้าน ຊ່ວຍເບິ່ງແຍງລູກຫລານຫຼືເດັກນ້ອຍ/ວຽກບ້ານ ....32<br>Helping with jobs/agriculture work (feeding animals/tending crops/covering shifts, etc.) ช่วยเหลือเกี่ยวกับอาชีพ/การเกษตร (ช่วยดูแลสัตว์เลี้ยง/งานเพาะปลูก/ทำงานแทน) ຊ່ວຍເຮັດວຽກ/ການກະສິກໍາ (ຊ່ວຍເບິ່ງແຍງສັດລ້ຽງ/ເຮັດໄຮ່ເຮັດນາ/ເຮັດວຽກແທນ)...33<br>Other (specify) อื่นๆ (โปรดระบุ) ອື່ນໆ (ກະລຸນາບອກ) _ ..99
Contact 2 คนที่ 2 ຄົນທີ່ 2	Name ชื่อ ຊື່	1 2 3 4 5 6 7 8 9	11 12 13 21 22 23 31 32 33 99
[*continue until Contact x – maximum 10 contacts*] [*บันทึกบุคคลที่มีความเกี่ยวข้องระหว่างการเจ็บป่วยทุกคน ไม่เกิน 10 คน*] [*ບັນທຶກບຸກຄົນທີ່ມີຄວາມກ່ຽວຂ້ອງລະຫ່ວາງອາການເຈັບປ່ວຍທຸກຄົນ ບໍ່ຫຼາຍກ່ວາ 10ຄົນ*]	Name ชื่อ ຊື່	1 2 3 4 5 6 7 8 9	11 12 13 21 22 23 31 32 33 99

a. Do you think these questions are too hard? ท่านรู้สึกว่าคำถามเหล่านี้ยากเกินไปหรือไม่ ທ່ານຮູ້ສຶກວ່າຄໍາຖາມເຫຼົ່ານີ້ຍາກເກີນໄປຫຼືບໍ່

b. Did you find it tedious to go through the illness in such detail? เป็นเรื่องน่าเบื่อหรือไม่ที่ท่านต้องอธิบายอาการป่วยของท่านโดยละเอียด ເປັນເລື່ອງໜ້າເບື່ອຫຼືບໍ່ທີ່ທ່ານຕ້ອງມາອະທິບາຍອາການບໍ່ສະບາຍຂອງທ່ານຢ່າງລະອຽດ

c. How well do you remember the medicines that you used during that illness? ท่านสามารถจดจำยาที่ท่านใช้ระหว่างการป่วยได้ดีเพียงใด ທ່ານສາມາດຈື່ຢາທີ່ທ່ານໃຊ້ລະຫ່ວາງຕອນເຈັບປ່ວຍ ໄດ້ດີພຽງໃດ

d. Did you think about anything in particular to help you recall the medicines? ที่ท่านนึกถึงอะไรเพื่อเป็นตัวช่วยในการทวนความจำเกี่ยวกับยาเหล่านี้ ທ່ານນຶກເຖິງຫຍັງເປັນຕົວຊ່ວຍໃນການຈື່ກ່ຽວກັບຢາເຫຼົ່ານີ້

e. How do you indicate that the symptoms are cured? Would you stop taking medicines then? What if the doctor's instruction is to complete the dose? ท่านสามารถบอกได้อย่างไรว่าอาการป่วยของท่านหายแล้ว หากอาการป่วยของท่านหายท่านจะหยุดรับประทานยาทันทีหรือไม่ หากแพทย์แนะนำให้ท่านใช้ยาจนหมดแต่อาการของท่านหายแล้วท่านยังจะปฏิบัติตามคำสั่งแพทย์หรือไม่ ທ່ານສາມາດບອກໄດ້ແນວໃດວ່າອາການເຈັບເປັນຂອງທ່ານດີແລ້ວ ຖ້າອາການເຈັບເປັນຂອງທ່ານດີ ທ່ານຈະຢຸດກິນຢາທັນທີຫຼືບໍ່ ຖ້າໝໍແນະນໍາໃຫ້ທ່ານໃຊ້ຢາຈົນໝົດແຕ່ອາການຂອງທ່ານດີແລ້ວທ່ານຍັງຈະປະຕິບັດຕາມຄໍາສັ່ງໝໍຫຼືບໍ່

10. The next question deals with medicine. Could you please tell me what is going through your mind while you are answering the question? คำถามต่อไปจะเป็นคำถามเกี่ยวกับยารักษาโรค เราขอให้ท่านบอกเราว่าท่านนึกถึงอะไรระหว่างการตอบคำถามเหล่านี้ ຄໍາຖາມຕໍ່ໄປຈະເປັນຄໍາຖາມກ່ຽວກັບຢາປົວພະຍາດ ເຮົາຂໍໃຫ້ທ່ານບອກເຮົາວ່າທ່ານນຶກເຖິງຫຍັງລະຫ່ວາງການຕອບຄໍາຖາມເຫຼົ່ານີ້

For peer review only - http://bmjopen.bmj.com/site/about/guidelines.xhtml

I would now like to ask you your opinion about medicine. There are no right or wrong answers, I only want to understand what you think. เราอยากทราบความคิดเห็นของท่านเกี่ยวกับการใช้ยา คำถามเหล่านี้ไม่มีคำตอบที่ถูกหรือผิด เราเพียงต้องการรู้ว่าท่านคิดอย่างไร ເຮົາຢາກຮູ້ຄວາມຄິດເຫັນຂອງທ່ານກ່ຽວກັບການໃຊ້ຢາ ຄຳຖາມເຫຼົ່ານີ້ບໍ່ມີຄຳຕອບຖືກຫຼືຜິດ ເຮົາພຽງຕ້ອງການຮູ້ວ່າທ່ານຄິດແນວໃດ

Consider the following medicines – they are commonly referred to as “antibiotics.”
พิจารณายาเหล่านี้ซึ่งเป็นยาที่ถูกรู้จักในนาม “ยาปฏิชีวนะ” ພິຈາລະນາຢາເຫຼົ່ານີ້ເຊິ່ງເປັນຢາທີ່ຮູ້ຈັກກັນໃນນາມ “ຢາຕ້ານເຊື້ອ”

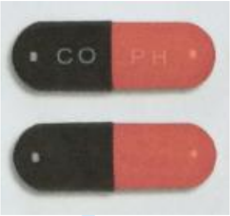

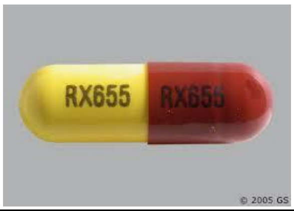

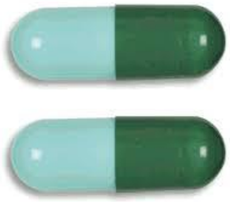

Would you buy this medicine if you think you need it but cannot get it prescribed by a doctor? หากท่านไม่ได้รับยานี้หลังจากเข้าพบแพทย์แม้ท่านรู้สึกว่าท่านควรรับประทานยานี้ ท่านจะซื้อยานี้จากแหล่งอื่นหรือไม่ ຖ້າທ່ານບໍ່ໄດ້ຮັບຢານີ້ຫຼັງຈາກເຂົ້າພົບໝໍເຖິງທ່ານຈະຮູ້ສຶກວ່າທ່ານຄວນກິນຢານີ້ ທ່ານຈະຊື້ຢານີ້ຈາກແຫຼ່ງອື່ນຫຼືບໍ່	Yes ใช่ ແມ່ນ .... 1<br>No ไม่ใช่ ບໍ່ແມ່ນ .... 2<br>I don't know ไม่ทราบ ບໍ່ຮູ້ 99
Would you try other remedies such as sponge bath, herbs, or paracetamol before using this medicine? ท่านจะลองใช้วิธีการรักษาอื่น เช่น เช็ดตัว สมุนไพร หรือ พาราเซตามอลก่อนใช้ยานี้หรือไม่ ທ່ານຈະພະຍາຍາມປິ່ນປົວໂດຍວິທີການອື່ນ ເຊັ່ນ ເຊັດຕົວ ຢາຮາກໄມ້ ຫຼືພາຣາເຊຕາມອນກ່ອນໃຊ້ຢານີ້ຫຼືບໍ່	Yes ใช่ ແມ່ນ .... 1<br>No ไม่ใช่ ບໍ່ແມ່ນ .... 2<br>I don't know ไม่ทราบ ບໍ່ຮູ້ 99
If you had to take this medicine and your symptoms disappear after 2 days, would you finish a complete course of 7 days or stop taking it immediately? หากท่านจำเป็นต้องรับประทานยานี้และอาการของท่านดีขึ้นภายใน 2 วัน ท่านจะรับประทานยาต่อจนครบ 7 วัน หรือหยุดรับประทานทันที ຖ້າຫາກທ່ານມີຄວາມຈຳເປັນຕ້ອງກິນຢານີ້ເພື່ອການປິ່ນປົວ ແລະອາການຂອງທ່ານດີຂຶ້ນພາຍໃນສອງມື້ ທ່ານຈະກິນຢາຕໍ່ຈົນສຸດ 7 ມື້ ຫຼືຢຸດກິນທັນທີ	Finish course รับประทานต่อจนหมด ກິນຢາຕໍ່ຈົນສຸດ .... 1<br>Stop taking หยุดรับประทาน ຢຸດກິນທັນທີ .... 2<br>I don't know ไม่ทราบ ບໍ່ຮູ້ 99
Do you think the way other people use this medicine affects its efficacy on you? ท่านคิดว่าการใช้ยานี้ของคนอื่นมีผลกระทบต่อผลลัพธ์ของการใช้ยานี้ของท่านหรือไม่ ທ່ານຄິດວ່າການໃຊ້ຢານີ້ຂອງຄົນອື່ນມີຜົນກະທົບກັບສະມັດຖະພາບຂອງການໃຊ້ຢານີ້ຂອງທ່ານຫຼືບໍ່	Yes ใช่ ແມ່ນ .... 1<br>No ไม่ใช่ ບໍ່ແມ່ນ .... 2<br>I don't know ไม่ทราบ ບໍ່ຮູ້ 99

a. As I have said before, these survey questions are not intended to measure your knowledge. Did you feel tested or pressured being asked these questions at all? เราได้บอกท่านข้างต้นเกี่ยวกับคำถามเหล่านี้ที่ไม่ได้ถูกออกแบบเพื่อเป็นข้อสอบวัดความรู้ ท่านรู้สึกถูกทดสอบหรือกดดันในการตอบคำถามเหล่านี้หรือไม่ ເຮົາໄດ້ບອກທ່ານຂັ້ນຕົ້ນກ່ຽວກັບຄຳຖາມເຫຼົ່ານີ້ທີ່ບໍ່ໄດ້ອອກແບບເພື່ອເປັນບົດເສັງວັດແທກຄວາມຮູ້ ທ່ານຮູ້ສຶກຖືກທົດສອບຫຼືກົດດັນໃນການຕອບຄຳຖາມເຫຼົ່ານີ້ຫຼືບໍ່
b. Have you heard of antibiotics? ท่านเคยได้ยินเกี่ยวกับยาปฏิชีวนะหรือไม่ ທ່ານເຄີຍໄດ້ຍິນກ່ຽວກັບຢາຕ້ານເຊື້ອຫຼືບໍ່
c. Other than the term “antibiotics,” how do you normally call these medicines? นอกจากคำว่า “ยาปฏิชีวนะ” ท่านเรียกยาชนิดนี้ได้ว่าอะไรอีกบ้าง ນອກຈາກຄຳວ່າ ຢາຕ້ານເຊື້ອ ທ່ານເອີ້ນຢາຊະນິດນີ້ໄດ້ວ່າຫຍັງອີກແດ່
d. Do you think other villagers will associate the pictures with other types of medicine? ท่านคิดว่าชาวบ้านคนอื่นๆ จะนึกถึงยาชนิดอื่นหรือไม่เมื่อเห็นภาพเหล่านี้ ທ່ານຄິດວ່າຊາວບ້ານຄົນອື່ນໆ ຈະນຶກເຖິງຢາຊະນິດອື່ນຫຼືບໍ່ເມື່ອເຫັນພາບເຫຼົ່ານີ້

For peer review only - http://bmjopen.bmj.com/site/about/guidelines.xhtml

Part V – Household-Related Survey Questions ส่วนที่ 5 – คำถามเกี่ยวกับครัวเรือน ສ່ວນທີ່ 5 - ຄຳຖາມກ່ຽວກັບຄົວເຮືອນ

Thank you. We now come to the last part. ขอบคุณครับ/ค่ะ ส่วนต่อไปเป็นส่วนสุดท้ายของการสัมภาษณ์ ຂອບໃຈ ສ່ວນຕໍ່ເປັນເປັນສ່ວນສຸດທ້າຍຂອງການສຳພາດ

11. The following questions relate to your household. คำถามต่อไปนี้เป็นคำถามเกี่ยวกับครัวเรือนของท่าน ຄຳຖາມຕໍ່ໄປນີ້ເປັນຄຳຖາມກ່ຽວກັບຄົວເຮືອນຂອງທ່ານ

I will now ask you for some items in your household. Please tell me… ต่อไปเราจะถามท่านเกี่ยวกับสิ่งของที่ท่านครอบครองในครัวเรือน ຕໍ່ໄປເຮົາຈະຖາມກ່ຽວກັບສິ່ງຂອງທີ່ທ່ານຄອບຄອງໃນຄົວເຮືອນ		Number of items in household นวนของสิ่งของที่มีภายในครัวเรือน ຈຳນວນຂອງສິ່ງຂອງທີ່ມີພາຍໃນຄົວເຮືອນ:
	Have you got a *functioning* **radio** in your household? If so, how many? ท่านมี**วิทยุ**ที่ใช้งานได้อยู่ในครัวเรือนของท่านหรือไม่ ถ้ามี กี่เครื่อง ທ່ານມີ**ວິທະຍຸ**ທີ່ໃຊ້ງານໄດ້ໃນຄົວເຮືອນຂອງທ່ານຫຼືບໍ່ ຖ້າມີ ຈັກອັນ	________
	Have you got a *functioning* **TV** in your household? If so, how many? ท่านมี**โทรทัศน์**ที่ใช้งานได้อยู่ในครัวเรือนของท่านหรือไม่ ถ้ามี กี่เครื่อง ທ່ານມີ**ທໍລະທັດ**ທີ່ໃຊ້ງານໄດ້ຢູ່ໃນຄົວເຮືອນຂອງທ່ານຫຼືບໍ່ ຖ້າມີ ຈັກອັນ	________
	Have you got a *functioning* **rice cooker** in your household? If so, how many? ท่านมี**หม้อหุงข้าว**ที่ใช้งานได้อยู่ในครัวเรือนของท่านหรือไม่ ถ้ามี กี่เครื่อง ທ່ານມີ**ໝໍ້ຫຸງເຂົ້າ**ທີ່ໃຊ້ງານໄດ້ຢູ່ໃນຄົວເຮືອນຂອງທ່ານຫຼືບໍ່ ຖ້າມີ ຈັກອັນ	________
	Have you got a *functioning* **landline telephone** in your household? If so, how many? ท่านมี**โทรศัพท์บ้าน**ที่ใช้งานได้อยู่ในครัวเรือนของท่านหรือไม่ ถ้ามี กี่เครื่อง ທ່ານມີ**ໂທລະສັບຕັ້ງໂຕະ**ທີ່ໃຊ້ງານໄດ້ຢູ່ໃນຄົວເຮືອນຂອງທ່ານຫຼືບໍ່ ຖ້າມີ ຈັກອັນ	________
	Have you got a *functioning* **mobile phone** in your household? If so, how many? ท่านมี**โทรศัพท์มือถือ**ที่ใช้งานได้อยู่ในครัวเรือนของท่านหรือไม่ ถ้ามี กี่เครื่อง ທ່ານມີ**ໂທລະສັບມືຖື**ທີ່ໃຊ້ງານໄດ້ຢູ່ໃນຄົວເຮືອນຂອງທ່ານຫຼືບໍ່ ຖ້າມີ ຈັກອັນ	________
	Have you got a *functioning* **computer** in your household? If so, how many? ท่านมี**คอมพิวเตอร์**ที่ใช้งานได้อยู่ในครัวเรือนของท่านหรือไม่ ถ้ามี กี่เครื่อง ທ່ານມີ**ຄອມພິວເຕີ**ທີ່ໃຊ້ງານໄດ້ຢູ່ໃນຄົວເຮືອນຂອງທ່ານຫຼືບໍ່ ຖ້າມີ ຈັກອັນ	________
	Have you got a *functioning* **bicycle** in your household? If so, how many? ท่านมี**รถจักรยาน**ที่ใช้งานได้อยู่ในครัวเรือนของท่านหรือไม่ ถ้ามี กี่คัน ທ່ານມີ**ລົດຖີບ**ທີ່ໃຊ້ງານໄດ້ຢູ່ໃນຄົວເຮືອນຂອງທ່ານຫຼືບໍ່ ຖ້າມີ ຈັກຄັນ	________
	Have you got a *functioning* **scooter, motorcycle, or tricycle** in your household? If so, how many? ท่านมี**จักรยานยนต์หรือรถสามล้อ**ที่ใช้งานได้อยู่ในครัวเรือนของท่านหรือไม่ ถ้ามี กี่คัน ທ່ານມີ**ລົດຈັກຫຼືລົດສາມລໍ້**ທີ່ໃຊ້ງານໄດ້ຢູ່ໃນຄົວເຮືອນຂອງທ່ານຫຼືບໍ່ ຖ້າມີ ຈັກຄັນ	________
	Have you got a *functioning* **car or truck** in your household? If so, how many? ท่านมี**รถยนต์ รถกระบะ หรือรถบรรทุก**ที่ใช้งานได้อยู่ในครัวเรือนของท่านหรือไม่ ถ้ามี กี่คัน ທ່ານມີ**ລົດໃຫຍ່ ລົດກະບະ ຫຼືລົດບັນທຸກ**ທີ່ໃຊ້ງານໄດ້ຢູ່ໃນຄົວເຮືອນຂອງທ່ານຫຼືບໍ່ ຖ້າມີ ຈັກຄັນ	________
	Have you got a *functioning* **tractor** in your household? If so, how many? ท่านมี**รถแทรกเตอร์หรือรถไถนา**ที่ใช้งานได้อยู่ในครัวเรือนของท่านหรือไม่ ถ้ามี กี่คัน ທ່ານມີ**ລົດໄຖນາ**ທີ່ໃຊ້ງານໄດ້ຢູ່ໃນຄົວເຮືອນຂອງທ່ານຫຼືບໍ່ ຖ້າມີ ຈັກຄັນ	________
	Have you got a *functioning* **refrigerator or freezer** in your household? If so, how many? ท่านมี**ตู้เย็นหรือตู้แช่เข็ง**ที่ใช้งานได้อยู่ในครัวเรือนของท่านหรือไม่ ถ้ามี กี่ตู้ ທ່ານມີ**ຕູ້ເຢັນຫຼືຕູ້ແຊ່ແຂງ**ທີ່ໃຊ້ງານໄດ້ຢູ່ໃນຄົວເຮືອນຂອງທ່ານຫຼືບໍ່ ຖ້າມີ ຈັກຕູ້	________

a. Do you feel comfortable (or uncomfortable) recalling these points for your household? ท่านรู้สึกสบายใจ (หรืออึดอัดใจ) ในการนึกถึงข้าวของเครื่องใช้เหล่านี้ในครัวเรือนของท่านหรือไม่ ທ່ານຮູ້ສຶກສະບາຍໃຈ (ຫຼືອຶດອັດໃຈ) ໃນການນຶກເຖິງເຄື່ອງໃຊ້ເຫຼົ່ານີ້ໃນຄົວເຮືອນຂອງທ່ານຫຼືບໍ່

b. Are there some common household items that you normally see in your village? ข้าวของเครื่องใช้ที่เรากล่าวมาข้างต้นเป็นสิ่งที่คนในหมู่บ้านนี้ใช้กันทั่วไปหรือไม่ ເຄື່ອງໃຊ້ທີ່ເຮົາກ່າວມາໃນຂັ້ນຕົ້ນເປັນສິ່ງທີ່ຄົນໃນໝູ່ບ້ານນີ້ໃຊ້ທົ່ວໄປຫຼືບໍ່

c. How would you distinguish whether somebody in your village is wealthy or not?

For peer review only - http://bmjopen.bmj.com/site/about/guidelines.xhtml

ในหมู่บ้านของท่าน ท่านสามารถแยกได้อย่างไรว่าใครรวยหรือจน ໃນໝູ່ບ້ານຂອງທ່ານ ທ່ານສາມາດແຍກໄດ້ແນວໃດວ່າໃຜລວຍຫຼືທຸກ

d. If you had to group these houses into 2 categories, rich and poor, how would you do that? [Sample pictures will be provided] หากท่านต้องจัดกลุ่มบ้านในภาพเหล่านี้ออกเป็นสองกลุ่ม ได้แก่กลุ่มรวยและกลุ่มจน ท่านจะจัดอย่างไร [แสดงบัตรภาพให้ผู้ให้สัมภาษณ์จัดกลุ่ม] ຫາກທ່ານຕ້ອງຈັດກຸ່ມເຮືອນໃນຮູບເຫຼົ່ານີ້ອອກເປັນສອງກຸ່ມໄດ້ແກ່ກຸ່ມລວຍແລະກຸ່ມທຸກ ທ່ານຈະຈັດແນວໃດ [ສະແດງບັດພາບໃຫ້ຜູ້ສຳພາດຈັດກຸ່ມ]

e. What kinds of "meals" did you have in mind when you answered the question? ตอนที่ท่านตอบคำถามเราเรื่องมื้ออาหารที่ท่านรับประทานสัปดาห์ที่แล้ว ท่านจินตนาการภาพถึงมื้ออาหารลักษณะใด ຕອນທ່ານຕອບຄຳຖາມເຮົາເລື່ອງຄາບອາຫານທີ່ທ່ານກິນໃນອາທິດທີ່ແລ້ວ ທ່ານຈິນຕະນາການພາບເຖິງຄາບອາຫານລັກຊະນະໃດ

12. And these are the last two questions. I would like to ask you once more to share your thoughts as you answer these questions. และคำถามสองคำถามสุดท้าย เราต้องการขอให้ท่านเล่าให้เราฟังอีกครั้งว่าท่านคิดอะไรบ้างระหว่างการตอบคำถามต่อไปนี้ ແລະຄຳຖາມສອງຄຳຖາມສຸດທ້າຍ ເຮົາຂໍໃຫ້ທ່ານເລົ່າໃຫ້ເຮົາຟັງອີກເທື່ອໜຶ່ງວ່າທ່ານຄິດຫຍັງແດ່ລະຫ່ວາງການຕອບຄຳຖາມເຫຼົ່ານີ້

And finally the last two questions: Have you heard about drug resistance? คำถามสองข้อสุดท้าย: ท่านเคยได้ยินเกี่ยวกับการดื้อยาหรือไม่ ຄຳຖາມສອງຂໍ້ສຸດທ້າຍ: ທ່ານເຄີຍໄດ້ຍິນກ່ຽວກັບການດື້ຢາຫຼືບໍ່	Yes เคย ເຄີຍ.. 1<br>No ไม่เคย ບໍ່ເຄີຍ 2
What do you think is drug resistance? ท่านคิดว่าการดื้อยาคืออะไร ທ່ານຄິດວ່າການດື້ຢາແມ່ນຫຍັງ	Answer relates to antibiotic or antimicrobial resistance คำตอบสอดคล้องกับการดื้อยาต้านจุลชีพ ຄຳຕອບສອດຄ່ອງກັບການດື້ຢາຕ້ານຈຸລະຊີບ..1<br>Answer relates to resistance to other types of drugs คำตอบสอดคล้องกับการดื้อยาชนิดอื่นๆ ຄຳຕອບສອງຄ່ອງກັບການດື້ຢາຊະນິດອື່ນໆ ....2<br>Answer does not relate to drug resistance คำตอบไม่สอดคล้องกับการดื้อยา ຄຳຕອບບໍ່ສອດຄ່ອງກັບການດື້ຢາ..3<br>"Don't know" ไม่ทราบ ບໍ່ຮູ້ 99

a. What your intuitive understanding of "drug resistance?" เมื่อพูดถึง "การดื้อยา" ท่านเข้าใจความหมายของคำคำนี้อย่างไร ເມື່ອເວົ້າເຖິງ "ການດື້ຢາ" ທ່ານເຂົ້າໃຈຄວາມໝາຍຂອງຄຳນີ້ແນວໃດ

b. Do you think other villagers will translate the meaning of "drug resistance" differently from you? ท่านคิดว่าชาวบ้านคนอื่นๆ จะแปลความหมายของคำว่า "ดื้อยา" แตกต่างจากท่านหรือไม่ ທ່ານຄິດວ່າຊາວບ້ານຄົນອື່ນ ຈະແປຄວາມໝາຍຂອງຄຳວ່າ "ດື້ຢາ" ແຕກຕ່າງຈາກທ່ານຫຼືບໍ່

c. Did you feel pressured answering these questions? ท่านรู้สึกกดดันในการตอบคำถามนี้หรือไม่ ທ່ານຮູ້ສຶກກົດດັນໃນການຕອບຄຳຖາມເຫຼົ່ານີ້ຫຼືບໍ່

We are coming to the end of our interview. Thank you so much for sharing your views with me. Your responses have been very helpful. การสัมภาษณ์นี้สิ้นสุดลงแล้ว คำตอบที่ท่านให้กับเราเป็นประโยชน์กับการวิจัยของเราอย่างมาก ขอบคุณสำหรับความคิดเห็นที่ท่านแบ่งปันกับเรา ການສຳພາດນີ້ສິ້ນສຸດແລ້ວ ຄຳຕອບທີ່ທ່ານໃຫ້ກັບເຮົາຈະເປັນປະໂຫຍດກັບການຄົ້ນຄ້ວາຂອງເຮົາຫຼາຍ ຂອບໃຈສຳລັບຄຳຄິດເຫັນທີ່ທ່ານແບ່ງປັນກັບເຮົາ

For peer review only - http://bmjopen.bmj.com/site/about/guidelines.xhtml

No	Topic	Item	Check	Comment
1a	Title & Abstract	(*a*) Indicate the study's design with a commonly used term in the title or the abstract	☑	Page 1
1b	Title & Abstract	(*b*) Provide in the abstract an informative and balanced summary of what was done and what was found	☑	Pages 2-3
**Introduction**				
2	Background/ rationale	Explain the scientific background and rationale for the investigation being reported	☑	See introduction, esp. page 4
3	Objectives	State specific objectives, including any prespecified hypotheses	☑	See introduction, page 5
**Methods**				
4	Study design	Present key elements of study design early in the paper	☑	Research design at beginning of methods section, page 6
5	Setting	Describe the setting, locations, and relevant dates, including periods of recruitment, exposure, follow-up, and data collection	☑	See research design section, pages 6-7; exposure and follow-up not applicable
6	Participants	Cross-sectional study—Give the eligibility criteria, and the sources and methods of selection of participants	☑	See research design and study population sections, pages 6-7
7	Variables	Clearly define all outcomes, exposures, predictors, potential confounders, and effect modifiers. Give diagnostic criteria, if applicable	☑	Key outcomes described in data collection section, pages 7-8; All variables described in Supplemental Material Table A2; diagnostic criteria not applicable
8	Data sources/ measurement	For each variable of interest, give sources of data and details of methods of assessment (measurement). Describe comparability of assessment methods if there is more than one group	☑	All variables described in Supplemental Material Table A2, data collection and translation process for the two sites described in data collection section, page 8
9	Bias	Describe any efforts to address potential sources of bias	☑	Recall biases mitigated through elicitation of behavioural sequences (pages 17-18)
10	Study size	Explain how the study size was arrived at	☑	See research design section, page 6
11	Quantitative variables	Explain how quantitative variables were handled in the analyses. If applicable, describe which groupings were chosen and why	☑	All variables described in Appendix Table A2 (incl. variable construction)
12a	Statistical methods	(*a*) Describe all statistical methods, including those used to control for confounding	☑	Data analysis section, page 9
12 b	Statistical methods	(*b*) Describe any methods used to examine subgroups and interactions	☑	Data analysis section, page 9
12c	Statistical methods	(*c*) Explain how missing data were addressed	☑	Not applicable (data complete; sub-group analysis of illness episodes indicated in data analysis section, page 9)
12 d	Statistical methods	Cross-sectional study—If applicable, describe analytical methods taking account of sampling strategy	☑	Page 9: SVY suite of commands in Stata 15
12 e	Statistical methods	Describe any sensitivity analyses	☑	Inclusion of "confirmed" and "potential" antibiotic use, data collection section, page 8
**Results**				
13a	Participants	(a) Report numbers of individuals at each stage of study—eg numbers potentially eligible, examined for eligibility, confirmed eligible, included in the study, completing follow-up, and analysed	☑	See flow diagram of sampling strategy in Figure 1, page 6
13 b	Participants	(b) Give reasons for non-participation at each stage	☑	Research design section: Non-availability of random selection, information on random replacement procedures in page 6 and selection data in Figure 1
13c	Participants	(c) Consider use of a flow diagram	☑	See flow diagram of sampling strategy in Figure 1, page 6
14a	Descriptive data	(a) Give characteristics of study participants (eg demographic, clinical, social) and information on exposures and potential confounders	☑	Appendix Table 1
14 b	Descriptive data	(b) Indicate number of participants with missing data for each variable of interest	☑	Not applicable (data complete)
15	Outcome data	Cross-sectional study—Report numbers of outcome events or summary measures	☑	Appendix Table 1
16a	Main results	(*a*) Give unadjusted estimates and, if applicable, confounder-adjusted estimates and their precision (eg, 95% confidence interval). Make clear which confounders were adjusted for and why they were included	☑	Table 2, pages 15-16
16 b	Main results	(*b*) Report category boundaries when continuous variables were categorized	☑	Not applicable
16c	Main results	(*c*) If relevant, consider translating estimates of relative risk into absolute risk for a meaningful time period	☑	Not applicable
17	Other analyses	Report other analyses done—eg analyses of subgroups and interactions, and sensitivity analyses	☑	Throughout results section, pages 10-16
**Discussion**				
18	Key results	Summarise key results with reference to study objectives	☑	Discussion/conclusion section, pages 16-17
19	Limitations	Discuss limitations of the study, taking into account sources of potential bias or imprecision. Discuss both direction and magnitude of any potential bias	☑	Discussion/conclusion section, pages 17-18
20	Interpretation	Give a cautious overall interpretation of results considering objectives, limitations, multiplicity of analyses, results from similar studies, and other relevant evidence	☑	Discussion/conclusion section, lines 18-19
21	Generalisability	Discuss the generalisability (external validity) of the study results	☑	Discussion/conclusion section, pages 17-18
**Other information**				
22	Funding	Give the source of funding and the role of the funders for the present study and, if applicable, for the original study on which the present article is based	☑	See funding declaration, page 21

For peer review only
